# Catalytic Asymmetric Cycloaddition of Olefins with In Situ Generated *N*-Boc-Formaldimine

**DOI:** 10.1021/jacs.4c13538

**Published:** 2024-11-18

**Authors:** Marian Guillén, Markus Leutzsch, Benjamin List

**Affiliations:** Max-Planck-Institut für Kohlenforschung, Kaiser-Wilhelm-Platz 1, 45470, Mülheim an der Ruhr, Germany

## Abstract

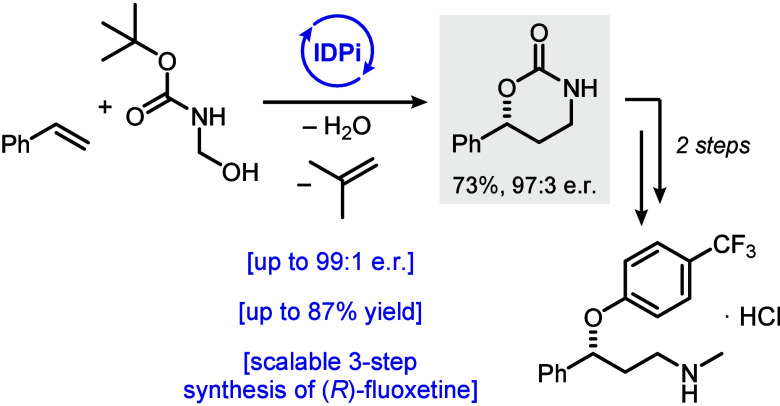

Chiral 1,3-amino alcohols are ubiquitous structural motifs in natural products and active pharmaceutical ingredients. We present a highly enantioselective, inverse-electron-demand hetero-Diels–Alder reaction of olefins with in situ generated *N*-Boc-formaldimine catalyzed by strong and confined Bro̷nsted acids. This transformation provides direct access to valuable 1,3-amino alcohols from styrenes and 1,1-disubtituted alkenes. Isotope labeling studies and kinetic analysis reveal an unusual mechanism involving an oxazinium intermediate and a catalyst order greater than one.

Readily available in bulk quantities from both petrochemical feedstocks and renewable resources, olefins are among the most versatile classes of organic compounds and capable of undergoing a plethora of transformations. The direct functionalization of olefins has proven to be particularly useful and, over the past century, led to frequently applied transformations, such as dihydroxylations and aminohydroxylations, to provide direct access to valuable 1,2-diols and amino alcohols (Figure 1a).^1−3^ Interestingly, while the corresponding 1,3-dioxygenated moieties can also be accessed from olefins via a Prins-type oxy-oxymethylation with aldehydes,^4,5^ an analogous oxy-aminomethylation of alkenes toward 1,3-amino alcohols remains elusive with only a few reports of nonasymmetric variants in the literature to date.^6−10^ Such enantiopure 1,3-amino alcohols, however, are extremely useful building blocks toward pharmaceuticals, especially for the synthesis of marketed blockbuster antidepressants, such as (*S*)-duloxetine, (*R*)-fluoxetine, and (*R*)-atomoxetine (Figure 1b). In this context, enantiomerically enriched 1,3-amino alcohols have been synthesized via asymmetric hydrogenation with transition metal catalysts,^11−13^ hydroamination of allylic alcohols,^14−16^ intermolecular C–H amination of prefunctionalized substrates,^17−19^ and aza-aldol reactions.^20,21^ Nevertheless, reports on the synthesis of 1,3-amino alcohols through alkene functionalization are scarce.^22−25^ We envisioned that a catalytic asymmetric [4 + 2]-cycloaddition of olefins with *N*-Boc-formaldimine to form the corresponding oxazinanones, valuable 1,3-amino alcohol precursors and often found as key structural elements in bioactives themselves,^26−29^ could provide a valuable approach within this underexplored area. Indeed, on the basis of our continuous efforts in exploring chemical transformations of unfunctionalized hydrocarbon feedstocks,^10,30,31^ we present here a highly enantioselective, Bro̷nsted acid-catalyzed, inverse-electron-demand hetero-Diels–Alder reaction of olefins with in situ generated *N*-Boc-formaldimine (Figure 1c). Our method provides an inexpensive, scalable, and straightforward approach to valuable pharmaceuticals, such as (*R*)-fluoxetine, from unbiased olefins.

**Figure 1:**
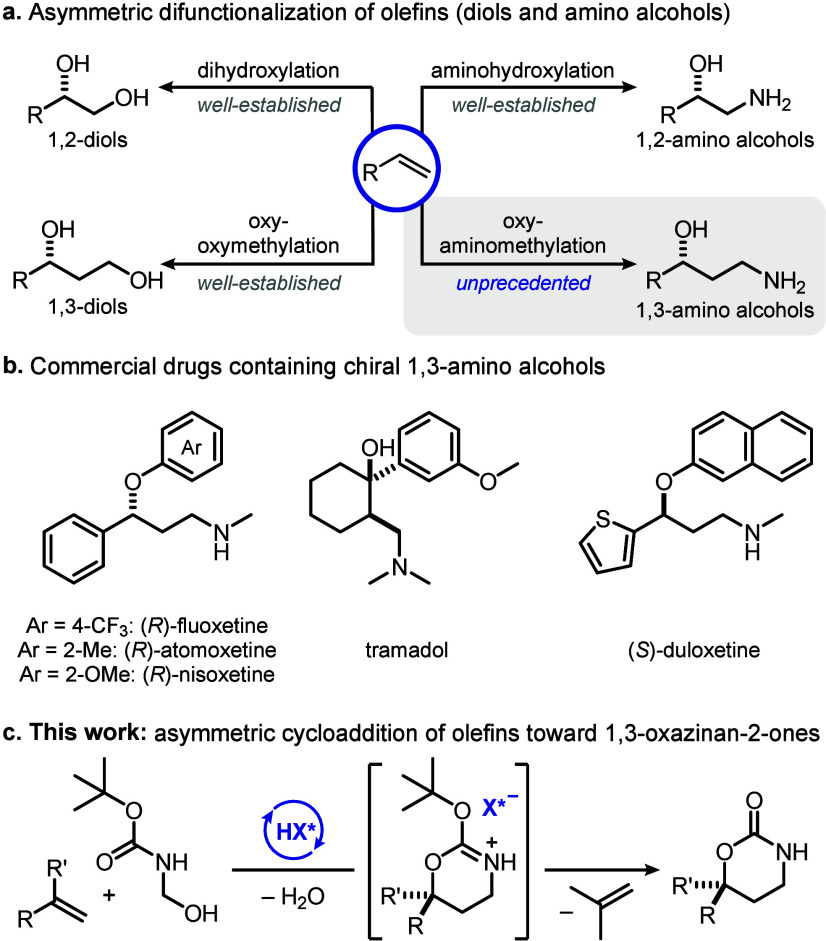
(a) Olefins as building blocks for the synthesis of 1,2- and 1,3-difunctionalized molecules. (b) Pharmacologically active molecules containing 1,3-amino alcohol units. (c) Our approach: IDPi-catalyzed cycloaddition of simple olefins.

We began our study by investigating the reaction of *tert*-butyl(hydroxymethyl)carbamate (**1a**) as *N*-Boc-formaldimine precursor with styrene (**2a**, 20 equivalents, Table 1). Moderately acidic chiral phosphoric acid catalysts (CPA, p*K*_a_ ∼ 13 in MeCN)^32^ failed to deliver the desired product **3a**. More acidic iminoimidodiphosphate (*i*IDP, p*K*_a_ ∼ 9.0 in MeCN) or disulfonimide (DSI, p*K*_a_ ∼ 4.8 in MeCN)^32,33^ catalysts led to the formation of 1,3-oxazinan-2-one **3a** in moderate yield and enantioselectivity (see the Supporting Information for details). In contrast, the even more acidic and confined imidodiphosphorimidates (IDPi, **4**–**7**, p*K*_a_ ∼ 4.5 to 2.0 in MeCN)^33^ improved the reactivity and enantiocontrol (Table 1, entries 1 and 2). Installation of the *p*-*tert*-butyl group on the 3,3′-aryl substituents of the catalyst BINOL backbone led to the formation of product **3a** in 50% yield and promising enantioselectivity (entry 3). Excellent enantiocontrol was achieved when the IDPi triflyl core was exchanged with perfluorophenylsulfonyl groups in catalyst **6b** (entry 4). Gratifyingly, further optimization, including lowering the temperature to −25 °C, the amount of styrene to 10 equivalents, and the catalyst loading to 1 mol %, provided product **3a** with 97:3 enantiomeric ratio and in 73% yield (entries 5–7 and the Supporting Information). Lowering the amount of styrene to 5 equivalents resulted in reduced yield and increased side product formation (entry 8 and the Supporting Information). Nonetheless, the catalyst loading could be reduced to 0.5 mol % on a larger-scale reaction (5 mmol of **1a**) without compromising the stereoselectivity to provide 0.46 g of **3a** (62% isolated yield, entry 9). Satisfactorily, the unreacted styrene was recovered almost quantitatively (94%) by simple distillation from the crude reaction alongside a 72% recovery of the IDPi catalyst (see the Supporting Information for details).

**Table 1:**
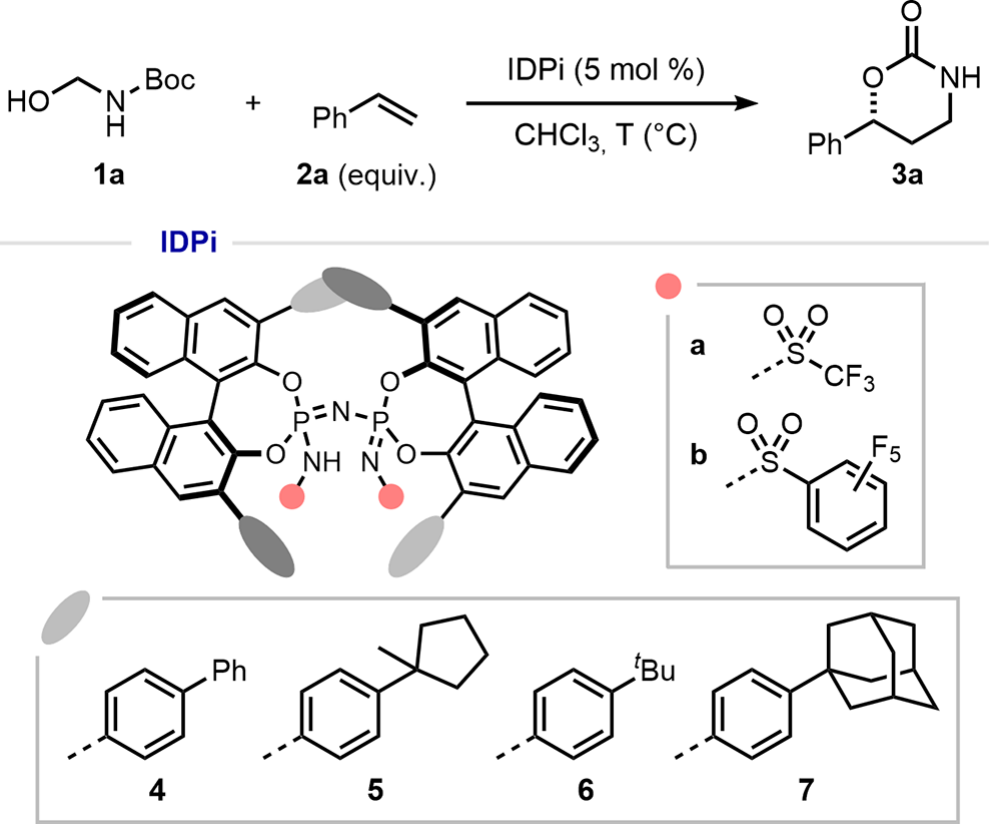
Reaction Development

entry^a^	IDPi	T (°C)	**2a** equiv.	yield (%)^b^	er
1	**4a**	rt	20	56	68:32
2	**5a**	rt	20	64	72:18
3	**6a**	rt	20	56	82:18
4	**6b**	rt	20	50	93:7
5	**6b**	–25	10	75	97:3
6^c^	**6b**	–25	10	73	97:3
7^c^	**7b**	–25	10	61	93:7
8^c^	**6b**	–25	5	51	97:3
9^d^^,^^e^	**6b**	–25	10	62	96.5:3.5

a Reactions conducted with eletrophile **1a** (0.025 mmol), olefin **2a**, and (*S,S*)-IDPi catalyst (5 mol %) in CHCl_3_ (0.3 M).

b Determined by ^1^H NMR analysis.

c Using 1 mol % catalyst loading.

d Performed on 5 mmol scale.

e Using 0.5 mol % catalyst loading.

The scope of the reaction was next explored by using the optimized conditions (Figure 2). The reaction was assessed using a diverse array of styrene derivatives with different electronic properties and substituents at different ring positions. Terminal styrenes, featuring either weakly electron-donating groups (Me, *t*-Bu, and CH_2_Cl) or electron-withdrawing groups (F, Cl, and Br) as *para*-substituents, could be transformed to the corresponding 1,3-oxazinan-2-ones **3** with moderate to good yields and excellent enantiomeric ratios (**3b**–**3d** and **3f**–**3k**). Markedly, 1,4-divinylbenzene **2c** provided the monofunctionalized product **3c** in 42% yield and 95.5:4.5 er. Styrenes bearing *meta*- or *ortho*- substituents also proved to be suitable substrates (**2e** and **2l**–**2n**). Remarkably, the methodology could also be applied to heteroaryl olefins. 3-Vinyl-thiophene (**2o**) gave product **3o** in excellent yield and with 93:7 enantiomeric ratio. In addition, benzofuran- and benzothiophene-derived olefins provided the respective cycloadducts **3p** and **3q** in moderate to good yields with high enantioselectivities. Furthermore, catalyst **6b** was also effective in reactions involving α-alkyl-substituted styrenes, which resulted in products containing a tertiary carbamate in reasonable to good yields and enantiomeric ratios of up to 99:1 (**3r**–**3t**). Having established a scope of amenable aromatic olefins, we subsequently turned our attention to purely aliphatic α,α-dialkyl olefins. The superior enantioinduction of IDPi catalyst **6b** was demonstrated by affording products **3v** and **3w** in moderate to good yields and exceptional enantioselectivities.

**Figure 2:**
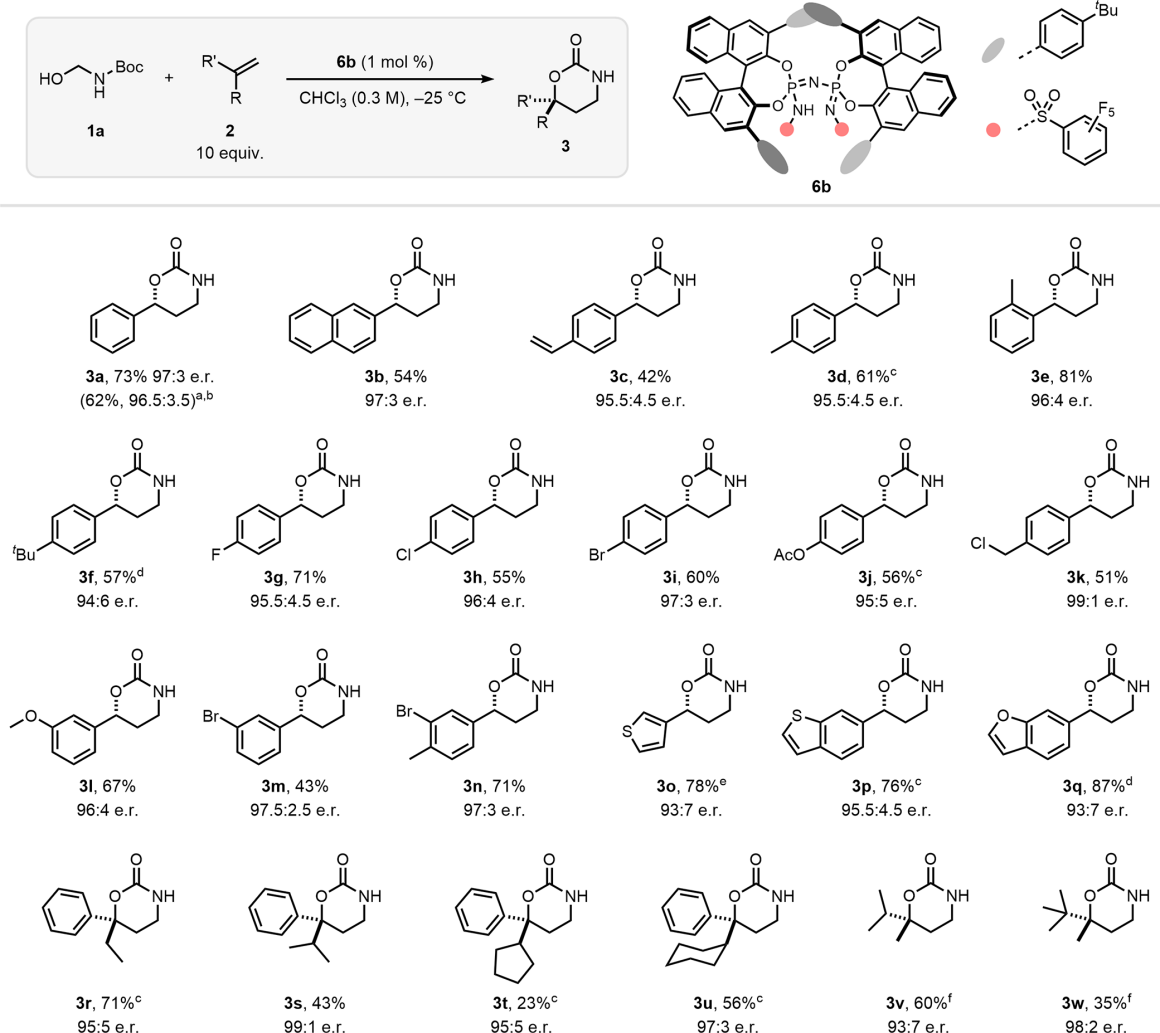
Substrate scope. Isolated yields were obtained after chromatographic purification. ^a^Performed on a 5 mmol scale. ^b^Using 0.5 mol % catalyst loading. ^c^Reaction in Et_2_O/CHCl_3_ (3:1 *v*/*v*) at −30 °C. ^d^Reaction in Et_2_O/CHCl_3_ (3:1 *v*/*v*) at −40 °C. ^e^Reaction in MTBE at −25 °C. ^f^Reaction in Et_2_O/CHCl_3_ (3:1 *v*/*v*) at −10 °C. See the Supporting Information for detailed reaction conditions.

To showcase the synthetic utility of the obtained cycloaddition products, we envisioned accessing the antidepressant (*R*)-fluoxetine hydrochloride in a concise synthesis on a multigram-scale (Figure 3). Using 2.50 g of electrophile **1a** and styrene (**2a**) provided 2.20 g of product **3a** after chromatographic purification. As shown in Table 1, the catalyst and unreacted styrene can be easily recovered. Treatment of **3a** with LiAlH_4_ resulted in the formation of (*R*)-3-(methylamino)-1-phenylpropan-1-ol (**8**), a common intermediate utilized in the synthesis of antidepressants (*R*)-atomoxetine, (*R*)-nisoxetine, and (*R*)-fluoxetine. After simple extractive workup, the base-mediated reaction of amino alcohol **8** with *p*-chlorobenzotrifluoride, followed by acidification, yielded 3.05 g (96.5:3.5 er) of (*R*)-fluoxetine hydrochloride salt (**9**) in 60% overall yield and only one chromatographic purification needed.

**Figure 3:**
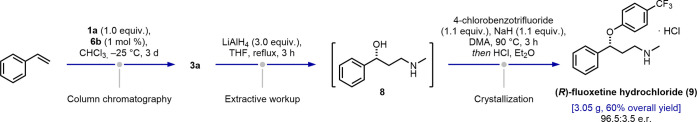
Synthesis of (*R*)-fluoxetine hydrochloride from styrene. See the Supporting Information for detailed reaction conditions.

With the aim of elucidating the reaction mechanism, a series of isotope labeling and spectroscopic studies were performed. The stereospecific nature of the IDPi-catalyzed reaction was first confirmed by utilizing β-deuterium-labeled styrenes (***cis*-2a-β-d**_**1**_ and ***trans*-2a-β-d**_**1**_) as substrates. If a stepwise mechanism were occurring, *cis/trans* scrambling would manifest at the benzylic position of products **3a-d**_**1**_. However, examination of the ^1^H NMR spectra of the reaction mixture revealed that the stereochemistry of the initial olefin was preserved in the resulting cycloadducts **3a-d**_**1**_. The observed outcomes indicate that the reaction catalyzed by IDPi **6b** is likely to follow a concerted, possibly asynchronous [4 + 2]-type cycloaddition pathway (Figure 4a).^10,31^

**Figure 4:**
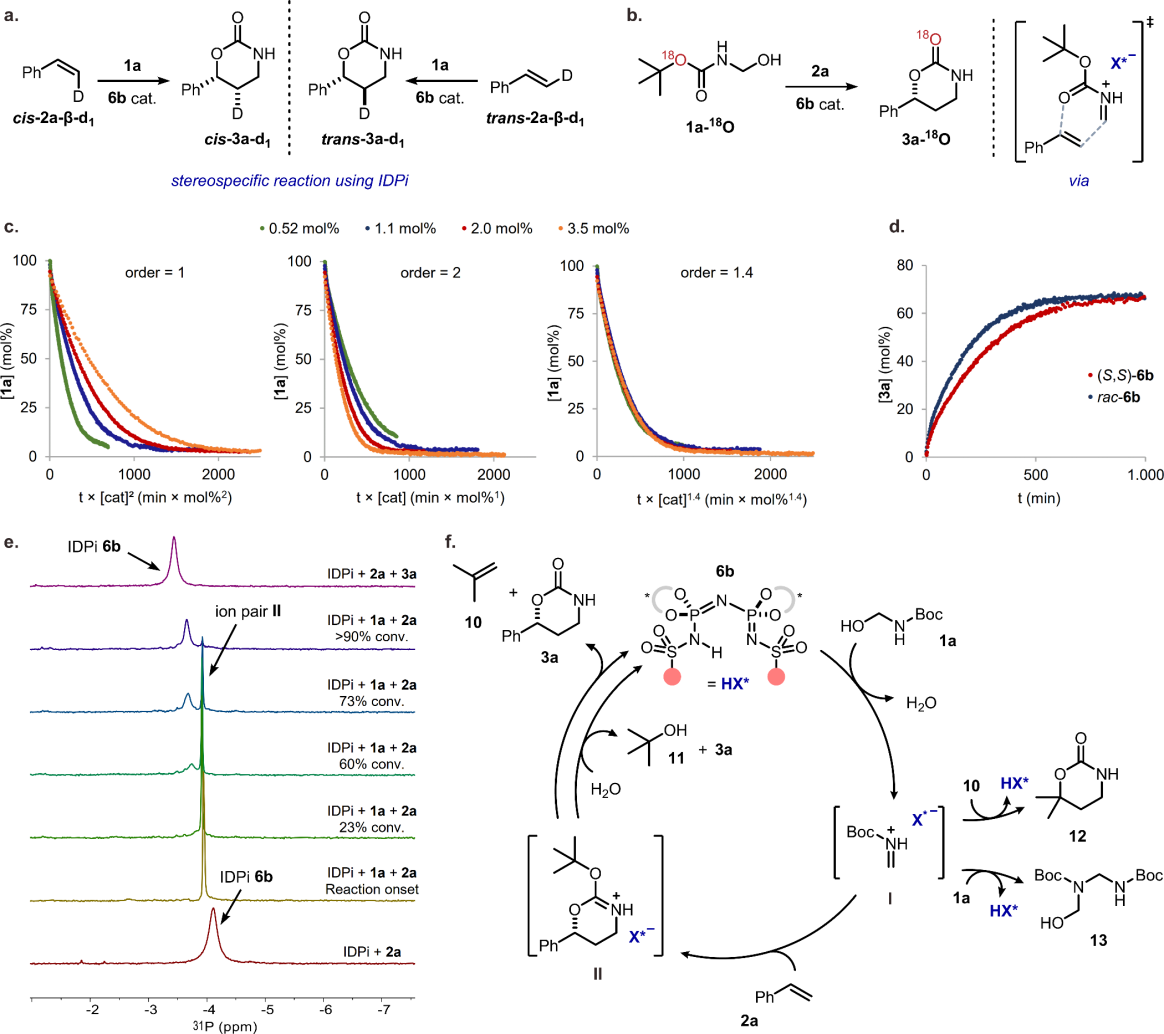
(a) Stereospecificity experiments with β-d-styrenes. (b) Experiments with ^18^O-labeled substrate. (c) Catalyst order determination by VTNA analysis.^35^ (d) Comparison of product formation when using racemic or enantiopure IDPi. (e) ^31^P NMR catalyst signals during the course of the reaction. (f) Proposed catalytic cycle.

To estimate the geometry of the transition state in the cycloaddition, selective ^18^O-labeling of the alkoxy oxygen in substrate **1a** was undertaken, which enabled differentiation between the carbonyl oxygen and the alkoxy oxygen without affecting the substrate’s inherent reactivity. Subsequently, **1a-**^**18**^**O** was subjected to the optimized reaction conditions using catalyst **6b**. Analysis of the ^13^C NMR spectrum indicated complete incorporation of the labeled oxygen into the carbonyl C=^18^O position of product **3a-**^**18**^**O**, suggesting that the nucleophilic attack of the olefin onto iminium ion **I** occurs exclusively from the carbonyl oxygen atom (Figure 4b and the Supporting Information for details).

Monitoring the reaction by ^1^H NMR revealed the expected formation of isobutene (**10**) together with several side products, including *tert*-butanol (**11**), isobutene cycloadduct **12**, and dimeric electrophile **13**. The observation of compounds **12** and **13** suggests the occurrence of competing off-pathway side reactions, which could explain the need for an excess of olefin **2** to achieve high yields in the desired transformation. It is plausible that the rapid conversion of a short-lived and highly electrophilic iminium ion **I** leads to the formation of the aforementioned side products (see the Supporting Information for further details). Remarkably, advanced NMR studies unveiled the formation of a long-lived reaction intermediate **II** whose sharp ^31^P NMR signal is distinguishable from the broad one of the free catalyst (Figure 4e and the Supporting Information). This observation suggests isobutene release from ionic intermediate **II** to product **3a**, which plausibly occurs after the stereochemical information is established, to be the turnover-limiting step in the reaction.^34^

Keen to understand the role of the catalyst, the reaction order of IDPi **6b** was investigated using variable time normalization analysis (VTNA) of different catalyst concentrations.^35^ Surprisingly, the best overlay between the reaction profiles was obtained when using a catalyst order of 1.4 (Figure 4c). Building on previous findings of a catalyst order greater than one, we hypothesized that the involvement of multiple catalyst molecules in the reaction enantiodetermining step could lead to nonlinear effects (NLE). The enantioenrichment of product **3a** was found to exhibit a linear relationship with that of catalyst **6b** used in the reaction, thereby ruling out the presence of NLE (see the Supporting Information). This result is consistent with only a single catalyst molecule being involved in the enantiodetermining step of the reaction. However, this does not exclude the possibility that multiple catalyst molecules might be involved in subsequent steps. This finding led us to hypothesize that, because of the high stability of intermediate **II**, a second catalyst molecule might assist in the isobutene release from **II** to furnish product **3a**. To further investigate this hypothesis, we compared the reaction rate with racemic catalyst *rac*-**6b** to that of the standard enantioselective reaction and observed a rate enhancement by a factor of 1.4 (Figure 4d and the Supporting Information). This observation suggests that the presence of heterochiral catalyst mixtures can lead to a faster decay of intermediate **II**, thereby accelerating the overall reaction, which supports the hypothesis that multiple catalyst molecules are involved in the consumption of **II**.

Another plausible explanation for the found catalyst order is that a water molecule would attack the electrophilic *tert*-butyl group of intermediate **II** in a parallel reaction pathway (see discussion in the Supporting Information) to form *tert*-butanol and **3a**. In recent years, Burés has discussed the scenario in which a byproduct from one step in the cycle serves as a reactant in a subsequent step.^36^ This situation can lead to catalyst orders greater than one despite only one catalyst molecule being involved in the rate-limiting step. Intrigued by the role of water in our system, we studied the contribution of additional water in the reaction. Our findings indicate that additional water decreased the reaction rate and reduced the formation of both isobutene and *tert*-butanol, thus suggesting overall catalyst inhibition (see the Supporting Information). However, it cannot be excluded that water generated from the activation of substrate **1a**, likely confined within the catalyst pocket, could react with intermediate **II** during the turnover-limiting step to produce **3a** and *tert*-butanol and result in an apparent order in catalyst greater than one.

Based on these findings, the following reaction mechanism is proposed (Figure 4f). The catalytic cycle is initiated by protonation of substrate **1a** and subsequent water elimination to yield iminium ion **I**. Because of the high electrophilicity of **I**, competing side reactions can occur at this point, as observed with the formation of byproducts **12** and **13**. The enantiodetermining step between olefin **2a** and **I** would take place in a concerted fashion, which leads to the second reaction intermediate **II**. We propose two plausible pathways to close the cycle: the successive elimination of isobutene from **II**, which can be aided by a second catalyst molecule to yield product **3a**, or the water-promoted elimination of *tert*-butanol from **II** to furnish the desired product. Further investigations about the intriguing mechanism are currently ongoing in our laboratory.

In summary, we developed a novel approach toward the synthesis of enantioenriched oxazinanones via the cycloaddition of electrophile **1a** and nonbiased alkenes. High levels of enantioselectivity across a broad range of substrates were achieved with privileged IDPi **6b**, showcasing the versatility of this method for synthesizing valuable 1,3-amino alcohols. Mechanistic investigations are consistent with a [4 + 2]-cycloaddition proceeding via a concerted pathway and with the involvement of intermediate **II**. Thorough analysis suggests the possibility of a second catalyst molecule contributing to restoring the catalytic cycle. The elucidation of this unusual intermediate not only enriches our understanding of the IDPi-catalyzed reactions but also sheds light on potential avenues for reaction kinetics.

## Supporting Information Available

Experimental details and analytical data for all new compounds and NMR spectra (PDF)

## Abbreviations

AcO: acetate
Ar: aryl
Boc: *tert*-butoxycarbonyl
Bu: butyl
cat.: catalyst
CPA: chiral phosphoric acid
conv.: conversion
d: days
DMA: dimethylacetamide
DSI: disulfonimide
equiv.: equivalents
er: enantiomeric ratio
Et: ethyl
IDPi: imidodiphosphorimidate
*i*IDP: iminoimidodiphosphate
h: hours
min: minutes
Me: methyl
MeCN: acetonitrile
MTBE: methyl *tert*-butyl ether
NLE: nonlinear effects
*rac*: racemic
NMR: nuclear magnetic resonance
*p*: *para*
Ph: phenyl
rt: room temperature
*t*: tertiary
T: temperature
THF: tetrahydrofuran
*v*: volume
VTNA: variable time normalization analysis
