# Supplementary material for: Catalytic Asymmetric Cycloaddition of Olefins with In Situ Generated N-Boc-Formaldimine
Source: J Am Chem Soc. 2024 Nov 18;146(47):32292–7. doi: 10.1021/jacs.4c13538 (PMC11613496; doi:10.1021/jacs.4c13538)
Supplement: Supplementary file 1 — ja4c13538_si_001.pdf [file ja4c13538_si_001.pdf]

Supporting Information for

Catalytic Asymmetric Cycloaddition of Olefins with *In Situ* Generated *N*-Boc-Formaldimine

Marian Guillén, Markus Leutzsch, and Benjamin List*

Max-Planck-Institut für Kohlenforschung, Kaiser-Wilhelm-Platz 1, 45470 Mülheim an der Ruhr, Germany

*E-mail: list@kofo.mpg.de

1. Materials and Methods 2
2. Substrate Synthesis 4
   2.1. Synthesis of Electrophiles 4
   2.2. Synthesis of ^{18}O-Labelled Substrate Analogous 6
   2.3. Synthesis of Olefins 8
3. Reaction Development 12
4. Asymmetric Oxy-Aminomethylation of Olefins 17
5. Absolute Configuration Determination 26
6. Scale-Up Experiments 27
   6.1. Catalyst and Olefin Recovery Experiment 27
   6.2. Formal Synthesis of (*R*)-Fluoxetine Hydrochloride from Styrene 2a 27
7. Synthesis of (*S*,*S*)-IDPi Catalysts 30
   7.1. Synthesis of substituted (*S*)-BINOLs 30
   7.2. Synthesis of (*S*,*S*)-IDPis 31
8. Mechanistic Investigations 34
   8.1. On the Stereospecificity: Reactions with D-Labeled Substrates 34
   8.2. ^{18}O-Labeling Reaction: Isotope Shift Effect 35
   8.3. NMR Kinetic Studies 36
9. Copies of NMR Spectra 53
10. Copies of HPLC and GC Traces 98
11. References 122

1. Materials and Methods

Unless otherwise stated, all reactions were magnetically stirred and conducted in oven-dried (90 °C) or flame-dried glassware in anhydrous solvents under argon, applying standard Schlenk techniques. Solvents and liquid reagents, as well as solutions of solid or liquid reagents were added via syringes, stainless steel or polyethylene cannulas through rubber septa or through a weak argon counter-flow. Solid reagents were added through a weak argon counter-flow. Reactions at lower temperatures (T < rt) were cooled to the specified temperature using appropriate cooling baths or cryostats, respectively. Cooling baths were prepared in Dewar vessels, filled with ice/water (0 °C), cooled acetone (< −78 °C) or dry ice/acetone (−78 °C). Alternatively, the reaction vessel was placed in an aluminum block inside a cryostat set to the desired temperature. Heated oil baths were used for reactions requiring elevated temperatures. Solvents were removed under reduced pressure at 40 °C using a rotary evaporator, and unless otherwise stated, the remaining compound was dried in high vacuum (10^{-3} mbar) at rt. All given yields are isolated yields of chromatographically and NMR-spectroscopically pure materials, unless otherwise stated.

Chemicals were purchased from commercial suppliers (including abcr, Acros Organics, Alfa Aesar, Fluorochem, Sigma-Aldrich, and TCI) and used without further purification unless otherwise stated.

Solvents (CyH, DCM, Et_2O, THF, toluene) were dried by distillation from an appropriate drying agent in the technical department of the Max-Planck-Institut für Kohlenforschung and received in Schlenk flasks under argon.[1] Other anhydrous solvents were purchased from commercial suppliers and used as received.

Reactions were monitored by thin layer chromatography (TLC) on silica gel pre-coated plastic sheets (0.2 mm, Macherey-Nagel). Visualization was accomplished by irradiation with UV light (254 nm and 366 nm) and/or phosphomolybdic acid (PMA) stain and/or Cerium Ammonium Molybdate (CAM) stain and/or permanganate stain.

Column chromatography was carried out using Merck silica gel (60 Å, 230−400 mesh, particle size 0.040−0.063 mm) using technical grade solvents. Elution was accelerated using compressed air. Automated column chromatography was conducted on a Biotage® Isolera™ ISO-4SW instrument, using SNAP Ultra HP-Sphere™ 25 µm chromatography cartridges. All fractions containing a desired substance were combined and concentrated in vacuo, then redissolved in an appropriate solvent and filtered through cotton to remove silica residues.

^{1}H, ^{13}C, ^{19}F, ^{31}P nuclear magnetic resonance (NMR) spectra were recorded on a Bruker AVIII-500 MHz or Bruker NEO 600 MHz (equipped with a BBO CryoProbe) spectrometer in a suitable deuterated solvent. The solvent employed and respective measuring frequency are indicated for each experiment. Chemical shifts are reported in ppm (δ) relative to tetramethylsilane (TMS) with the residual solvent resonance serving as the internal reference (δ 7.26 ppm for $CDCl_3$; δ 5.32 ppm for CD_2Cl_2). The resonance multiplicity is described as s (singlet), d (doublet), t (triplet), q (quadruplet), p (pentet), hept (heptet), m (multiplet), and b (broad). All spectra were recorded at 298 K unless otherwise noted, processed with the program MestReNova 15.0.0, and coupling constants are reported as observed. Data are provided as follows: chemical shift in ppm, resonance multiplicity, coupling constant *J* in Hz, and number of protons. All spectra are broadband decoupled unless otherwise noted.

Electron impact (EI) mass spectrometry (MS) was performed on a Finnigan MAT 8200 (70 eV) or MAT 8400 (70 eV) spectrometer. Electrospray ionization (ESI) mass spectrometry was conducted on a Bruker ESQ 3000 spectrometer. High resolution mass spectrometry (HRMS) was performed on a Finnigan MAT 95 (EI) or Bruker APEX III FTMS (7T magnet, ESI). The ionization method and mode of detection employed is indicated for the respective experiment and all masses are reported in atomic units per elementary charge (*m/z*) with an intensity normalized to the most intense peak.

Specific rotations $[a]_T^D$ were measured with a Rudolph RA Autopol IV Automatic Polarimeter at the indicated temperature (T) with a sodium lamp (sodium D line, λ = 589 nm). Measurements were performed in an acid resistant 1 mL cell (50 mm length) with concentrations (g/100 mL) reported in the corresponding solvent.

High-performance liquid chromatography (HPLC) was performed on Shimadzu LC-20AD liquid chromatograph (SIL-20AC auto sampler, CMB-20A communication bus module, DGU-20A5 degasser, CTO-20AC column oven, SPD-M20A diode array detector), Shimadzu LC-20AB liquid chromatograph (SIL-20ACHT auto sampler, DGU-20A5 degasser, CTO-20AC column oven, SPD-M20A diode array detector), or Shimadzu LC-20AB liquid chromatograph (reversed phase, SIL-20ACHT auto sampler, CTO-20AC column oven, SPD-M20A diode array detector) using Daicel columns with chiral stationary phases. All solvents used were HPLC-grade solvents, purchased from Merck. The column employed and respective solvent mixture are indicated for each experiment.

Liquid Chromatography-Mass Spectrometry Liquid chromatography-mass spectrometry (LC-MS) was performed on Shimadzu LC-MS 2020 liquid chromatograph. All solvents used were HPLC-grade solvents purchased from Sigma-Aldrich. The column employed, the respective solvent mixture, and the MS parameters are indicated for each experiment.

Gas chromatography (GC) analyses on a chiral stationary phase were performed on HP 6890 and 5890 series instruments (split-mode capillary injection system, flame ionization detector (FID), hydrogen carrier gas). All of these analyses were conducted in the GC department of the Max-Planck-Institut für Kohlenforschung. The conditions employed are described in detail for the individual experiments.

2. Substrate Synthesis

Unless otherwise stated, chemicals were purchased from commercial suppliers (abcr, Acros, Alfa Aesar, Fluorochem, Sigma-Aldrich, and TCI) and used without further purification. Other substrates were prepared by methods indicated below.

2.1. Synthesis of Electrophiles

tert-Butyl (hydroxymethyl)carbamate (1a)

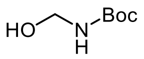

Following a reported procedure:[2] to a round-bottomed flask was added *tert*-butyl carbamate (4.6 g, 34.1 mmol, 1 equiv.), Na_2CO_3 (1.8 g, 17.1 mmol, 0.5 equiv.), paraformaldehyde (1.4 g, 47.8 mmol, 1.4 equiv.) and water (50 mL). The mixture was vigorously stirred and heated to 60 °C for 30 min, until a clear solution was obtained. Then, the mixture was further stirred at rt overnight. The mixture was then diluted with water (20 mL), poured into a separatory funnel and extracted with EtOAc (3x20 mL). The combined organic phases were washed with water (1x20 mL) and brine (1x20 mL), then dried over anhydrous Na_2SO_4, filtered and concentrated. A viscous oily residue was obtained, which was purified by silica gel column chromatography using *n*-hexane/EtOAc (2:1) as eluent to afford 2.6 g (52% yield) of compound **1a** as a white solid. Spectroscopic data was consistent with the values reported in the literature.[2]

^{1}H NMR (501 MHz, $CDCl_3$) δ 5.52 (s, 1H), 4.66 (t, *J* = 6.6 Hz, 2H), 3.01 (s, 1H), 1.47 (s, 9H).

^{13}C NMR (126 MHz, $CDCl_3$) δ 20.2, 65.4, 80.1, 156.1.

Isopropyl (hydroxymethyl)carbamate (1b)

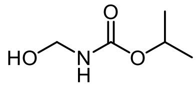

Following a reported procedure:[2] to a round-bottomed flask was added isopropyl carbamate (500 mg, 4.8 mmol, 1 equiv.), Na_2CO_3 (260 mg, 2.4 mmol, 0.5 equiv.), paraformaldehyde (200 mg, 6.8 mmol, 1.4 equiv.) and water (7 mL). The mixture was vigorously stirred and heated to 60 °C for 30 min, until a clear solution was obtained. Then, the mixture was further stirred at rt overnight. The mixture was then diluted with water (5 mL), poured into a separatory funnel and extracted with EtOAc (3x5 mL). The combined organic phases were washed with water (1x5 mL) and brine (1x5 mL), then dried over anhydrous Na_2SO_4, filtered and concentrated. A viscous oily residue was obtained, which was purified by silica gel column chromatography using *n*-hexane/EtOAc (2:1) as eluent to afford 285 mg (44% yield) of compound **1b** as a white solid.

^{1}H NMR (501 MHz, $CDCl_3$) δ 5.62 (s, 1H), 4.94 (hept, *J* = 6.3 Hz, 1H), 4.70 (t, *J* = 7.1 Hz, 2H), 3.22 (d, *J* = 7.4 Hz, 1H), 1.24 (d, *J* = 6.3 Hz, 6H).

^{13}C NMR (126 MHz, $CDCl_3$) δ 156.6, 68.9, 66.3, 22.2.

HRMS (ESI) calculated for $C_5H_{11}NO_2Na$ ([M+Na$^+$]): 156.06311, found: 156.06407.

Ethyl (hydroxymethyl)carbamate (1c)

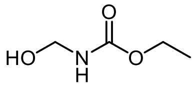

Following a reported procedure:[2] to a round-bottomed flask was added urethane (432 mg, 4.8 mmol, 1 equiv.), Na_2CO_3 (257 mg, 2.4 mmol, 0.5 equiv.), paraformaldehyde (204 mg, 6.8 mmol, 1.4 equiv.) and water (7.3 mL). The mixture was vigorously stirred and heated to 60 °C for 30 min, until a clear solution was obtained. Then, the mixture was further stirred at rt overnight. The mixture was then diluted with water (5 mL), poured into a separatory funnel and extracted with EtOAc (3x5 mL). The combined organic phases were washed with

water (1x5 mL) and brine (1x5 mL), then dried over anhydrous Na_2SO_4, filtered and concentrated. A viscous oily residue was obtained, which was purified by silica gel column chromatography using *n*-hexane/EtOAc (2:1) as eluent to afford 176 mg (30% yield) of compound **1c** as a white solid. Spectroscopic data was consistent with the values reported in the literature.[3]

^{1}H NMR (501 MHz, $CDCl_3$) δ 5.74 (s, 1H), 4.71 (t, J = 5.7 Hz, 2H), 4.15 (q, J = 7.0 Hz, 2H), 3.45 (s, 1H), 1.25 (t, J = 7.1 Hz, 3H).

^{13}C NMR (126 MHz, CDCl3) δ 157.0, 66.3, 61.4, 14.6.
HRMS (ESI) calculated for $C_4H_9NO_2Na$ ([M+Na$^+$]): 142.04746, found: 142.04725.

((*tert*-Butoxycarbonyl)amino)methyl acetate (1d)

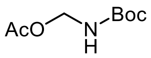

Following a reported procedure:[4] *tert*-butyl carbamate (586 mg, 5.0 mmol, 1 equiv.) and paraformaldehyde (165 mg, 5.5 mmol, 1.1 equiv.) were suspended in a mixture of acetic acid (4.5 mL) and acetic anhydride (13.5 mL) in a round-bottomed flask. The reaction was vigorously stirred and heated at 60 °C for 24 h. After this time, the solvent was removed under vacuum at 70 °C to give a yellow oily residue, which was purified by silica gel column chromatography using *n*-hexane/EtOAc (8:2) as eluent to afford 685 mg (72% yield) of product **1d** as a viscous colorless liquid. Spectroscopic data was consistent with the values reported in the literature.[4]

^{1}H NMR (501 MHz, $CDCl_3$) δ 5.70 (s, 1H), 5.14 (d, J = 7.6 Hz, 2H), 2.06 (s, 3H), 1.45 (s, 9H).

^{13}C NMR (126 MHz, $CDCl_3$) 171.8, 155.0, 80.6, 66.6, 28.2, 21.0.

(((Benzyloxy)carbonyl)amino)methyl acetate (1e)

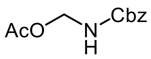

Following a reported procedure:[4] benzyl carbamate (756 mg, 5.0 mmol, 1 equiv.) and paraformaldehyde (165 mg, 5.5 mmol, 1.1 equiv.) were suspended in a mixture of acetic acid (4.5 mL) and acetic anhydride (13.5 mL) in a round-bottomed flask. The reaction was vigorously stirred and heated at 60 °C for 24 h. After this time, the solvent was removed under vacuum at 70 °C to give a yellow oily residue, which was purified by silica gel column chromatography using *n*-hexane/EtOAc (8:2) as eluent to afford 669 mg (60% yield) of product **1e** as a viscous colorless liquid. Spectroscopic data was consistent with the values reported in the literature.[4]

^{1}H NMR (501 MHz, $CDCl_3$) δ 7.40 – 7.26 (m, 5H), 6.40 (t, J = 7.5 Hz, 1H), 5.25 – 5.17 (m, 2H), 5.13 (s, 2H), 2.02 (s, 3H).

^{13}C NMR (126 MHz, $CDCl_3$) 171.3, 155.8, 135.7, 128.3, 128.0, 127.9, 66.9, 66.5, 20.6.

((((9*H*-Fluoren-9-yl)methoxy)carbonyl)amino)methyl acetate (1f)

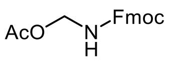

Following a reported procedure:[4] (9*H*-fluoren-9-yl)methyl carbamate (1.20 g, 5.0 mmol, 1 equiv.) and paraformaldehyde (165 mg, 5.5 mmol, 1.1 equiv.) were suspended in a mixture of acetic acid (4.5 mL) and acetic anhydride (13.5 mL) in a round-bottomed flask. The reaction was vigorously stirred and heated at 60 °C for 24 h. After this time, the solvent was removed under vacuum at 70 °C to give a yellow oily residue, which was purified by silica gel column chromatography using *n*-hexane/EtOAc (8:2) as eluent to afford 713 mg (46% yield) of product **1f** as a white solid. Spectroscopic data was consistent with the values reported in the literature.[4]

^{1}H NMR (501 MHz, $CDCl_3$) δ 7.77 (d, *J* = 7.5 Hz, 2H), 7.64 – 7.55 (m, 2H), 7.41 (t, *J* = 7.5 Hz, 2H), 7.32 (td, *J* = 7.4, 1.2 Hz, 2H), 6.03 (t, *J* = 7.7 Hz, 1H), 5.22 (d, *J* = 7.6 Hz, 2H), 4.45 (d, *J* = 7.0 Hz, 2H), 4.22 (t, *J* = 7.0 Hz, 1H), 2.08 (s, 3H).

^{13}C NMR (126 MHz, $CDCl_3$) 143.8, 141.5, 127.9, 127.2, 125.1, 120.2, 67.4, 66.7, 47.2, 21.1.

tert-Butyl (((_tert_-butoxycarbonyl)amino)methyl)(hydroxymethyl)carbamate (13)

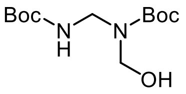

A flame-dried Schlenk under argon atmosphere was charged with 265 mg of **1a** (1.8 mmol) and 2.2 mL of $CHCl_3$. The mixture was cooled down to –20 ºC for 15 min. After this time, a stock solution of catalyst **6b** in 0.1 mL of $CHCl_3$ (1 mol%) was added to the previous flask via syringe in one portion. After checking full conversion (TLC monitoring) the reaction was quenched with one equivalent of triethylamine. The mixture was warmed up to rt, suspended on Celite, and further purified by silica gel column chromatography (*n*-hexane/EtOAc mixtures from 8:2 to 6:4) to afford 59 mg (24% yield) of product **13**.

2 rotamers are observed in the NMR data at 233 K (–40 °C).

Major rotamer:

^{1}H NMR (600 MHz, 233K, $CDCl_3$) δ 5.86 (t, *J* = 6.6 Hz, 1H), 4.89 (t, *J* = 7.6 Hz, 1H), 4.80 (d, *J* = 7.5 Hz, 2H), 4.50 (d, *J* = 6.6 Hz, 2H), 1.47 (s, 9H), 1.43 (s, 9H).

^{13}C NMR (151 MHz, 233 K, $CDCl_3$) δ 158.0, 155.1, 81.4, 72.7, 55.2, 28.3, 28.2.

Minor rotamer:

^{1}H NMR (600 MHz, 233K, $CDCl_3$) δ 5.46 (t, *J* = 6.7 Hz, 1H), 4.83 (d, *J* = 7.7 Hz, 2H), 4.60 (t, *J* = 7.7 Hz, 1H), 4.52 (d, *J* = 6.7 Hz, 2H), 1.48 (s, 9H), 1.44 (s, 9H).

^{13}C NMR (151 MHz, 233 K $CDCl_3$) δ 157.5, 154.6, 81.6, 81.4, 72.5, 55.1, 28.4, 28.3.

HRMS (ESI) calculated for $C_{12}H_{24}N_2O_5Na$ ($[M+Na^+]$): 299.157742, found: 299.15788.

2.2. Synthesis of ^{18}O-Labelled Substrate Analogous

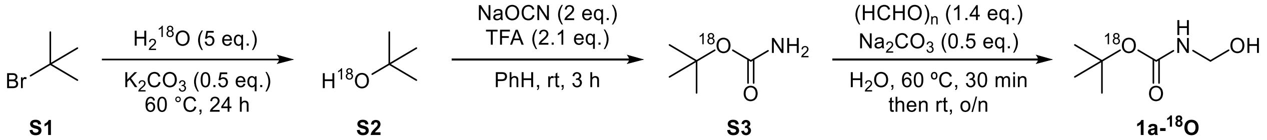

2-Methylpropan-2-ol-^{18}O (S2)

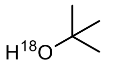

Following a modified procedure:[5] a flame-dried Schlenk under argon atmosphere was charged with 1.37 g of *tert*-butyl bromide (10.0 mmol) and 1.0 mL of $H_2{}^{18}O$ (97%, 5 equiv.) 690 mg (0.5 equiv.) of Na_2CO_3 were added slowly and the mixture was vigorously stirred at 70 °C overnight. After cooling to rt, and analysis of the reaction mixture with ^{1}H NMR spectroscopy revealed that the reaction had gone to >95% conversion. The mixture was diluted with DCM and anhydrous Na_2SO_4 was added. The resulting mixture was filtered and carefully evaporated at 25 °C to obtain 2-methylpropan-2-ol-^{18}O **S2** as a colorless oil. Spectroscopic data was consistent with the values reported in the literature.[5]

^{1}H NMR (501 MHz, $CDCl_3$) δ 1.29 (s, 9H).

^{13}C NMR (126 MHz, $CDCl_3$) δ 69.3, 31.4.

HRMS (ESI) calculated for $C_4H_{10}{}^{18}ONa$ ([M+Na$^+$]): 99.06663, found: 99.06653.

tert-Butyl carbamate-^{18}O (S3)

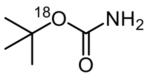

Following a modified procedure:[6] to a round-bottomed flask was added 2-methylpropan-2-ol-^{18}O **S2** (0.72 g, 9.5 mmol, 1 equiv.), sodium cyanate (1.24 g, 19.0 mmol, 2 equiv.), and 8.0 mL of benzene. Trifluoroacetic acid (1.5 mL, 20.0 mmol, 2.1 equiv.) was added dropwise under stirring at rt. The flask was closed loosely with a glass stopper and the mixture was stirred at rt overnight. Water (10 mL) was added and both phases were separated. The aqueous phase was extracted with EtOAc (3x5 mL). The combined organic phases were washed with water (1x10 mL) and brine (1x10 mL), dried over anhydrous Na_2SO_4 and evaporated. The obtained solid was recrystallized from *n*-hexane to obtain 336 mg (30% yield, 82.8% ^{18}O-incorporation) of compound **S3** as colorless crystal needles. Spectroscopic data was consistent with the values reported in the literature.[6]

^{1}H NMR (501 MHz, $CDCl_3$) δ 4.70 (s, 1H), 1.43 (s, 4H).

^{13}C NMR (126 MHz, $CDCl_3$) δ 156.7, 79.7, 28.4.

HRMS (GC-EI) calculated for $C_5H_{12}NO^{18}O$ ([M$^+$]): 120.090498, found: 120.090338.

tert-Butyl (hydroxymethyl)carbamate-^{18}O (1a-^{18}O)

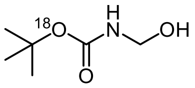

Following a reported procedure:[2] to a round-bottomed flask was added *tert*-butyl carbamate-^{18}O **S3** (290 mg, 2.4 mmol, 1 equiv.), Na_2CO_3 (130 mg, 1.2 mmol, 0.5 equiv.), paraformaldehyde (102 mg, 3.4 mmol, 1.4 equiv.) and water (3.7 mL). The flask was closed with a septum and the mixture was vigorously stirred and heated to 60 °C for 30 min, until a clear solution was obtained. Then, the mixture was further stirred at rt overnight. The mixture was then diluted with water (2 mL), poured into a separatory funnel and extracted with EtOAc (3x2 mL). The combined organic phases were washed with water (1x2 mL) and brine (1x2 mL), then dried over anhydrous Na_2SO_4, filtered and concentrated. A viscous oily residue was obtained, which was purified by silica gel column chromatography using *n*-hexane/EtOAc (2:1) as eluent to afford 78 mg (22% yield, 81.7% ^{18}O-incorporation) of product **1a-^{18}O** as a white solid. Spectroscopic data was consistent with the values reported in the literature.[2]

NMR at 253 K (–20 °C) shows two rotamers (ratio approx. 10:1).

Major rotamer:

^{1}H NMR (600 MHz, $CDCl_3$) δ 5.65 (t, J = 6.9 Hz, 1H), 4.67 (t, J = 7.3 Hz, 2H), 3.60 (t, J = 7.6 Hz, 1H), 1.44 (s, 9H).

^{13}C NMR (151 MHz, 253 K, $CDCl_3$) δ 156.125 (^{16}O-C2), 156.114 (^{18}O-C2), 80.516 (^{16}O-C3), 80.469 (^{18}O-C3), 65.855 (C1), 28.339 (C4).

HRMS (ESI) calculated for $C_6H_{13}NO_2{}^{18}ONa$ ([M+Na$^+$]): 172.08301, found: 172.08225.

2.3. Synthesis of Olefins

General Procedure A: Wittig Olefination

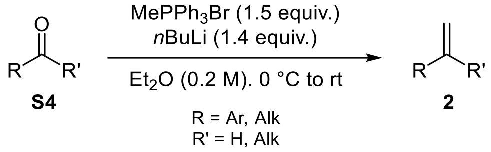

In a flame-dried double neck round-bottomed flask, methyltriphenylphosphonium bromide (2.14 g, 6.0 mmol, 1.5 equiv.) was suspended in Et_2O (15 mL) and cooled to 0 °C. *n*-BuLi was added dropwise and the mixture was stirred at 0 °C for 1 h before a solution of aldehyde or ketone **S4** (4.0 mmol, 1.0 equiv.) in Et_2O (5 mL) was added dropwise. The mixture was further stirred at rt overnight. After checking full conversion (TLC monitoring), the mixture was diluted with Et_2O (10 mL) and water (20 mL), and the aqueous layer was extracted with Et_2O (3x20 mL). The combined organic layers were washed with brine (1x20 mL), dried over anhydrous Na_2SO_4, filtered and concentrated under reduced pressure. The crude was suspended in pentane and filtered to remove triphenylphosphine oxide. Purification by flash column chromatography on silica gel (*n*-pentane/Et_2O mixtures) afforded the corresponding olefin **2**.

General Procedure B: Suzuki–Miyaura Cross-Coupling

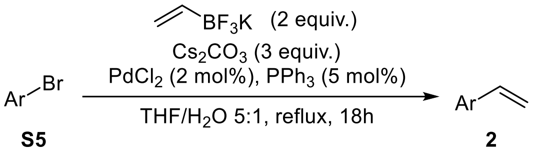

A flame-dried double neck round-bottomed flask equipped with a reflux condenser was charged with aryl bromide **S5** (3.5 mmol, 1 equiv.) and THF (12.5 mL). To this was added potassium vinyltrifluoroborate (7 mmol, 2 equiv.), $PdCl_2$ (0.07 mmol, 2 mol%), PPh_3 (0.21 mmol, 6 mol%), Cs_2CO_3 (10.5 mmol, 3 equiv.), and water (2.8 mL). The mixture was stirred at rt and degassed by bubbling argon for 10 min, then heated to reflux under argon. After allowing to cool to rt and checking full conversion (TLC monitoring), the mixture was filtered through a short pad of Celite, washing with MTBE. The filtrate was washed with water (1 x 30 mL) and brine (1 x 30 mL), then dried over anhydrous Na_2SO_4, filtered and concentrated under reduced pressure. Purification by flash column chromatography on silica gel (*n*-pentane/DCM mixtures) afforded the corresponding olefin **2**.

1,4-Divinylbenzene (2c)

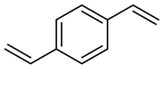

Following *General Procedure A*, employing terephthaladehyde (1.61 g, 12.0 mmol) as starting material, 2.05 equiv. of methyltriphenylphosphonium bromide (8.79 g, 24.6 mmol), and 2.2 equiv. of potassium *tert*-butoxide (2.96 g, 26.4 mmol) as base. The crude product was purified by silica gel column chromatography using *n*-pentane as eluent to give **2c** as a white solid (1.07 g, 68%). Spectroscopic data was consistent with the values reported in the literature.[7]

^{1}H NMR (501 MHz, $CDCl_3$) δ 7.40 (s, 4H), 6.73 (dd, J = 17.6, 10.9 Hz, 2H), 5.78 (dd, J = 17.6, 0.9 Hz, 2H), 5.27 (dd, J = 10.9, 0.9 Hz, 2H).

^{13}C NMR (126 MHz, $CDCl_3$) δ 137.1, 136.5, 126.4, 113.8.

2-Bromo-1-methyl-4-vinylbenzene (2n)

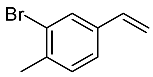

Following *General Procedure A*, employing 3-bromo-4-methylbenzaldehyde (497 mg, 2.5 mmol) as starting material: The crude product was purified by silica gel column chromatography using *n*-pentane/Et2O 9.5:0.5 as eluent to give **2n** as a colorless liquid (396 mg, 80%). Spectroscopic data was consistent with the values reported in the literature.[8]

^{1}H NMR (501 MHz, $CDCl_3$) δ 7.62 (d, *J* = 1.8 Hz, 1H), 7.30 – 7.24 (m, 1H), 7.20 (d, *J* = 7.8 Hz, 1H), 6.65 (ddd, *J* = 17.6, 10.9, 1.3 Hz, 1H), 5.74 (dd, *J* = 17.5, 1.5 Hz, 1H), 5.27 (dd, *J* = 10.9, 1.6 Hz, 1H), 2.42 (s, 3H).

^{13}C NMR (126 MHz, $CDCl_3$) δ 137.4, 137.3, 135.5, 130.9, 130.1, 125.3, 125.2, 114.5, 22.8.

6-Vinylbenzo[*b*]thiophene (2p)

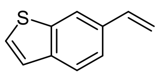

Following *General Procedure B,* employing 6-bromobenzo[*b*]thiophene (448 mg, 2.1 mmol) as starting material. The crude product was purified by silica gel column chromatography using *n*-pentane/DCM 95:5 as eluent to give **2p** as a white solid (269 mg, 80%).

^{1}H NMR (501 MHz, $CDCl_3$) δ 7.88 (s, 1H), 7.77 (d, *J* = 8.3 Hz, 1H), 7.48 (dd, *J* = 8.2, 1.6 Hz, 1H), 7.43 (d, *J* = 5.2 Hz, 1H), 7.31 (dd, *J* = 5.4, 0.9 Hz, 1H), 6.84 (dd, *J* = 17.6, 10.9 Hz, 1H), 5.83 (dd, *J* = 17.5, 0.9 Hz, 1H), 5.30 (dd, *J* = 10.8, 0.8 Hz, 1H).

^{13}C NMR (126 MHz, $CDCl_3$) δ 140.4, 139.4, 136.9, 134.2, 126.9, 123.8, 123.6, 122.5, 120.6, 113.9.

6-Vinylbenzofuran (2q)

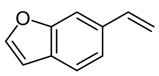

Following *General Procedure B,* employing 6-bromobenzofuran (690 mg, 3.5 mmol) as starting material. The crude product was purified by silica gel column chromatography using *n*-pentane/DCM 95:5 as eluent to give **2q** as a colorless liquid (362 mg, 72%).

^{1}H NMR (501 MHz, $CDCl_3$) δ 7.62 (d, *J* = 2.1 Hz, 1H), 7.54 (d, *J* = 7.9 Hz, 2H), 7.34 (dd, *J* = 7.9, 1.5 Hz, 1H), 6.82 (dd, *J* = 17.5, 10.9 Hz, 1H), 6.75 (dd, *J* = 2.2, 1.0 Hz, 1H), 5.79 (d, *J* = 17.5 Hz, 1H), 5.26 (dd, *J* = 10.9, 0.8 Hz, 1H).

^{13}C NMR (126 MHz, $CDCl_3$) δ 155.6, 145.7, 137.1, 134.6, 127.3, 121.5, 121.1, 113.5, 109.1, 106.7.

But-1-en-2-ylbenzene (2r)

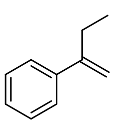

Following *General Procedure A,* employing propiophenone (1.61 g, 12.0 mmol) as starting material. The crude product was purified by silica gel column chromatography using *n*-pentane as eluent to give **2r** as a colorless liquid (1.15 g, 73%). Spectroscopic data was consistent with the values reported in the literature.[9]

^{1}H NMR (501 MHz, CD_2Cl_2) δ 7.45 – 7.39 (m, 2H), 7.36 – 7.29 (m, 2H), 7.28 – 7.23 (m, 1H), 5.30 – 5.25 (m, 1H), 5.07 (d, *J* = 1.5 Hz, 1H), 2.56 – 2.48 (m, 2H), 1.10 (t, *J* = 7.4 Hz, 3H).

^{13}C NMR (126 MHz, CD_2Cl_2) δ 150.6, 141.9, 128.6, 127.7, 126.4, 111.1, 28.4, 13.2.

(3-Methylbut-1-en-2-yl)benzene (2s)

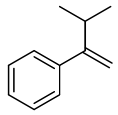

Following *General Procedure A*, employing isobutyrophenone (1.78 g, 12.0 mmol) as starting material. The crude product was purified by silica gel column chromatography using *n*-pentane as eluent to give **2s** as a colorless liquid (1.33 g, 76%). Spectroscopic data was consistent with the values reported in the literature.[10]

^{1}H NMR (501 MHz, $CDCl_3$) δ 7.45 – 7.35 (m, 4H), 7.35 – 7.29 (m, 1H), 5.22 (t, *J* = 1.7 Hz, 1H), 5.11 (p, *J* = 1.2 Hz, 1H), 2.96 – 2.85 (m, 1H), 1.18 (dt, *J* = 6.8, 1.3 Hz, 6H).

^{13}C NMR (126 MHz, $CDCl_3$) δ 155.9, 142.9, 128.2, 127.1, 126.7, 110.0, 32.4, 22.1.

(1-Cyclopentylvinyl)benzene (2t)

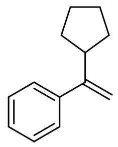

Following *General Procedure A*, employing cyclopentyl(phenyl)methanone (697 mg, 4.0 mmol) as starting material. The crude product was purified by silica gel column chromatography using *n*-pentane as eluent to give **2t** as a colorless liquid (525 mg, 76%). Spectroscopic data was consistent with the values reported in the literature.[11]

^{1}H NMR (501 MHz, $CDCl_3$) δ 7.36 (dt, *J* = 8.1, 1.8 Hz, 2H), 7.29 (ddt, *J* = 8.0, 6.3, 1.5 Hz, 2H), 7.26 – 7.21 (m, 1H), 5.16 (d, *J* = 0.9 Hz, 1H), 5.06 (q, *J* = 1.2 Hz, 1H), 2.94 (ddt, *J* = 13.3, 8.2, 4.2 Hz, 1H), 1.93 – 1.81 (m, 2H), 1.77 – 1.52 (m, 4H), 1.52 – 1.36 (m, 2H).

^{13}C NMR (126 MHz, $CDCl_3$) δ 153.1, 143.4, 128.2, 127.1, 126.7, 110.2, 44.7, 32.3, 25.0

(1-Cyclohexylvinyl)benzene (2u)

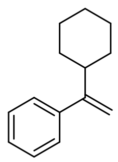

Following *General Procedure A*, employing cyclohexyl(phenyl)methanone (1.73 g, 9.2 mmol) as starting material. The crude product was purified by silica gel column chromatography using *n*-pentane as eluent to give **2u** as a colorless liquid (1.62 g, 95%). Spectroscopic data was consistent with the values reported in the literature.[12]

^{1}H NMR (501 MHz, $CDCl_3$) δ 7.44 – 7.35 (m, 4H), 7.35 – 7.30 (m, 1H), 5.21 (d, *J* = 1.4 Hz, 1H), 5.08 (t, *J* = 1.4 Hz, 1H), 2.50 (tdd, *J* = 11.6, 4.0, 2.0 Hz, 1H), 1.97 – 1.72 (m, 5H), 1.45 – 1.33 (m, 2H), 1.32 – 1.19 (m, 3H).

^{13}C NMR (126 MHz, $CDCl_3$) δ 155.1, 143.0, 128.1, 126.9, 126.6, 110.3, 42.6, 32.7, 26.8, 26.4.

***cis*-Styrene-(β)-d$_1$ (*cis*-2a-β-d$_1$)**

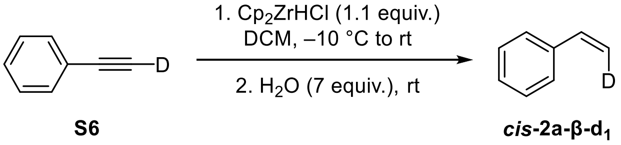

Following a reported procedure:[8] A Schlenk flask under argon atmosphere was charged with phenylacetylene-d$_1$ (245 mg, 2.4 mmol)[13] and dry DCM (7.5 mL). The flask was covered with aluminum foil and the mixture was cooled to 0 °C. Schwartz's Reagent (680 mg, 2.6 mmol, 1.1 equiv.) was then added in two equal portions in rapid succession (over 2 min). The mixture was allowed to stir at 0 °C for 15 min, then the cold bath was removed and the stirring was continued at rt in the dark for 2 h. The flask was cooled to 0 °C, and the mixture was quenched with water (0.35 mL, 19.2 mmol, 8 equiv.) and stirred vigorously at rt for 3 h. The mixture was diluted with DCM (3 mL), followed by the addition of anhydrous

Na_2SO_4 and filtration. The filtrate was concentrated under reduced pressure (400 mbar, water bath of rotavap at 25 °C) until 1 mL remained. *n*-Pentane (2 mL) was added and the mixture was filtered over a Celite pad to remove the white precipitate; the filter cake was rinsed with *n*-pentane and the filtrate was again concentrated under reduced pressure (400 mbar, 25 °C). Purification by silica gel column chromatography using *n*-pentane as eluent afforded the corresponding olefin ***cis*-2a-β-d₁** a colorless liquid (75 mg, 30% yield, approx. 99% D-incorporation). Spectroscopic data was consistent with the values reported in the literature.[8]

^{1}H NMR (501 MHz, $CDCl_3$) δ 7.45 – 7.40 (m, 2H), 7.37 – 7.31 (m, 2H), 7.26 (tt, *J* = 7.3, 1.3 Hz, 1H), 6.72 (dt, *J* = 10.9, 2.6 Hz, 1H), 5.23 (d, *J* = 10.9 Hz, 1H).

***trans*-Styrene-(β)-*d* (*trans*-2a-β-d$_1$)**

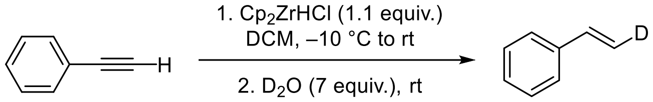

S7 ***trans*-2a-β-d$_1$**

Following a reported procedure:[8] A Schlenk flask under argon atmosphere was charged with phenylacetylene (306 mg, 3.0 mmol) and dry DCM (7.5 mL). The flask was covered with aluminum foil and the mixture was cooled to 0 °C. Schwartz's Reagent (850 mg, 3.3 mmol, 1.1 equiv.) was then added in two equal portions in rapid succession (over 2 min). The mixture was allowed to stir at 0 °C for 15 min, then the cold bath was removed and the stirring was continued at rt in the dark for 2 h. The flask was cooled to 0 °C, and the mixture was quenched with D_2O (0.45 mL, 99.9% D, 24.9 mmol, 8.3 equiv.) and stirred vigorously at rt for 3 h. The mixture was diluted with DCM (3 mL), followed by the addition of anhydrous Na_2SO_4 and filtration. The filtrate was concentrated under reduced pressure (400 mbar, water bath of rotavap at 25 °C) until 1 mL remained. *n*-Pentane (2 mL) was added and the mixture was filtered over a Celite pad to remove the white precipitate; the filter cake was rinsed with *n*-pentane and the filtrate was again concentrated under reduced pressure (400 mbar, 25 °C). Purification by silica gel column chromatography using *n*-pentane as eluent afforded the corresponding olefin ***trans*-2a-β-d$_1$** as a colorless liquid (95 mg, 30% yield, approx. 91% D-incorporation). Spectroscopic data was consistent with the values reported in the literature.[8]

^{1}H NMR (501 MHz, $CDCl_3$) δ 7.45 – 7.40 (m, 2H), 7.37 – 7.31 (m, 2H), 7.26 (tt, *J* = 7.3, 1.3 Hz, 1H), 6.72 (dt, *J* = 17.5, 1.5 Hz, 1H), 5.75 (d, *J* = 17.7 Hz, 1H).

3. Reaction Development

Reaction conditions were optimized as described below using styrene (**2a**) as representative substrate.

An oven-dried screwcap vial (1.5 mL) equipped with a magnetic stir bar was charged with carbamate **1** (0.025 mmol), styrene (**2a**) (10 equiv.), solvent (90 µL), placed under argon, and if convenient cooled down to the desired reaction temperature. The catalyst was dissolved separately in 30 µL of solvent and was added to the reaction vial in one portion via syringe. The vial was then sealed and the reaction was stirred for 2 days. The reaction was quenched with one drop of triethylamine and analyzed by ^{1}H NMR spectroscopy using 1,3,5-trimethoxybenzene as internal standard for yield determination. After evaporation of the solvent, the crude mixture was purified by preparative TLC (silica gel, DCM/MeOH 3%, partially stained with PMA) and analyzed via HPLC or GC for the determination of the enantiomeric ratio.

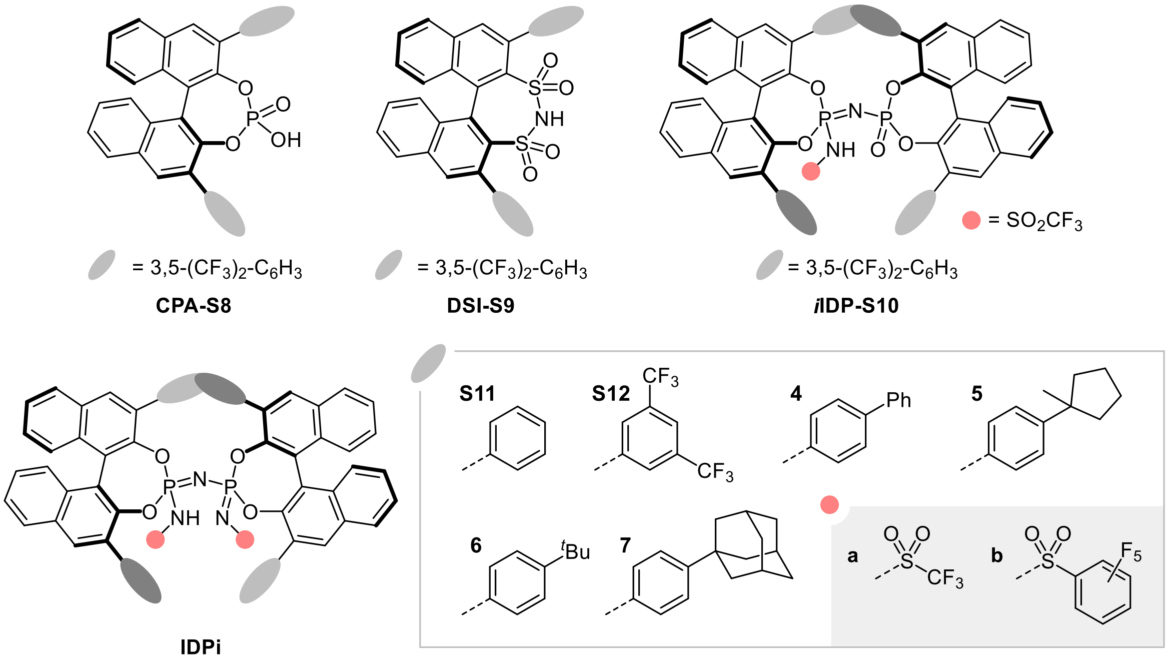

Figure SI-1. Catalysts used in the reaction optimization.

Table SI-1. Reaction optimization for styrene (**2a**) as representative substrate for the cycloaddition reaction.

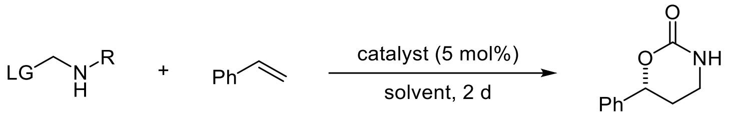

1 **2a** **3a**

Entry	LG	R	2a equiv.	Catalyst	Solvent (conc.)	Temp (°C)	Yield	e.r.
**1**	OH	Boc	20	**CPA-S8** (5 mol%)	$CHCl_3$ (0.2 M)	rt	<5%	-
**2**	OH	Boc	20	**DSI-S9** (5 mol%)	$CHCl_3$ (0.2 M)	rt	30%	65:35
**3**	OH	Boc	20	***i*IDP-S10** (5 mol%)	$CHCl_3$ (0.2 M)	rt	46%	75:25
**4**	OH	Boc	20	**S11a** (5 mol%)	$CHCl_3$ (0.2 M)	rt	77%	68:32
**5**	OH	Boc	20	**S12a** (5 mol%)	$CHCl_3$ (0.2 M)	rt	63%	70:30
**6**	OH	Boc	20	**S12b** (5 mol%)	$CHCl_3$ (0.2 M)	rt	66%	55:45
**7**	OH	Boc	20	**4b** (5 mol%)	$CHCl_3$ (0.2 M)	rt	64%	72:28
**8**	OH	Boc	20	**5a** (5 mol%)	$CHCl_3$ (0.2 M)	rt	56%	68:32
**9**	OH	Boc	20	**6a** (5 mol%)	$CHCl_3$ (0.2 M)	rt	20%	58:42
**10**	OH	Boc	20	**6b** (5 mol%)	$CHCl_3$ (0.2 M)	rt	50%	93:7
**11**	AcO	Boc	20	**6b** (5 mol%)	$CHCl_3$ (0.2 M)	rt	35%	93:7
**12**	AcO	Cbz	20	**6b** (5 mol%)	$CHCl_3$ (0.2 M)	rt	-	-
**13**	AcO	Fmoc	20	**6b** (5 mol%)	$CHCl_3$ (0.2 M)	rt	-	-
**14**	OH	CO_2Et	20	**6b** (5 mol%)	$CHCl_3$ (0.2 M)	rt	-	-
**15**	OH	$CO_2{}^iPr$	20	**6b** (5 mol%)	$CHCl_3$ (0.2 M)	rt	-	-
**16**[a]	OH	Boc	1	**6b** (5 mol%)	$CHCl_3$ (0.2 M)	rt	28%	92:8
**17**[a]	OH	Boc	1	**6b** (5 mol%)	$CHCl_3$ (1 M)	rt	36%	92:8
**18**	OH	Boc	20	**6b** (5 mol%)	CyH (0.2 M)	rt	57%	91:9
**19**	OH	Boc	20	**6b** (5 mol%)	PhMe (0.2 M)	rt	46%	93:7
**20**	OH	Boc	20	**6b** (5 mol%)	Et_2O (0.2 M)	rt	38%	91:9
**21**	OH	Boc	20	**6b** (5 mol%)	DCM (0.2 M)	rt	42%	92:8
**22**	OH	Boc	20	**6b** (5 mol%)	THF (0.2 M)	rt	53%	93:7
**23**	OH	Boc	20	**6b** (5 mol%)	$CHCl_3$ (0.3 M)	10	62%	93:7
**24**	OH	Boc	20	**6b** (5 mol%)	$CHCl_3$ (0.3 M)	0	28%	92.5:7.5
**25**	OH	Boc	20	**6b** (5 mol%)	$CHCl_3$ (0.3 M)	–25	72%	97:3
**26**	OH	Boc	20	**6b** (5 mol%)	$CHCl_3$ (0.3 M)	–40	58%	97:3
**27**	OH	Boc	20	**6b** (5 mol%)	DCM (0.3 M)	–60	13%	97:3
**28**	OH	Boc		**6b** (5 mol%)	neat (0.1 M)	–25	79%	97:3
**29**	OH	Boc	10	**6b** (5 mol%)	$CHCl_3$ (0.3 M)	–25	75%	97:3
**30**	OH	Boc	5	**6b** (5 mol%)	$CHCl_3$ (0.3 M)	–25	51%	97:3
**31**	OH	Boc	2	**6b** (5 mol%)	$CHCl_3$ (0.3 M)	–25	42%	96.5:3.5
**32**	OH	Boc	10	**6b** (5 mol%)	$CHCl_3$ (1 M)	–25	52%	97:3
**33**	OH	Boc	10	**6b** (5 mol%)	$CHCl_3$ (0.1 M)	–25	67%	96.5:3.5
**34**	OH	Boc	10	**6b** (2 mol%)	$CHCl_3$ (0.3 M)	–25	75%	97:3
**35**	OH	Boc	10	6b (1 mol%)	$CHCl_3$ (0.3 M)	–25	73%	97:3

**36**	OH	Boc	10	**6b** (0.5 mol%)	$CHCl_3$ (0.3 M)	–25	63%	97:3
**37**	OH	Boc	10	**7b** (1 mol%)	$CHCl_3$ (0.3 M)	–25	61%	93:7
**38**[b]	OH	Boc	10	6b (1 mol%)	$CHCl_3$ (0.3 M)	–25	63%	96.3:3.7
**39**[c]	OH	Boc	10	6b (1 mol%)	$CHCl_3$ (0.3 M)	–25	56%	96.5:3.3

[a]Using 5 equivalents of substrate **1**. [b]Using 4Å molecular sieves (200mg/mmol **1**). [c]Using 5Å molecular sieves (200mg/mmol **1**).

Table SI-2. Substrates reoptimization.

3	2 equiv.	catalyst	Solvent (conc.)	Temp (°C)	Yield	e.r.
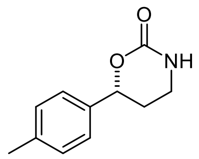	10	6b	$CHCl_3$ (0.3 M)	–25	62%	93.5:6.5
	10	5b	THF (0.3 M)	–25	37%	94.5:5.5
	10	6b	DCM (0.3 M)	–25	59%	94:6
	10	6b	$Et_2O/CHCl_3$ (3:1, 0.3 M)	–30	63%	96:4
	10	6b	$CHCl_3$ (0.3 M)	–40	51%	93:7
	10	6b	$Et_2O/CHCl_3$ (3:1, 0.3 M)	–40	50%	96:4
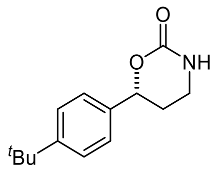	10	6b	$CHCl_3$ (0.5 M)	–25	60%	89:11
	10	6b	THF (0.3 M)	–25	38%	91:9
	10	6b	$Et_2O/CHCl_3$ (3:1, 0.3 M)	–40	52%	94:6
	10	6b	$CHCl_3$ (0.3 M)	–40	62%	93:7
	10	6b	$Et_2O/CHCl_3$ (3:1, 0.3 M)	–50	9%	94:6
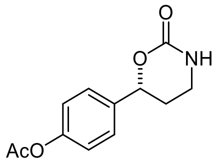	10	6b	$CHCl_3$ (0.3 M)	–25	46%	94:6
	10	6b	$CHCl_3$ (0.3 M)	–30	43%	94:6
	10	6b	$Et_2O/CHCl_3$ (3:1, 0.3 M)	–30	57%	95:5
	10	6b	$CHCl_3$ (0.3 M)	–40	38%	94:6
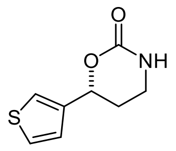	10	6b	$CHCl_3$ (0.3 M)	–25	55%	91:9
	10	6b	$Et_2O/CHCl_3$ (3:1, 0.3 M)	–25	69%	91:9
	10	6b	$Et_2O/CHCl_3$ (3:1, 0.3 M)	–40	75%	91:9
	10	6b	$Et_2O/CHCl_3$ (3:1, 0.3 M)	–50	69%	90.5:9.5
	10	6b	MTBE (0.3 M)	–25	73%	92.2:7.8
	10	6b	MTBE (0.3 M)	–40	46%	92:8
	10	6b	PhMe (0.3 M)	–25	71%	91.5:8.5
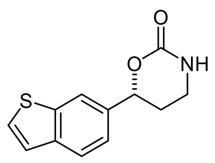	10	6b	$CHCl_3$ (0.3 M)	–25	62%	93:7
	10	6b	$Et_2O/CHCl_3$ (3:1, 0.3 M)	–30	68%	95:5

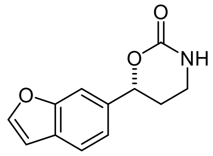	10	6b	$CHCl_3$ (0.3 M)	–25	74%	90.5:9:5
	10	6b	$Et_2O/CHCl_3$ (3:1, 0.3 M)	–30	87%	91:9
	10	6b	$Et_2O/CHCl_3$ (3:1, 0.3 M)	–40	88%	93:7
	10	6b	MTBE (0.3 M)	–40	32%	93:7
	10	6b	$Et_2O/CHCl_3$ (3:1, 0.3 M)	–50	53%	93:7
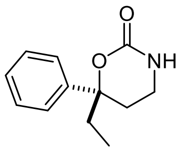	10	6b	$CHCl_3$ (0.3 M)	–25	52%	7.5:92:5
	10	6b	$THF/CHCl_3$ (3:1, 0.3 M)	–30	37%	6:94
	10	6b	$Et_2O/CHCl_3$ (3:1, 0.3 M)	–30	42%	5:95
	10	6b	$CHCl_3$ (0.3 M)	–40	48%	7.5:92:5
	10	6b	$Et_2O/CHCl_3$ (3:1, 0.3 M)	–40	36%	6:94
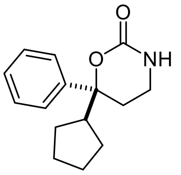	10	6b	$CHCl_3$ (0.3 M)	–25	28%	10:90
	10	6b	$CHCl_3$ (0.3 M)	–30	27%	9:91
	10	6b	$Et_2O/CHCl_3$ (3:1, 0.3 M)	–30	24%	5:95
	10	6b	$CHCl_3$ (0.3 M)	–40	28%	9:91
	10	6b	$Et_2O/CHCl_3$ (3:1, 0.3 M)	–40	10%	6:94
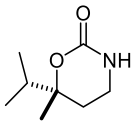	10	6b	$CHCl_3$ (0.3 M)	rt	23%	91:9
	10	6b	$CHCl_3$ (0.3 M)	–25	42%	90:10
	10	6b	$Et_2O/CHCl_3$ (3:1, 0.3 M)	rt	46%	92.2:7.8
	10	6b	$Et_2O/CHCl_3$ (3:1, 0.3 M)	–10	78%	93.2:6.8
	10	6b	MTBE (0.3 M)	–10	61%	92.5:7.5
	10	6b	$THF/CHCl_3$ (3:1, 0.3 M)	40	37%	91:9
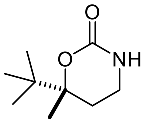	10	6b	$Et_2O/CHCl_3$ (3:1, 0.3 M)	rt	20%	95:5
	10	6b	$Et_2O/CHCl_3$ (3:1, 0.3 M)	0	36%	97.5:2.5
	10	6b	$Et_2O/CHCl_3$ (3:1, 0.3 M)	–10	37%	98:2
	10	6b	$Et_2O/CHCl_3$ (3:1, 0.3 M)	–20	26%	98:2
	10	6b	$Et_2O/CHCl_3$ (3:1, 0.3 M)	–30	18%	98:2

Table SI-2. Current scope limitations.

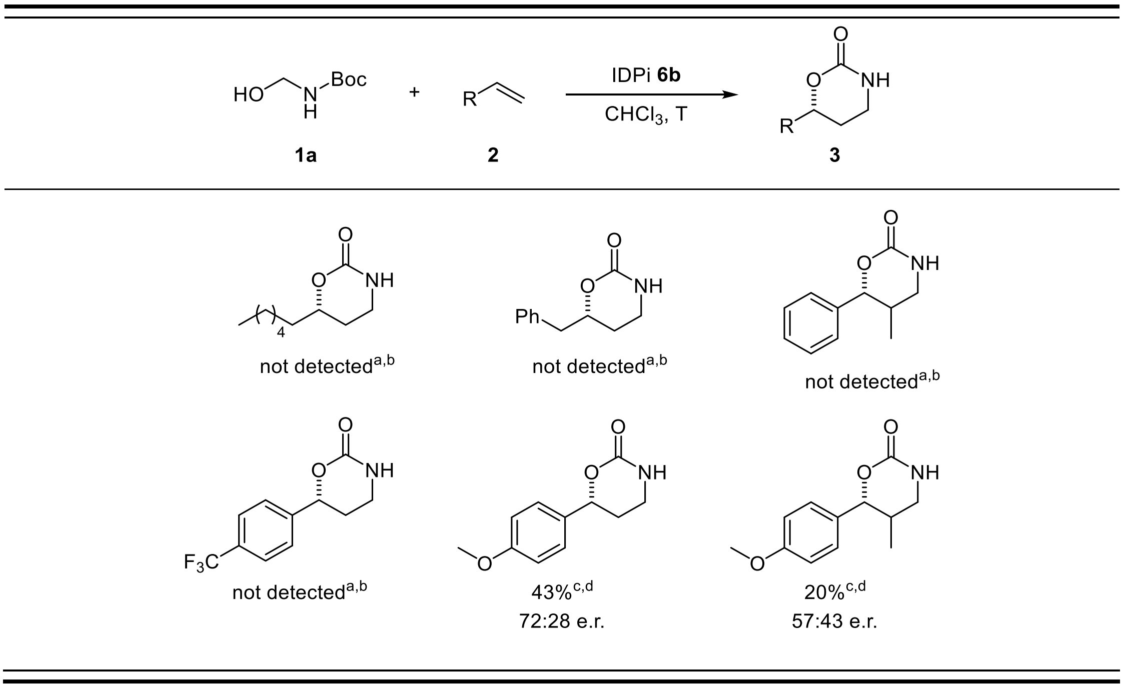

[a]Using 5 mol% of IDPi **6b**. [b]Reaction conducted at rt. [c]Using 1 mol% of IDPi **6b**. [d]Reaction conducted at –25 ºC.

4. Asymmetric Oxy-Aminomethylation of Olefins

General Procedure C: IDPi-catalyzed Oxy-Aminomethylation of Olefins

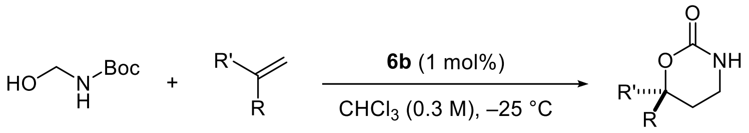

1a **2** **3**

An oven-dried screwcap vial (10 mL) equipped with a magnetic stir bar was charged with carbamate **1a** (0.5 mmol), olefin **2** (5 mmol, 10 equiv.) and dry $CHCl_3$ (1.5 mL). The tube was sealed and cooled down to the respective temperature for 30 min. After this time, a stock solution of catalyst **6b** in 0.1 mL of $CHCl_3$ (1 mol%) was added to the previous vial via syringe in one portion. After checking full conversion (TLC monitoring) the reaction was quenched with one equivalent of triethylamine. The mixture was warmed up to rt, suspended on Celite, and further purified by silica gel column chromatography (DCM/MeOH mixtures from 0.5 to 3%).

(*R*)-6-Phenyl-1,3-oxazinan-2-one (3a)

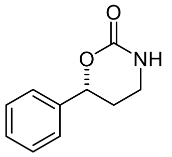

Following *General Procedure C,* employing **1a** (74 mg, 0.5 mmol, 1 equiv.) and olefin **2a** (0.57 mL, 5.0 mmol) as starting materials. After chromatographic purification, compound **3a** was obtained as a white solid (65 mg, 73%). Spectroscopic data was consistent with the values reported in the literature.[14]

^{1}H NMR (501 MHz, $CDCl_3$) δ 7.43 – 7.30 (m, 5H), 5.96 (s, 1H), 5.35 (dd, *J* = 9.9, 2.8 Hz, 1H), 3.48 (td, *J* = 11.0, 4.9 Hz, 1H), 3.39 (ddt, *J* = 12.0, 5.7, 3.6 Hz, 1H), 2.23 (dq, *J* = 14.0, 3.8 Hz, 1H), 2.09 (dtd, *J* = 14.0, 10.1, 5.5 Hz, 1H).

^{13}C NMR (126 MHz, $CDCl_3$) δ 154.5, 139.1, 128.8, 128.5, 125.8, 78.7, 39.2, 28.9.

HRMS (ESI) calculated for $C_{10}H_{11}NO_2Na^+$ ([M+Na$^+$]): 200.068198, found: 200.068117.

$[\boldsymbol{a}]_{\boldsymbol{T}}^{\mathbf{20}}$ = +50.44 (*c* = 0.23, $CHCl_3$).

HPLC (Chiralpak IC-3, *n*-heptane/*i*-PrOH 50:50, 298 K, 215 nm): t_R (major) = 14.1 min, t_R (minor) = 20.6 min, e.r. = 97:3 (94% e.e.).

Absolute configuration of **3a** was determined by comparison of the optical rotation with available literature data.[15] The absolute configuration of products **3b-3w** was assigned by analogy.

(*R*)-6-(naphthalen-2-yl)-1,3-oxazinan-2-one (3b)

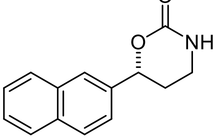

Following *General Procedure C,* employing **1a** (74 mg, 0.5 mmol, 1 equiv.) and olefin **2b** (770 mg, 5.0 mmol) as starting materials. After chromatographic purification, compound **3b** was obtained as a white solid (61 mg, 54%).

^{1}H NMR (501 MHz, $CDCl_3$) δ 7.90 – 7.81 (m, 4H), 7.54 – 7.47 (m, 2H), 7.45 (dd, *J* = 8.6, 1.7 Hz, 1H), 5.82 (s, 1H), 5.52 (dd, *J* = 9.8, 2.9 Hz, 1H), 3.53 (dddd, *J* = 11.5, 10.2, 4.9, 1.2 Hz, 1H), 3.42 (ddt, *J* = 11.6, 5.6, 3.6 Hz, 1H), 2.32 (dqd, *J* = 11.7, 4.0, 2.0 Hz, 1H), 2.18 (dtd, *J* = 14.0, 10.0, 5.5 Hz, 1H).

^{13}C NMR (126 MHz, $CDCl_3$) δ 154.4, 136.4, 133.3, 133.3, 128.7, 128.3, 127.9, 126.6, 126.5, 124.8, 123.4, 78.8, 39.2, 28.9.

HRMS (ESI) calculated for $C_{14}H_{13}NO_2Na^+$ ($[M+Na^+]$): 250.083848, found: 200.083915.

$[\boldsymbol{a}]_{\boldsymbol{T}}^{\boldsymbol{20}}$ = +50.38 (*c* = 0.13, $CHCl_3$).

HPLC (Chiralpak IC-3, *n*-heptane/*i*-PrOH 50:50, 298 K, 209 nm): t_R (major) = 16.5 min, t_R (minor) = 21.0 min, e.r. = 97:3 (94% e.e.).

(*R*)-6-(4-vinylphenyl)-1,3-oxazinan-2-one (3c)

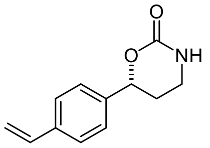

Following *General Procedure C,* employing **1a** (74 mg, 0.5 mmol, 1 equiv.) and olefin **2c** (0.65 g, 5.0 mmol) as starting materials. After chromatographic purification, compound **3c** was obtained as a white solid (43 mg, 42%).

^{1}H NMR (501 MHz, $CDCl_3$) δ 7.42 (d, *J* = 8.4 Hz, 2H), 7.33 (d, *J* = 8.2 Hz, 2H), 6.71 (dd, *J* = 17.6, 10.9 Hz, 1H), 6.57 – 6.53 (m, 1H), 5.76 (dd, *J* = 17.5, 0.9 Hz, 1H), 5.32 (dd, *J* = 9.9, 2.8 Hz, 1H), 5.27 (dd, *J* = 10.9, 0.9 Hz, 1H), 3.46 (dddd, *J* = 11.6, 10.3, 4.9, 1.1 Hz, 1H), 3.37 (ddt, *J* = 11.8, 5.6, 3.6 Hz, 1H), 2.24 – 2.15 (m, 1H), 2.05 (dtd, *J* = 14.0, 10.2, 5.6 Hz, 1H).

^{13}C NMR (126 MHz, $CDCl_3$) δ 154.81, 138.59, 137.82, 136.32, 126.55, 125.94, 114.57, 78.47, 39.01, 28.76.

HRMS (GC-EI) calculated for $C_{12}H_{13}NO_2$ ($[M^+]$): 203.094079, found: 203.093985.

$[\boldsymbol{a}]_{\boldsymbol{T}}^{\boldsymbol{20}}$ = +36.04 (*c* = 0.11, $CHCl_3$).

HPLC (Chiralpak IC-3, *n*-heptane/*i*-PrOH 50:50, 298 K, 254 nm): t_R (major) = 15.0 min, t_R (minor) = 19.6 min, e.r. = 95.4:4.6 (90.8% e.e.).

(*R*)-6-(*p*-Tolyl)-1,3-oxazinan-2-one (3d)

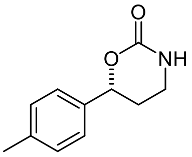

Following *General Procedure C,* employing **1a** (74 mg, 0.5 mmol, 1 equiv.) and olefin **2d** (0.66 mL, 5.0 mmol) as starting material, in $Et_2O/CHCl_3$ (3:1 *v/v*) mixture at –30 °C. After chromatographic purification, compound **3d** was obtained as a white solid (58 mg, 61%).

^{1}H NMR (501 MHz, $CDCl_3$) δ 7.31 – 7.23 (m, 2H), 7.20 (d, *J* = 7.9 Hz, 2H), 6.92 – 6.89 (m, 1H), 5.31 (dd, *J* = 9.9, 2.8 Hz, 1H), 3.46 (tdd, *J* = 10.4, 4.9, 1.1 Hz, 1H), 3.38 (ddt, *J* = 11.9, 5.5, 3.5 Hz, 1H), 2.37 (s, 3H), 2.23 – 2.14 (m, 1H), 2.06 (dtd, *J* = 13.9, 10.2, 5.6 Hz, 1H).

^{13}C NMR (126 MHz, $CDCl_3$) δ 155.1, 138.2, 136.2, 129.3, 125.7, 78.6, 38.9, 28.7, 21.2.

HRMS (GC-EI) calculated for $C_{11}H_{13}NO_2$ ($[M^+]$): 191.094079, found: 191.094073.

$[\boldsymbol{a}]_{\boldsymbol{T}}^{\boldsymbol{20}}$ = +34.06 (*c* = 0.28, $CHCl_3$).

HPLC (Chiralpak IC-3, *n*-heptane/*i*-PrOH 50:50, 298 K, 225 nm): t_R (major) = 16.3 min, t_R (minor) = 21.9 min, e.r. = 95.5:4.5 (91% e.e.).

(*R*)-6-(*o*-Tolyl)-1,3-oxazinan-2-one (3e)

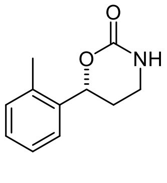

Following *General Procedure C,* employing **1a** (74 mg, 0.5 mmol, 1 equiv.) and olefin **2e** (0.65 mL, 5.0 mmol) as starting material. After chromatographic purification, compound **3e** was obtained as a white solid (77 mg, 81%).

^{1}H NMR (501 MHz, $CDCl_3$) δ 7.45 (dd, *J* = 7.0, 2.2 Hz, 1H), 7.24 (tt, *J* = 7.3, 4.9 Hz, 2H), 7.17 (dd, *J* = 6.8, 2.1 Hz, 1H), 6.83 – 6.77 (m, 1H), 5.50 (dd, *J* = 10.4, 2.6 Hz, 1H), 3.52 – 3.45 (m, 1H), 3.45 – 3.38 (m, 1H), 2.35 (s, 3H), 2.20 – 2.11 (m, 1H), 2.03 (dtd, *J* = 14.0, 10.7, 5.7 Hz, 1H).

^{13}C NMR (126 MHz, $CDCl_3$) δ 155.3, 137.1, 134.4, 130.7, 128.3, 126.5, 125.7, 76.1, 39.3, 27.7, 19.0.

HRMS (ESI) calculated for $C_{11}H_{13}NO_2Na$ ([M+Na$^+$]): 214.083847, found: 214.083794.

$[\boldsymbol{a}]_{\boldsymbol{T}}^{\boldsymbol{20}}$ = +31.84 (*c* = 0.25, $CHCl_3$).

HPLC (Chiralpak IC-3, *n*-heptane/*i*-PrOH 50:50, 298 K, 209 nm): t_R (major) = 15.7 min, t_R (minor) = 26.9 min, e.r. = 95.8:4.2 (91.6% e.e.).

(*R*)-6-(4-(*tert*-Butyl)phenyl)-1,3-oxazinan-2-one (3f)

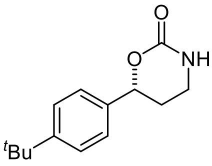

Following *General Procedure C,* employing **1a** (74 mg, 0.5 mmol, 1 equiv.) and olefin **2f** (0.91 mL, 5.0 mmol) as starting material, in $Et_2O/CHCl_3$ (3:1 *v/v*) mixture at –40 °C. After chromatographic purification, compound **3f** was obtained as a white solid (67 mg, 57%).

^{1}H NMR (501 MHz, $CDCl_3$) δ 7.46 – 7.40 (m, 2H), 7.35 – 7.30 (m, 2H), 6.83 – 6.75 (m, 1H), 5.33 (dd, *J* = 9.9, 2.8 Hz, 1H), 3.47 (dddd, *J* = 11.5, 10.2, 4.9, 1.1 Hz, 1H), 3.39 (ddt, *J* = 12.0, 5.6, 3.5 Hz, 1H), 2.21 (dddd, *J* = 13.5, 6.8, 3.7, 1.8 Hz, 1H), 2.09 (dtd, *J* = 13.9, 10.1, 5.5 Hz, 1H), 1.34 (s, 9H).

^{13}C NMR (126 MHz, $CDCl_3$) δ 155.1, 151.5, 136.1, 125.6, 125.5, 78.5, 38.9, 34.7, 31.4, 28.6.

HRMS (ESI) calculated for $C_{14}H_{19}NO_2Na$ ([M+Na$^+$]): 256.130798, found: 256.130875.

$[\boldsymbol{a}]_{\boldsymbol{T}}^{\boldsymbol{20}}$ = +23.73 (*c* = 0.30, $CHCl_3$).

HPLC (Chiralpak IC-3, *n*-heptane/*i*-PrOH 50:50, 298 K, 209 nm): t_R (major) = 13.4 min, t_R (minor) = 16.9 min, e.r. = 94:6 (88% e.e.).

(*R*)-6-(4-Fluorophenyl)-1,3-oxazinan-2-one (3g)

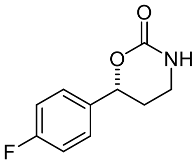

Following *General Procedure C,* employing **1a** (74 mg, 0.5 mmol, 1 equiv.) and olefin **2g** (0.60 mL, 5.0 mmol) as starting material. After chromatographic purification, compound **3g** was obtained as a white solid (69 mg, 71%).

^{1}H NMR (501 MHz, $CDCl_3$) δ 7.38 – 7.32 (m, 2H), 7.12 – 7.02 (m, 2H), 6.49 (s, 1H), 5.30 (dd, *J* = 10.2, 2.7 Hz, 1H), 3.47 (dddd, *J* = 11.7, 10.7, 4.9, 1.0 Hz, 1H), 3.38 (ddt, *J* = 12.0, 5.6, 3.4 Hz, 1H), 2.18 (ddtd, *J* = 13.9, 4.2, 3.1, 1.0 Hz, 1H), 2.05 (dtd, *J* = 13.9, 10.4, 5.6 Hz, 1H).

^{13}C NMR (126 MHz, $CDCl_3$) δ 162.8 (d, *J* = 246.9 Hz), 154.7, 134.9 (d, *J* = 3.4 Hz), 127.7 (d, *J* = 8.3 Hz), 115.71 (d, *J* = 21.7 Hz), 78.1, 39.1, 28.9.

^{19}F NMR (471 MHz, $CDCl_3$) δ -113.53.

HRMS (ESI) calculated for $C_{10}H_{10}NO_2FNa$ ([M+Na$^+$]): 218.058776, found: 218.058696.

$[\boldsymbol{a}]_{\boldsymbol{T}}^{\boldsymbol{20}}$ = +32.84 (*c* = 0.24, $CHCl_3$).

HPLC (Chiralpak IC-3, *n*-heptane/*i*-PrOH 50:50, 298 K, 209 nm): t_R (major) = 11.8 min, t_R (minor) = 16.6 min, e.r. = 95.5:4.5 (91% e.e.).

(*R*)-6-(4-Fluorophenyl)-1,3-oxazinan-2-one (3h)

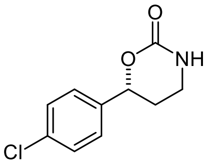

Following *General Procedure C*, employing **1a** (74 mg, 0.5 mmol, 1 equiv.) and olefin **2h** (0.63 mL, 5.0 mmol) as starting material. After chromatographic purification, compound **3h** was obtained as a white solid (58 mg, 55%).

^{1}H NMR (501 MHz, $CDCl_3$) δ 7.36 (d, *J* = 8.5 Hz, 2H), 7.31 (d, *J* = 8.6 Hz, 2H), 6.44 (s, 1H), 5.30 (dd, *J* = 10.1, 2.8 Hz, 1H), 3.51 – 3.43 (m, 1H), 3.38 (ddt, *J* = 12.0, 5.6, 3.5 Hz, 1H), 2.23 – 2.15 (m, 1H), 2.03 (dtd, *J* = 13.9, 10.3, 5.6 Hz, 1H).

^{13}C NMR (126 MHz, $CDCl_3$) δ 154.5, 137.6, 134.4, 128.9, 127.2, 78.0, 39.1, 28.8.

HRMS (ESI) calculated for $C_{10}H_{10}NO_2ClNa$ ([M+Na$^+$]): 234.029226, found: 234.029267.

$[a]_T^{20}$ = +41.46 (*c* = 0.16, $CHCl_3$).

HPLC (Chiralpak IC-3, *n*-heptane/*i*-PrOH 50:50, 298 K, 209 nm): t_R (major) = 11.6 min, t_R (minor) = 15.4 min, e.r. = 95.9:4.1 (91.8% e.e.).

(*R*)-6-(4-Bromophenyl)-1,3-oxazinan-2-one (3i)

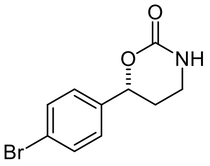

Following *General Procedure C*, employing **1a** (74 mg, 0.5 mmol, 1 equiv.) and olefin **2i** (0.63 mL, 5.0 mmol) as starting material. After chromatographic purification, compound **3i** was obtained as a white solid (77 mg, 60%).

^{1}H NMR (501 MHz, $CDCl_3$) δ 7.58 – 7.50 (m, 2H), 7.31 – 7.19 (m, 2H), 6.59 – 6.55 (m, 1H), 5.31 (dd, *J* = 10.1, 2.8 Hz, 1H), 3.49 (tdd, *J* = 10.6, 4.9, 1.1 Hz, 1H), 3.39 (ddt, *J* = 12.0, 5.6, 3.5 Hz, 1H), 2.26 – 2.16 (m, 1H), 2.04 (dtd, *J* = 13.9, 10.4, 5.6 Hz, 1H).

^{13}C NMR (126 MHz, $CDCl_3$) δ 154.5, 138.2, 131.9, 127.5, 122.5, 78.0, 39.0, 28.8.

HRMS (ESI) calculated for $C_{10}H_{10}NO_2BrNa$ ([M+Na$^+$]): 277.978723, found: 277.978836.

$[a]_T^{20}$ = +30.12 (*c* = 0.17, $CHCl_3$).

HPLC (Chiralpak IC-3, *n*-heptane/*i*-PrOH 50:50, 298 K, 220 nm): t_R (major) = 11.4 min, t_R (minor) = 15.4 min, e.r. = 96.9:3.1 (93.8% e.e.).

(*R*)-4-(2-Oxo-1,3-oxazinan-6-yl)phenyl acetate (3j)

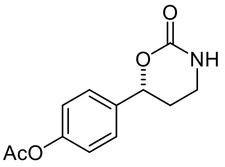

Following *General Procedure C*, employing **1a** (74 mg, 0.5 mmol, 1 equiv.) and olefin **2j** (0.77 mL, 5.0 mmol) as starting material, in $Et_2O/CHCl_3$ (3:1 *v/v*) mixture at –30 °C. After chromatographic purification, compound **3j** was obtained as a white solid (66 mg, 56%).

^{1}H NMR (501 MHz, $CDCl_3$) δ 7.37 (d, *J* = 8.5 Hz, 2H), 7.09 (d, *J* = 8.6 Hz, 2H), 6.75 – 6.71 (s, 1H), 5.30 (dd, *J* = 10.1, 2.7 Hz, 1H), 3.43 (dddd, *J* = 11.7, 10.6, 4.8, 1.0 Hz, 1H), 3.39 – 3.29 (m, 1H), 2.28 (s, 3H), 2.21 – 2.12 (m, 1H), 2.02 (dtd, *J* = 13.9, 10.4, 5.6 Hz, 1H).

^{13}C NMR (126 MHz, $CDCl_3$) δ 169.5, 154.8, 150.6, 136.7, 126.9, 121.9, 78.1, 38.9, 28.8, 21.2.

HRMS (ESI) calculated for $C_{12}H_{13}NO_4Na$ ([M+Na$^+$]): 258.073678, found: 258.073665.

$[a]_T^{20}$ = +36.24 (*c* = 0.15, $CHCl_3$).

HPLC (Chiralpak IC-3, *n*-heptane/*i*-PrOH 50:50, 298 K, 215 nm): t_R (major) = 23.8 min, t_R (minor) = 35.3 min, e.r. = 95.1:4.9 (90.2% e.e.).

(*R*)-6-(4-(Chloromethyl)phenyl)-1,3-oxazinan-2-one (3k)

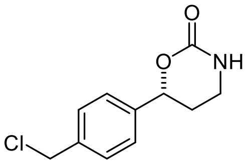

Following *General Procedure C*, employing **1a** (74 mg, 0.5 mmol, 1 equiv.) and olefin **2k** (0.70 mL, 5.0 mmol) as starting material. After chromatographic purification, compound **3k** was obtained as a white solid (57 mg, 51%).

^{1}H NMR (501 MHz, $CDCl_3$) δ 7.47 – 7.33 (m, 4H), 5.97 (s, 1H), 5.35 (dd, *J* = 9.9, 2.8 Hz, 1H), 4.59 (s, 2H), 3.49 (td, *J* = 11.0, 4.9 Hz, 1H), 3.39 (ddt, *J* = 12.2, 6.1, 3.6 Hz, 1H), 2.22 (dq, *J* = 14.2, 3.9 Hz, 1H), 2.14 – 2.01 (m, 1H).

^{13}C NMR (126 MHz, $CDCl_3$) δ 154.4, 139.4, 137.8, 129.0, 126.2, 78.3, 45.9, 39.2, 28.9.

HRMS (ESI) calculated for $C_{11}H_{12}NO_2ClNa$ ($[M+Na^+]$): 248.044876, found: 258.044939.

$[\boldsymbol{a}]_{\boldsymbol{T}}^{\mathbf{20}}$ = +43.29 (*c* = 0.12, $CHCl_3$).

HPLC (Chiralpak IC-3, *n*-heptane/*i*-PrOH 50:50, 298 K, 220 nm): t_R (major) = 16.0 min, t_R (minor) = 20.9 min, e.r. = 98.8:1.2 (97.6% e.e.).

(*R*)-6-(3-Methoxyphenyl)-1,3-oxazinan-2-one (3l)

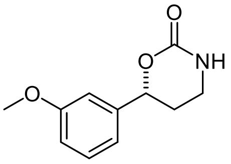

Following *General Procedure C,* employing **1a** (74 mg, 0.5 mmol, 1 equiv.) and olefin **2l** (0.69 mL, 5.0 mmol) as starting material. After chromatographic purification, compound **3l** was obtained as a white solid (68 mg, 67%).

^{1}H NMR (501 MHz, $CDCl_3$) δ 7.36 – 7.21 (m, 1H), 6.98 – 6.91 (m, 2H), 6.90 – 6.79 (m, 1H), 6.47 (s, 1H), 5.31 (dd, *J* = 9.8, 2.8 Hz, 1H), 3.82 (s, 3H), 3.47 (dddd, *J* = 11.7, 10.4, 4.9, 1.2 Hz, 1H), 3.38 (ddt, *J* = 11.6, 5.5, 3.6 Hz, 1H), 2.26 – 2.17 (m, 1H), 2.07 (dtd, *J* = 13.9, 10.2, 5.6 Hz, 1H).

^{13}C NMR (126 MHz, $CDCl_3$) δ 159.9, 154.8, 140.8, 129.8, 117.9, 114.0, 111.3, 78.5, 55.5, 39.1, 28.9.

HRMS (ESI) calculated for $C_{11}H_{13}NO_3Na$ ($[M+Na^+]$): 230.078763, found: 230.078873.

$[\boldsymbol{a}]_{\boldsymbol{T}}^{\mathbf{20}}$ = +37.29 (*c* = 0.12, $CHCl_3$).

HPLC (Chiralpak IC-3, *n*-heptane/*i*-PrOH 50:50, 298 K, 209 nm): t_R (major) = 15.8 min, t_R (minor) = 22.1 min, e.r. = 95.7:4.3 (92% e.e.).

(*R*)-6-(3-Bromophenyl)-1,3-oxazinan-2-one (3m)

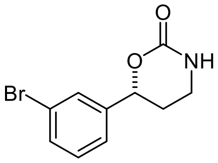

Following *General Procedure C,* employing **1a** (74 mg, 0.5 mmol, 1 equiv.) and olefin **2m** (0.65 mL, 5.0 mmol) as starting material. After chromatographic purification, compound **3m** was obtained as a white solid (55 mg, 43%).

^{1}H NMR (501 MHz, $CDCl_3$) δ 7.48 (t, *J* = 1.9 Hz, 1H), 7.40 (dt, *J* = 7.8, 1.6 Hz, 1H), 7.27 – 7.12 (m, 2H), 6.47 (s, 1H), 5.23 (dd, *J* = 10.1, 2.8 Hz, 1H), 3.41 (dddd, *J* = 11.7, 10.5, 4.9, 1.1 Hz, 1H), 3.32 (ddt, *J* = 11.9, 5.6, 3.4 Hz, 1H), 2.19 – 2.09 (m, 1H), 1.97 (dtd, *J* = 14.0, 10.4, 5.6 Hz, 1H).

^{13}C NMR (126 MHz, $CDCl_3$) δ 154.4, 141.4, 131.1, 130.4, 128.9, 124.4, 122.9, 77.8, 39.0, 28.9.

HRMS (GC-EI) calculated for $C_{10}H_{1}NO_{2}Br$ ($[M^+]$): 254.988954, found: 254.988967.

$[\boldsymbol{a}]_{\boldsymbol{T}}^{\mathbf{20}}$ = +43.77 (c = 0.20, $CHCl_3$).

HPLC (Chiralpak IC-3, *n*-heptane/*i*-PrOH 50:50, 298 K, 270 nm): t_R (major) = 11.4 min, t_R (minor) = 15.5 min, e.r. = 97.4:2.6 (94.5% e.e.).

(*R*)-6-(3-Bromo-4-methylphenyl)-1,3-oxazinan-2-one (3n)

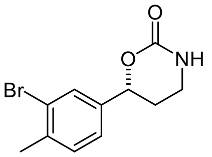

Following *General Procedure C,* employing **1a** (74 mg, 0.5 mmol, 1 equiv.) and olefin **2n** (0.65 mL, 5.0 mmol) as starting material. After chromatographic purification, compound **3n** was obtained as a white solid (68 mg, 71%).

^{1}H NMR (501 MHz, $CDCl_3$) δ 7.49 (d, J = 1.8 Hz, 1H), 7.24 – 7.07 (m, 2H), 5.97 (s, 1H), 5.21 (dd, J = 10.0, 2.8 Hz, 1H), 3.41 (td, J = 11.0, 4.9 Hz, 1H), 3.32 (ddt, J = 11.9, 5.6, 3.5 Hz, 1H), 2.33 (s, 3H), 2.17 – 2.09 (m, 1H), 1.98 (dtd, J = 14.0, 10.3, 5.6 Hz, 1H).

^{13}C NMR (126 MHz, $CDCl_3$) δ 154.3, 138.5, 138.2, 131.1, 129.7, 125.2, 124.7, 77.7, 39.1, 28.8, 22.8.

HRMS (GC-EI) calculated for $C_{11}H_{12}NO_{2}Br$ ($[M^+]$): 269.004604, found: 269.004583.

$[\boldsymbol{a}]_{\boldsymbol{T}}^{\mathbf{20}}$ = +40.00 (c = 0.12, $CHCl_3$).

HPLC (Chiralpak IC-3, *n*-heptane/*i*-PrOH 50:50, 298 K, 220 nm): t_R (major) = 13.2 min, t_R (minor) = 16.5 min, e.r. = 97.1:2.9 (94.2% e.e.).

(*R*)-6-(Thiophen-3-yl)-1,3-oxazinan-2-one (3o)

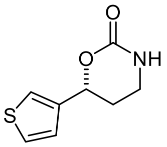

Following *General Procedure C,* employing **1a** (74 mg, 0.5 mmol, 1 equiv.) and olefin **2o** (0.37 mL, 5.0 mmol) as starting material, in MTBE at –30 °C. After chromatographic purification, compound **3o** was obtained as a white solid (71 mg, 78%).

^{1}H NMR (501 MHz, $CDCl_3$) δ 7.37 – 7.28 (m, 2H), 7.07 (dd, J = 5.0, 1.4 Hz, 1H), 6.68 (s, 1H), 5.41 (dd, J = 9.5, 2.9 Hz, 1H), 3.48 – 3.32 (m, 2H), 2.24 (dq, J = 11.9, 3.8 Hz, 1H), 2.10 (dtd, J = 13.9, 9.7, 5.6 Hz, 1H).

^{13}C NMR (126 MHz, $CDCl_3$) δ 154.7, 140.3, 126.7, 125.3, 122.0, 75.3, 38.8, 27.8.

HRMS (ESI) calculated for $C_8H_9NO_2SNa$ ($[M+Na^+]$): 206.024620 found: 206.024649.

$[\boldsymbol{a}]_{\boldsymbol{T}}^{\mathbf{20}}$ = +1.40 (c = 0.14, $CHCl_3$).

HPLC (Chiralpak IC-3, *n*-heptane/*i*-PrOH 50:50, 298 K, 233 nm): t_R (major) = 14.3 min, t_R (minor) = 16.6 min, e.r. = 93:7 (86% e.e.).

(*R*)-6-(Benzo[*b*]thiophen-6-yl)-1,3-oxazinan-2-one (3p)

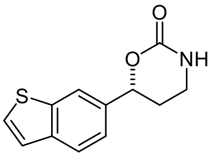

Following *General Procedure C,* employing **1a** (44 mg, 0.3 mmol, 1 equiv.) and olefin **2p** (480 mg, 3.0 mmol) as starting material, in $Et_2O/CHCl_3$ (3:1 *v/v*) mixture at –30 °C. After chromatographic purification, compound **3p** was obtained as a white solid (53 mg, 76%).

^{1}H NMR (501 MHz, $CDCl_3$) δ 7.96 – 7.92 (m, 1H), 7.83 (d, *J* = 8.2 Hz, 1H), 7.47 (d, *J* = 5.4 Hz, 1H), 7.37 – 7.30 (m, 2H), 6.28 (s, 1H), 5.47 (dd, *J* = 9.9, 2.8 Hz, 1H), 3.50 (dddd, *J* = 11.6, 10.3, 4.8, 1.1 Hz, 1H), 3.40 (ddt, *J* = 11.9, 5.5, 3.6 Hz, 1H), 2.32 – 2.23 (m, 1H), 2.14 (dtd, *J* = 13.9, 10.2, 5.5 Hz, 1H).

^{13}C NMR (126 MHz, $CDCl_3$) δ 154.6, 140.2, 139.7, 135.4, 127.4, 123.9, 123.7, 122.1, 119.8, 78.8, 39.2, 29.2.

HRMS (GC-EI) calculated for $C_{12}H_{11}NO_2S$ ([M$^+$]): 233.050501, found: 233.050416.

$[\boldsymbol{a}]_{\boldsymbol{T}}^{\boldsymbol{20}}$ = +52.94 (*c* = 0.14, $CHCl_3$).

HPLC (Chiralpak IC-3, *n*-heptane/*i*-PrOH 50:50, 298 K, 209 nm): t_R (major) = 17.1 min, t_R (minor) = 22.4 min, e.r. = 95.5:4.5 (91% e.e.).

(*R*)-6-(Benzofuran-6-yl)-1,3-oxazinan-2-one (3q)

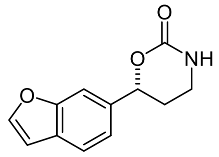

Following *General Procedure C*, employing **1a** (37 mg, 0.25 mmol, 1 equiv.) and olefin **2q** (360 mg, 2.5 mmol) as starting material. After chromatographic purification, compound **3q** was obtained as a white solid (47 mg, 87%).

^{1}H NMR (501 MHz, $CDCl_3$) δ 7.64 (d, *J* = 2.2 Hz, 1H), 7.62 – 7.54 (m, 2H), 7.23 (dd, *J* = 8.2, 1.5 Hz, 1H), 6.76 (d, *J* = 2.1 Hz, 1H), 6.57 (s, 1H), 5.44 (dd, *J* = 10.0, 2.8 Hz, 1H), 3.49 (td, *J* = 11.1, 4.8 Hz, 1H), 3.40 (ddt, *J* = 11.9, 5.6, 3.5 Hz, 1H), 2.24 (ddt, *J* = 14.1, 5.4, 3.2 Hz, 1H), 2.12 (dtd, *J* = 13.9, 10.2, 5.6 Hz, 1H).

^{13}C NMR (126 MHz, $CDCl_3$) δ 155.08, 154.82, 145.90, 135.75, 127.63, 121.41, 120.63, 109.02, 106.54, 78.83, 39.09, 29.21.

HRMS (GC-EI) calculated for $C_{12}H_{11}NO_3$ ([M$^+$]): 217.073343, found: 217.073229.

$[\boldsymbol{a}]_{\boldsymbol{T}}^{\boldsymbol{20}}$ = +41.51 (*c* = 0.11, $CHCl_3$).

HPLC (Chiralpak IC-3, *n*-heptane/*i*-PrOH 50:50, 298 K, 254 nm): t_R (major) = 16.3 min, t_R (minor) = 21.3 min, e.r. = 93:7 (86% e.e.).

(*R*)-6-Ethyl-6-phenyl-1,3-oxazinan-2-one (3r)

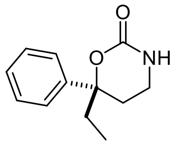

Following *General Procedure C*, employing **1a** (57 mg, 0.4 mmol, 1 equiv.) and olefin **2r** (528 mg, 4.0 mmol) as starting material, in $Et_2O/CHCl_3$ (3:1 *v/v*) mixture at –30 °C. After chromatographic purification, compound **3r** was obtained as a white solid (59 mg, 71%).

^{1}H NMR (501 MHz, $CDCl_3$) δ 7.38 (dd, *J* = 8.4, 6.9 Hz, 2H), 7.34 – 7.26 (m, 3H), 5.10 (s, 1H), 3.23 (ddt, *J* = 11.9, 6.3, 3.1 Hz, 1H), 2.96 (td, *J* = 11.5, 4.7 Hz, 1H), 2.33 – 2.26 (m, 1H), 2.18 (ddd, *J* = 13.9, 11.5, 5.6 Hz, 1H), 1.95 (ddt, *J* = 25.4, 14.2, 7.1 Hz, 2H), 0.84 (t, *J* = 7.4 Hz, 3H).

^{13}C NMR (126 MHz, $CDCl_3$) δ 141.6, 128.8, 127.7, 125.0, 37.3, 35.9, 30.6, 7.6. (other signals not detected or observed).

HRMS (ESI) calculated for $C_{12}H_{15}NO_2Na$ ([M+Na$^+$]): 228.099498, found: 228.099418.

$[\boldsymbol{a}]_{\boldsymbol{T}}^{\boldsymbol{20}}$ = +89.17 (*c* = 0.16, $CHCl_3$).

HPLC (Chiralpak IE-3, *n*-heptane/*i*-PrOH 80:20, 298 K, 209 nm): t_R (minor) = 10.9 min, t_R (major) = 11.7 min, e.r. = 5.3:94.7 (89.4% e.e.).

(*S*)-6-Isopropyl-6-phenyl-1,3-oxazinan-2-one (3s)

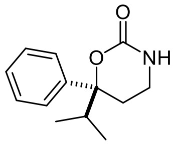

Following *General Procedure C,* employing **1a** (57 mg, 0.4 mmol, 1 equiv.) and olefin **2s** (528 mg, 4.0 mmol) as starting material. After chromatographic purification, compound **3s** was obtained as a white solid (38 mg, 43%).

^{1}H NMR (501 MHz, $CDCl_3$) δ 7.40 – 7.33 (m, 2H), 7.33 – 7.23 (m, 3H), 5.78 (s, 1H), 3.21 (dddd, *J* = 11.7, 5.9, 4.0, 2.0 Hz, 1H), 2.91 (td, *J* = 11.9, 4.6 Hz, 1H), 2.33 (ddt, *J* = 14.0, 4.6, 1.7 Hz, 1H), 2.22 (ddd, *J* = 13.9, 12.3, 5.7 Hz, 1H), 2.08 (hept, *J* = 6.8 Hz, 1H), 0.96 (d, *J* = 6.8 Hz, 3H), 0.85 (d, *J* = 6.9 Hz, 3H).

^{13}C NMR (126 MHz, $CDCl_3$) δ 154.7, 140.6, 128.6, 127.6, 125.7, 86.9, 38.6, 37.2, 27.7, 17.1, 16.7.

HRMS (ESI) calculated for $C_{13}H_{17}NO_2Na$ ([M+Na$^+$]): 242.115148, found: 242.115159.

$[\boldsymbol{a}]_{\boldsymbol{T}}^{\boldsymbol{20}}$ = +75.47 (*c* = 0.16, $CHCl_3$).

HPLC (Chiralpak ID-3, *n*-heptane/*i*-PrOH 91:10, 298 K, 209 nm): t_R (major) = 10.6 min, t_R (minor) = 11.7 min, e.r. = 98.7:1.3 (97.4% e.e.).

(*S*)-6-Cyclopentyl-6-phenyl-1,3-oxazinan-2-one (3t)

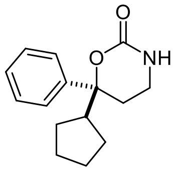

Following *General Procedure C,* employing **1a** (34 mg, 0.25 mmol, 1 equiv.) and olefin **2t** (400 mg, 2.5 mmol) as starting material, in $Et_2O/CHCl_3$ (3:1 *v/v*) mixture at –30 °C. After chromatographic purification, compound **3t** was obtained as a white solid (13 mg, 23%).

^{1}H NMR (501 MHz, $CDCl_3$) δ 7.34 – 7.16 (m, 5H), 5.84 (s, 1H), 3.10 (dddd, *J* = 11.7, 7.1, 4.3, 2.4 Hz, 1H), 2.79 (tt, *J* = 11.5, 5.5 Hz, 1H), 2.30 – 2.12 (m, 3H), 1.72 (dh, *J* = 11.2, 3.1 Hz, 1H), 1.60 – 1.25 (m, 6H), 1.13 (ddt, *J* = 10.8, 7.3, 4.0 Hz, 1H).

^{13}C NMR (126 MHz, $CDCl_3$) δ 154.9, 142.1, 128.7, 127.5, 125.2, 85.7, 51.1, 37.0, 29.8, 26.7, 26.5, 25.6, 25.2.

HRMS (ESI) calculated for $C_{15}H_{19}NO_2Na$ ([M+Na$^+$]): 268.13080, found: 268.13117.

$[\boldsymbol{a}]_{\boldsymbol{T}}^{\boldsymbol{20}}$ = +76.77 (*c* = 0.15, $CHCl_3$).

HPLC (Chiralpak ID-3, *n*-heptane/*i*-PrOH 80:20, 298 K, 220 nm): t_R (major) = 7.3 min, t_R (minor) = 9.2 min, e.r. = 94.8:5.2 (89.6% e.e.).

(*S*)-6-Cyclohexyl-6-phenyl-1,3-oxazinan-2-one (3u)

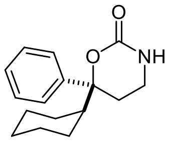

Following *General Procedure C,* employing **1a** (59 mg, 0.4 mmol, 1 equiv.) and olefin **2u** (745 mg, 4.0 mmol) as starting material. After chromatographic purification, compound **3u** was obtained as a white solid (58 mg, 56%).

^{1}H NMR (501 MHz, $CDCl_3$) δ 7.29 (dd, *J* = 8.4, 6.3 Hz, 2H), 7.26 – 7.16 (m, 3H), 5.40 (d, *J* = 3.7 Hz, 1H), 3.13 (dddd, *J* = 11.5, 5.9, 4.0, 2.0 Hz, 1H), 2.84 (td, *J* = 11.9, 4.7 Hz, 1H), 2.26 (ddt, *J* = 14.0, 4.7, 1.7 Hz, 1H), 2.16 (ddd, *J* = 13.9, 12.2, 5.7 Hz, 1H), 1.87 (dt, *J* = 12.7, 3.3 Hz, 1H), 1.64 (dtt, *J* = 14.9, 9.3, 3.1 Hz, 3H), 1.57 – 1.42 (m, 3H), 1.14 – 0.83 (m, 5H).

^{13}C NMR (126 MHz, $CDCl_3$) δ 154.5, 140.8, 128.5, 127.6, 125.8, 86.9, 48.5, 37.2, 27.5, 26.9, 26.7, 26.6, 26.4, 26.3.

HRMS (GC-EI) calculated for $C_{16}H_{22}NO_2$ ([M$^+$]): 260.164504, found: 260.164391.

$[\boldsymbol{a}]_{\boldsymbol{T}}^{\boldsymbol{20}}$ = +76.36 (*c* = 0.11, $CHCl_3$).

HPLC (Chiralpak ID-3, *n*-heptane/*i*-PrOH 80:20, 298 K, 209 nm): t_R (major) = 8.1 min, t_R (minor) = 8.9 min, e.r. = 96.7:3.3 (93.4% e.e.).

(*R*)-6-Isopropyl-6-methyl-1,3-oxazinan-2-one (3v)

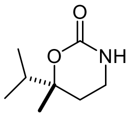

Following *General Procedure C,* employing **1a** (74 mg, 0.5 mmol, 1 equiv.) and olefin **2v** (0.62 mL, 5.0 mmol) as starting material, in $Et_2O/CHCl_3$ (3:1 *v/v*) mixture at –10 °C. After chromatographic purification, compound **3v** was obtained as a white solid (47 mg, 60%).

^{1}H NMR (501 MHz, $CDCl_3$) δ 6.62 (s, 1H), 3.33 (ddqd, *J* = 9.9, 6.8, 5.1, 1.9 Hz, 2H), 1.94 (p, *J* = 6.8 Hz, 1H), 1.90 – 1.81 (m, 1H), 1.71 (dt, *J* = 13.8, 4.9 Hz, 1H), 1.26 (s, 3H), 0.96 (dd, *J* = 35.4, 6.9 Hz, 6H).

^{13}C NMR (126 MHz, $CDCl_3$) δ 154.9, 83.3, 36.6, 36.5, 27.6, 20.2, 17.3, 16.7.

HRMS (ESI) calculated for $C_{15}H_{19}NO_2Na$ ([M+Na$^+$]): 180.099497, found: 180.099440.

$[\boldsymbol{a}]_{\boldsymbol{T}}^{\boldsymbol{20}}$ = +1.82 (*c* = 0.22, $CHCl_3$).

GC 30.0 m BGB-174, injection temperature: 220 °C, 170 °C iso 30 min, 240 °C iso 10 min, 0.6 bar H_2): t_R (major) = 25.0 min, t_R (minor) = 16.6 min, e.r. = 93.2:6.8 (86.4% e.e.).

(*R*)-6-(*tert*-Butyl)-6-methyl-1,3-oxazinan-2-one (3w)

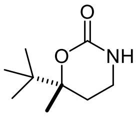

Following *General Procedure C,* employing **1a** (74 mg, 0.5 mmol, 1 equiv.) and olefin **2w** (0.70 mL, 5.0 mmol) as starting material, in $Et_2O/CHCl_3$ (3:1 *v/v*) mixture at –10 °C. After chromatographic purification, compound **3w** was obtained as a white solid (30 mg, 35%).

^{1}H NMR (501 MHz, $CDCl_3$) δ 5.84 (s, 1H), 3.41 (td, *J* = 12.1, 4.7 Hz, 1H), 3.33 (dddd, *J* = 12.0, 6.1, 4.1, 1.7 Hz, 1H), 2.03 (td, *J* = 13.1, 6.4 Hz, 1H), 1.67 (ddt, *J* = 13.6, 4.7, 1.4 Hz, 1H), 1.37 – 1.28 (m, 3H), 1.02 (s, 9H).

^{13}C NMR (126 MHz, $CDCl_3$) δ 154.9, 84.9, 37.8, 36.9, 25.3, 25.0, 18.8.

HRMS (ESI) calculated for $C_9H_{17}NO_2Na$ ([M+Na$^+$]): 194.115150, found: 194.115212.

$[\boldsymbol{a}]_{\boldsymbol{T}}^{\boldsymbol{20}}$ = +4.92 (*c* = 0.12, $CHCl_3$).

GC 25.0 m Hydrodex beta-TBDAc, injection temperature: 220 °C, 150 °C iso 45 min, 8°C/min, 220 °C, 0.6 bar H_2): t_R (major) = 39.1 min, t_R (minor) = 41.3 min, e.r. = 97.8:2.2 (95.6% e.e.).

5. Absolute Configuration Determination

The absolute configuration of **3a** was determined by comparison of the optical rotation with available literature data.[15] Similarly, the absolute configuration of **3s** was established after transformation to amino alcohol **S13** and comparison of its optical rotation with available literature data.[16] Other products' absolute configuration were assigned by analogy.

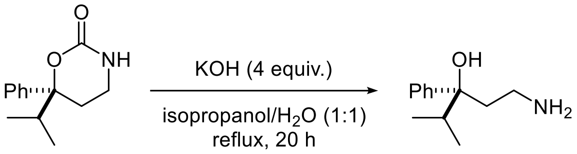

3s 88%, **S13**

Compound	Measured $[\alpha]_D$	Literature value
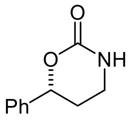<br>**3a**	$[\alpha]_T^{20}$ = +50.44 (c = 0.23, $CHCl_3$).<br>(*R*)	$[\alpha]_T^{20}$ = +30.20 (c = 1.0, $CHCl_3$).<br>(*R*)[15]
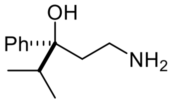<br>**S13**	$[\alpha]_T^{25}$ = +10.21 (c = 0.12, EtOH).<br>(*S*)	$[\alpha]_T^{25}$ = –3.36 (c = 3.3, EtOH).<br>(*R*)[16]

6. Scale-Up Experiments

6.1. Catalyst and Olefin Recovery Experiment

An oven-dried Schleck flask equipped with a magnetic stir bar was charged with carbamate **1a** (735 mg, 5.0 mmol, 1 equiv.), olefin **2a** (3.7 mL, 50 mmol) and dry $CHCl_3$ (12 mL). The flask was sealed and cooled down to –25 °C for 30 min. After this time, a stock solution of catalyst **6b** in 4 mL of $CHCl_3$ (42 mg, 0.5 mol%) was added dropwise via syringe. After the reaction was completed (TLC monitoring) it was quenched with one equivalent of triethylamine and stirred for 30 min at rt. The reaction crude mixture was transferred to a round-bottomed flask to facilitate manipulation. A short distillation apparatus was attached to the flask and $CHCl_3$ and triethylamine were distilled off the crude under vacuum (100 mbar, rt). The receiving flask was changed and the non-reacted styrene was further recovered by bulb-to-bulb vacuum distillation (94% styrene recovered, 1 mbar, 35 °C). The crude residue was purified by silica gel column chromatography (DCM/MeOH mixtures from 0.5 to 3%) to give 545 mg of **3a** as a white solid (62%, e.r. = 96.5:3.5). Fractions containing IDPi catalyst were combined and purified by silica gel column chromatography (*n*-hexane/EtOAc 9:1 to 4:1) to afford a white solid, which was subjected to acidification by filtration over a plug of DOWEX 50WX8 (H-form, eluted with DCM) to obtain the reisolated catalyst **6b** (30 mg, 72%) as a yellowish solid.

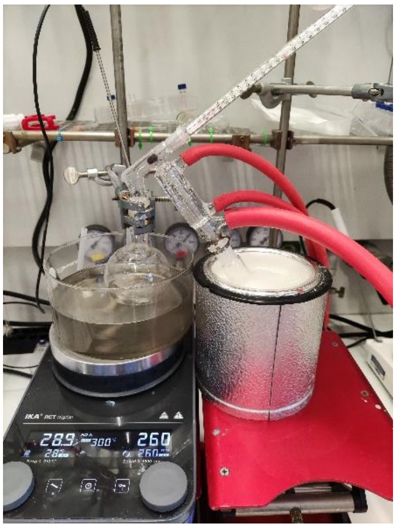

Figure SI-2. Distillation of the non-reacted styrene under vacuum.

6.2. Formal Synthesis of (*R*)-Fluoxetine Hydrochloride from Styrene 2a

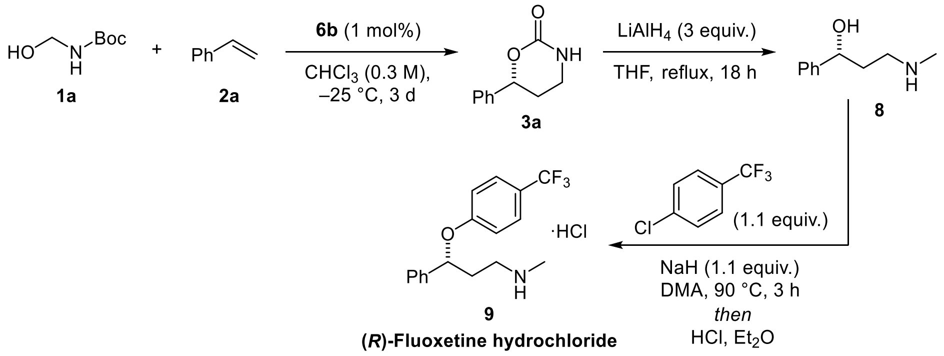

(*R*)-6-Phenyl-1,3-oxazinan-2-one (3a)

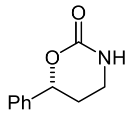

Following General Procedure C: an oven-dried Schleck flask equipped with a magnetic stir bar was charged with carbamate **1a** (2.5 g, 17 mmol, 1 equiv.), olefin **2a** (19.5 mL, 170 mmol) and dry $CHCl_3$ (40 mL). The flask was sealed and cooled down to –25 °C for 30 min. After this time, a stock solution of catalyst **6b** in 14 mL of $CHCl_3$ (283 mg, 1 mol%) was added dropwise via syringe. After the reaction was completed (TLC monitoring) it was quenched with one equivalent of triethylamine and stirred for 30 min at rt. The mixture was suspended on Celite and further purified by silica gel column chromatography (DCM/MeOH mixtures from 0.5 to 3%) to give 2.20 g **3a** as a white solid (73%, e.r. = 96.5:3.5).

(*R*)-3-(Methylamino)-1-phenylpropan-1-ol (8)

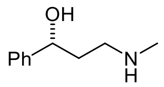

Following a reported procedure:[14] to a two-necked round-bottomed oven-dried Schleck flask charged with product **3a** (2.20 g, 73 mmol, 1 equiv.) and dry THF (124 mL) was added $LiAlH_4$ (1.4 mg, 37 mmol, 3 equiv.) under argon at 0 °C. The mixture was refluxed overnight. After cooling down to rt, the reaction was diluted with MTBE (30 mL), and cooled down to 0 °C. $LiAlH_4$ is quenched by a careful addition of water (3 mL), followed by an aqueous NaOH 15% solution (3 mL), and water (15 mL). The mixture is warmed up to rt and stirred for 30 min. Then it was transferred to a separatory funnel and both phases were separated. The aqueous phase was extracted with MTBE (3x10 mL). Combined organic phases were washed with brine (1x10 mL), dried over anhydrous Na_2SO_4 and evaporated to give compound **8** as a pure pale yellow oil (1.86 g, 91%) without further purification. Spectroscopic data was consistent with the values reported in the literature.[14]

^{1}H NMR (501 MHz, $CDCl_3$) δ 7.44 – 7.32 (m, 4H), 7.30 – 7.22 (m, 1H), 4.96 (dd, *J* = 8.8, 3.1 Hz, 1H), 2.96 – 2.83 (m, 2H), 2.47 (s, 3H), 1.90 (ddt, *J* = 14.5, 6.1, 3.3 Hz, 1H), 1.78 (dtd, *J* = 14.5, 9.1, 3.7 Hz, 1H).

^{13}C NMR (126 MHz, $CDCl_3$) δ 145.2, 128.2, 126.9, 125.6, 75.3, 50.3, 37.0, 36.0.

(*R*)-Fluoxetine hydrochloride (9)

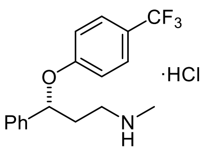

Following a reported procedure:[17] in a flame-dried Schlenk under argon atmosphere, (*R*)-3-(methylamino)-1-phenylpropan-1-ol **8** (1.60 g, 9.7 mmol, 1 equiv.) was dissolved in 9 mL of dry dimethylacetamide. The mixture was cooled to 0 °C and sodium hydride (0.46 g, 11.6, 1.2 equiv., 60% dispersion in paraffin liquid) was added slowly. The mixture was heated at 90 °C for 1.5 h, and an orange solution resulted. To this solution was added 4-chlorobenzotrifluoride (1.92 g, 10.7 mmol, 1.1 equiv.), and the mixture was heated at 105°C for 2 h. After cooling to rt and dilution with EtOAc (10 mL), the mixture was washed with water (5 mL), and the aqueous layer was separated and extracted with EtOAc (3x5 mL). The combined organic solutions were washed with sat. aq. $NaHCO_3$ (1x10 mL) and brine (1x10 mL), dried over anhydrous Na_2SO_4 and evaporated. The crude free base Fluoxetine was dissolved in 10 mL Et_2O and acidified with gaseous HCl to afford 3.05 g (91% yield) of (*R*)-Fluoxetine hydrochloride as pale yellow crystals. Spectroscopic data was consistent with the values reported in the literature.[17]

^{1}H NMR (501 MHz, $CDCl_3$) δ 9.72 (s, 2H), 7.41 (d, *J* = 8.6 Hz, 5H), 7.36 – 7.22 (m, 5H), 6.90 (d, *J* = 8.5 Hz, 2H), 5.47 (dd, *J* = 8.3, 4.3 Hz, 1H), 3.18 – 3.05 (m, 2H), 2.61 (t, *J* = 5.5 Hz, 3H), 2.57 – 2.39 (m, 2H).

^{13}C NMR (126 MHz, $CDCl_3$) δ 159.8, 139.2, 129.2, 128.6, 126.9 (q, *J* = 3.6 Hz), 125.9, 116.0, 77.1, 46.2, 34.7, 33.1.

^{19}F NMR (471 MHz, $CDCl_3$) δ -61.67.

$[\boldsymbol{a}]_{\boldsymbol{T}}^{\mathbf{20}}$ = –13.8 (c = 0.19, $CHCl_3$).

HPLC (Chiralpak IG-3, 70:30 MeOH/20 mM NH_4HCO_3 aq. pH = 9, 298 K, 220 nm): t_R (major) = 8.8 min, t_R (minor) = 11.1 min, e.r. = 96.5:3.5 (93% e.e.).

7. Synthesis of (*S*,*S*)-IDPi Catalysts

(*S*,*S*)-IDPi catalysts **S11, S12** and **4–7** were prepared following modified literature procedures.[18-20] Phosphazene reagent **S14** was prepared following reported literature procedures.[21]

4-((3*r*,5*r*,7*r*)-adamantan-1-yl)phenol (S15)

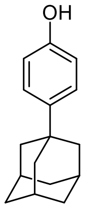

Following a reported procedure:[22] to a solution of phenol (1.00 g, 10.6 mmol. 1 equiv.) in trifluoroacetic acid (32 mL) was added 1-adamantol (1.61 g, 10.6 mmol, 1 equiv.) in a two-necked round-bottomed flask under argon. The suspension was stirred for 6 h at rt. After this time, the mixture was diluted with H_2O (50 mL) and the precipitate was filtered off, washed with an aqueous solution of Na_2CO_3 (until pH = 7) and dried to give product **S15** (2.40 g, quant.) as a colorless solid. Spectroscopic data was consistent with the values reported in the literature.[22]

^{1}H NMR (501 MHz, $CDCl_3$) δ 7.30 – 7.16 (m, 2H), 6.78 (d, *J* = 8.7 Hz, 2H), 4.77 (s, 1H), 2.14 – 2.02 (m, 3H), 1.88 (d, *J* = 2.9 Hz, 6H), 1.82 – 1.66 (m, 6H).

4-((3*r*,5*r*,7*r*)-adamantan-1-yl)phenyl trifluoromethanesulfonate (S16)

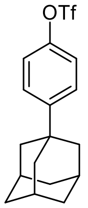

Following a reported procedure:[23] in a flame-dried Schlenk under argon atmosphere, substrate **S15** (2.41 g, 10.5 mmol, 1 equiv.) was dissolved in 67 mL of dry DCM. The solution was cooled down to 0 °C using an ice water bath and triethylamine (8.8 mL, 6 equiv.) was added. After stirring for 10 min, trifluoromethanesulfonic anhydride (3.5 mL, 21.0 mmol, 2 equiv.) was added dropwise. The solution was stirred at 0 °C for 30 min, then warmed up to rt, quenched with aqueous HCl 10% (50 mL) and the layers were separated. The aqueous layer was extracted with DCM (3x20 mL) and the combined organic extracts were washed with water (1x20 mL), dried over anhydrous Na_2SO_4, filtered and concentrated. The crude residue was purified by silica gel column chromatography using *n*-hexane/EtOAc (15:1) as eluent to afford 2.53 g (67% yield) of product **S16**. Spectroscopic data was consistent with the values reported in the literature.[23]

^{1}H NMR (501 MHz, $CDCl_3$) δ 7.41 (d, *J* = 8.8 Hz, 2H), 7.20 (d, *J* = 8.8 Hz, 2H), 2.18 – 2.04 (m, 3H), 1.89 (d, *J* = 3.0 Hz, 6H), 1.85 – 1.63 (m, 6H).

^{13}C NMR (126 MHz, $CDCl_3$) δ 151.9, 147.6, 126.9, 120.8, 119.0 (q, $^1J_{CF}$ = 320 Hz, CF_3), 43.2, 36.7, 36.4, 28.9.

^{19}F NMR (471 MHz, $CDCl_3$) δ –72.94.

7.1. Synthesis of substituted (*S*)-BINOLs

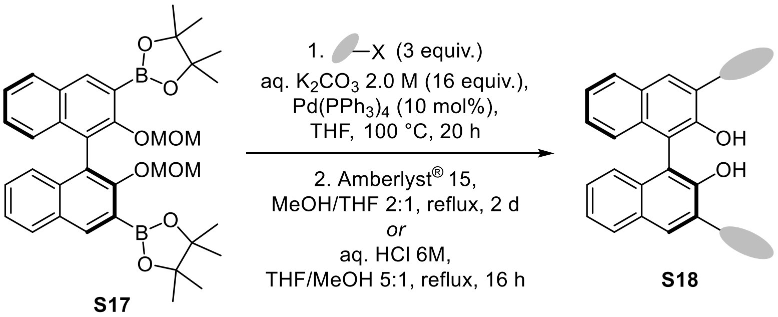

S17 **S18**

(*S*)-3,3'-bis(4-((3r,5r,7r)-adamantan-1-yl)phenyl)-[1,1'-binaphthalene]-2,2'-diol (S18b)

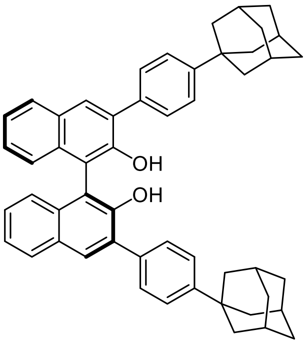

A flame-dried Schlenk under argon atmosphere was charged with (*S*)-MOM-BINOL Bpin ester **S17** (626 mg, 1 mmol, 1 equiv.) and triflate **S16** (1.08 g, 3 mmol, 3 equiv.). THF (10 mL) and K_2CO_3 (2.0 M in H_2O, 8 mL, 16 mmol, 16 equiv.) were added and the solution was sparged with argon for 20 min. Subsequently, $Pd(PPh_3)_4$ (116 mg, 0.1 mmol, 10 mol%) was added and the reaction was heated to 100 ºC for 20 h. After cooling to rt, the reaction was diluted with water (10 mL) and the aqueous layer was extracted with DCM (3x15 mL). The combined organic phases were washed with brine, dried over anhydrous Na_2SO_4, filtered and concentrated under reduced pressure. The crude residue was purified by silica gel column chromatography using *n*-hexane/EtOAc mixtures as eluent. The pure white-off solid was transferred to a round-bottomed flask and dissolved in 30 mL MeOH/THF 2:1 (*v/v*). 590 mg of Amberlyst® 15 ion-exchange resin were added and the mixture was heated at reflux for 2 days, after which full conversion was confirmed by TLC (*n*-hexane/EtOAc 10:1). The mixture was cooled down to rt and the solid resin was removed by filtration. The crude product was repurified through a short silica gel column chromatography using *n*-hexane/EtOAc mixtures to afford 399 mg (76%) of BINOL **S18b** as a white solid.

^{1}H NMR (501 MHz, $CDCl_3$) δ 8.09 (s, 2H), 7.97 (d, *J* = 8.1 Hz, 2H), 7.79 – 7.73 (d, *J* = 8.4 Hz, 4H), 7.58 – 7.53 (d, *J* = 8.4 Hz, 4H), 7.44 (ddd, *J* = 8.1, 6.7, 1.3 Hz, 2H), 7.36 (ddd, *J* = 8.1, 6.7, 1.3 Hz, 2H), 7.31 – 7.25 (m, 2H), 5.45 (s, 2H), 2.19 (m, 6H), 2.05 (d, *J* = 2.9 Hz, 12H), 1.96 (d, *J* = 2.9 Hz, 2H), 1.93 – 1.71 (m, 10H).

^{13}C NMR (126 MHz, $CDCl_3$) δ 151.9, 151.1, 150.4, 147.5, 134.6, 133.0, 131.3, 130.7, 129.6, 129.4, 128.5, 127.3, 126.9, 125.2, 124.5, 124.3, 120.9, 112.6, 43.3, 43.2, 36.9, 36.7, 36.3, 29.1, 28.9.

HRMS (ESI) calculated for $C_{52}H_{49}O_2$ ([M–H$^-$]): 705.373805, found: 705.373803.

$[\boldsymbol{\alpha}]_{\boldsymbol{T}}^{\mathbf{20}}$ = -4.55 (*c* = 0.13, $CHCl_3$).

7.2. Synthesis of (*S*,*S*)-IDPis

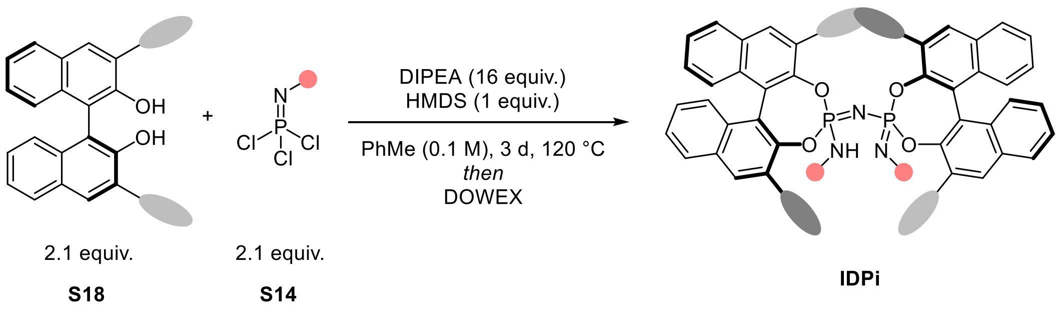

(*S*,*S*)-(4-*tert*-Butylphenyl)-C_6F_5 IDPi (6b)

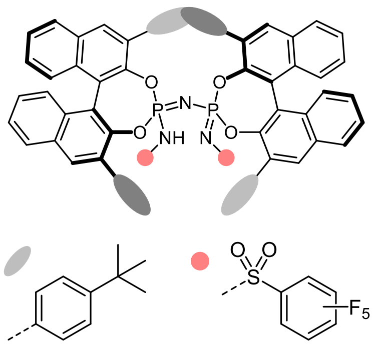

In a flame-dried Schlenk flask under argon, 3,3'-bis(4-(tert-butyl)phenyl)-[1,1'-binaphthalene]-2,2'-diol (**S18a**, 1.00 g, 1.81 mmol, 2.1 equiv.) and ((perfluorophenyl)sulfonyl)phosphorimidoyl trichloride **S14** (694 mg, 1.81 mmol, 2.1 equiv.) were dissolved in toluene (8.2 mL), then diisopropylethylamine (2.4 mL, 13.8 mmol, 16.0 equiv.) was added and the yellow suspension was stirred at rt for 10 min. Hexamethyldisilazane (180 µL, 0.86 mmol, 1.0 equiv.) was added to the reaction mixture, which was stirred at rt for 10 min, then heated to reflux for 3 d. After cooling to rt, the mixture was diluted with DCM and quenched with aq. HCl 10%. The aqueous layer was extracted with DCM (3x30 mL) and the combined organic layers were dried over anhydrous Na_2SO_4 and concentrated under reduced pressure. Purification by silica gel column chromatography (*n*-hexane/EtOAc 9:1 to 4:1) afforded a white solid, which was subjected to acidification by filtration over a plug of DOWEX 50WX8 (H-form, eluted with DCM) to afford catalyst **6b** (936 mg, 65%) as a yellowish solid. Spectroscopic data was consistent with the values reported in the literature.[24]

^{1}H NMR (501 MHz, $CDCl_3$) δ 8.18 (d, *J* = 8.2 Hz, 2H), 8.05 (s, 2H), 8.00 – 7.92 (m, 4H), 7.73 – 7.66 (m, 2H), 7.57 – 7.46 (m, 5H), 7.40 – 7.30 (m, 5H), 6.82 (d, *J* = 8.0 Hz, 4H), 6.52 (d, *J* = 8.1 Hz, 4H), 1.30 (s, 18H), 0.89 (s, 18H).

^{13}C NMR (126 MHz, $CDCl_3$) δ 151.3, 150.3, 143.8 (dm, *J* = 260 Hz), 143.6 (dt, *J* = 9.4, 5.3 Hz), 142.8 (dm, *J* = 260 Hz), 136.9 (dm, *J* = 257 Hz), 134.5, 133.2, 132.8, 132.5, 132.1, 131.7 (d, *J* = 6.3 Hz), 131.2 (d, *J* = 6.7 Hz), 130.4, 129.4, 129.0, 128.9, 128.3, 127.4, 126.9 (d, *J* = 6.7 Hz), 126.8 – 126.6 (m), 126.1, 124.7, 123.3, 122.7, 117.5 (m), 34.7, 34.4, 31.2.

^{19}F NMR (471 MHz, $CDCl_3$) δ -134.85 (d, *J* = 22.2 Hz, 4F), -145.89 (t, *J* = 21.7 Hz, 2F), -160.15 (dd, *J* = 22.3, 17.0 Hz, 4F).

^{31}P NMR (203 MHz, $CDCl_3$) δ -8.58.

HRMS (ESI) calculated for $C_{92}H_{72}F_{10}N_3O_8P_2S_2F_{10}$ ([M–H$^-$]): 1662.40819, found: 1662.40802.

$[\alpha]_T^{20}$ = +135.48 (*c* = 0.12, $CHCl_3$).

LC-MS (50 mm Zorbax SB300-C8, 3.5 µm, 4.6 mm i.d., 1% TFA/MeCN 25:75, 1.0 mL/min, 4.7 MPa, 308 K, 254 nm): t_R = 16.8 min (98% purity).

(*S*,*S*)-(4-(3*r*,5*r*,7*r*)-adamantan-1-yl)phenyl)-C_6F_5 IDPi (7b)

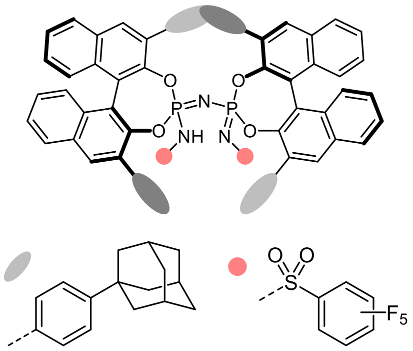

In a flame-dried Schlenk flask under argon, BINOL **S17b** (240 mg, 0.34 mmol, 2.1 equiv.) and ((perfluorophenyl)sulfonyl)phosphorimidoyl trichloride **S13** (130 mg, 0.34 mmol, 2.1 equiv.) were dissolved in toluene (1.5 mL), then diisopropylethylamine (450 µL, 2.59 mmol, 16.0 equiv.) was added and the yellow suspension was stirred at rt for 10 min. Hexamethyldisilazane (34 µL, 0.16 mmol, 1.0 equiv.) was added to the reaction mixture, which was stirred at rt for 10 min, then heated to reflux for 4 d. After cooling to rt, the mixture was diluted with DCM and quenched with aq. HCl 10%. The aqueous layer was extracted with DCM (3x10 mL) and the combined organic layers were dried over anhydrous Na_2SO_4 and concentrated under reduced pressure. Purification by silica gel column chromatography (*n*-hexane/EtOAc 9:1 to 4:1) afforded a white

solid, which was subjected to acidification by filtration over a plug of DOWEX 50WX8 (H-form, eluted with DCM) to afford catalyst **7b** (47 mg, 15%) as a yellowish solid.

^{1}H NMR (501 MHz, $CDCl_3$) δ 8.14 (d, *J* = 8.2 Hz, 2H), 8.03 (s, 2H), 7.94 (d, *J* = 8.3 Hz, 2H), 7.87 (s, 2H), 7.69 (t, *J* = 7.5 Hz, 2H), 7.54 (ddd, *J* = 8.1, 5.5, 2.5 Hz, 2H), 7.49 – 7.43 (m, 6H), 7.40 (d, *J* = 8.4 Hz, 4H), 7.35 – 7.29 (m, 4H), 6.81 (d, *J* = 8.2 Hz, 4H), 6.48 (d, *J* = 8.1 Hz, 4H), 5.07 (bs, 1H), 2.05 – 1.97 (m, 6H), 1.87 (m, 12H), 1.76 – 1.66 (m, 18H), 1.57 – 1.45 (m, 18H), 1.42 – 1.35 (m, 6H).

^{13}C NMR (126 MHz, $CDCl_3$) δ 151.3, 150.6, 134.5, 133.6, 132.8, 132.4, 132.2, 131.7 (d, *J* = 5.8 Hz), 131.2, 130.4, 129.5, 128.9 (d, *J* = 15.2 Hz), 128.3, 127.5, 127.0 – 126.3 (m), 125.5, 124.2, 123.3, 122.5, 42.8, 42.7 36.9, 36.7, 36.2, 35.9, 29.1, 28.9 (other signals not detected or observed).

^{19}F NMR (471 MHz, CDCl3) δ -134.74 (t, *J* = 22.3 Hz, 4F), -146.11 (t, *J* = 22.3 Hz, 2F), -160.14 (t, *J* = 20.3 Hz, 4F).

^{31}P NMR (203 MHz, $CDCl_3$) δ -8.14.

HRMS (ESI) calculated for $C_{116}H_{96}F_{10}N_3O_8P_2S_2$ ([M–H^{-}]): 1974.596000, found: 1974.59597.

$[\boldsymbol{a}]_{\boldsymbol{T}}^{\boldsymbol{20}}$ = +144.52 (*c* = 0.11, $CHCl_3$).

LC-MS (50 mm Zorbax SB300-C8, 3.5 µm, 4.6 mm i.d., 1% TFA/MeCN 10:90, 1.0 mL/min, 4.7 MPa, 308 K, 254 nm): t_R = 3.57 min (99% purity).

8. Mechanistic Investigations

8.1. On the Stereospecificity: Reactions with D-Labeled Substrates

Two possible mechanisms for the IDPi-catalyzed cycloaddition can be considered: a stepwise reaction pathway (involving the intermediacy of a benzyl cation) or a concerted-asynchronous pathway (Figure 3).[8, 25]

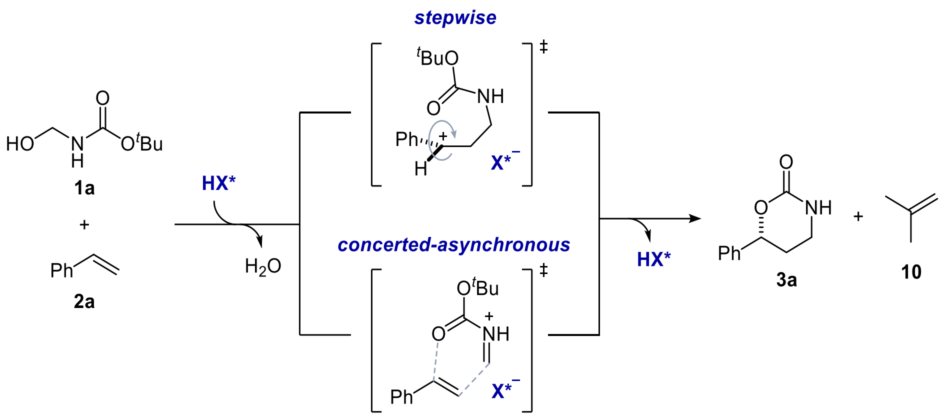

Figure SI-3. Possible reaction pathways of the hetero-[4+2] cycloaddition.

The stereospecific nature of the reaction was established by employing β-deuterium-labeled styrenes (***cis*-2a-β-d₁** and ***trans*-2a-β-d₁**, respectively) as substrates. Analysis of the ^{1}H NMR spectra of the reaction crude under optimized reaction conditions (see Figure SI-4) revealed that the stereochemistry of the starting material olefin was translated into the cycloadduct **3a-d₁**. These results are in line with a more concerted-asynchronous pathway, where such benzylic freely rotating species is either not formed or, if it is formed, is rather short-lived.

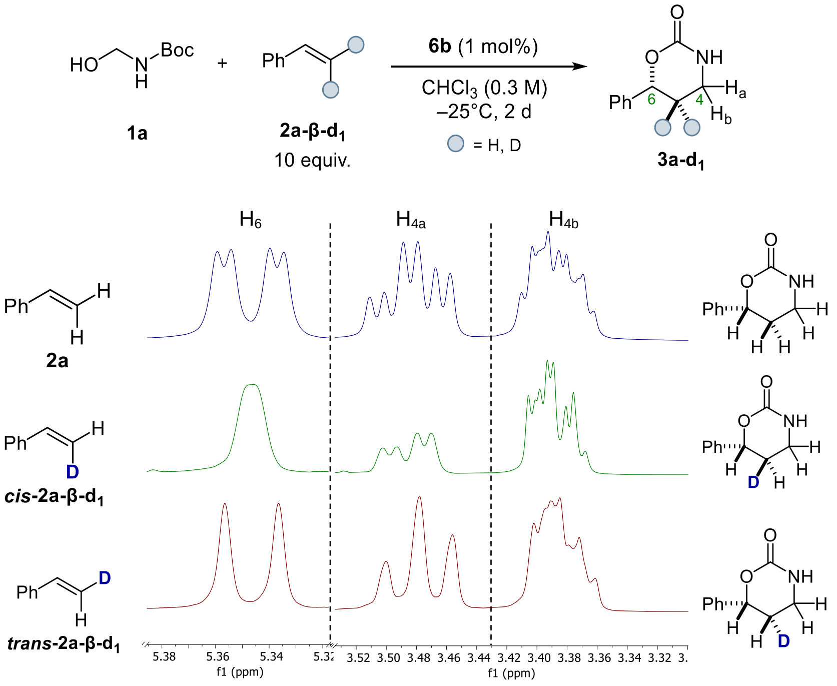

Figure SI-4. Comparison of ^{1}H NMR of the IDPi-catalyzed cycloaddition reaction of β-deuterostyrenes (**2a-β-d₁**).

8.2. ^{18}O-Labeling Reaction: Isotope Shift Effect

As summarized in Figure SI-5, subsequent to the activation of the electrophile **1a**, two different transition state conformations between styrene and iminium ion **I** can be envisioned, referred to as TS1-a and TS1-b.

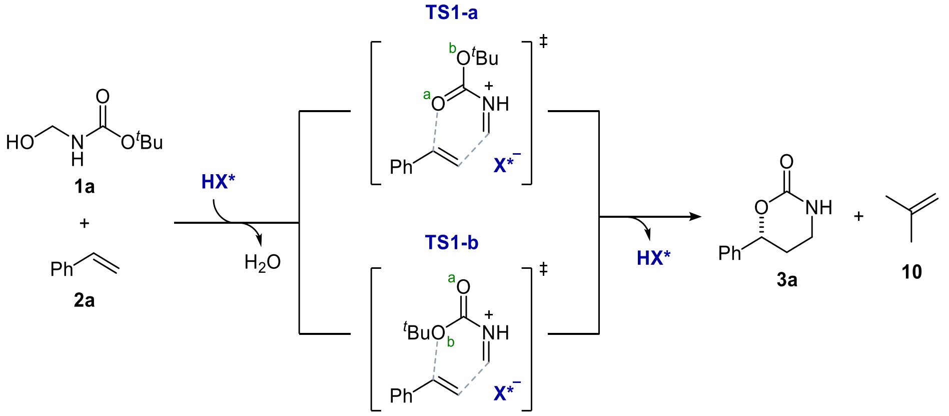

Figure SI-5. Possible conformations of the iminium ion **I** during the cycloaddition reaction.

In order to gain further insights into the reaction mechanism and to rationalize the geometry of the transition state of the [4+2] hetero-cycloaddition reaction, selective ^{18}O-labeling of the alkoxy oxygen of substrate **1a** was implemented. In this way, it is possible to differentiate the carbonyl oxygen (O_a) from the alkoxy oxygen (O_b) of the substrate without affecting its inherent reactivity.

Isotopic substitution frequently causes a measurable change in the NMR chemical shift of neighboring nuclei. The detection of such shifts in ^{13}C NMR spectra permits direct observation of the position and extent of ^{18}O-labeling in the molecule.[26] Figure SI-6 shows the ^{13}C NMR spectrum of the substrate **1a-^{18}O** at –20 °C: two different NMR signals can be observed for C_2 and C_3. The one with a higher chemical shift value can be assigned to the ^{16}O-^{13}C isotopomer and the other one to the ^{18}O-^{13}C derivative.[I]

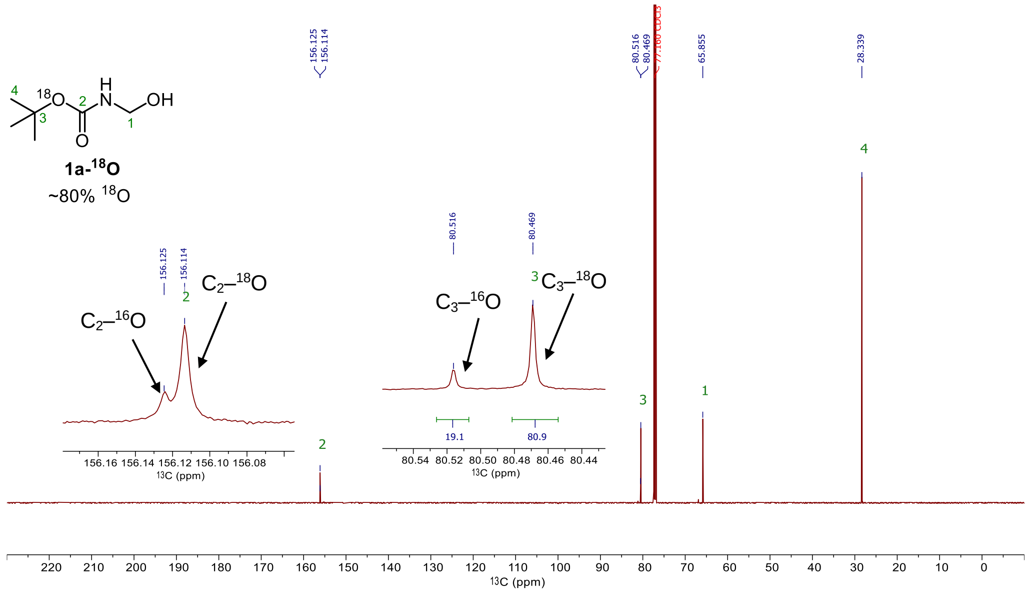

Figure SI-6. ^{13}C NMR analysis of substrate **1a-^{18}O** at 253 K.

[I] Enrichment could not be assigned at 25 °C due to line broadening caused by rotamer exchange of the ^{13}C signals of interest.

Subsequently, ^{18}O-enriched substrate **1a-^{18}O** was submitted under optimized reaction conditions using IDPi **6b**, which furnished product **3a-^{18}O** in 60% yield. Analysis of ^{13}C NMR spectrum at 25 °C indicated that the product is approximately 80% ^{18}O-enriched at the carbonyl oxygen (Figure SI-7). An isotope shift at position C_4 was not observed. The absence of oxygen isotope scrambling at positions C_1 and C_4 suggests that the nucleophilic attack of the olefin on iminium ion **I** occurs exclusively through the TS1-a conformation.

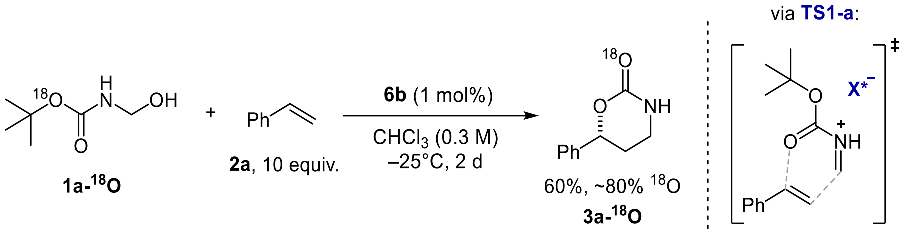

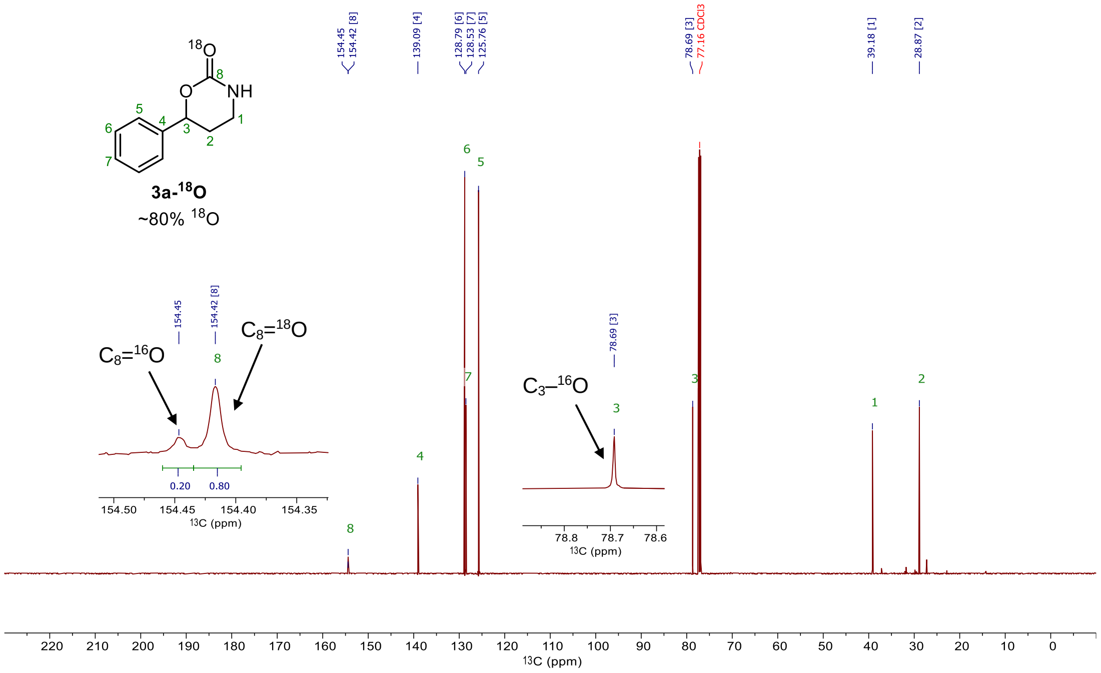

Figure SI-7. ^{13}C NMR analysis of product **3a-^{18}O** at 298 K.

8.3. NMR Kinetic Studies

General procedure for sample preparation and data processing with MNOVA

An oven-dried (80 °C, overnight) NMR tube was charged with substrate **1a** (23 mg, 1 equiv.) and styrene (**2a**, 180 µL, 10 equiv.) in 0.38 mL $CDCl_3$. The initial mixture was precooled to –78 °C in a dry ice/ethanol bath. After addition of a solution of catalyst **6b** (1 mol%) in $CDCl_3$ (125 µL), the NMR tube was quickly turned upside down, vortexed, and transferred to the precooled NMR probe at 248 K (–25 °C). After quick shimming, single-scan ^{1}H and ^{31}P NMR spectra were acquired every 5 min until completion of the reaction (24–48 h). The acquired NMR data was imported with the Reaction Monitoring Plugin into MNOVA 14.3.2 and processed therein (baseline correction, phase correction, integration).

8.3.1. Reaction Monitoring

The following Figure SI-8 shows ^{1}H NMR spectra taken at different time points during the reaction at –25 °C in presence of IDPi **6b**. This experiment enabled the detection of isobutene (**10**) together with various reaction side products, including *tert*-butanol (**11**), 6,6-dimethyl-1,3-oxazinan-2-one (**12**), and dimeric electrophile **13**.

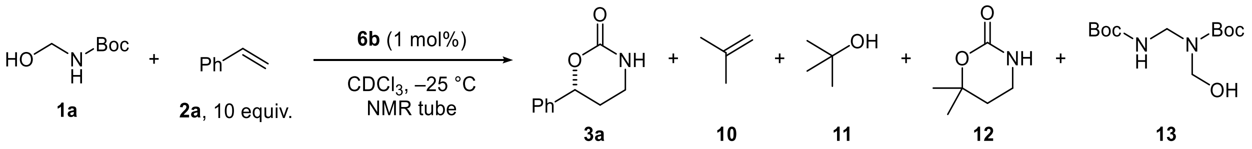

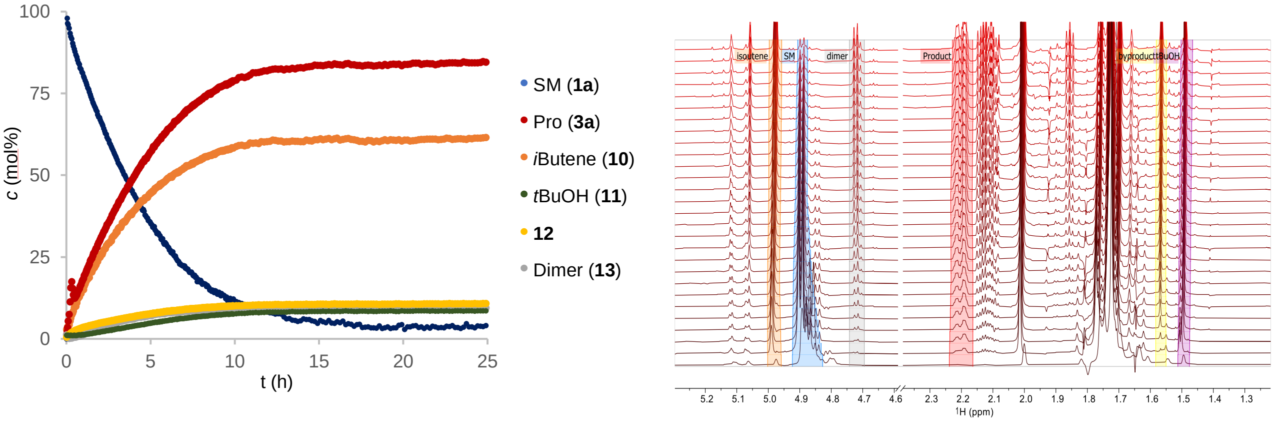

Figure SI-8. Left: Concentration plots obtained from ^{1}H NMR reaction monitoring during the reaction of **1a** and styrene (**2a**) with 1 mol% of catalyst **6b** at 248 K. Right: ^{1}H NMR spectra recorded at different time points (every 30 min from t = 0).

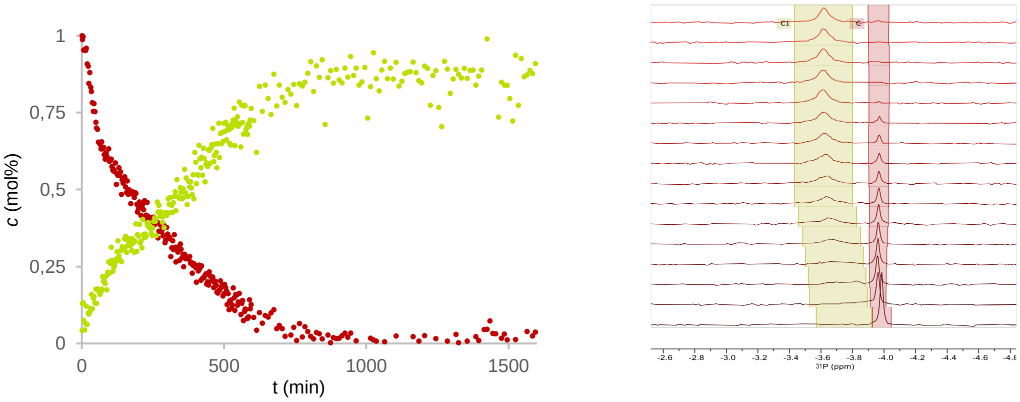

Figure SI-9. Left: Concentration plots obtained from the integration of ^{31}P NMR signals. Right: ^{31}P NMR spectra recorded at different time points (every 30 min from t = 0).

As depicted in Figure SI-9, the substrate **1a** conversion rate drops non-linearly during the reaction. Interestingly, analysis of the ^{31}P NMR profile revealed two distinct species. This observation triggered us to uncover the different catalytic species present in the reaction.

8.3.2. Interaction of Substrates and Catalyst 6b

Sample Preparation

An oven-dried (80 °C, overnight) NMR tube was charged with catalyst **6b** (2.6 mg, 1 mol%) and styrene (**2a**, 180 µL, 10 equiv.) in 0.38 mL $CDCl_3$. After the measurement of a ^{31}P NMR, a solution of substrate (1 equiv.) in $CDCl_3$ (125 µL) was added, the NMR tube was quickly turned upside down, vortexed, and transferred to the precooled NMR probe at 248 K (–25 °C). After quick shimming, single-scan ^{1}H and ^{31}P NMR spectra were acquired every 5 min until completion of the reaction.

Interaction with 2a

To study the nature of the different catalytic species, we first measured the ^{1}H and ^{31}P NMR of catalyst **6b** in presence of styrene (**2a**) and solvent. It is worth mentioning that due to the excess of styrene present in the reaction this reactant should also be considered as a co-solvent in the reaction system. The catalyst ^{31}P peak appears as a broad singlet at –4.1 ppm (Figure SI-10a).

Interaction with 1a and 2a

Next, one equivalent of substrate **1a** in $CDCl_3$ solution was transferred to the aforementioned mixture at 248 K. Subsequent analysis revealed that addition of the substrate **1a** to the previous mixture shifted the ^{31}P NMR signal by approximately 0.2 ppm toward higher frequencies, together with a change on the signal shape (sharp peak at –3.9 ppm, Figure SI-10b). It has been previously shown by our group that the IDPi catalyst in an ion pair can form such sharp signals due to the high symmetry in its anionic form.[27] Upon the progression of the reaction, the sharp peak decays and a second ^{31}P signal arises below and shifts toward higher frequencies upon advancement of the reaction (–3.5 ppm at the end of the reaction, Figure SI-10c to f). This broad peak can be assigned to the acidic form of the IDPi catalyst, which interacts weakly with other components in the reaction mixture. The chemical shift can be attributed to changes in the composition of the reaction mixture.

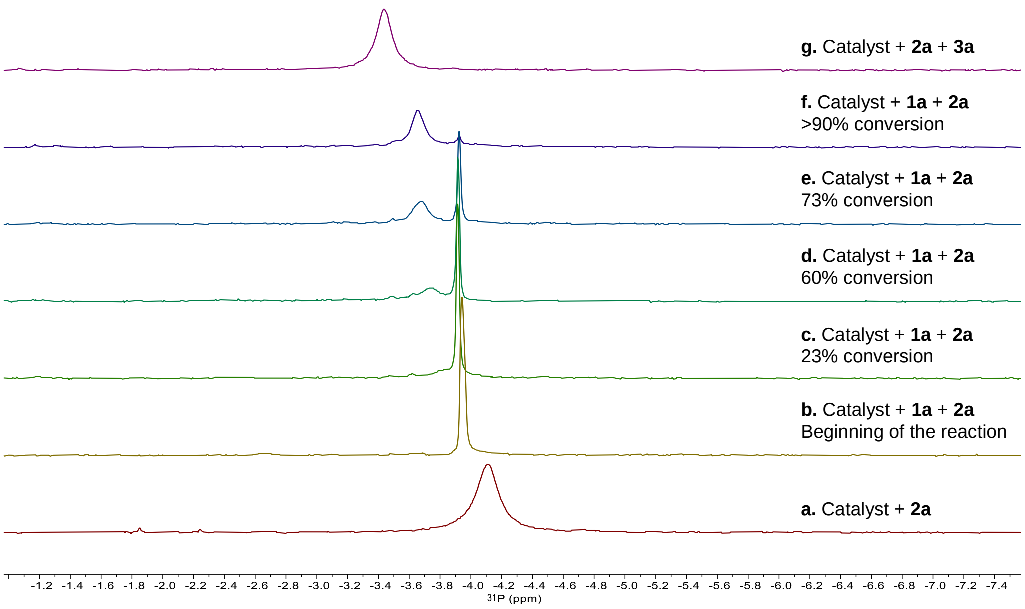

Figure SI-10. ^{31}P NMR catalyst **6b** signals during the course of the reaction.

Intrigued by the finding of the stable ionic intermediate, we carefully analyzed the ^{1}H NMR spectra in the initial minutes of the reaction. The appearance of a characteristic peak at 10.1 ppm that decayed overtime led us to hypothesize the detection of an iminium ion intermediate on the NMR time scale (Figure SI-11). To our surprise, additional experiments with higher catalyst loadings and lower temperatures revealed the formation of ionic intermediate **II**, which was assigned by advanced 1D and 2D NMR methods and HRMS (see section 7.3.3.). According to the ^{1}H and ^{31}P NMR data, the continual transformation of intermediate **II** successfully regenerated catalyst **6b** and produced the desired product **3a**.

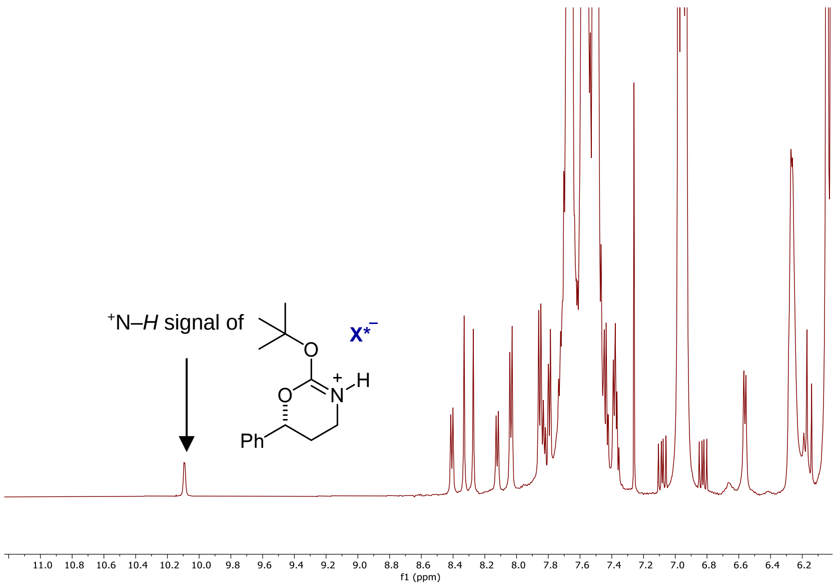

Figure SI-11. Identification of reaction ion pair intermediate **II** by ^{1}H NMR at the beginning of the reaction.

No direct evidence for the formation of the iminium ion intermediate **I**, which was long-lived enough to be spectroscopically characterized by NMR, could be collected. However, it is plausible to hypothesize that a rapid conversion of the short-lived cationic intermediate **I** leads to the formation of product **3a**. Moreover, competing off-pathway side reactions of ion pair **I** with isobutene (**10**) or starting material **1a** would form the abovementioned side products **12** and **13**, respectively (Figure SI-12). Due to the presence of various nucleophilic species in solution that can attack the highly reactive iminium ion **I**, an excess of styrene is required to favor the desired transformation (see Optimization Table on Section 3). These observations let us to conclude that the product formation from the ionic intermediate **II** via generation of isobutene and *tert*-butanol are turnover-limiting steps of the reaction. The release of the catalyst from the stable ionic form enables the restart of the catalytic cycle.

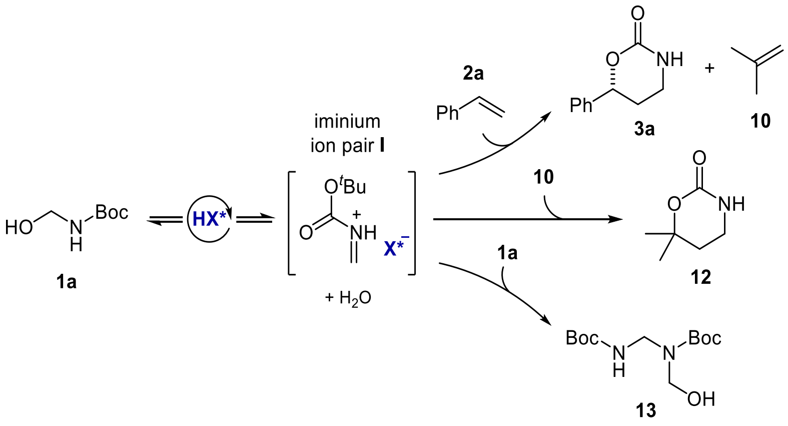

Figure SI-12. Possible reaction counterparts of iminium ion pair **I** in the reaction system.

In another aspect, four different pathways for the formation of *tert*-butanol are conceivable (Figure SI-13): through H_2O attacking on C_8 of intermediate **II**, H_2O attacking C_9 of the same intermediate, or via H_2O attack to *tert*-butyl cation (which is formed through different routes). Subsequently, the viability of these options will be discussed.

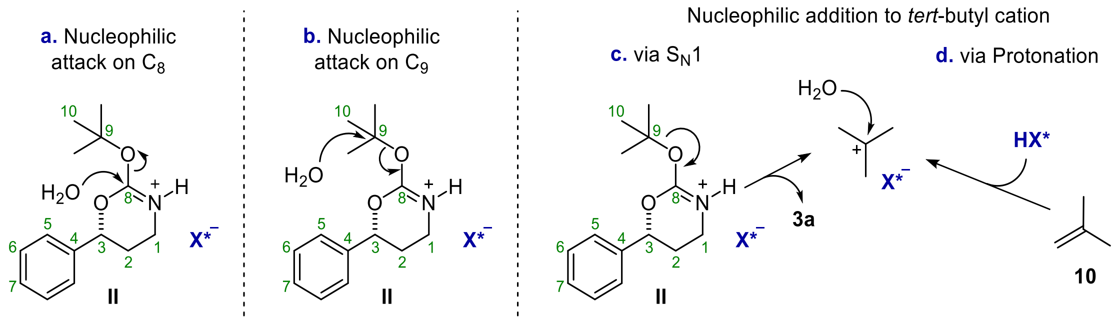

Figure SI-13. Potential pathways for the formation of *tert*-butanol (**11**).

Since an isotopic scrambling was not observed at position C_8 in the previous experiment with ^{18}O-labeled substrate (see section 7.2.), the first option a. can be disregarded.

Option b. would involve the S_N2'-like reaction of water with intermediate **II**, to yield product **3a**. The ^{13}C NMR chemical shift of C_9 is notably deshielded for a typical *tert*-butyl group (see section 7.3.3 for further details), suggesting a partial positive charge on the quaternary carbon, thereby enhancing its leaving group capability.

Options c. and d. entail the nucleophilic addition over the *tert*-butyl cation which was formed upon cleavage of the reaction product from ion pair **II** (c.) or via protonation of isobutene by the IDPi catalyst (d.).

The latter option d. also seems unfeasible: ^{1}H NMR monitoring reveals no *tert*-butanol formation upon completion of the reaction. As shown in the previous section (see Section 7.3.1., Figure SI-8), *tert*-butanol production reaches a plateau after the starting material **1a** is consumed (t = 13 h) whilst isobutene, water, and catalyst are still present in the solution.

In conclusion, options b. and c. appear to be the most feasible under the reaction conditions, and their implications will be discussed in the following sections.

Interaction with 2a and 3a

In this independent experiment, the interaction between catalyst **6b** in presence of styrene and reaction product **3a** was studied. The catalyst ^{31}P peak appears as a broad singlet at –3.4 ppm (Figure SI-10g). No further signal shifting was observed. This experiment allowed us to assign the broad peak appearing at –3.8 ppm, which shifted toward higher frequencies in the previous experiment, to the catalyst weakly interacting with various reaction products and side products.

8.3.3. NMR Characterization of Ion Pair II

Sample preparation

An oven-dried (80 °C, overnight) NMR tube was charged with substrate **1a** (23 mg, 1 equiv.) and styrene (**2a**, 180 µL, 10 equiv.) in 0.38 mL $CDCl_3$. The initial mixture was precooled to –78 °C in a dry ice/ethanol bath. After addition of a solution of catalyst **6b** (10.4 mg, 4 mol%) in $CDCl_3$ (125 µL), the NMR tube was quickly turned upside down, vortexed, and transferred to the precooled NMR probe at 233 K (–40 °C). Afterwards a set of different NMR experiments was performed (see NMR data below) to characterize the intermediate as best as possible.

During the course of the reaction, a characteristic signal at 10.1 ppm was consistently observed. Initially, based on the comparable magnitude of the chemical shifts of iminium ion pairs reported by Mayr,[28] we presumed that the signal at 10.1 ppm corresponded to the $^{+}N{=}CH_2$ protons of the transient intermediate **I**. Conversely, 1D selective TOCSY spectrum obtained after selective excitation of the characteristic signal was in line with the above suggested reaction intermediate **II**.

The connectivity to the nitrogen was further confirmed by a ^{1}H–^{15}N HMBC optimized to a $^{1}J_{NH}$ coupling of 80 Hz where a signal at –273.9 ppm was observed ($^{1}J_{NH}$ ~ 94 Hz). The adjacent carbon chemical shifts of the assignable other protons were extracted from an edited HSQC and/or HMBC measurements. The signal of H_{10} was assigned based on the relative integral from the ^{1}H NMR and the cross peaks in the ^{13}C HMBC. The Table SI-4 shows all the chemical shifts extracted for this intermediate.

Table SI-4. Chemical shifts and cross peaks of intermediate **II** at 233 K.

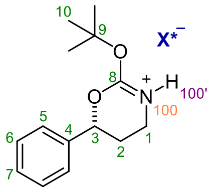

II

Atom	δ (ppm)	*J* (Hz)	COSY	HSQC	HMBC	ROESY
1 C	38.279			1', 1"		
H'	2.227		1", 100'	1		1", 100'
H"	4.338		1', 2'	1		1', 100'
2 C	27.949			2'		
H'	2.525		1", 2"	2		
H"	1.781		2', 3			
3 C	85.834			3		
H	6.178		2"	3		
4 C						
5 C						
H						
6 C						
H						
7 C						
H						
8 C						
9 C	91.55				10	
10 C	28.128			10	10	
H3	1.52			10	9, 10	100'
100 N	-273.91	94.00 (100')				
100' H	10.089	94.00 (100N)	1'			1', 1", 10

Interestingly, the ^{13}C chemical shift of C_9 is rather high (91.6 ppm) for a *tert*-butyl group (e.g. δ C_q(MTBE) = 72.8 ppm) and would be in line with a slight positive charge on the carbon which makes it a good leaving group (via H_2O attack to form *tert*-butanol or via deprotonation on H_{10} to eliminate isobutene).

All of the intermediate signals decay over time, as well as two characteristic peaks that can be assigned to the diastereotopic *tert*-butyl signals of the catalyst 3,3'-substituents. The ^{1}H NMR data is also in line with the two catalytic species that can be observed in the ^{31}P NMR during the reaction.

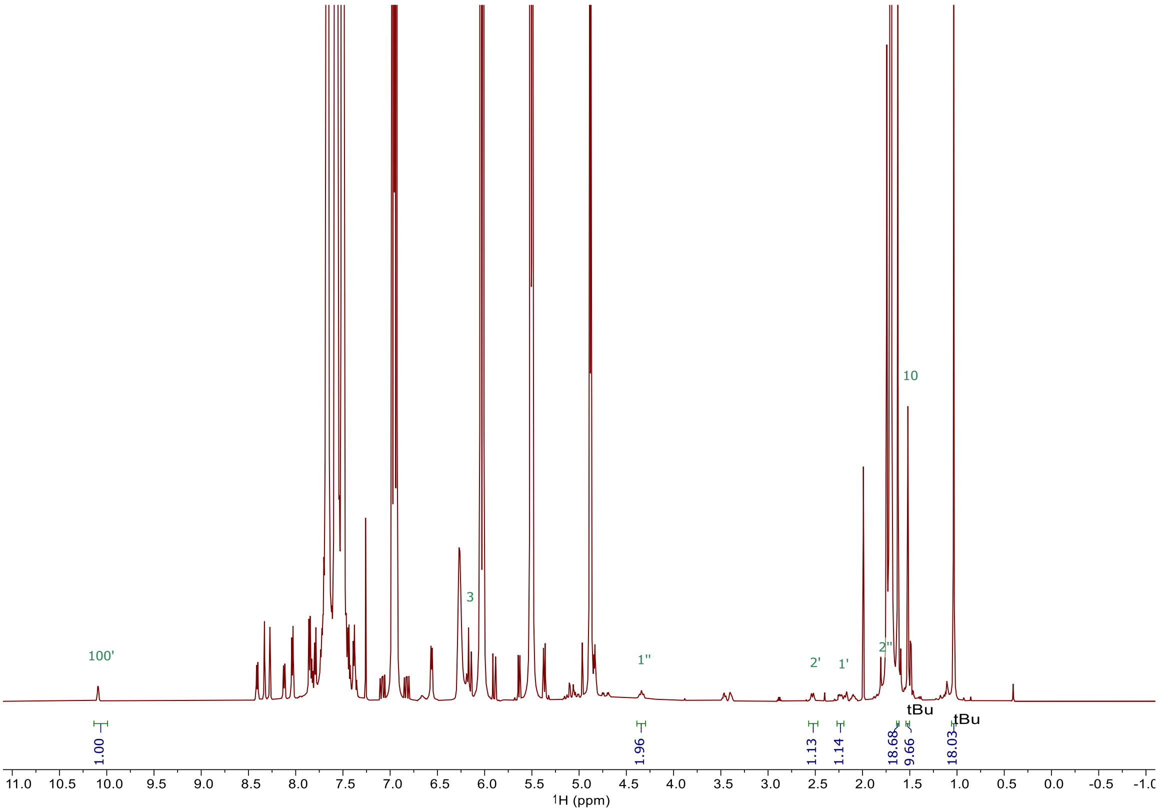

Figure SI-14. ^{1}H NMR spectrum of intermediate **II** (600 MHz, $CDCl_3$, 233 K).

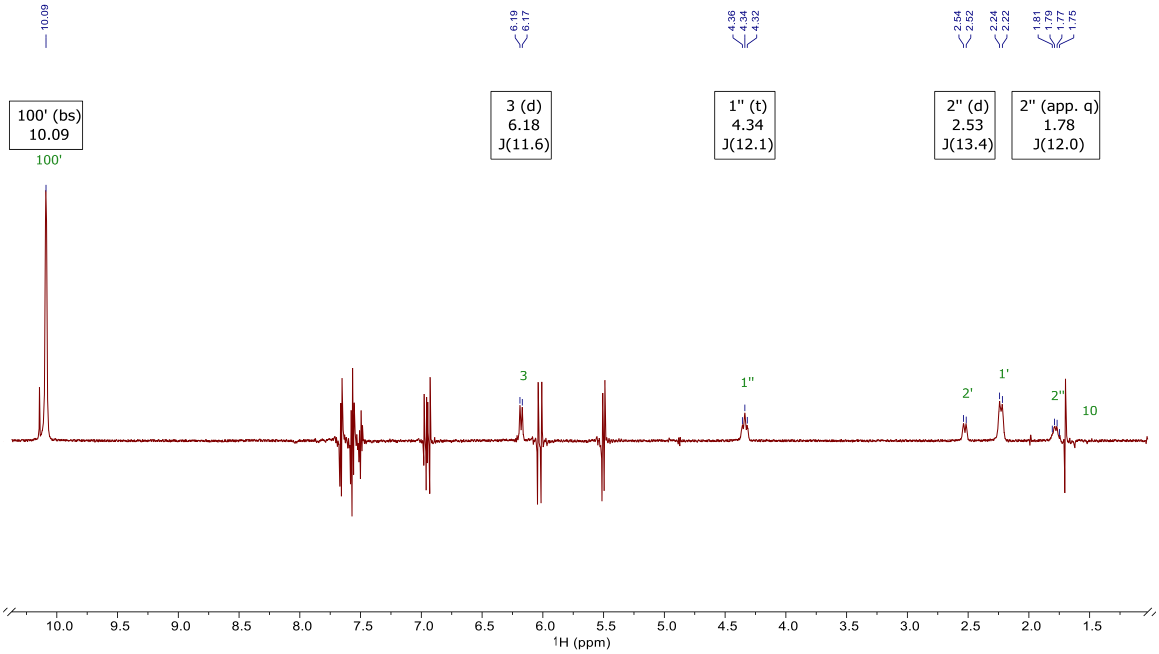

Figure SI-15. 1D selective TOCSY of intermediate **II** with excitation of H-100’ (spin lock mixing time 150 ms, 600 MHz, $CDCl_3$, 233 K).

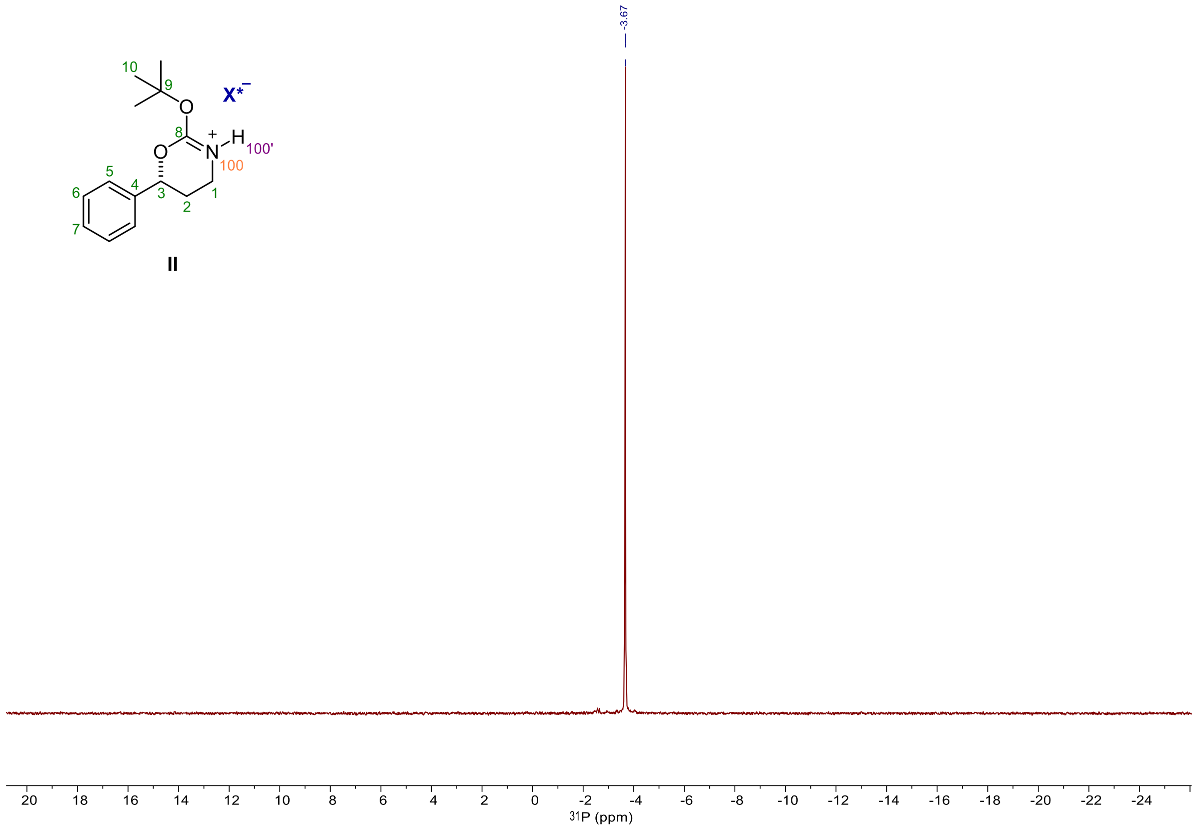

Figure SI-16. ^{31}P NMR spectrum of intermediate **II** (243 MHz, $CDCl_3$, 233 K).

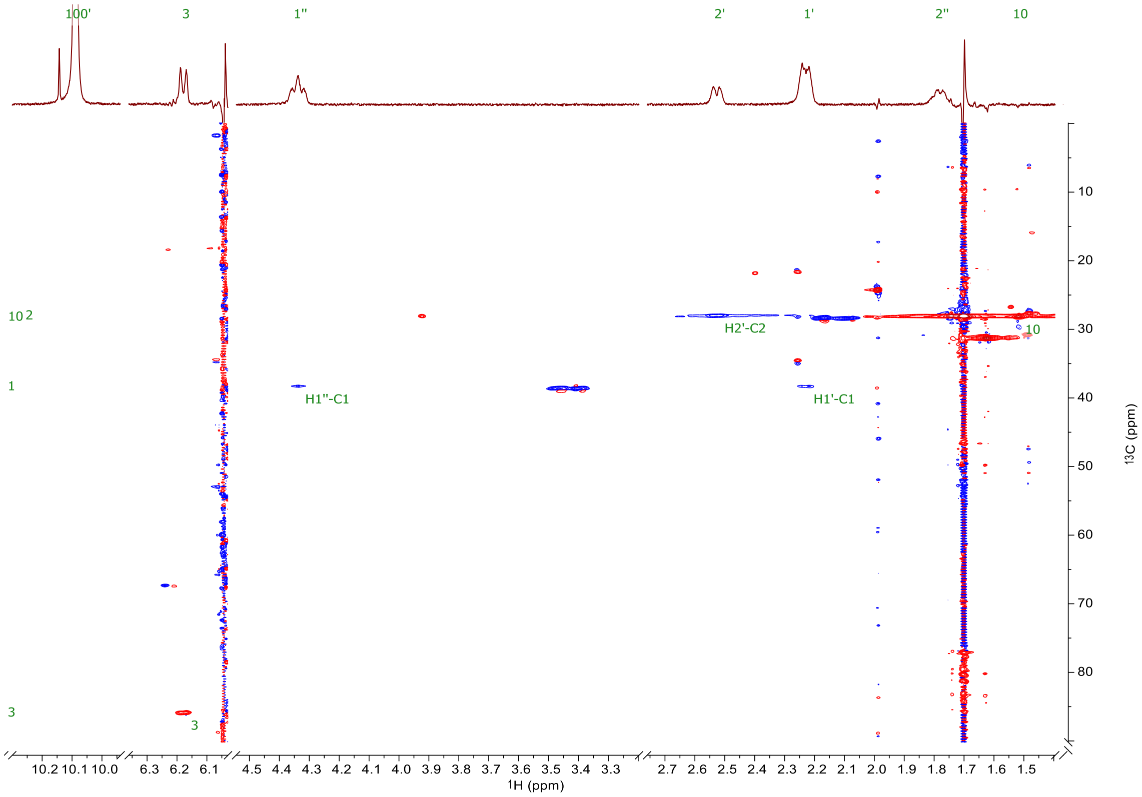

Figure SI-17. Excerpt of the ^{1}H-^{13}C-edited HSQC NMR spectrum showing relevant cross peaks of intermediate **II** (600 MHz, $CDCl_3$, 233 K).

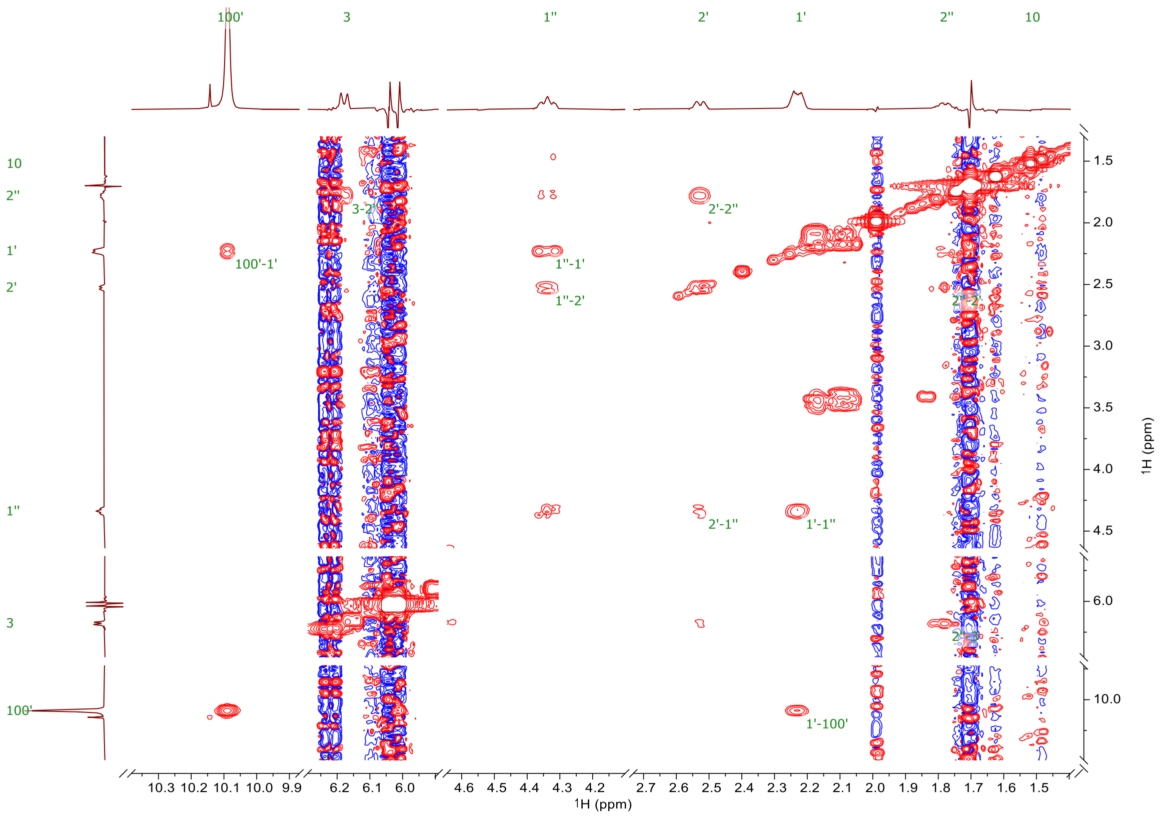

Figure SI-18. Excerpt of the ^{1}H,^{1}H-COSY NMR spectrum showing relevant correlations of intermediate **II** (600 MHz, $CDCl_3$, 233 K).

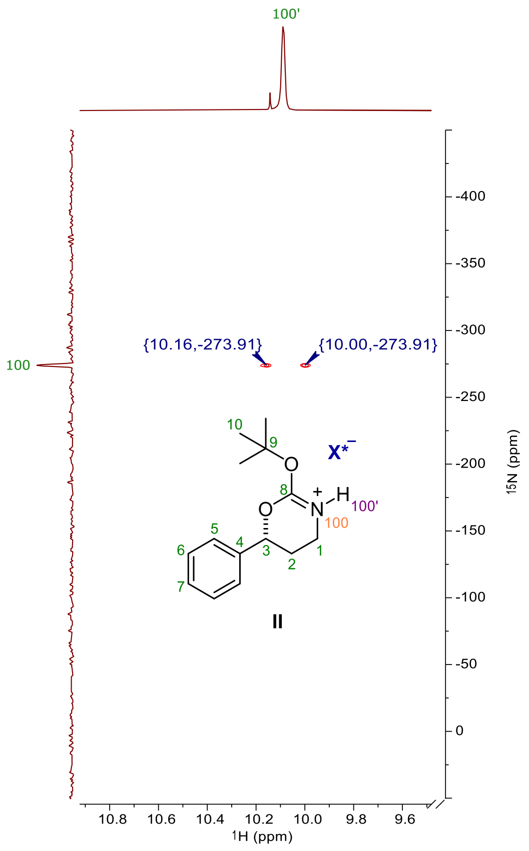

Figure SI-20. Cross section of the ^{1}H,^{15}N-HMBC spectrum showing the relevant cross peak of intermediate **II** (600 MHz, $CDCl_3$, 233 K).

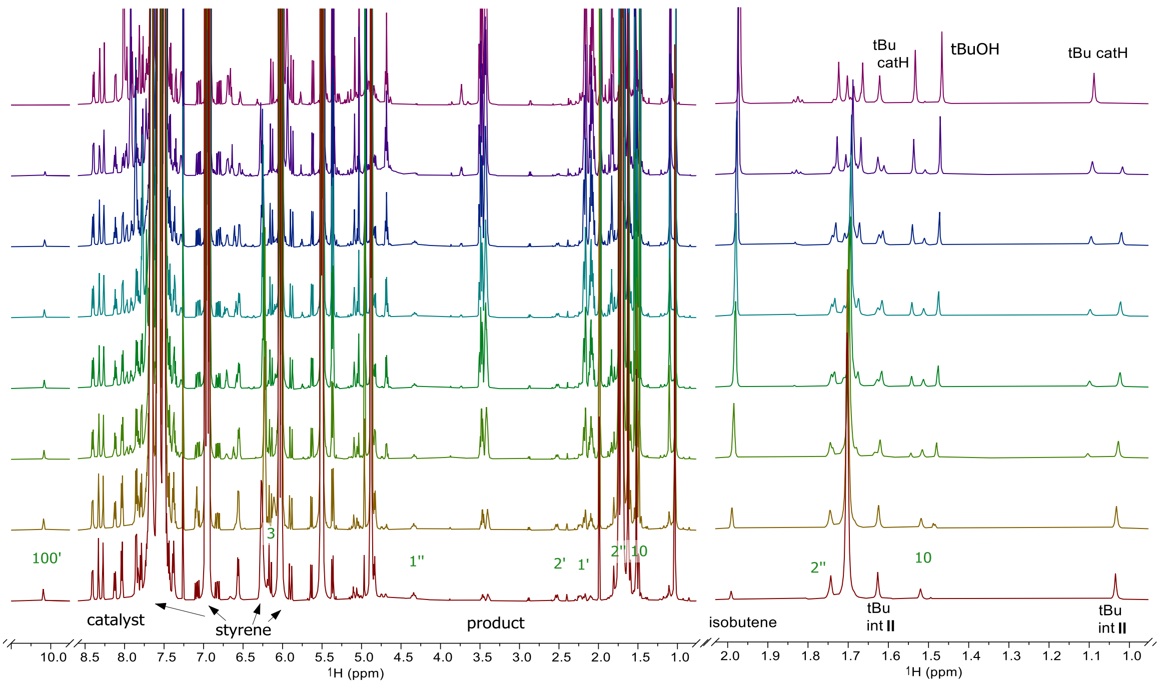

Figure SI-21. ^{1}H NMR spectra taken at different time points showing the decay of the intermediate **II** signals as well as the change of signals from the catalyst and product formation (600 MHz, $CDCl_3$, 233 K).

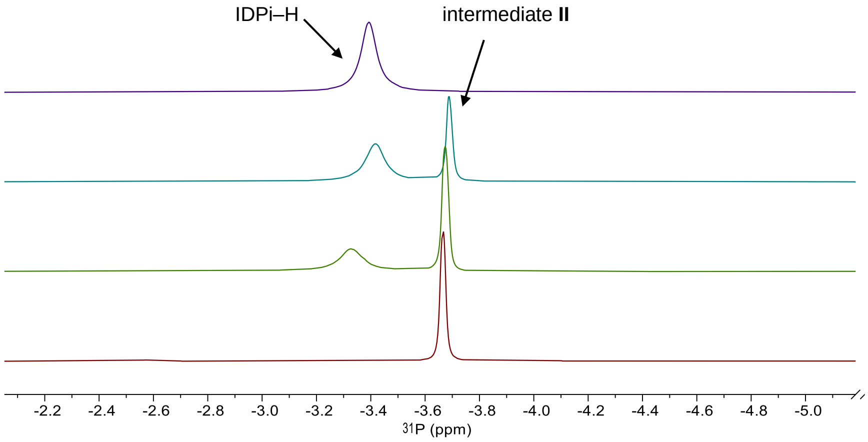

Figure SI-22. ^{31}P NMR spectra taken at different time points showing the decay of the intermediate **II** signals as well as the formation of a broadened IDPi–H species (243 MHz, $CDCl_3$, 233 K).

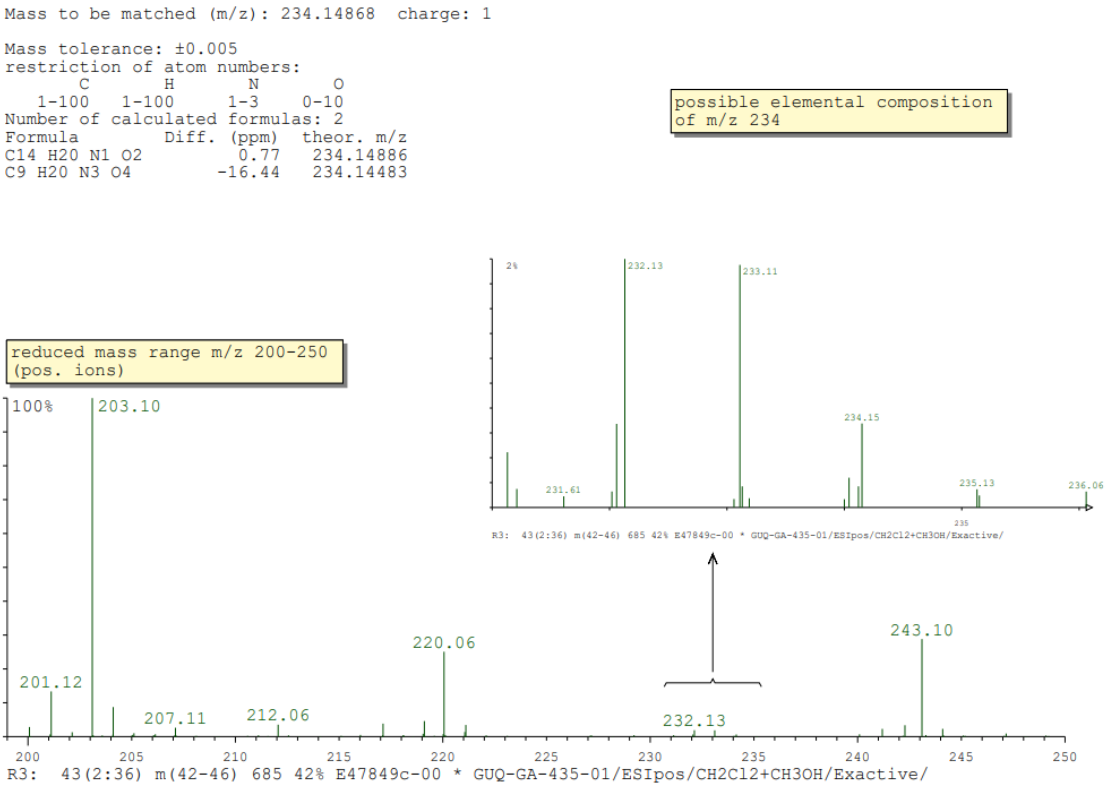

Figure SI-23. HRMS of the reaction intermediate **II**.

8.3.4. Determination of Catalyst Order

In order to determine the reaction order of the catalyst with Variable Time Normalization Analysis (VTNA),[29] the reaction of **1a** was monitored at four different concentrations of IDPi **6b** (0.52 mol%, 1.1 mol%, 2.0 mol%, and 3.5 mol%). The catalyst concentration was extracted as average from the ^{1}H NMR data over the entire reaction time. The Figure SI-24

shows an overview of different reaction profiles with time scales normalized to different catalyst orders. The best overlap was found when the reaction profiles are normalized to a 1.4 order dependence in catalyst concentration.

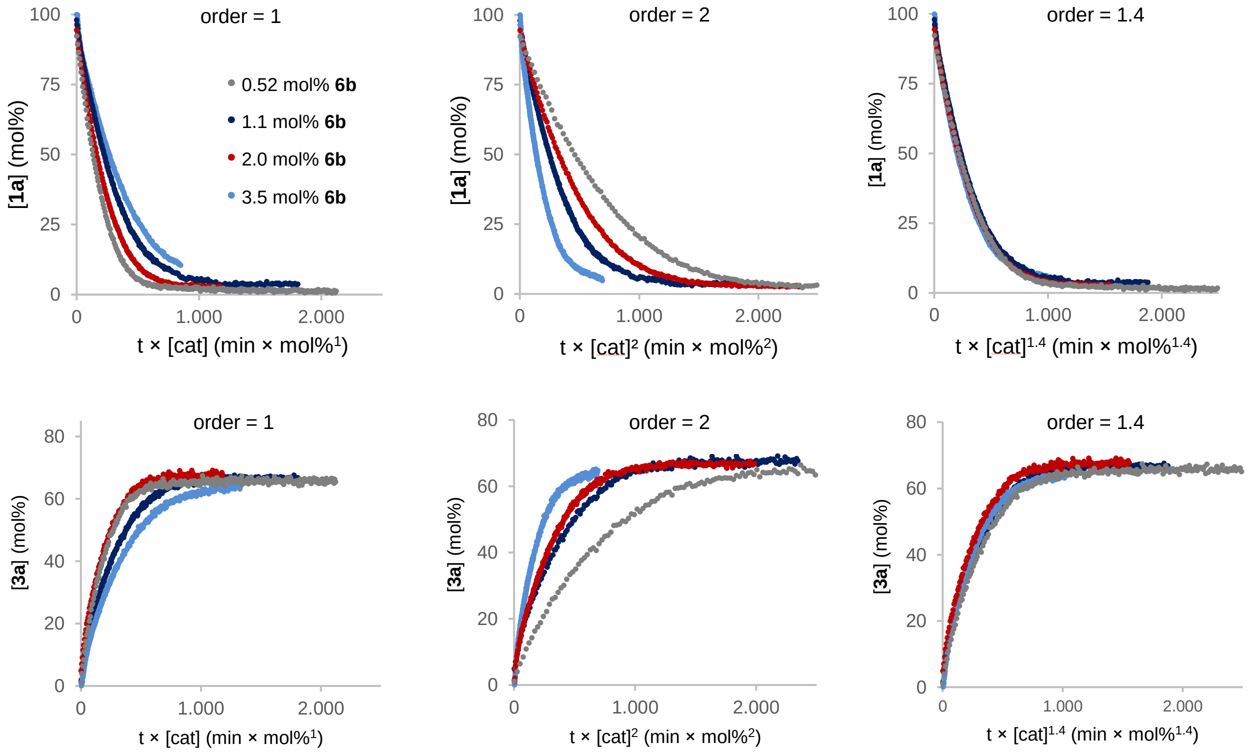

Figure SI-24. ^{1}H NMR concentrations profiles in $CDCl_3$ at 248 K in presence of 0.52 mol%, 1.1 mol%, 2.0 mol%, and 3.5 mol% **6b** of **1a** (first row), and **3a** (2nd row) with time scales normalized to a 1st (left), 2nd (middle), and 1.4th (right) order in catalyst concentration.

This result was unexpected and let us to hypothesize that due to the high stability of the ionic intermediate **II**, a second catalyst molecule could facilitate the isobutene elimination in the rate-limiting step and restore the catalytic cycle. Another plausible explanation for the observed catalyst order higher than one is the assistance of a water molecule found in the catalyst pocket, which would attack the electrophilic position of the *tert*-butyl of intermediate **II** (as depicted in Section 7.3.2., figure SI-13b), leading to the formation of *tert*-butanol and **3a**. A situation, where a byproduct from one step in the cycle serves as a reactant in a subsequent catalytic step has been discussed by Burés in the recent years and can result in catalysts orders higher than one although only one catalyst molecule is involved in the rate limiting step.[30] As two different byproducts (isobutene and *tert*-butanol) are formed, a scenario where a catalyst order between one and two is observed is therefore not unreasonable.

The following Figure SI-25 shows concentration profiles of intermediate **II** in the presence of different amount of catalyst **6b**. At high catalyst concentrations the intermediate decays rather quickly, whereas at low concentrations a steady state concentration seems reached. Additionally, two decay regimes seem to be present for higher catalyst concentrations.

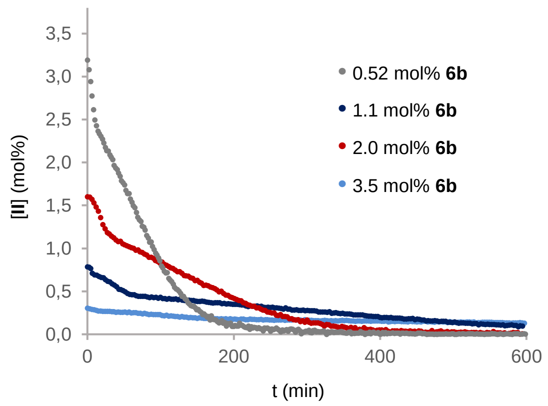

Figure SI-25. Concentration plots obtained from ^{1}H NMR reaction monitoring of intermediate **II** in presence of 0.52 mol%, 1.1 mol%, 2.0 mol%, and 3.5 mol% of IDPi **6b** during the course of the reaction

8.3.5. Nonlinear Effect Studies

Building on previous findings of a catalyst order greater than one, we hypothesized that the involvement of multiple catalyst molecules in the enantiodetermining step of the reaction could lead to nonlinear effects (NLE).

To verify this hypothesis, NLE experiments were set up according to the general procedure using scalemic mixtures of catalyst **6b**. Subsequently, the resulting enantiomeric excess of the product was measured.

As depicted in Figure SI-26, the enantioselectivity of product **3a** was found to exhibit a linear relationship with the enantiopurity of the catalyst used in the reaction, thereby ruling out the presence of NLE. This result suggests the presence of only one catalyst molecule on the enantiodetermining step. However, this result does not exclude the possibility of multiple catalyst molecules being involved in other turnover-limiting steps. Based on this observation and on the previous experiments, we can hypothesize that a second catalyst molecule could assist in the isobutene elimination of intermediate **II**. Since this step occurs subsequent to the enantiodetermining step, the presence of more than one catalyst does not affect the enantiomeric excess of the product.

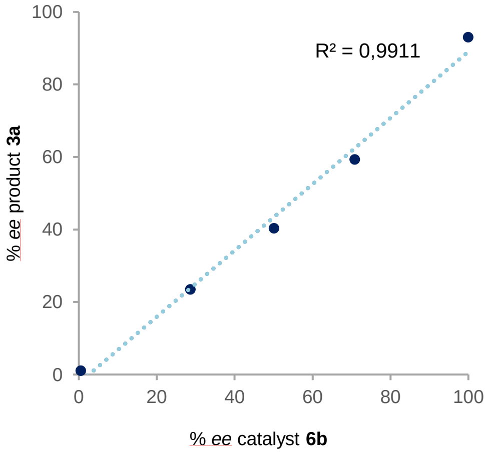

Figure SI-26. Plot of % *ee* of the product **3a** versus % *ee* of catalyst **6b**.

8.3.6. Experiments with Racemic Catalyst

To further investigate the unexpected results observed in our study, we examined the reaction rates of both racemic and enantiopure catalysts under identical conditions. As depicted in Figure SI-27, we observed an intriguing kinetic behavior where racemic form of the catalyst exhibited significantly higher reaction rates compared to their enantiopure counterparts: the reaction with *rac*-IDPi resulted in a rate enhancement by a factor of 1.4.

This suggests that the presence of heterochiral catalyst mixtures can lead to a faster decay of the reaction intermediate **II**, thereby accelerating the overall reaction. This result is consistent with the hypothesis positing the presence of more than one catalyst molecule assisting in the intermediate **II** consumption step.

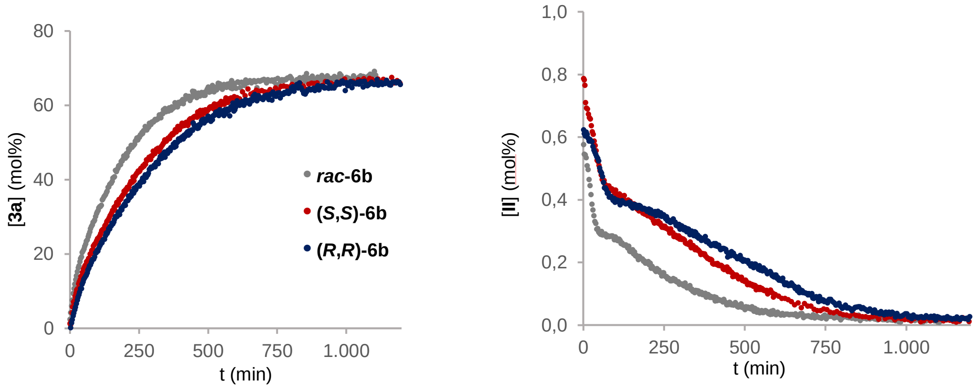

Figure SI-27. Concentration plots obtained from ^{1}H NMR reaction monitoring of **3a** (left) and **II** (right) when 1 mol% of racemic IDPi ***rac*-6b** is used (grey); reaction with 1 mol% of **(*S*,*S*)-6b** (red); reaction with 1 mol% of **(*R*,*R*)-6b** (blue).

The reaction order in catalyst was furthermore determined with Variable Time Normalization Analysis (VTNA).[29] The reaction of **1a** was monitored at four different concentrations of ***rac*-6b** (0.48 mol%, 1.0 mol%, and 2.1 mol%). The catalyst concentration was extracted as average from the ^{1}H NMR data over the entire reaction time. The Figure SI-28 shows an overview of different reaction profiles with time scales normalized to different catalyst orders. The best overlap was found when the reaction profiles are normalized to a 1.5 order dependence in catalyst concentration.

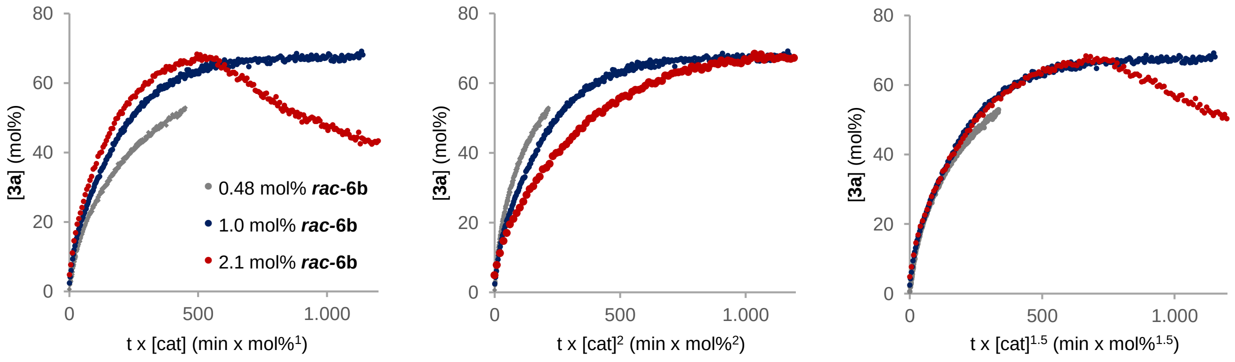

Figure SI-28. ^{1}H NMR concentrations profiles of **3a** in $CDCl_3$ at 248K in presence of 0.48 mol%, 1.0 mol%, and 2.1 mol% of ***rac*-6b** with time scales normalized to a 1st (left), 2nd (middle), and 1.5th (right) order in catalyst concentration.

8.3.7. Time Adjust and Inhibition Experiments

To examine the influence of the different side products on the substrate **1a** conversion rate, five independent experiments were conducted and compared against the standard conditions.

Sample Preparation for Time Adjust Experiment

An oven-dried (80 °C, overnight) NMR tube was charged with substrate **1a** (11.5 mg, 0.5 equiv.) and styrene (**2a**, 180 µL, 10 equiv.) in 0.38 mL $CDCl_3$. The initial mixture was precooled to –78°C in a dry ice/ethanol bath. After addition of a solution of catalyst **6b** (1 mol%) in $CDCl_3$ (125 µL), the NMR tube was quickly turned upside down, vortexed, and transferred to the precooled NMR probe at 248 K (–25 °C).

Sample Preparation for Product and Side Product Inhibition Experiments

Preparation of water solubilized in $CDCl_3$: a flame-dried Schlenk flask is charged with 10 mL of dry $CDCl_3$ (under Ar) and 16 µL of distilled water. The mixture is vigorously stirred at rt for 30 min. After this time, the water content present in the solution was determined by Karl Fischer titration (556 ppm of water).

Next, an oven-dried (80 °C, overnight) NMR tube was charged with substrate **1a** (23 mg, 1 equiv.), styrene (**2a**, 180 µL, 10 equiv.) in 0.38 mL of the previously prepared $CDCl_3$ solution. The initial mixture was precooled to –78°C in a dry ice/ethanol bath. After addition of a solution of catalyst **6b** (1 mol%) in $CDCl_3$ (125 µL), the NMR tube was quickly turned upside down, vortexed, and transferred to the precooled NMR probe at 248 K (–25 °C).

Sample Preparation for Product and Side Product Inhibition Experiments

An oven-dried (80 °C, overnight) NMR tube was charged with substrate **1a** (23 mg, 1 equiv.), styrene (**2a**, 180 µL, 10 equiv.), additive (equiv.) in 0.38 mL $CDCl_3$. The initial mixture was precooled to –78°C in a dry ice/ethanol bath. After addition of a solution of catalyst **6b** (1 mol%) in $CDCl_3$ (125 µL), the NMR tube was quickly turned upside down, vortexed, and transferred to the precooled NMR probe at 248 K (–25 °C).

In the first experiment the reaction was monitored at a lower initial **1a** concentration (0.6 M) and the time scale was shifted to match the same **1a** concentration of the standard condition following a procedure by Blackmond.[31] In absence of catalyst inhibition, an overlap between the standard and the shifted concentration profiles is expected. As seen in Figure SI-29, the "time-adjusted" profile does not overlay with that of reaction from the marked time point onward, suggesting that some product formed during the reaction is resulting in catalyst inhibition.

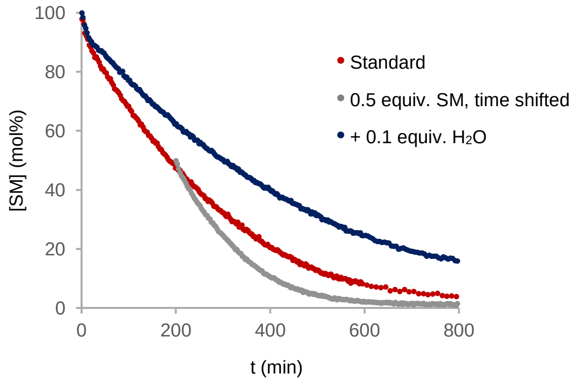

Figure SI-29. Concentration plots obtained from ^{1}H NMR reaction monitoring of **1a** under standard conditions (red); reaction at a lower concentration of **1a** where the time t_0 was shifted to match the same time point under standard conditions (grey); reaction with additional 0.1 equivalents of water (blue).

Intrigued by the role of water in our system, we studied the contribution of additional water in the reaction. The outcome depicted in Figure SI-29 indicates that introducing 0.1 equivalents of additional water reduces the reaction rate. This result was surprising since we expected that the addition of water could accelerate the consumption of intermediate **II** yielding *tert*-butanol and **3a**. However, as shown in Figure SI-30, the addition water is instead resulting in a decrease in both isobutene and *tert*-butanol production. These findings may suggest that simple addition of external water inhibits the overall reaction. However, it cannot be excluded that the water originating from the activation of substrate **1a**, which is likely enclosed in the catalyst pocket, could potentially react with intermediate **II** in the turnover-limiting step to yield **3a** and *tert*-butanol.

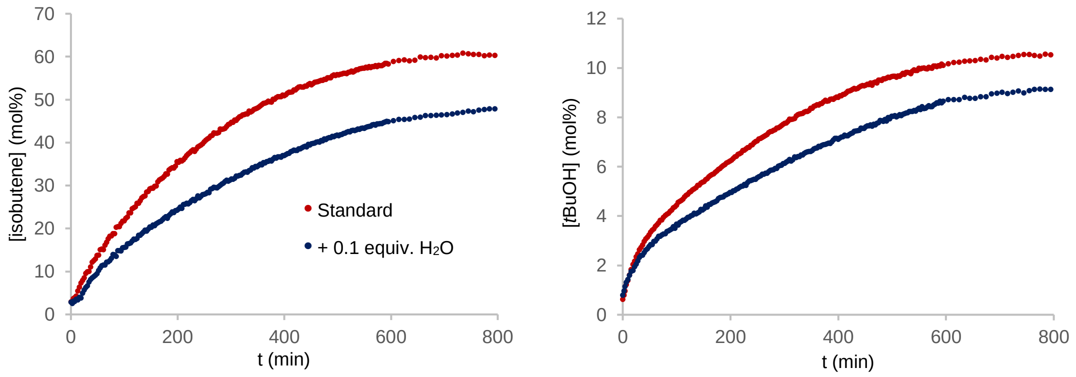

Figure SI-30. Concentration plots obtained from ^{1}H NMR reaction monitoring of isobutene (left) or *tert*-butanol (right). Reaction performed under standard conditions (red); reaction with additional 0.1 equivalents of water (blue).

Next, we studied the contribution of the product **3a** and side products **11** and **13** as potential inhibitors in the reaction. Three independent experiments were set up, where the additive was added to the initial reaction mixture. The outcome depicted in Figure SI-31 indicates that the presence of **11** and **13** can led to a substantial catalyst inhibition. On the other hand, the presence of **3a** seems to have little effect on the reaction rate. Due to the complexity of the reaction system, further inhibition contributions were not investigated.

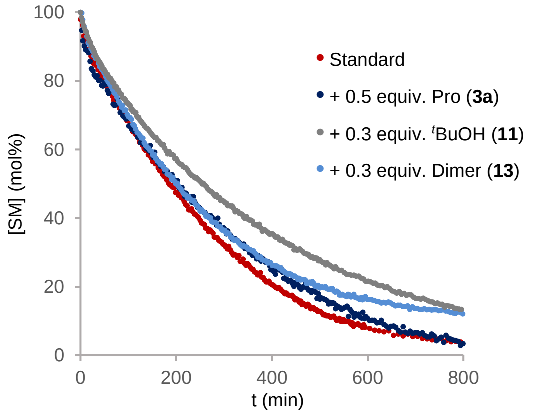

Figure SI-31. Concentration plots obtained from ^{1}H NMR reaction monitoring of **1a**. Reaction performed under standard conditions (red); reaction with additional 0.5 equivalents of **3a** (blue); reaction with additional 0.3 equivalents of **11** (grey); reaction with additional 0.5 equivalents of **13** (light blue).

8.3.8. Proposed Catalytic Cycle

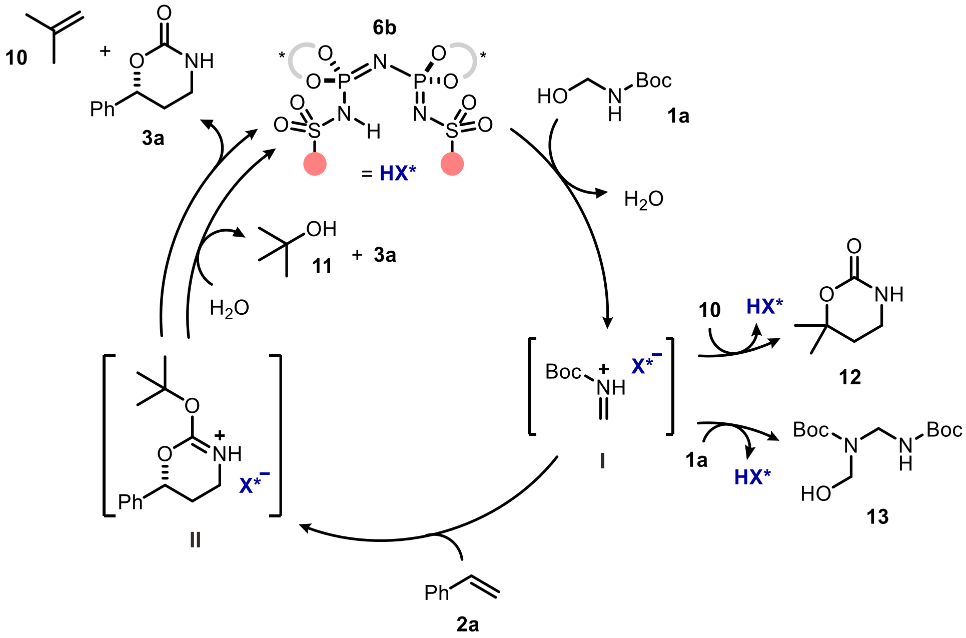

Figure SI-32. Proposed reaction mechanism for the IDPi-catalyzed hetero-Diels–Alder reaction of in situ generated *N*-Boc-formaldimine with olefins.

9. Copies of NMR Spectra

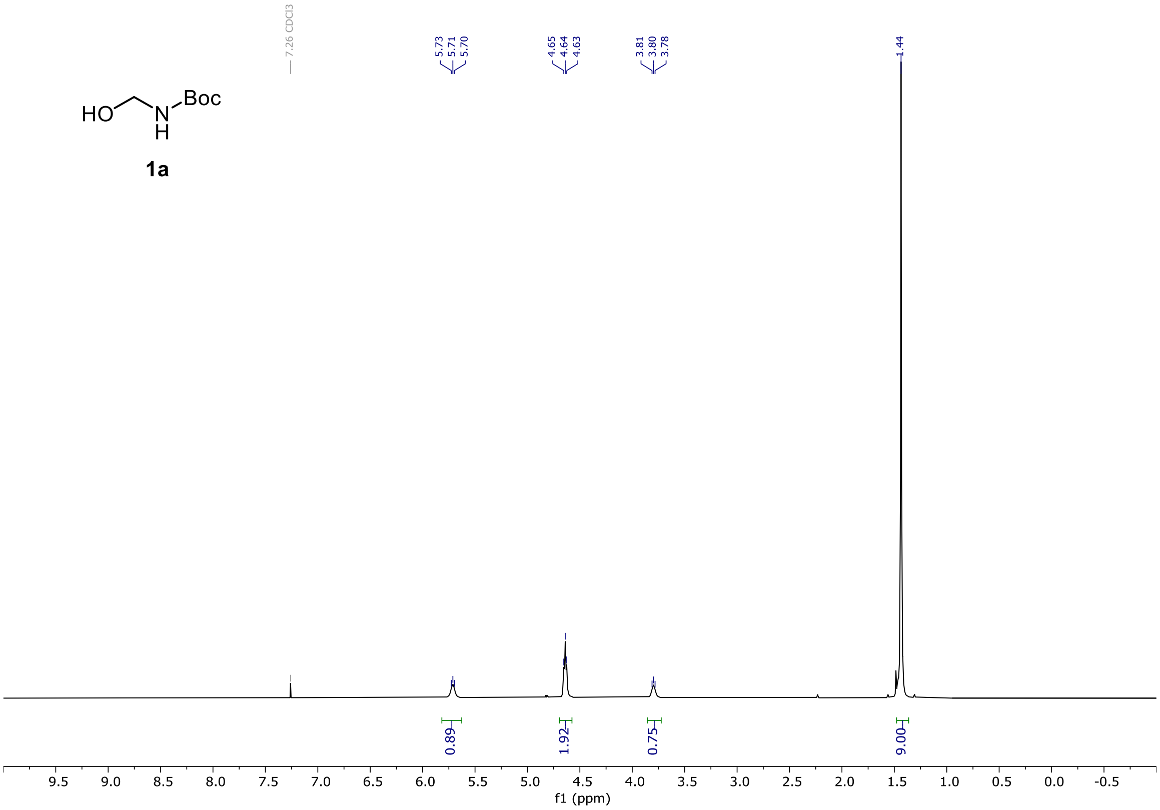

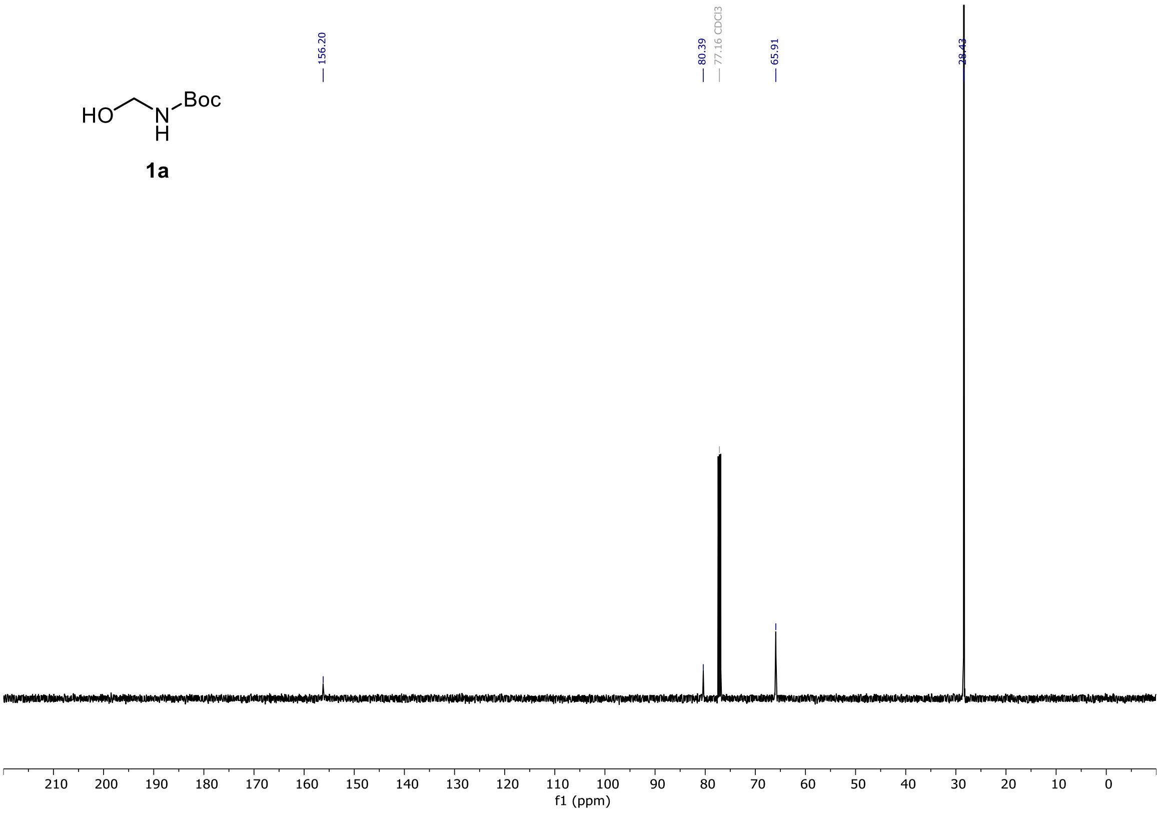

NMR spectra for compound **1a**: ^{1}H (501 MHz) and ^{13}C (126 MHz), in $CDCl_3$.

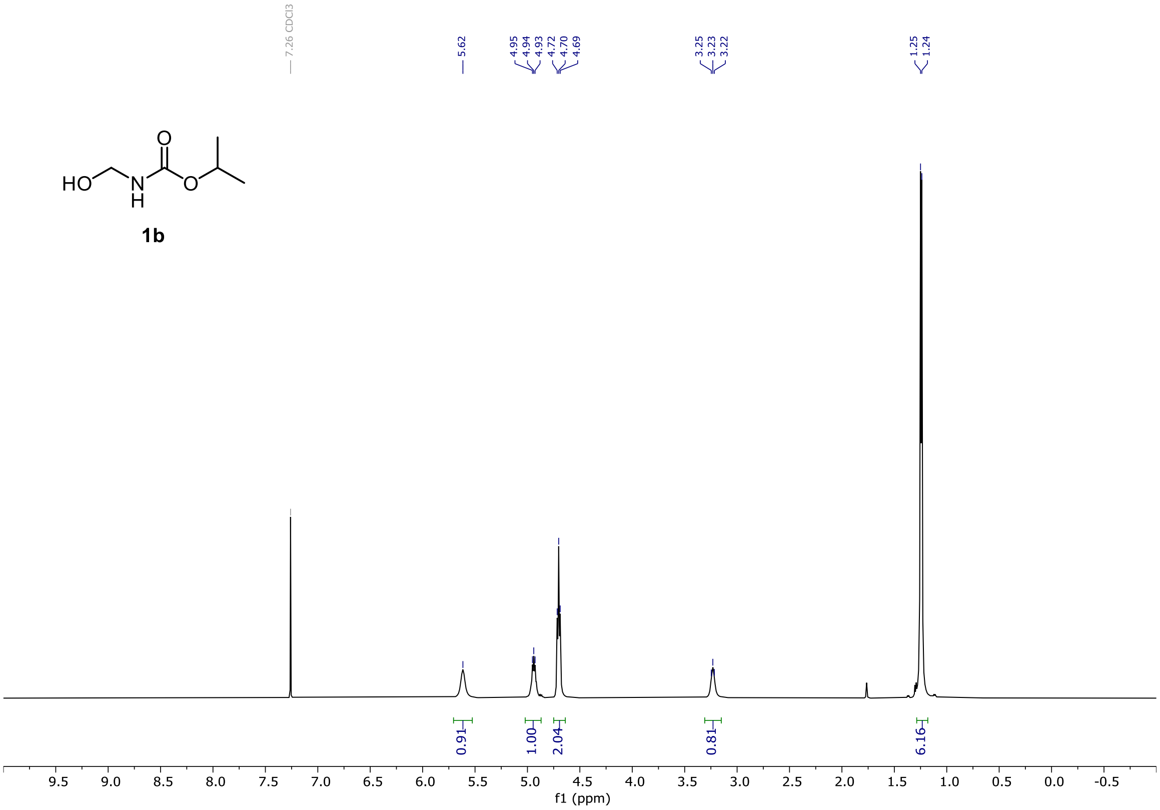

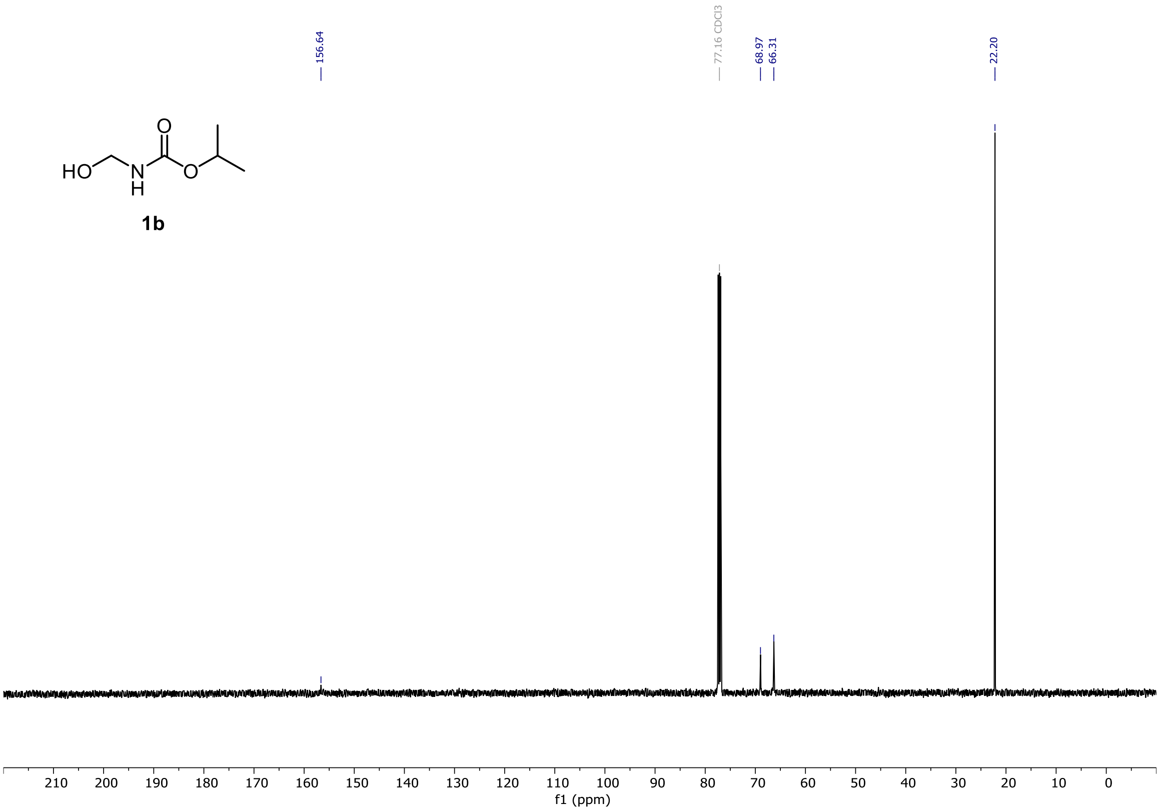

NMR spectra for compound **1b**: ^{1}H (501 MHz) and ^{13}C (126 MHz), in $CDCl_3$.

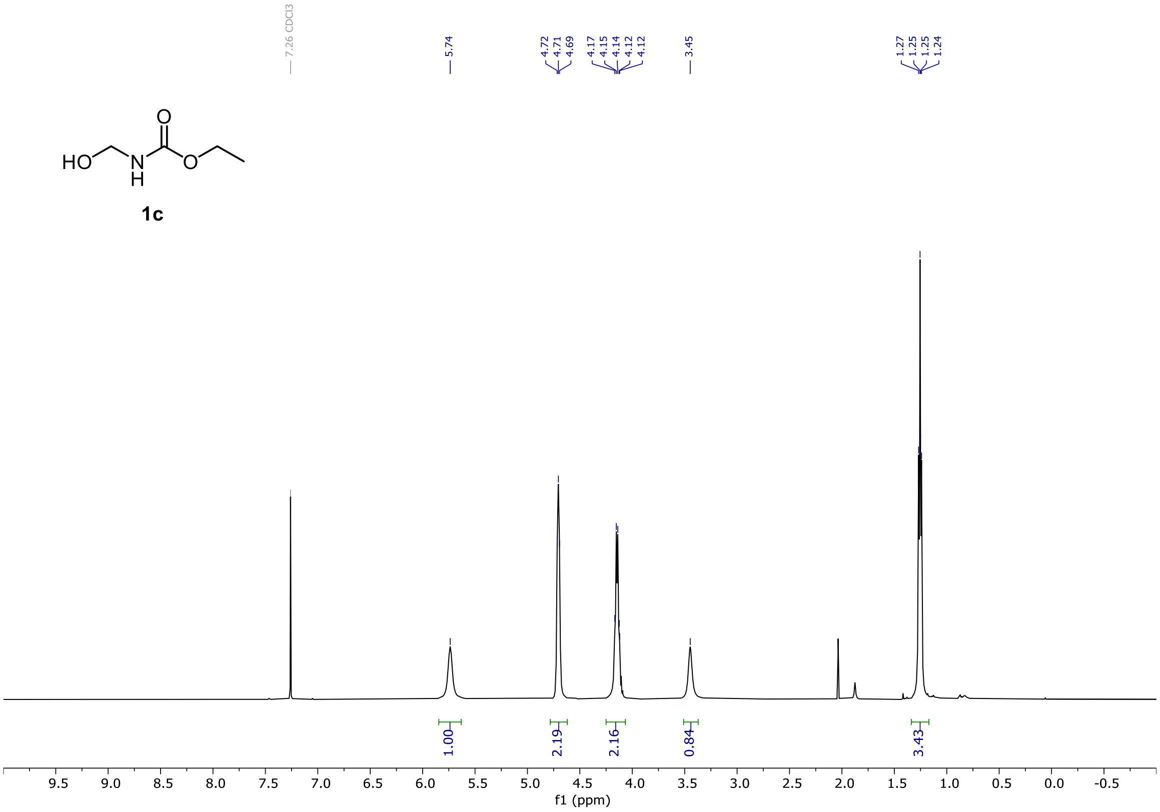

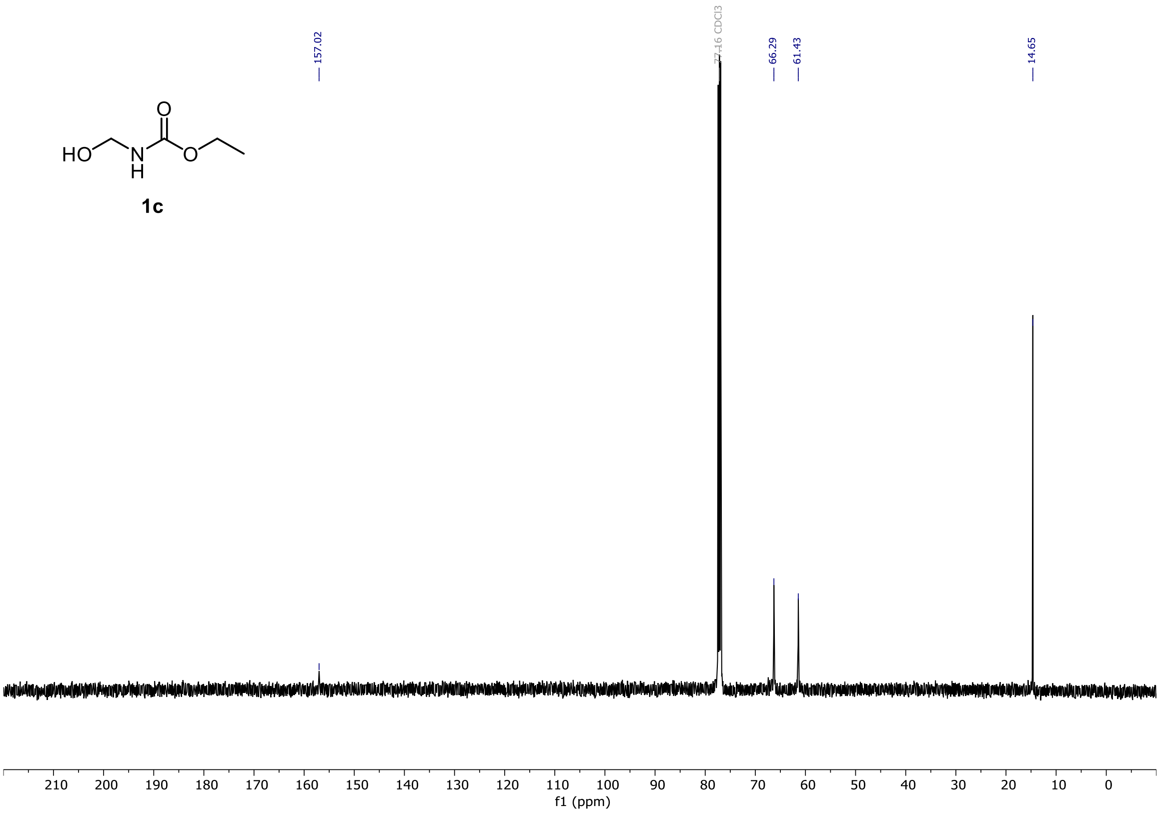

NMR spectra for compound **1c**: ^{1}H (501 MHz) and ^{13}C (126 MHz), in $CDCl_3$.

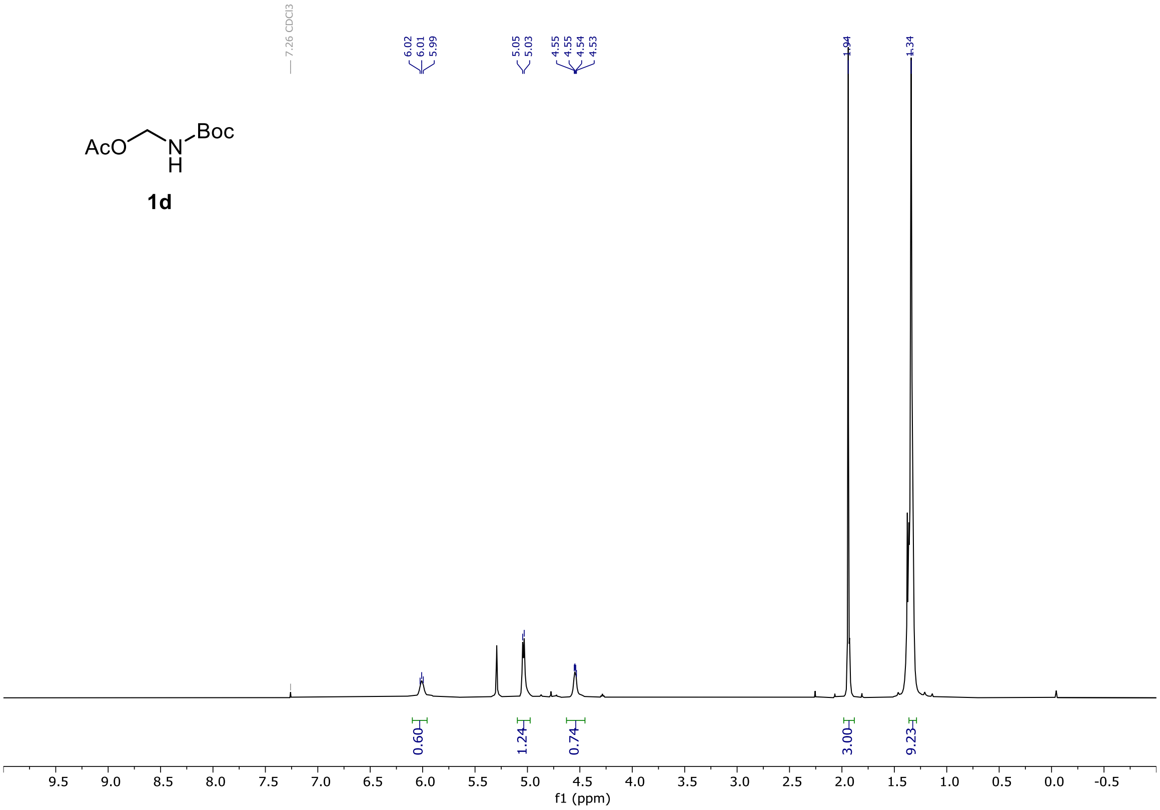

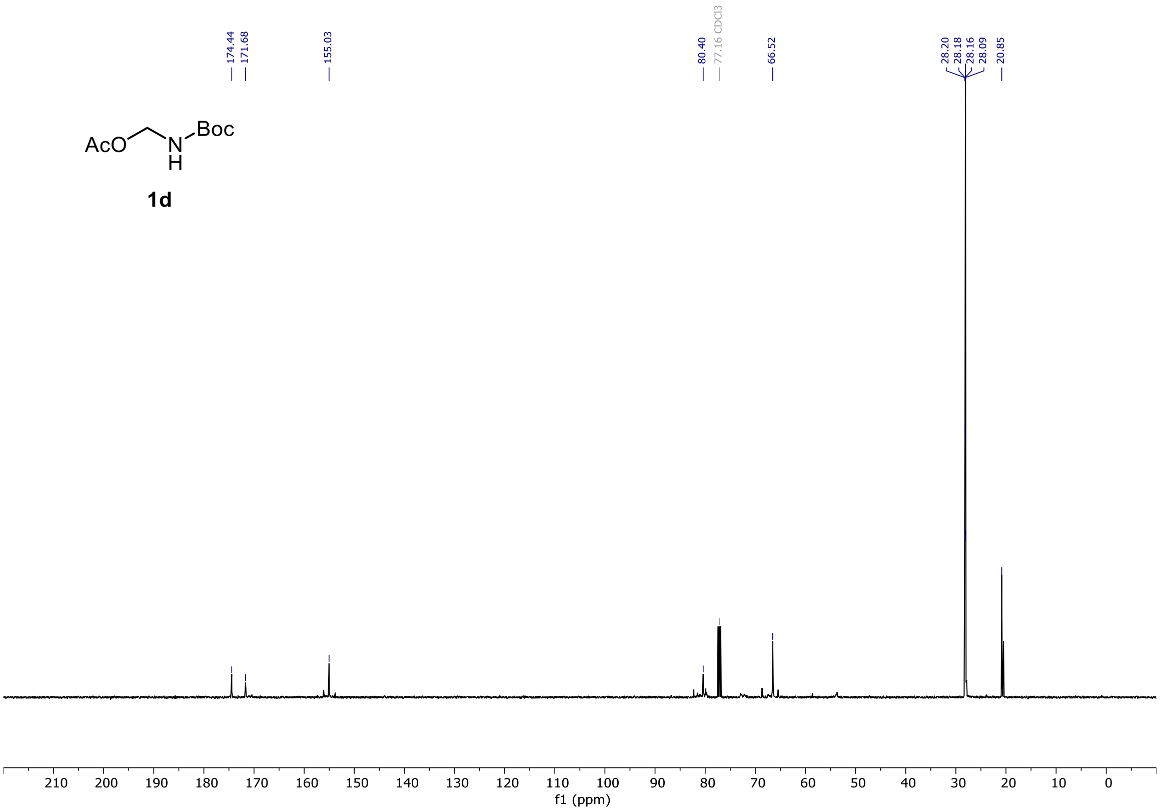

NMR spectra for compound **1d**: ^{1}H (501 MHz) and ^{13}C (126 MHz), in $CDCl_3$.

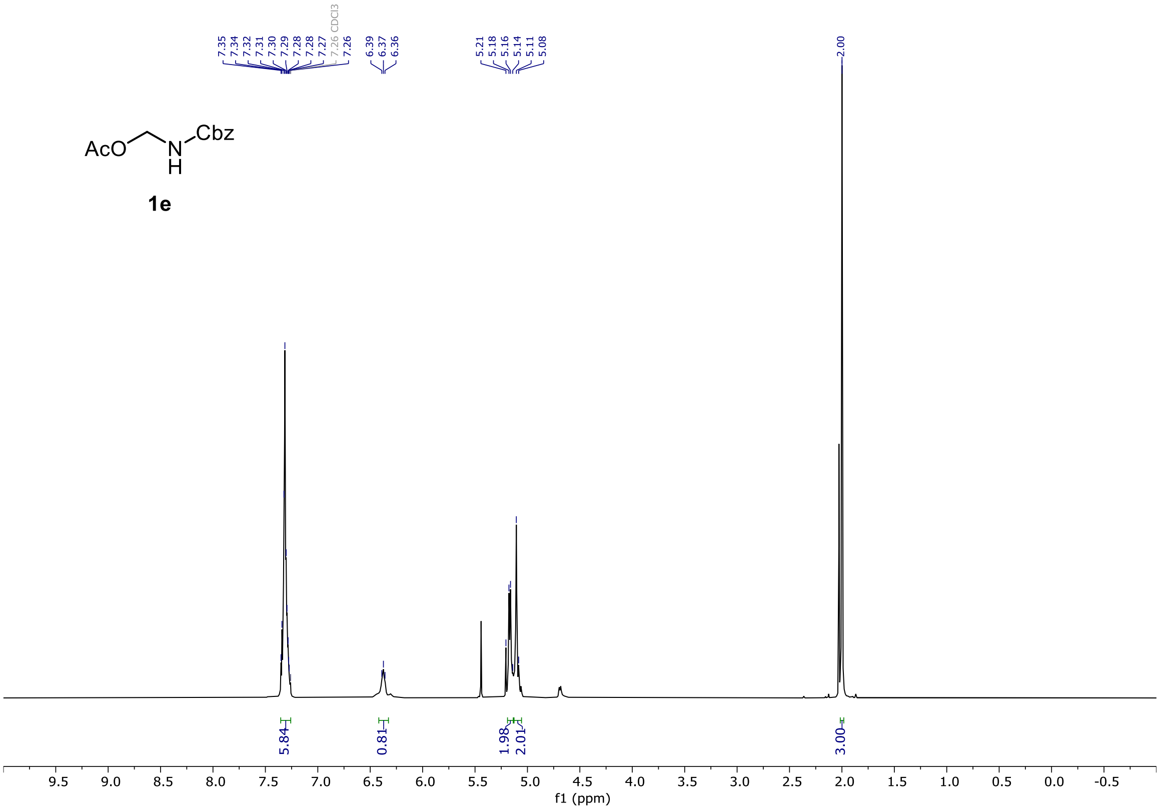

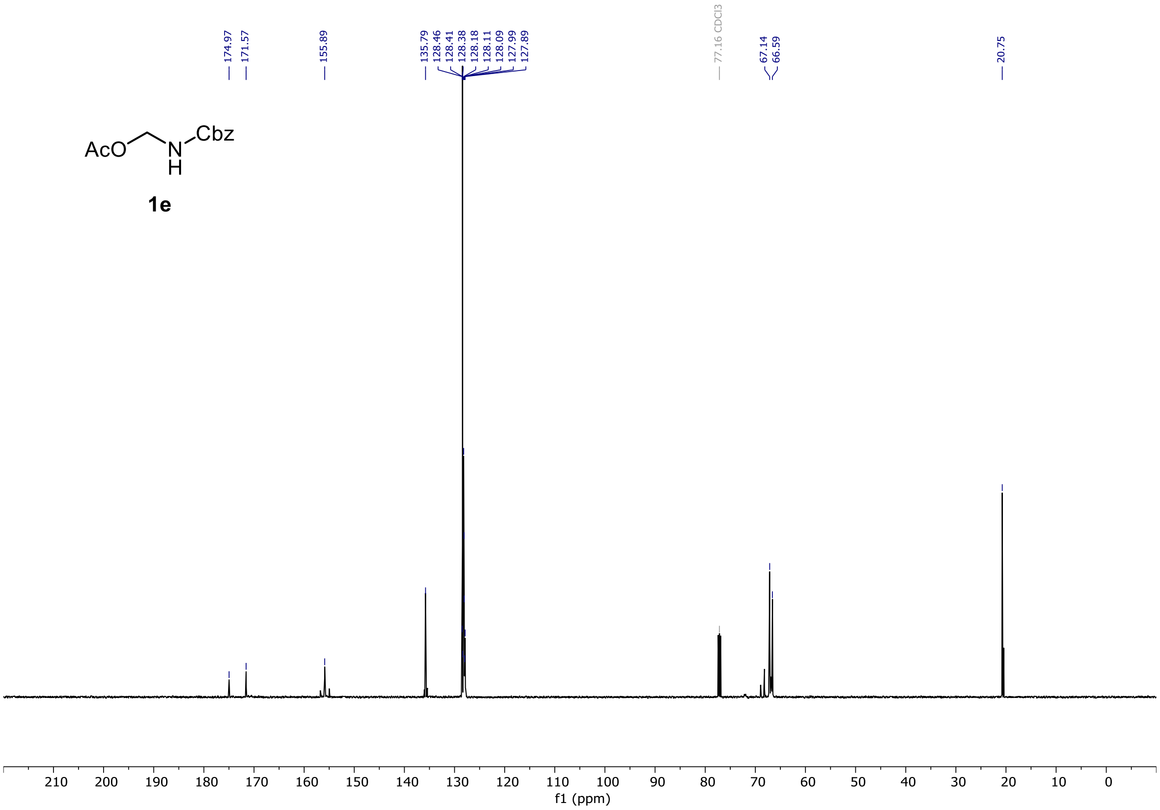

NMR spectra for compound **1e**: ^{1}H (501 MHz) and ^{13}C (126 MHz), in $CDCl_3$.

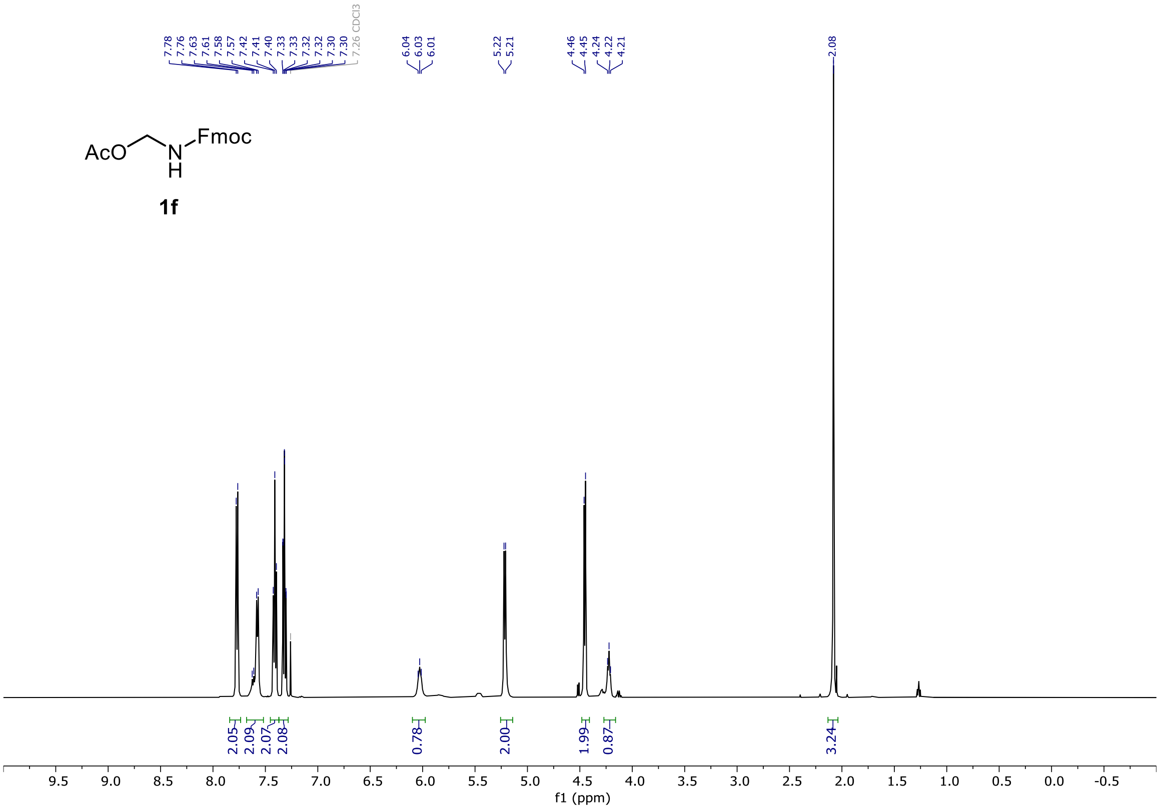

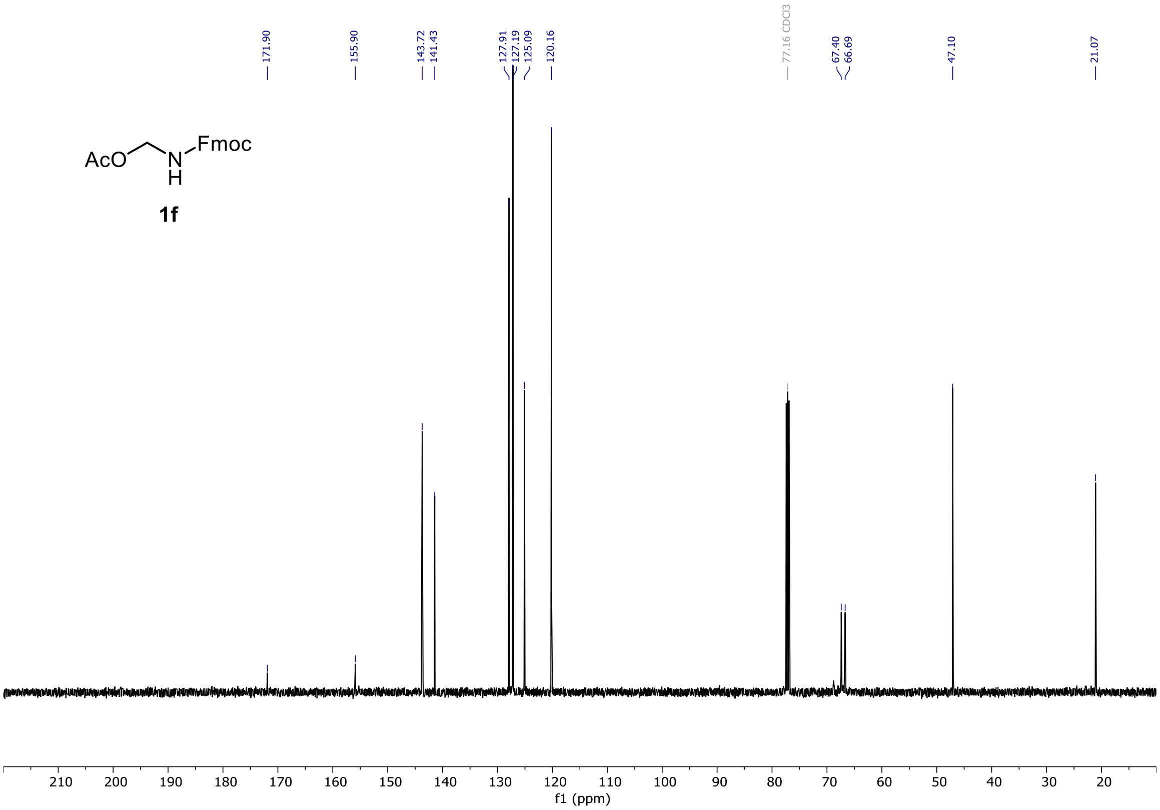

NMR spectra for compound **1f**: ^{1}H (501 MHz) and ^{13}C (126 MHz), in $CDCl_3$.

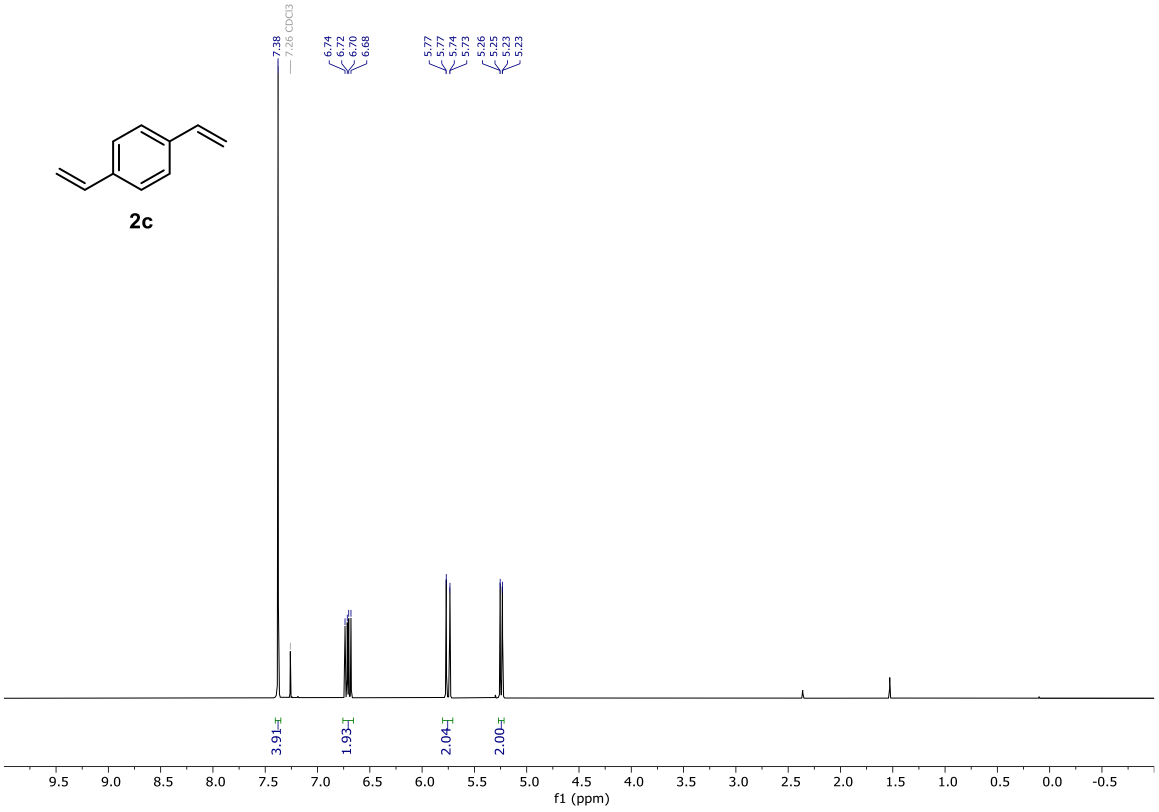

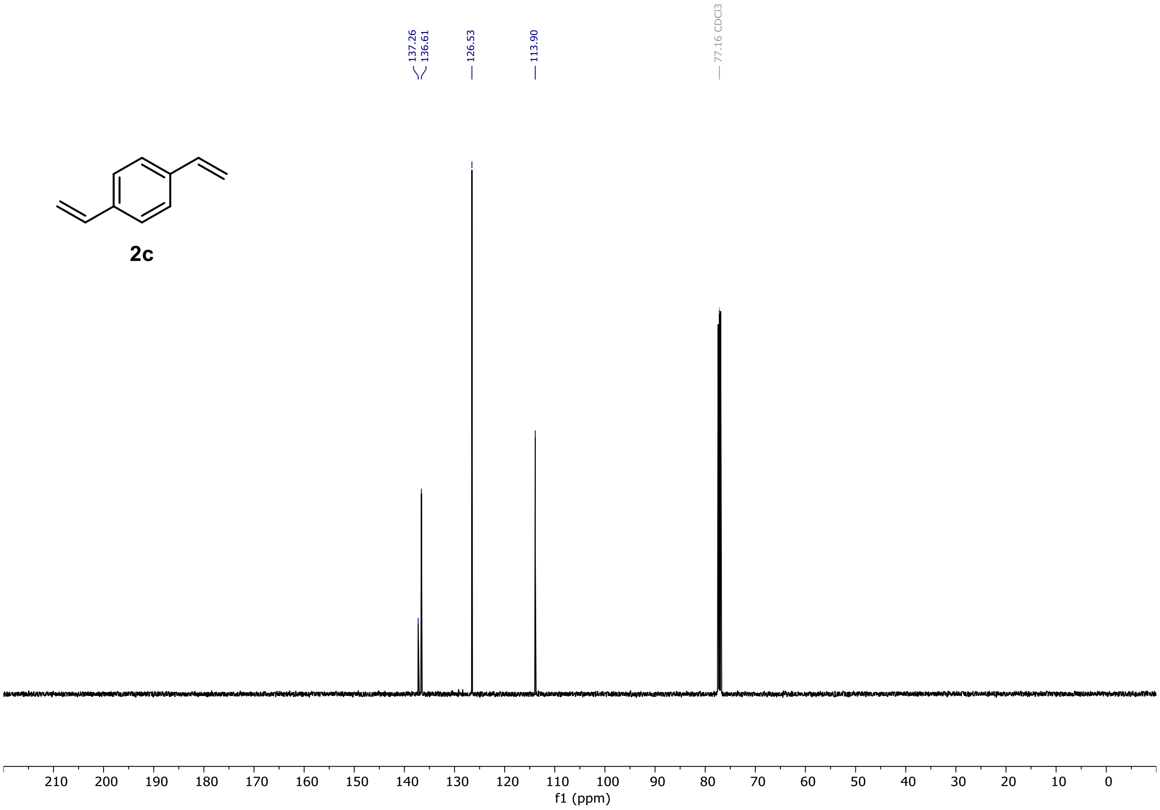

NMR spectra for compound **2c**: ^{1}H (501 MHz) and ^{13}C (126 MHz), in $CDCl_3$.

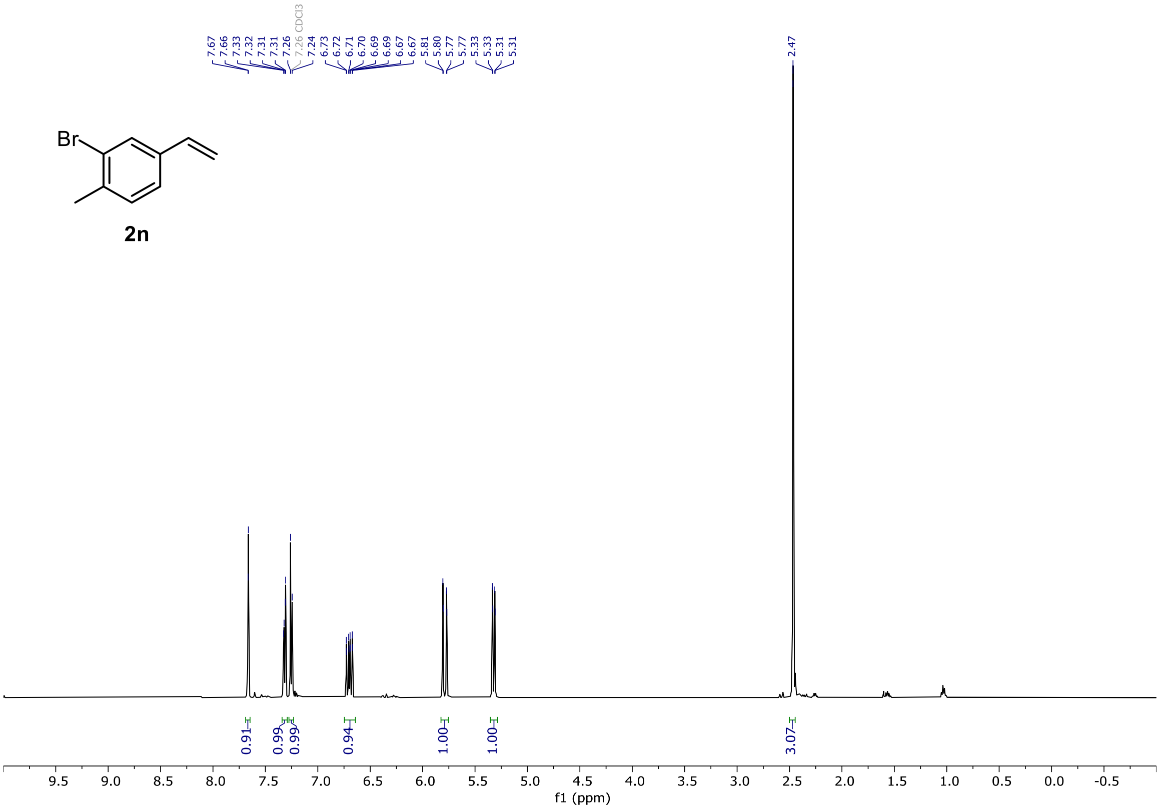

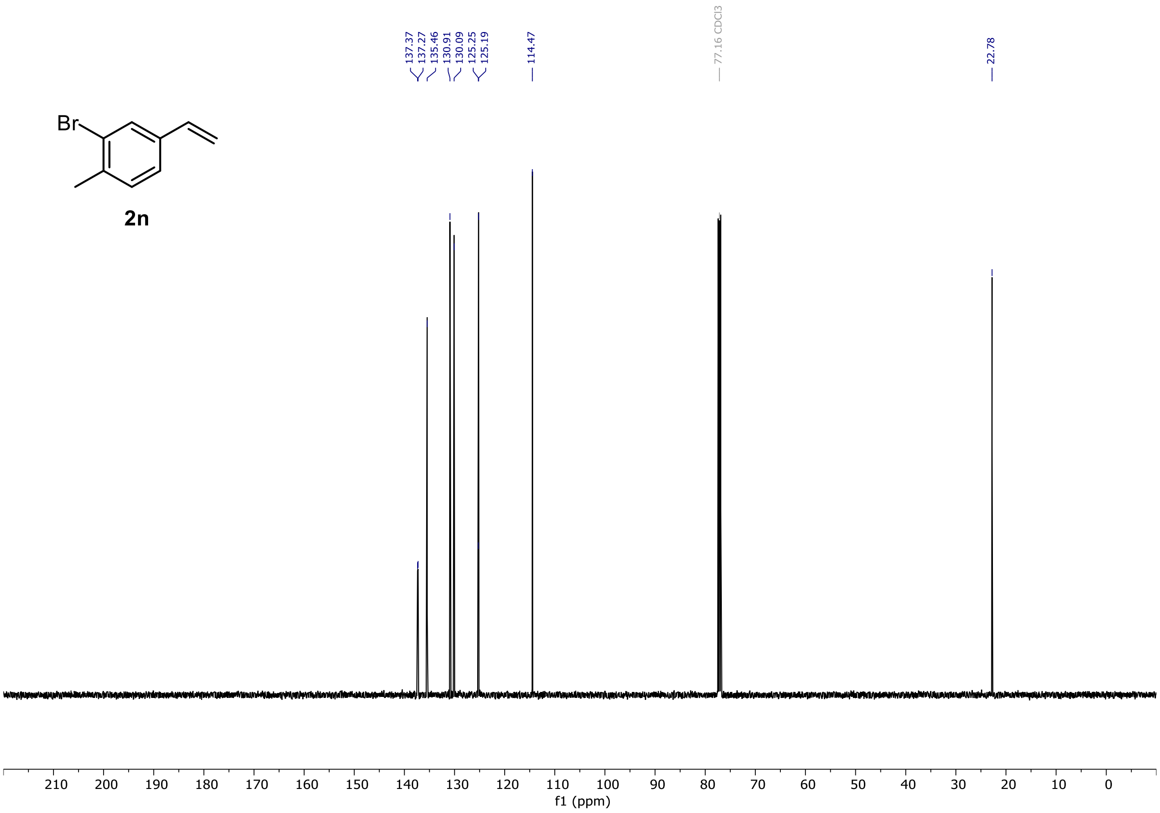

NMR spectra for compound **2n**: ^{1}H (501 MHz) and ^{13}C (126 MHz), in $CDCl_3$.

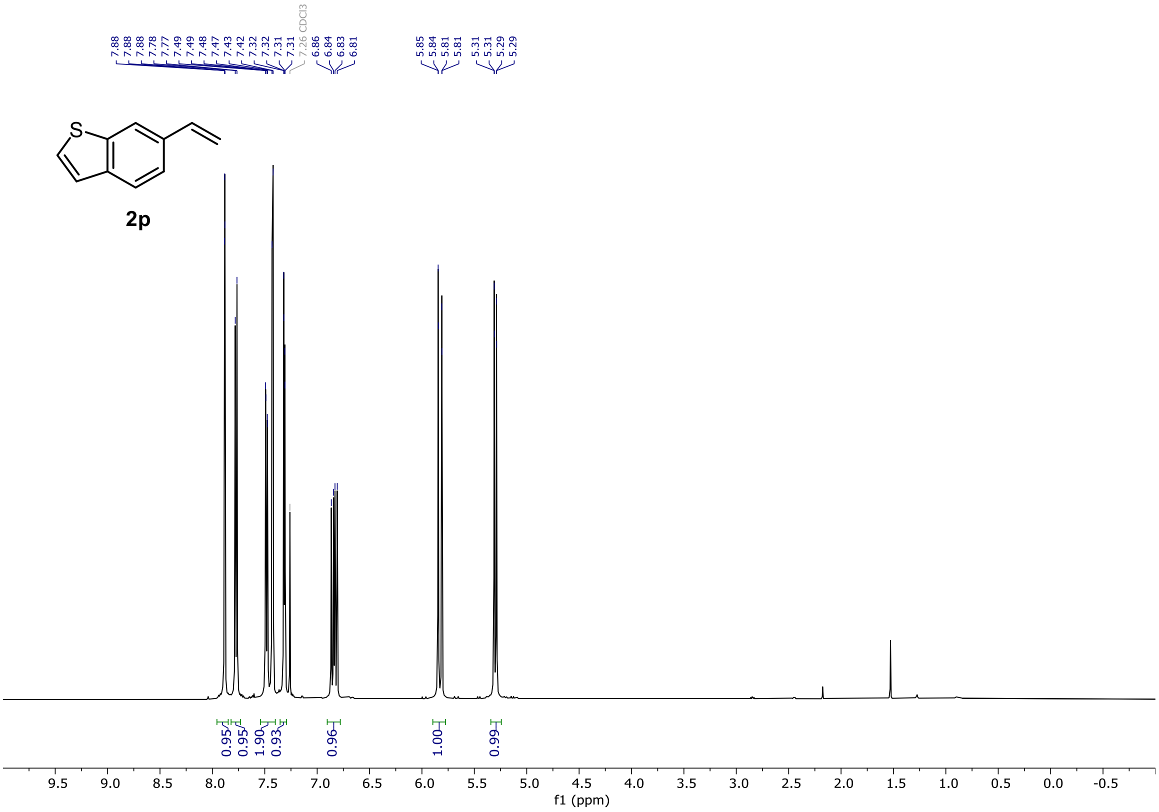

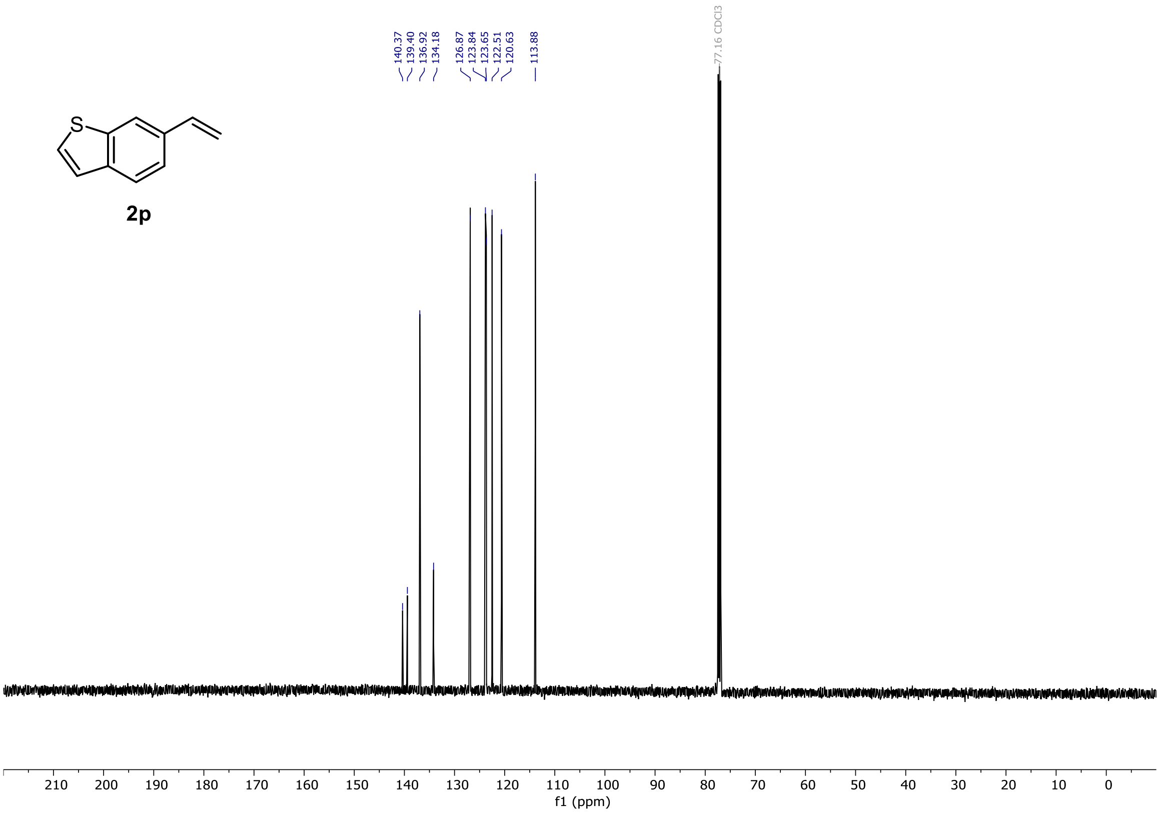

NMR spectra for compound **2p**: ^{1}H (501 MHz) and ^{13}C (126 MHz), in $CDCl_3$.

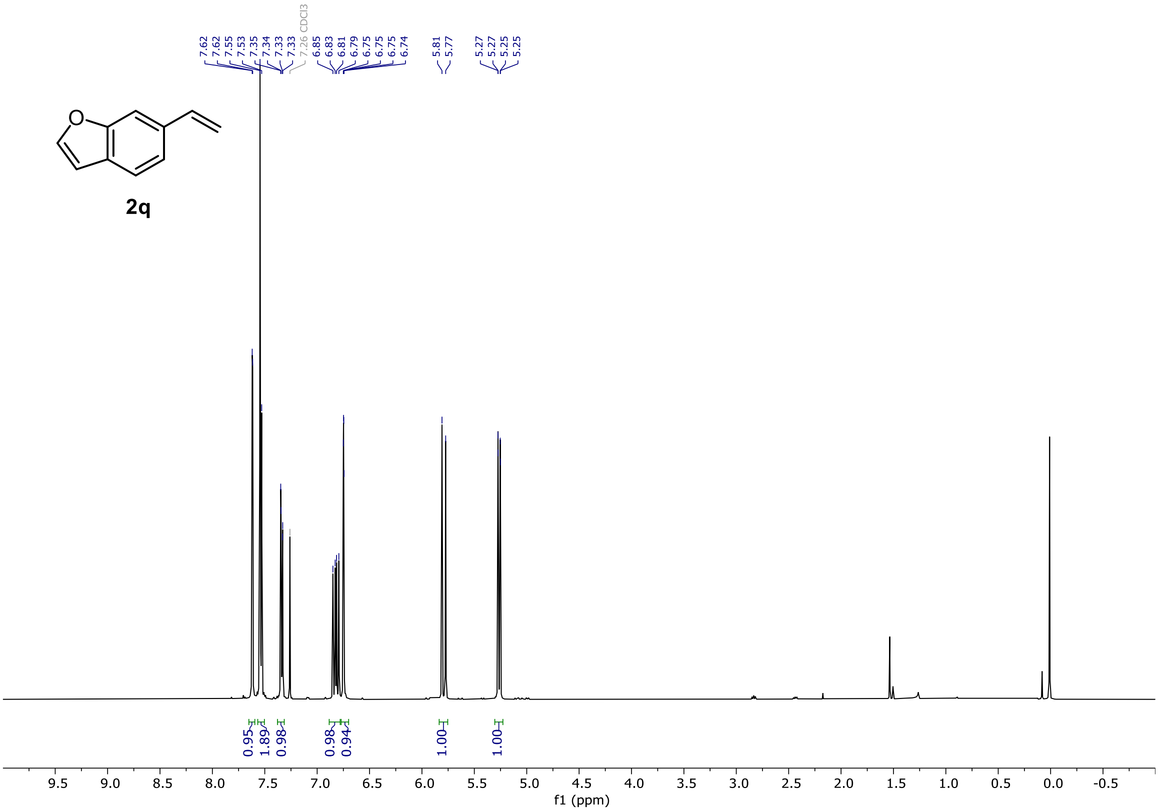

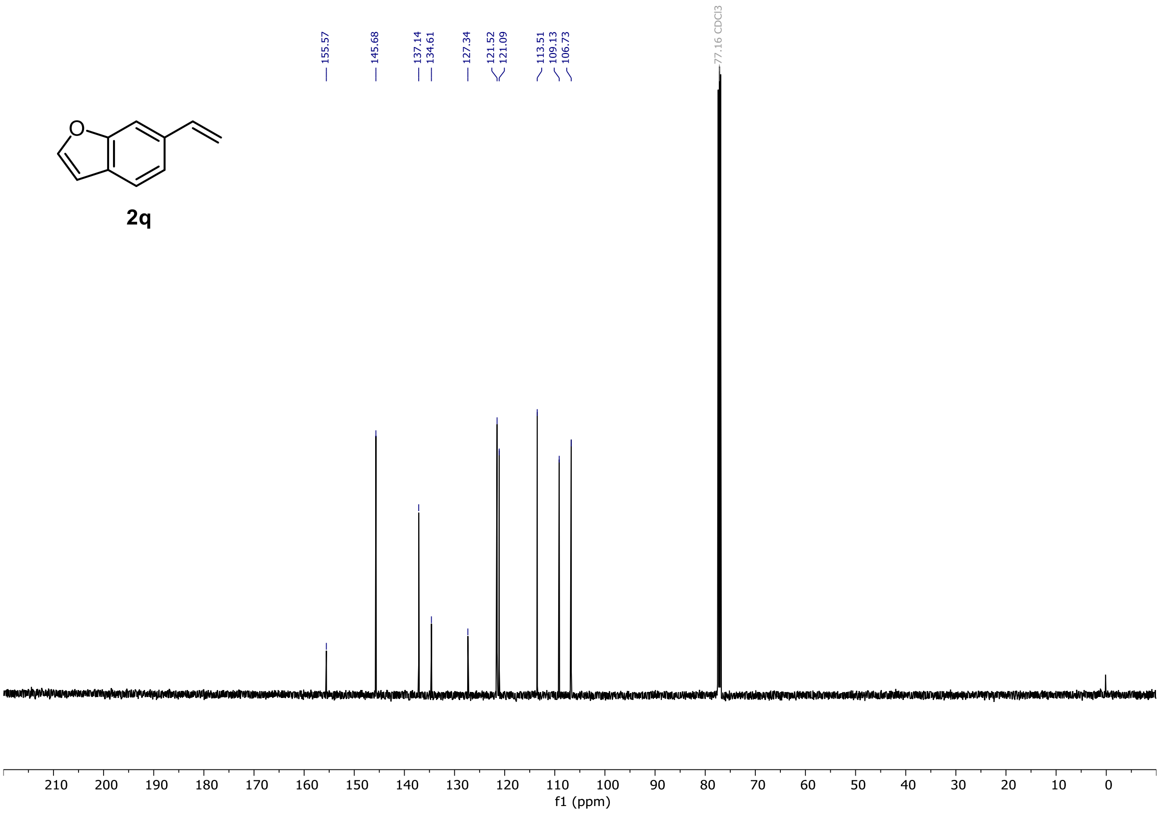

NMR spectra for compound **2q**: ^{1}H (501 MHz) and ^{13}C (126 MHz), in $CDCl_3$.

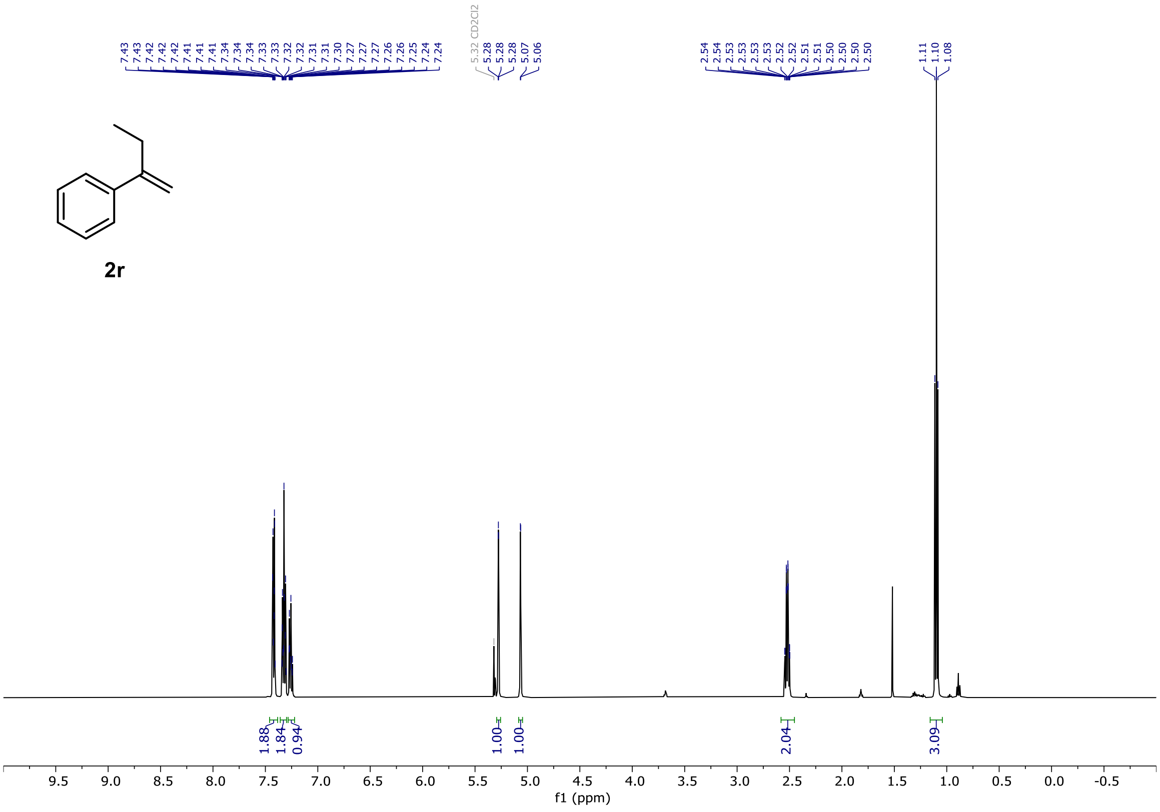

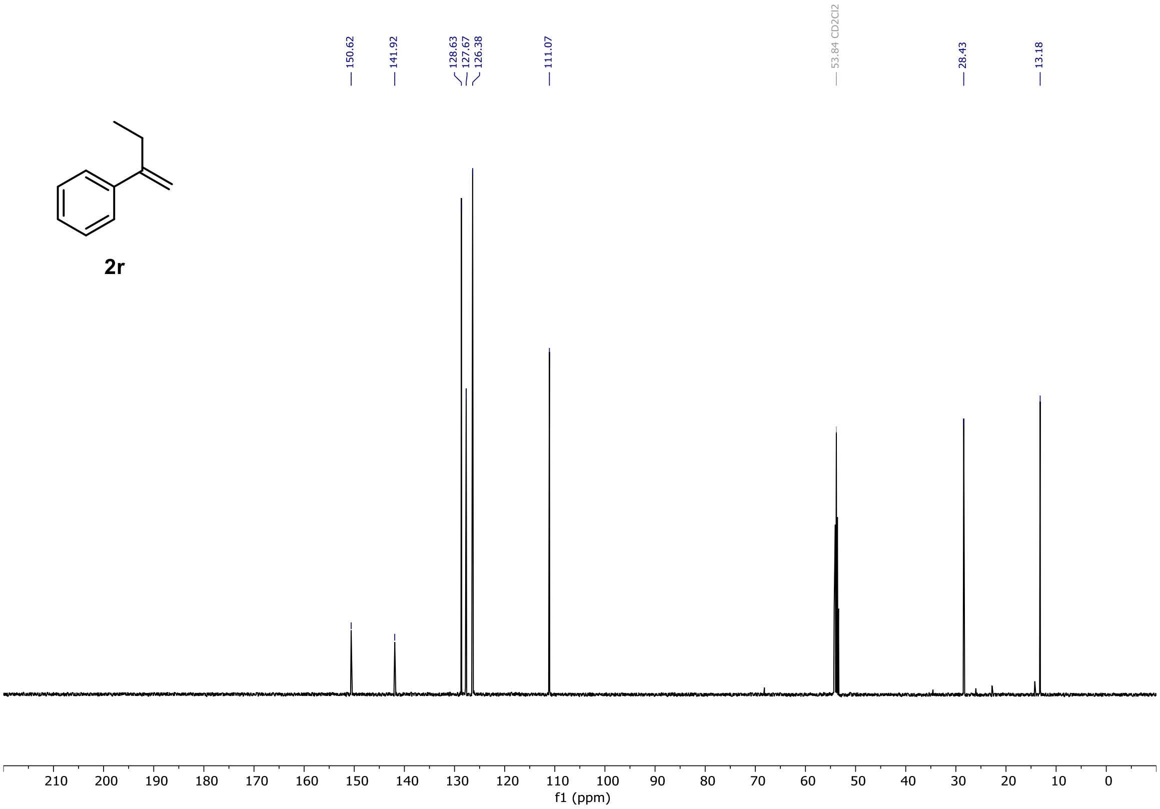

NMR spectra for compound **2r**: ^{1}H (501 MHz) and ^{13}C (126 MHz), in CD_2Cl_2.

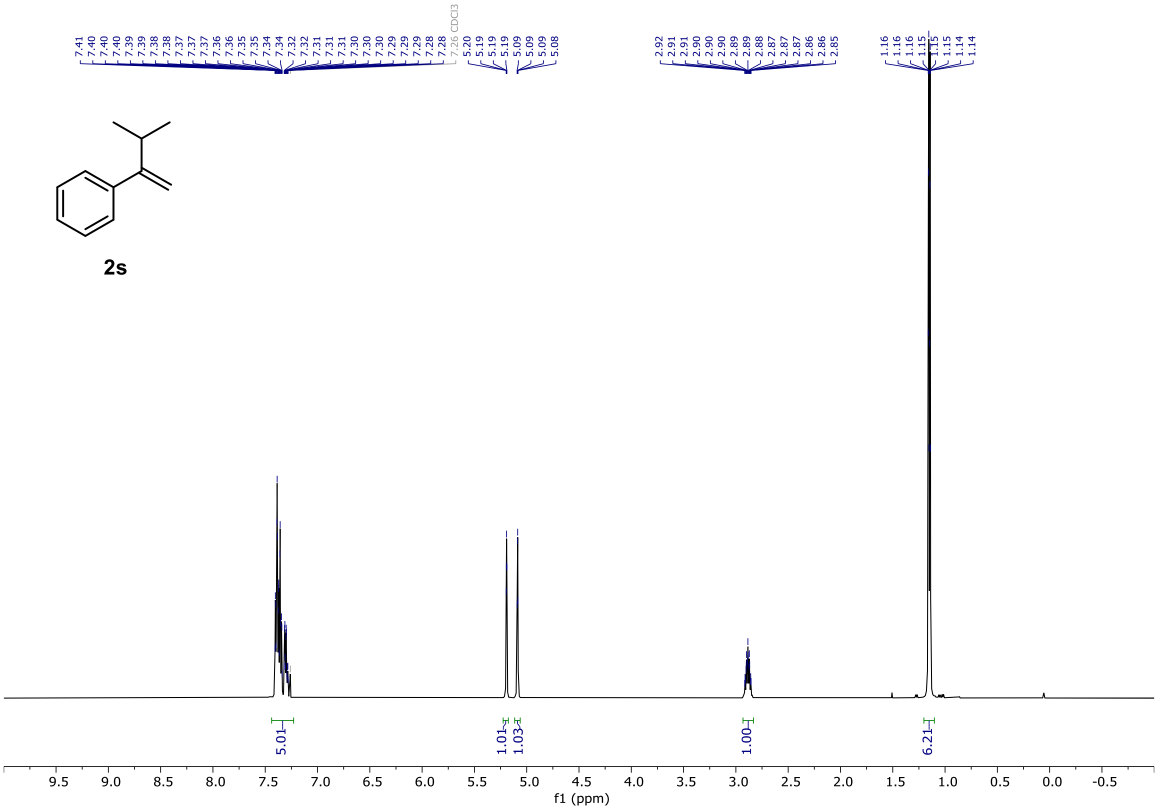

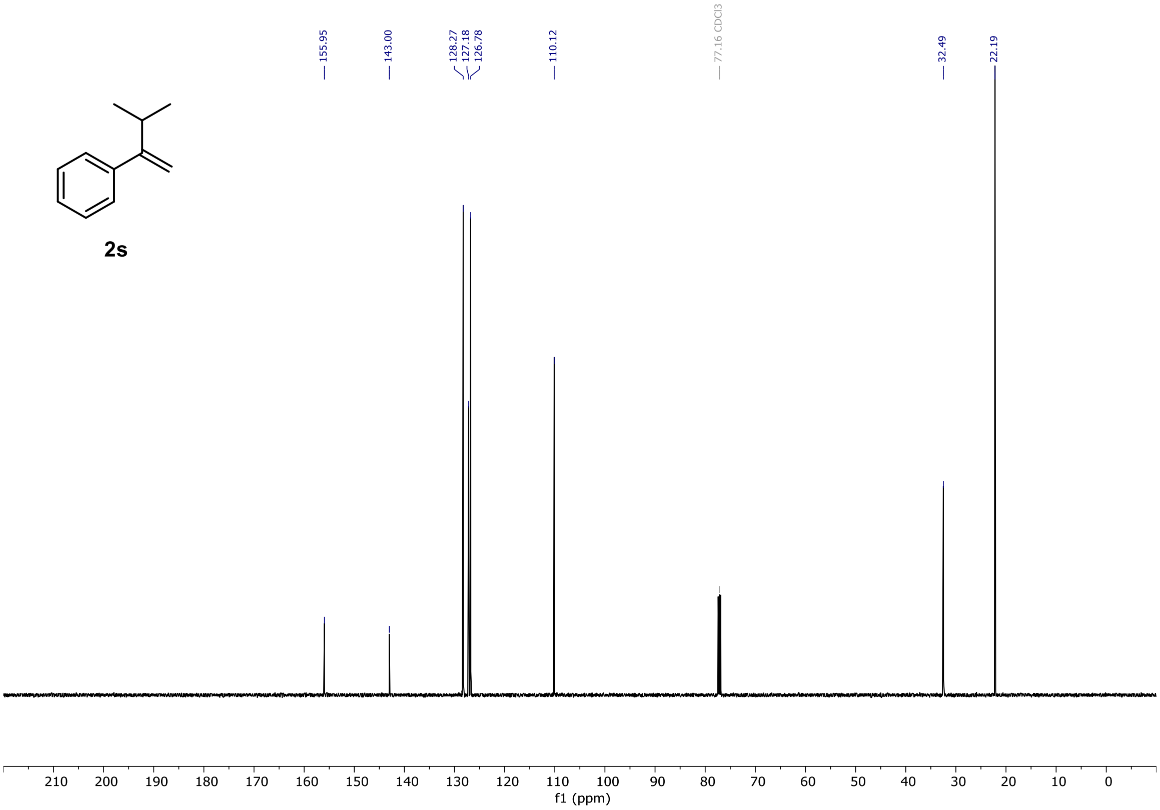

NMR spectra for compound **2s**: ^{1}H (501 MHz) and ^{13}C (126 MHz), in $CDCl_3$.

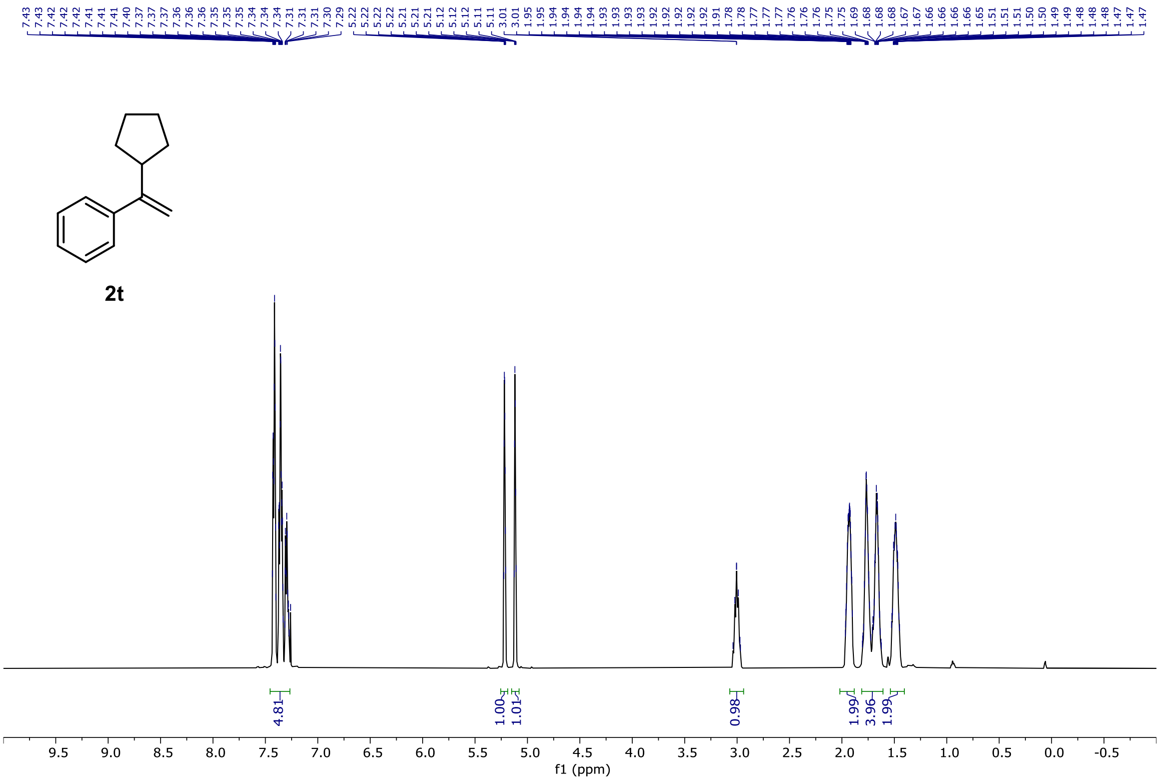

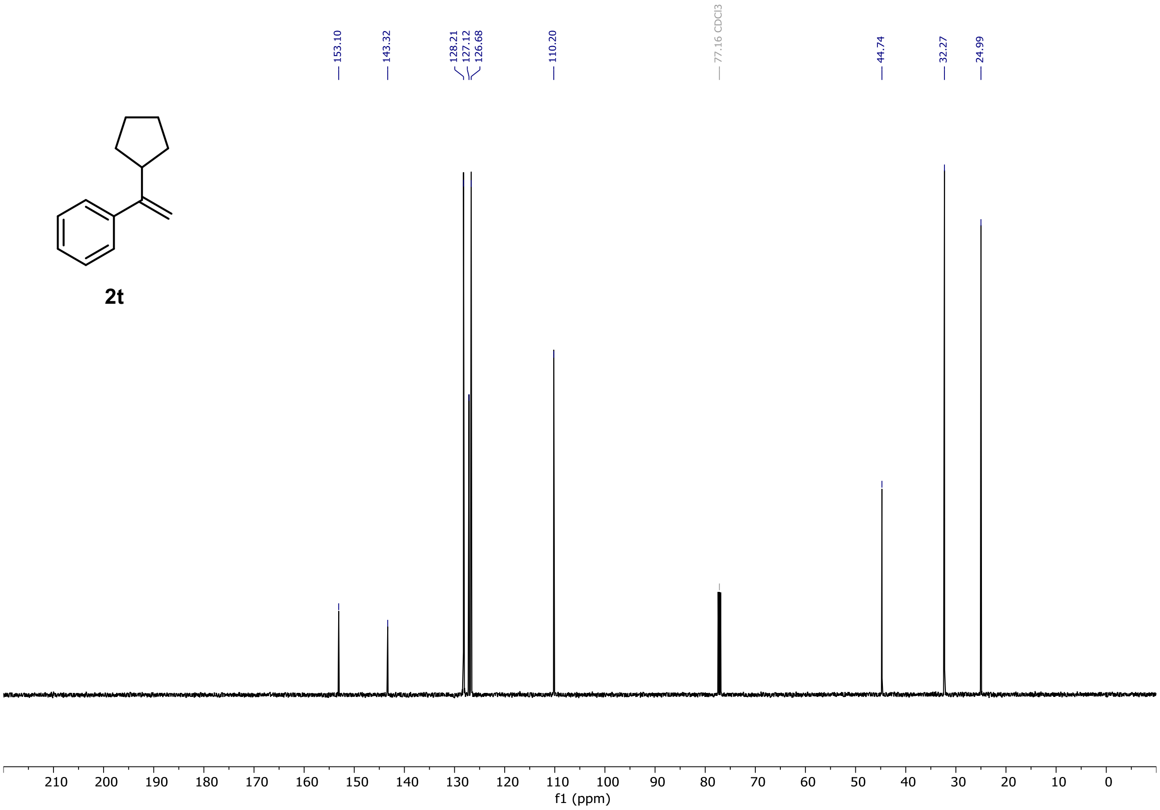

NMR spectra for compound **2t**: ^{1}H (501 MHz) and ^{13}C (126 MHz), in $CDCl_3$.

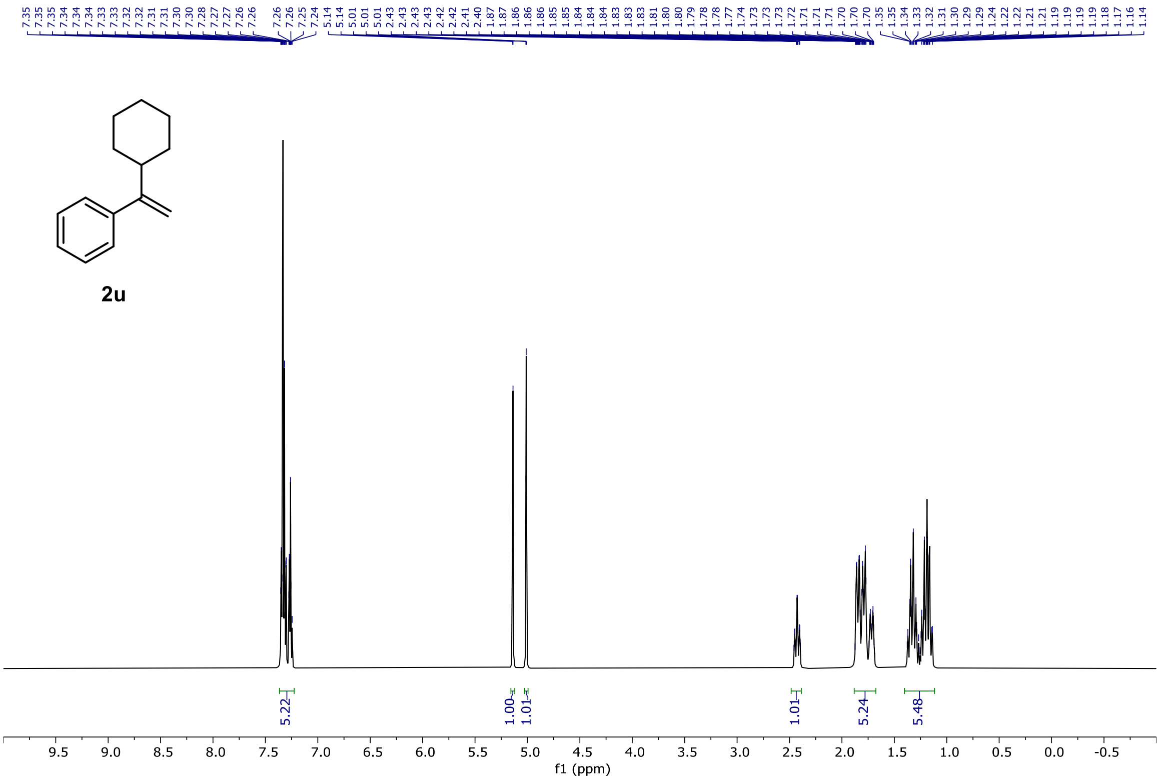

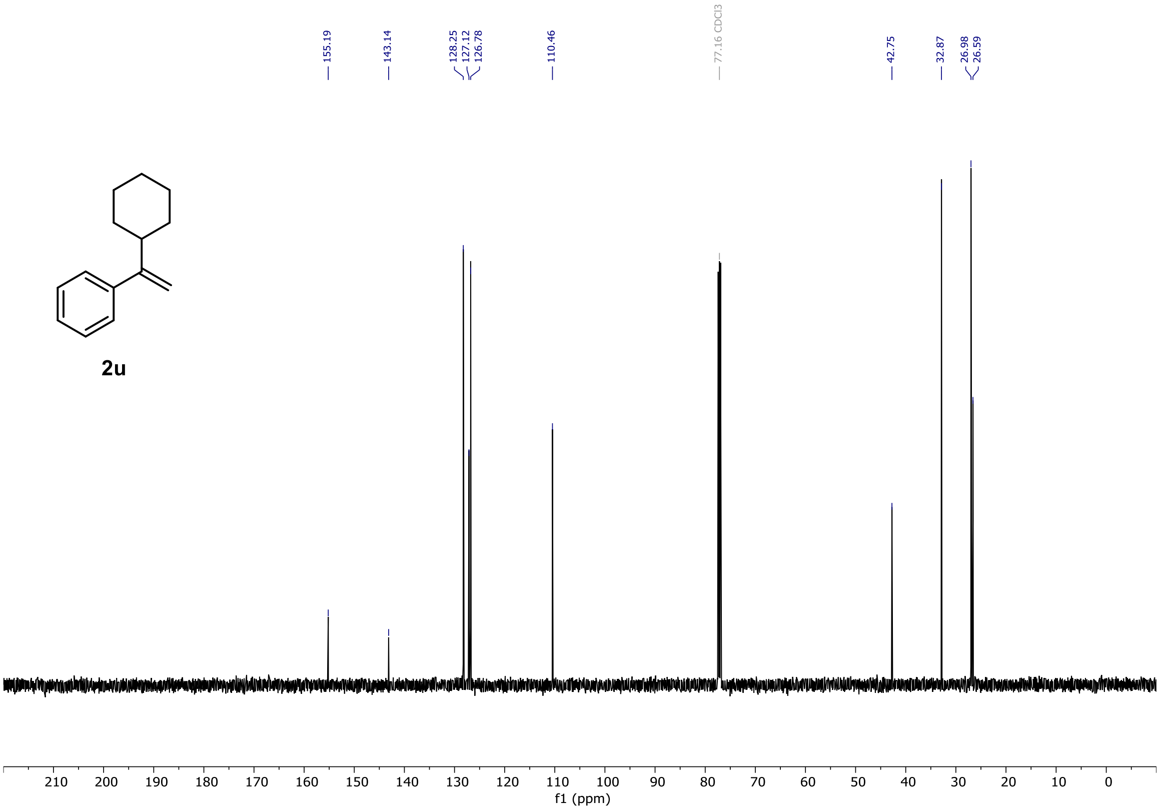

NMR spectra for compound **2u**: ^{1}H (501 MHz) and ^{13}C (126 MHz), in $CDCl_3$.

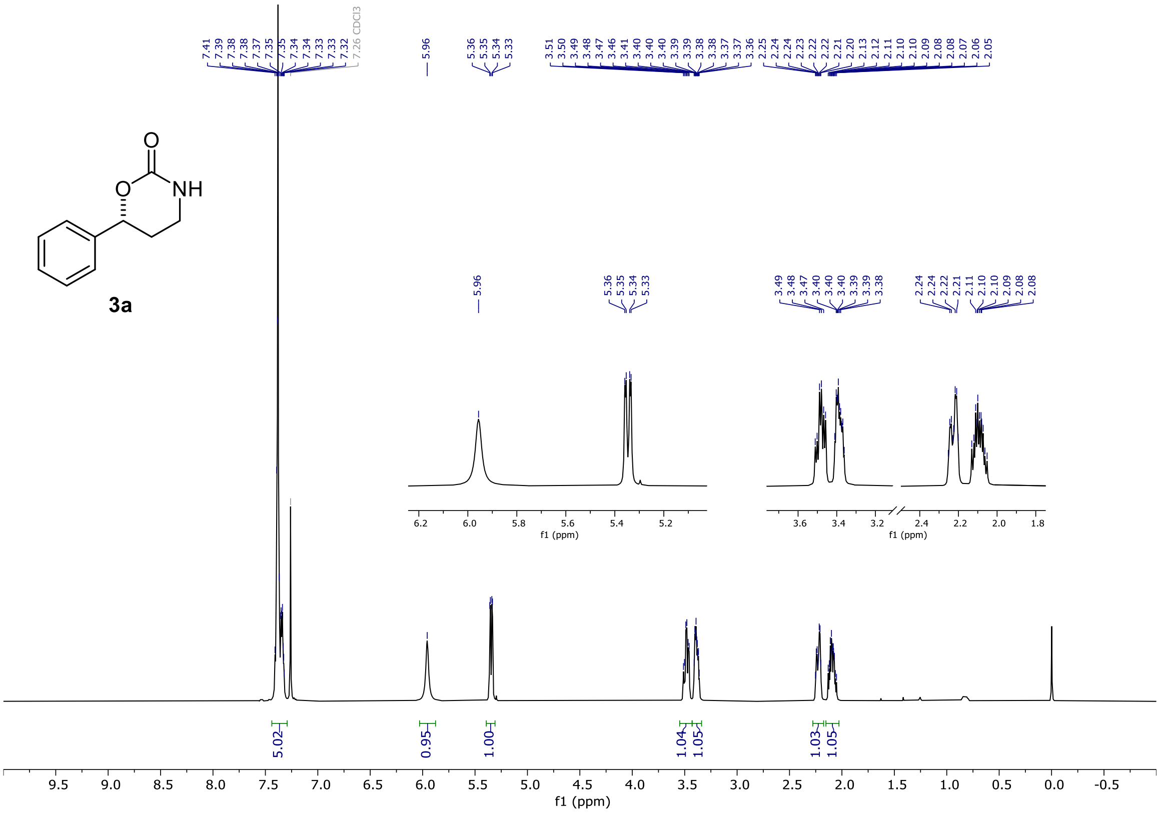

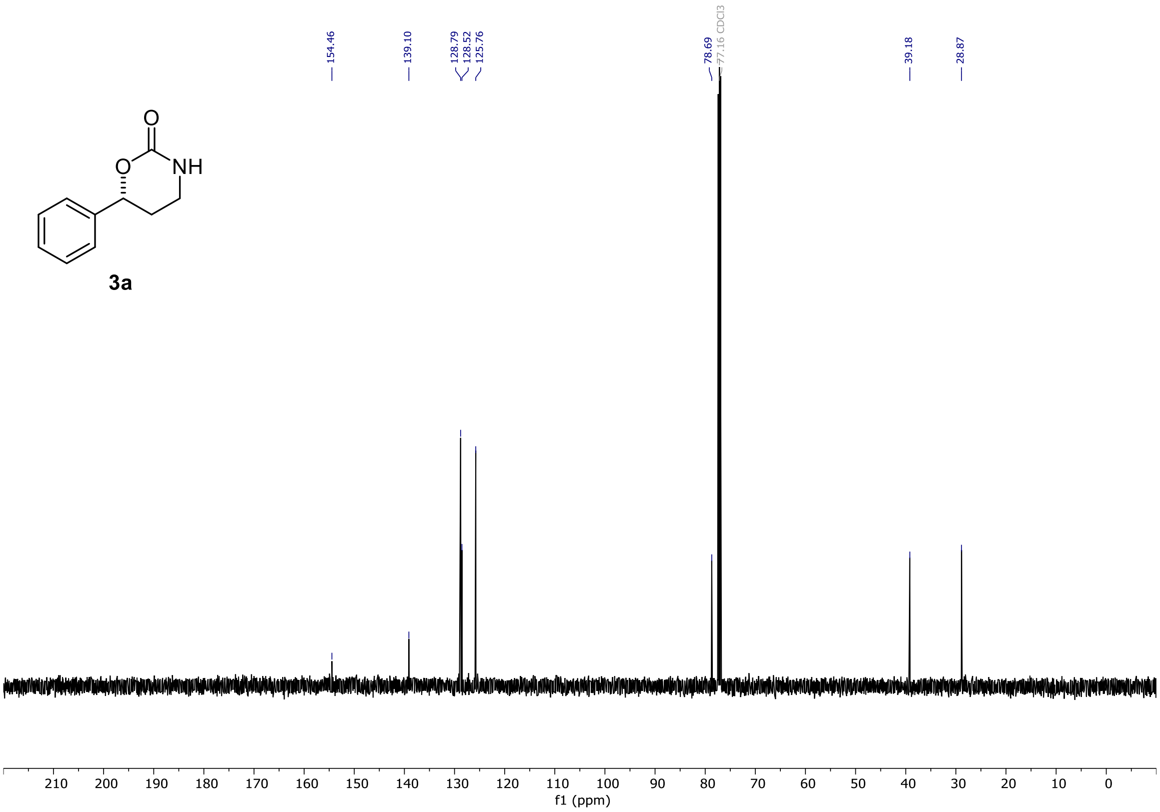

NMR spectra for compound **3a**: ^{1}H (501 MHz) and ^{13}C (126 MHz), in $CDCl_3$.

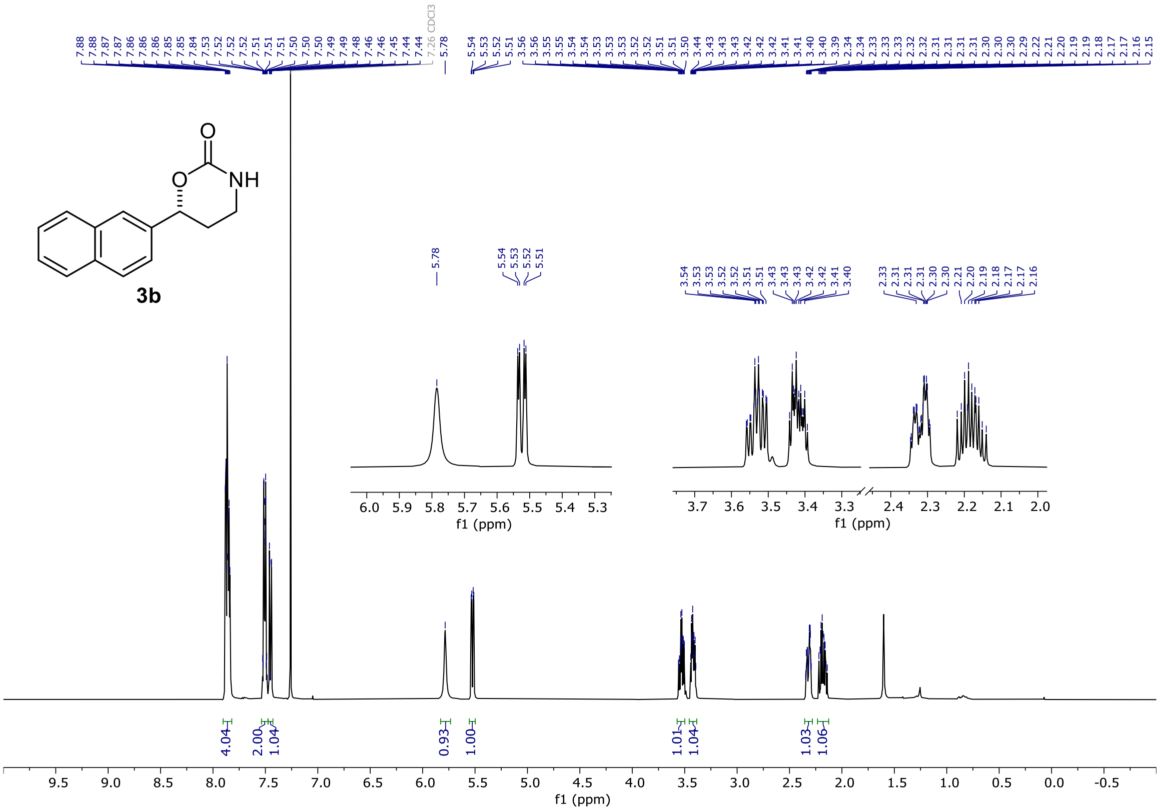

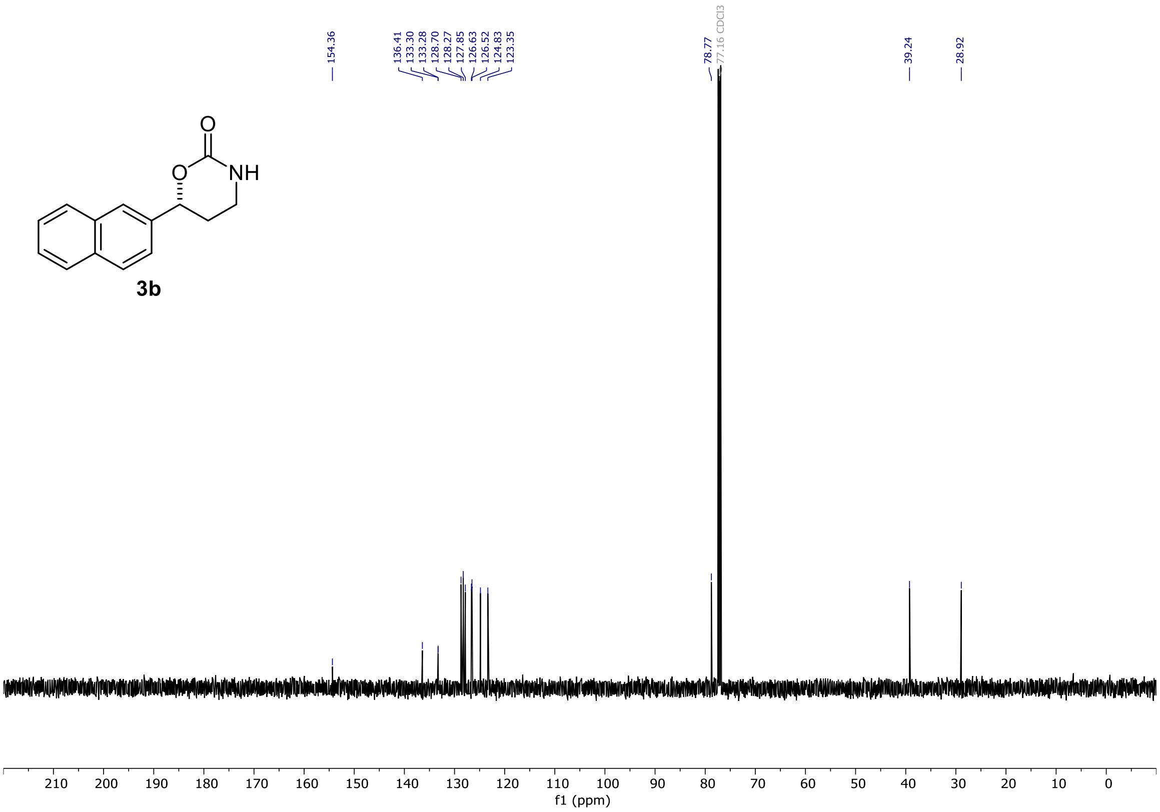

NMR spectra for compound **3b**: ^{1}H (501 MHz) and ^{13}C (126 MHz), in $CDCl_3$.

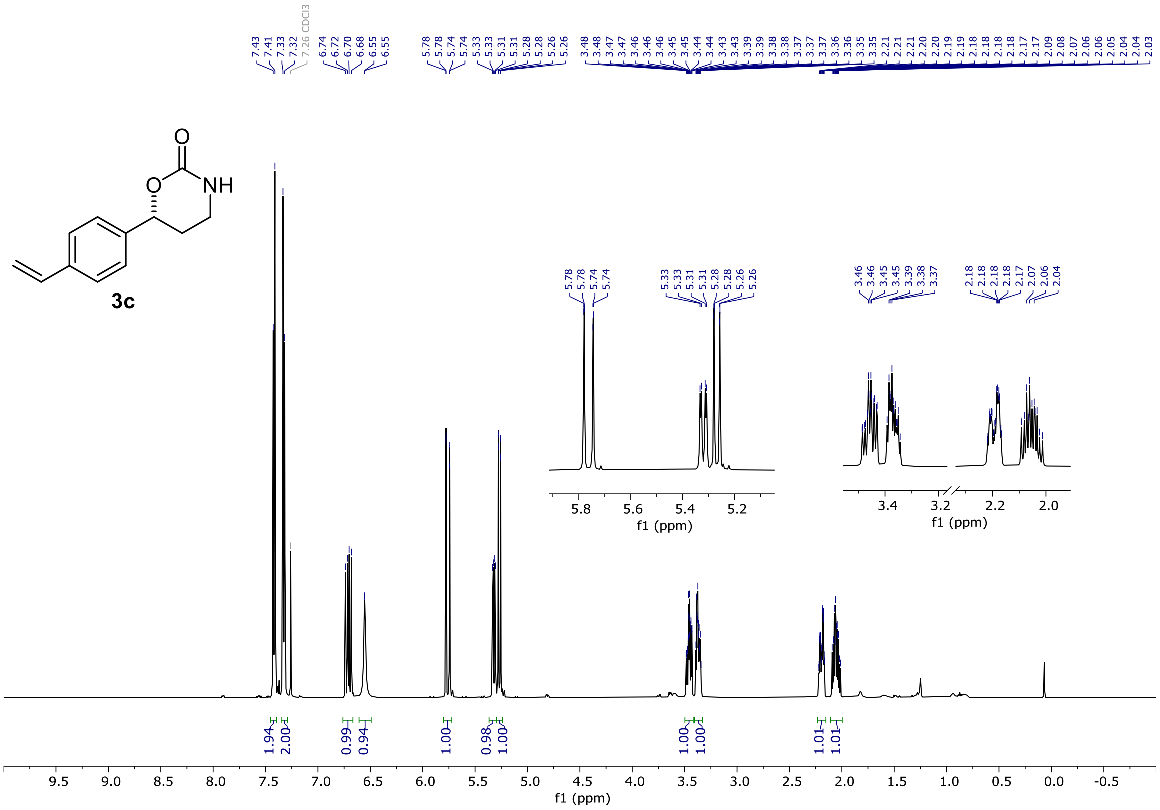

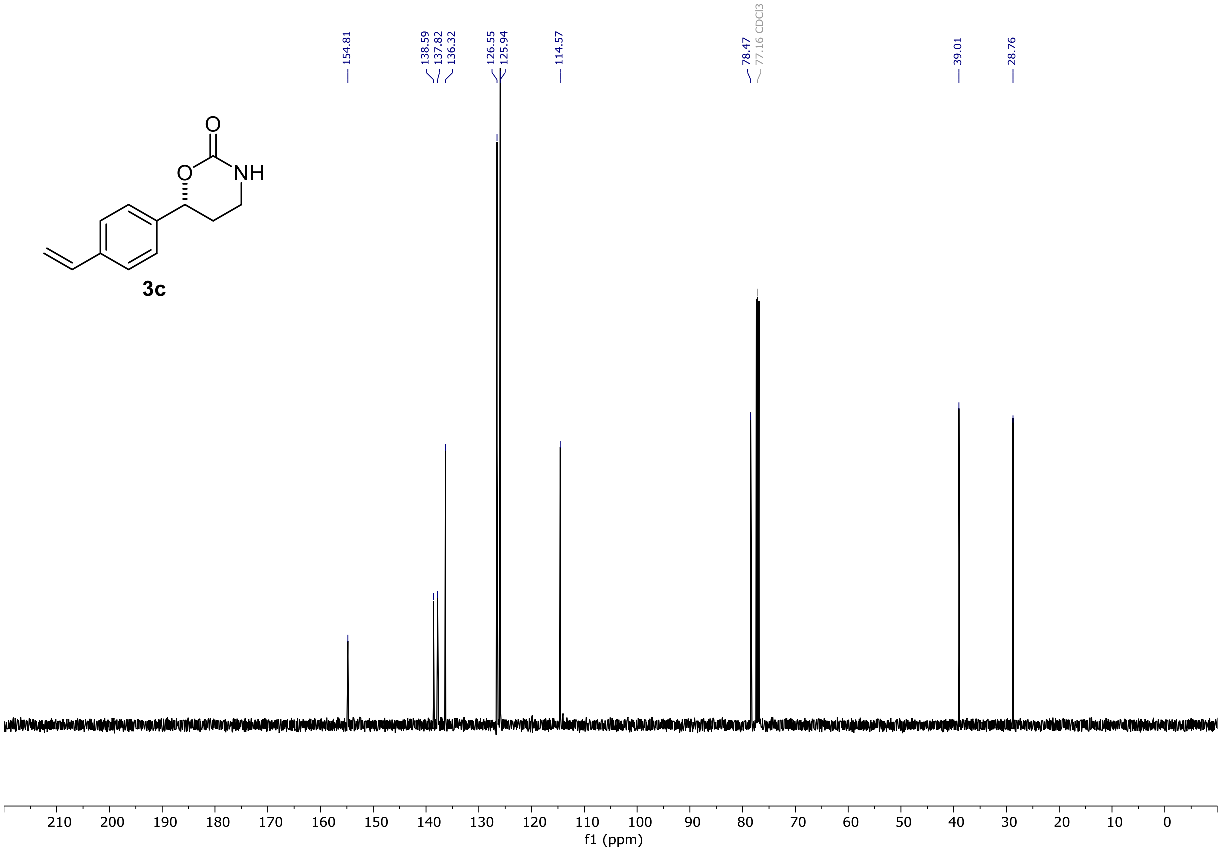

NMR spectra for compound **3c**: ^{1}H (501 MHz) and ^{13}C (126 MHz), in $CDCl_3$.

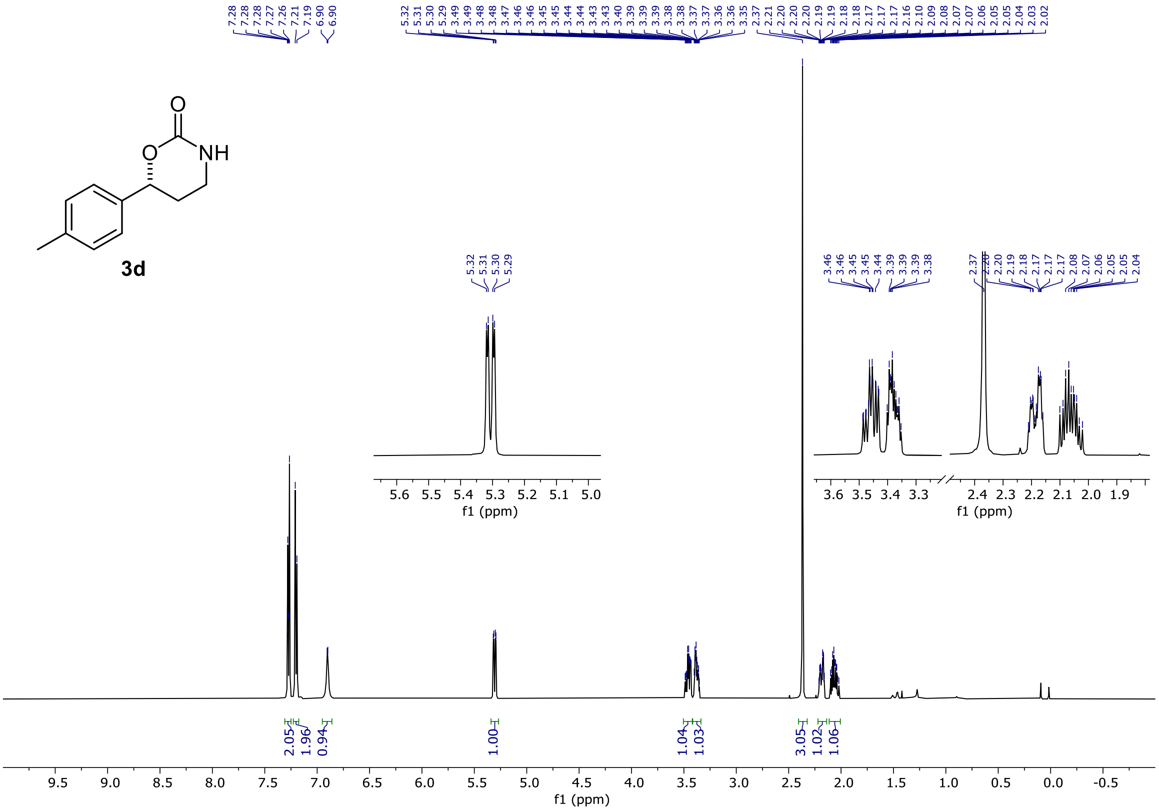

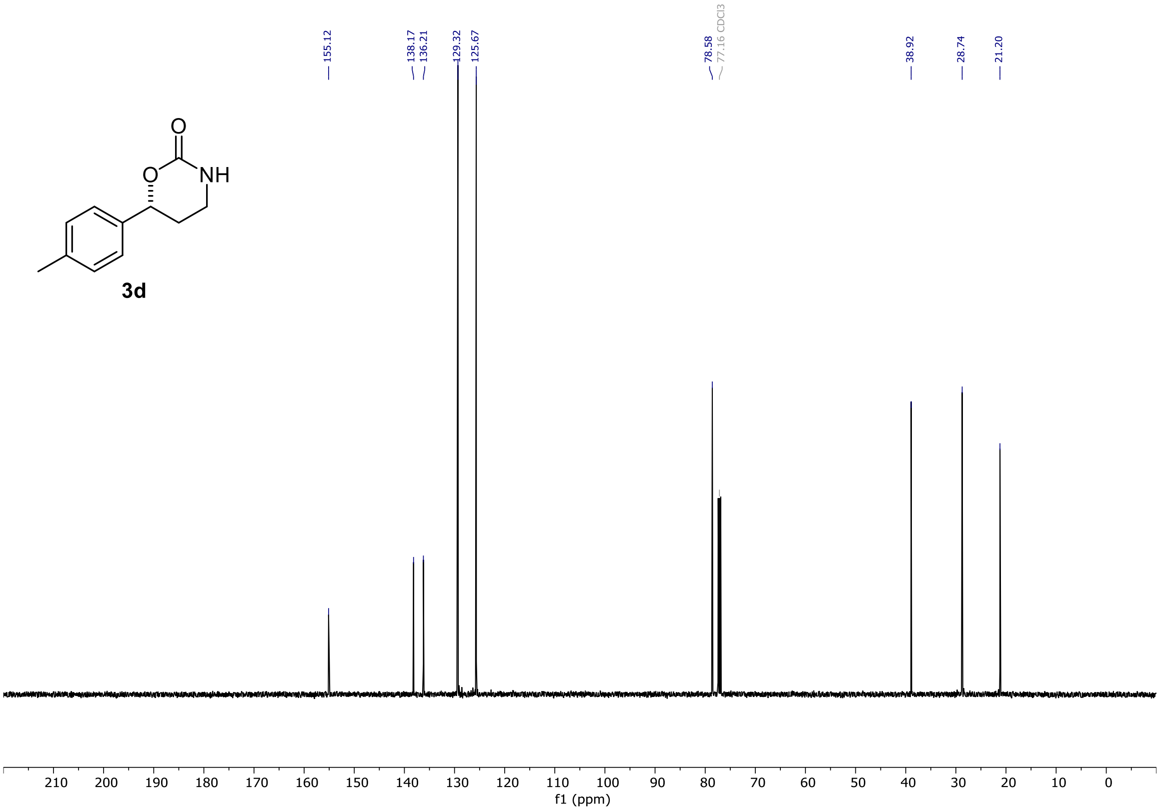

NMR spectra for compound **3d**: ^{1}H (501 MHz) and ^{13}C (126 MHz), in $CDCl_3$.

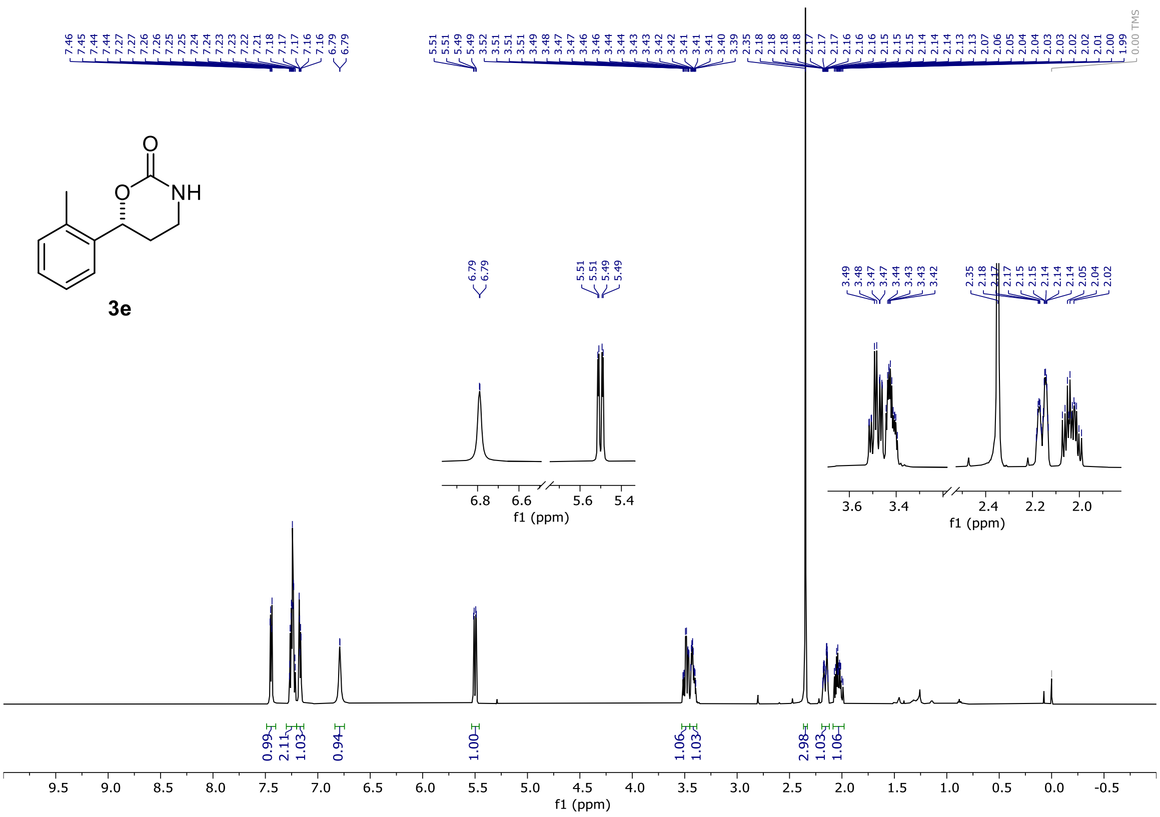

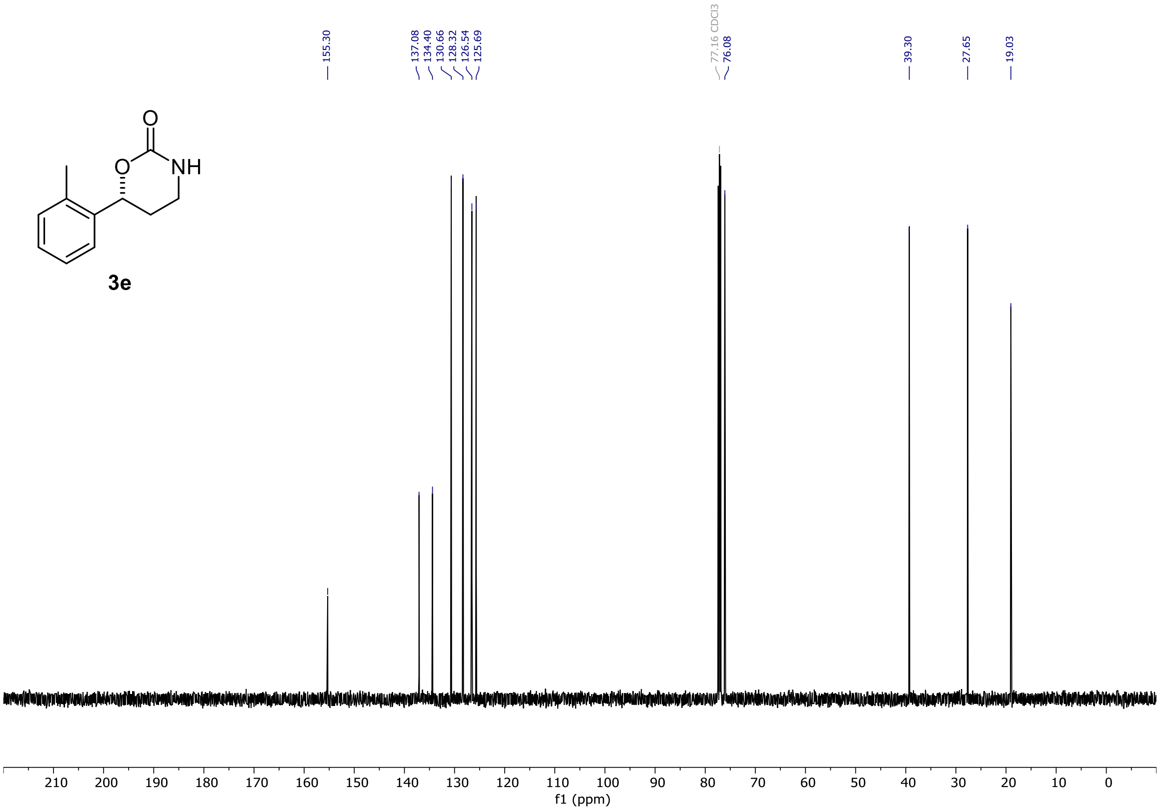

NMR spectra for compound **3e**: ^{1}H (501 MHz) and ^{13}C (126 MHz), in $CDCl_3$.

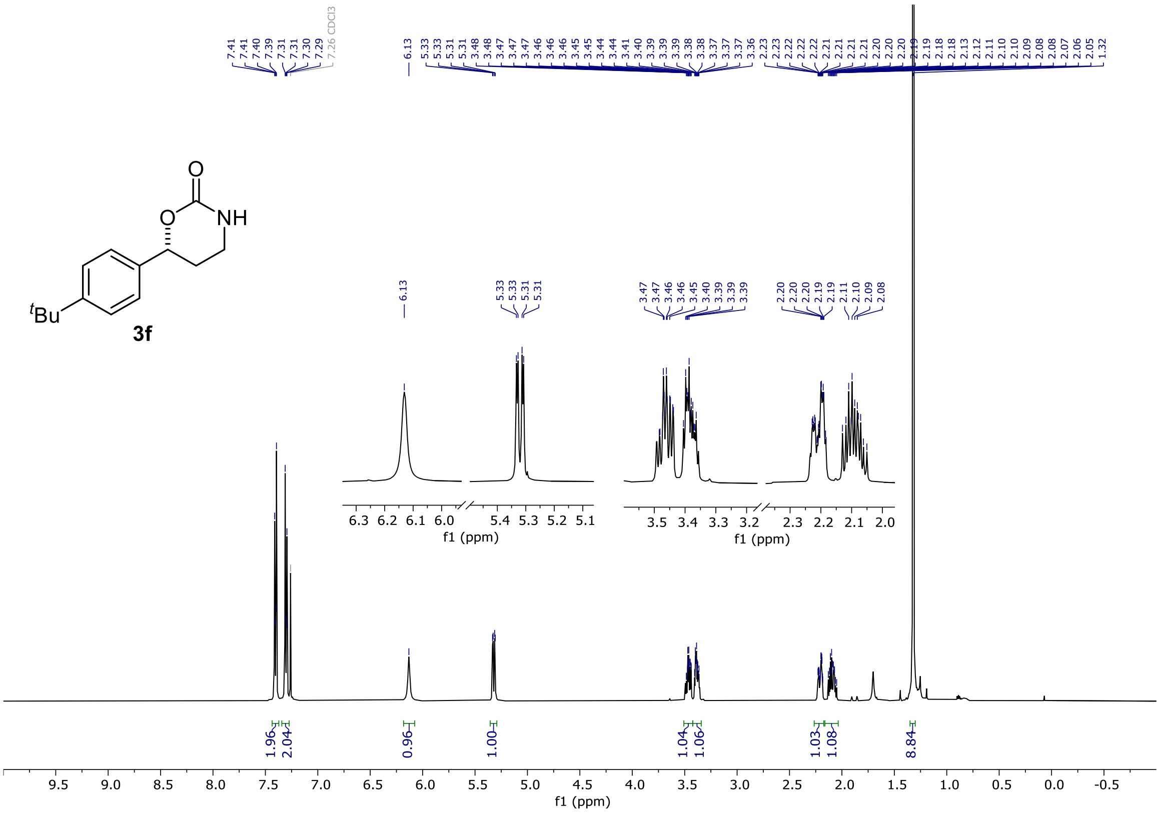

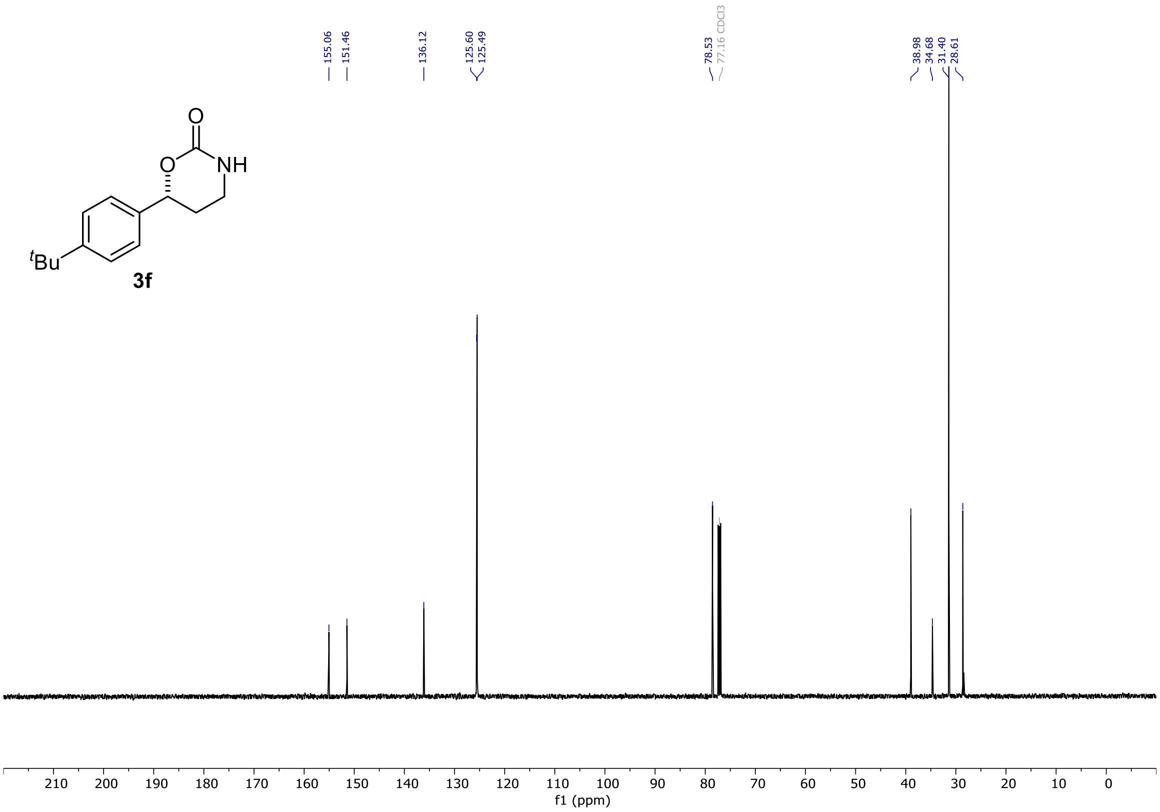

NMR spectra for compound **3f**: ^{1}H (501 MHz) and ^{13}C (126 MHz), in $CDCl_3$.

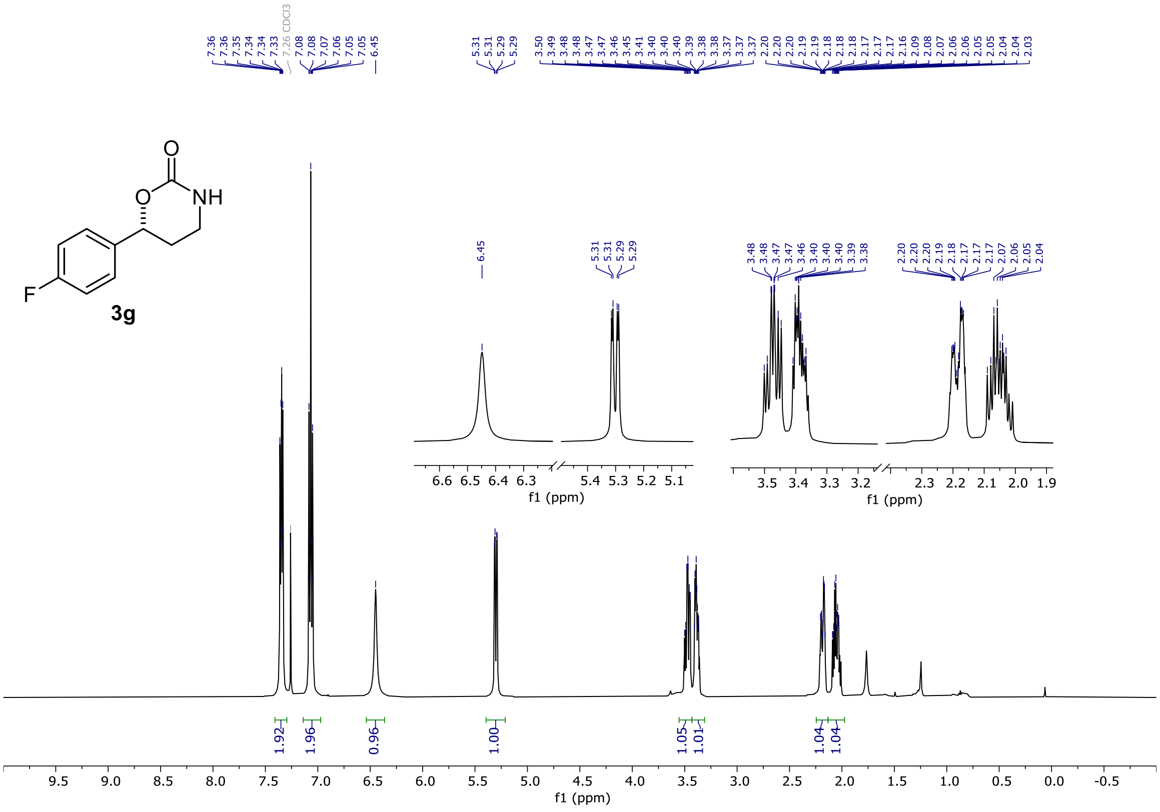
7.36
7.36
7.35
7.34
7.34
7.33
7.26 CDCl3
7.08
7.08
7.07
7.06
7.05
7.05
6.45
5.31
5.31
5.29
5.29
3.50
3.49
3.48
3.48
3.47
3.47
3.46
3.45
3.41
3.40
3.40
3.40
3.39
3.38
3.38
3.37
3.37
3.37
2.20
2.20
2.20
2.19
2.19
2.18
2.18
2.18
2.17
2.17
2.16
2.09
2.08
2.07
2.06
2.06
2.05
2.05
2.04
2.04
2.03
O
O
NH
F
1.92
1.96
0.96
1.00
1.05
1.01
1.04
1.04
f1 (ppm)
3g

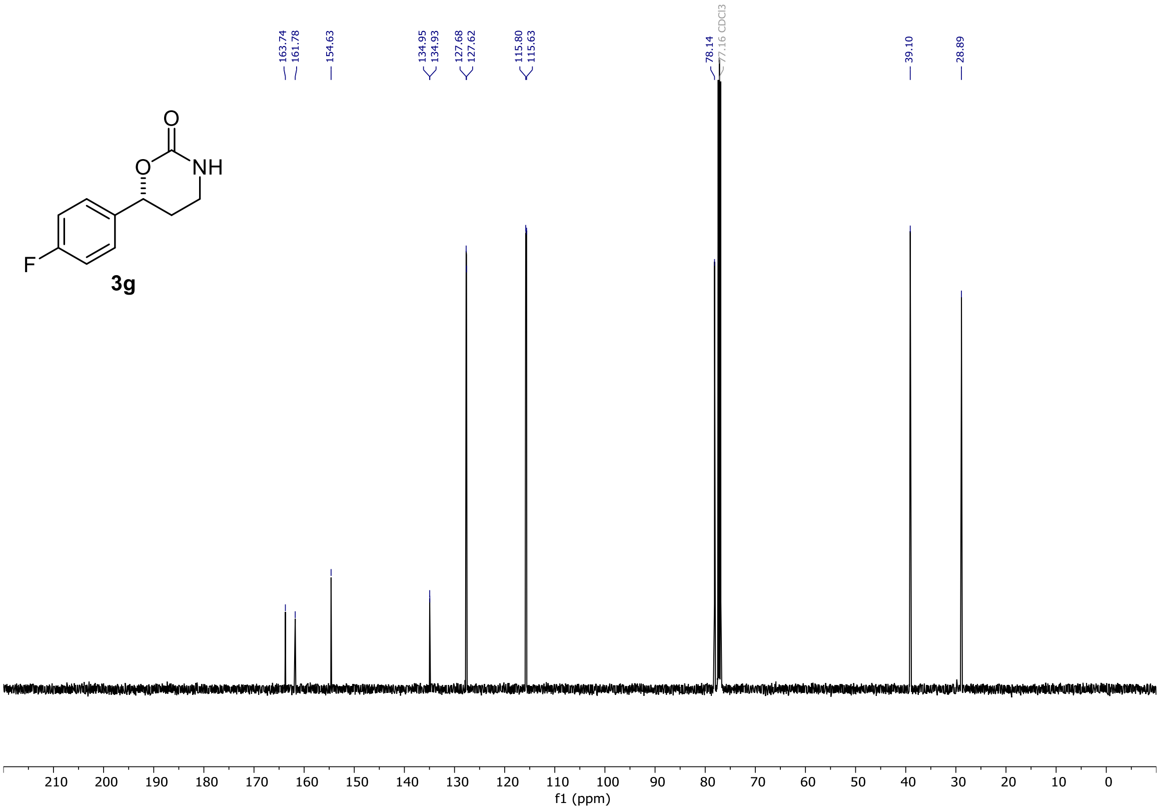
163.74
161.78
154.63
134.95
134.93
127.68
127.62
115.80
115.63
78.14
77.16 CDCl3
39.10
28.89
O
O
NH
F
f1 (ppm)
3g

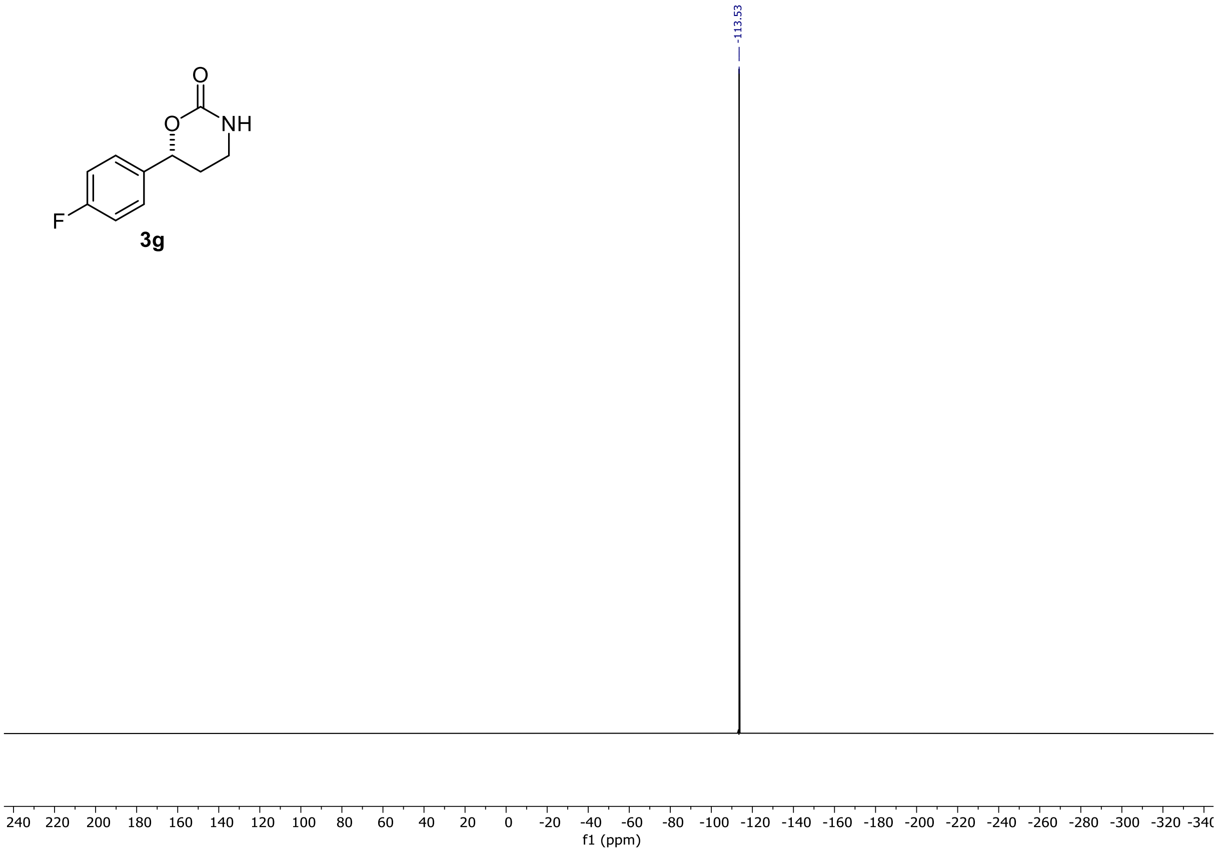

NMR spectra for compound **3g**: ^{1}H (501 MHz), ^{13}C (126 MHz), and ^{19}F (471 MHz), in $CDCl_3$.

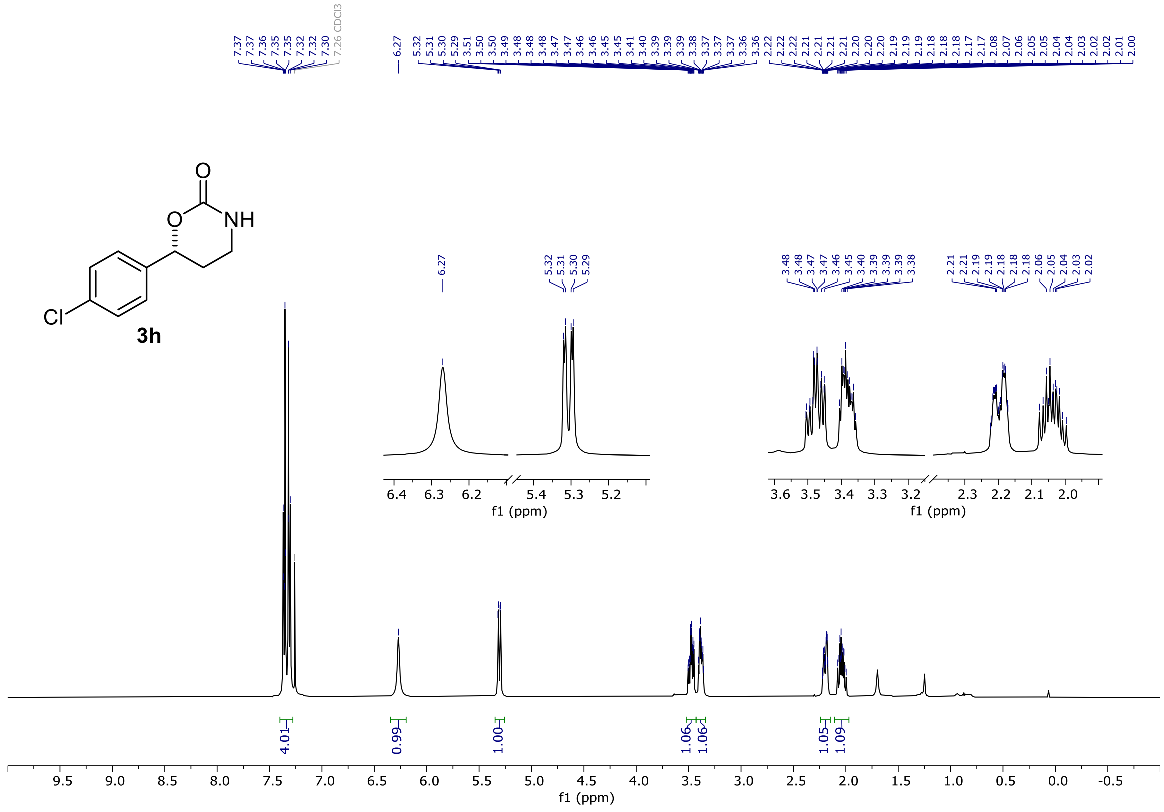

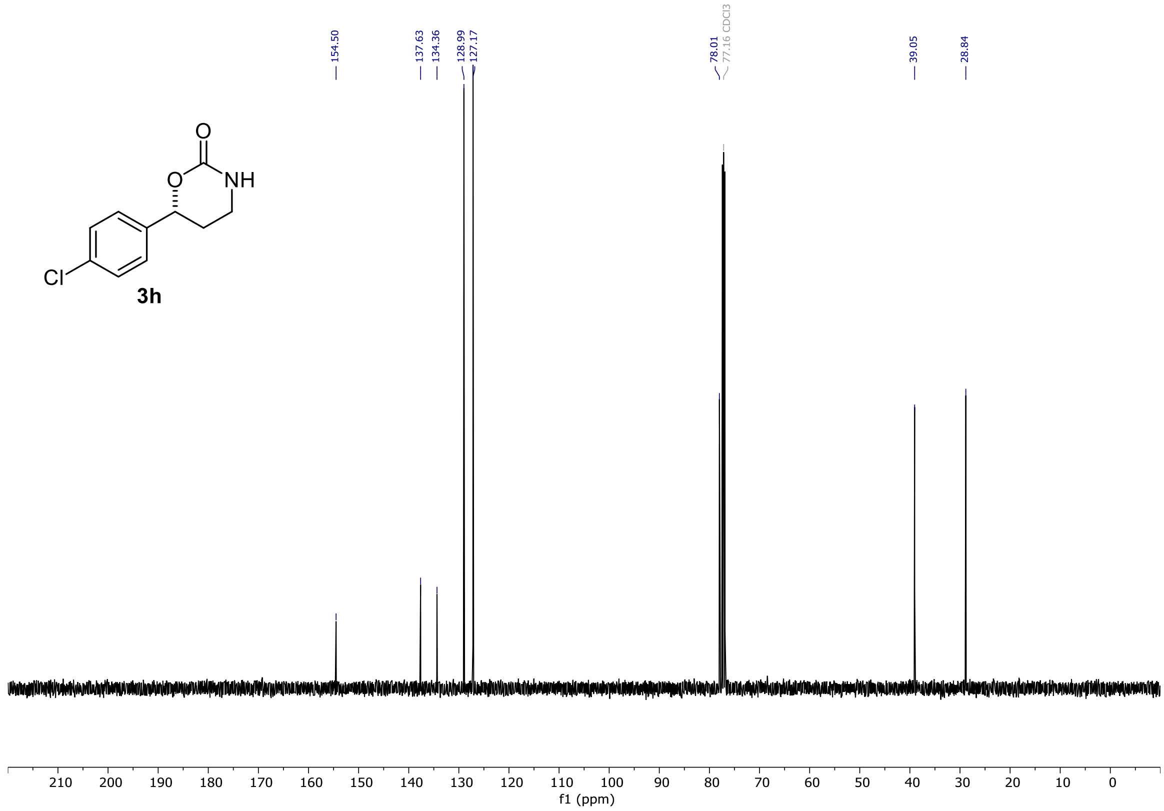

NMR spectra for compound **3h**: ^{1}H (501 MHz) and ^{13}C (126 MHz), in $CDCl_3$.

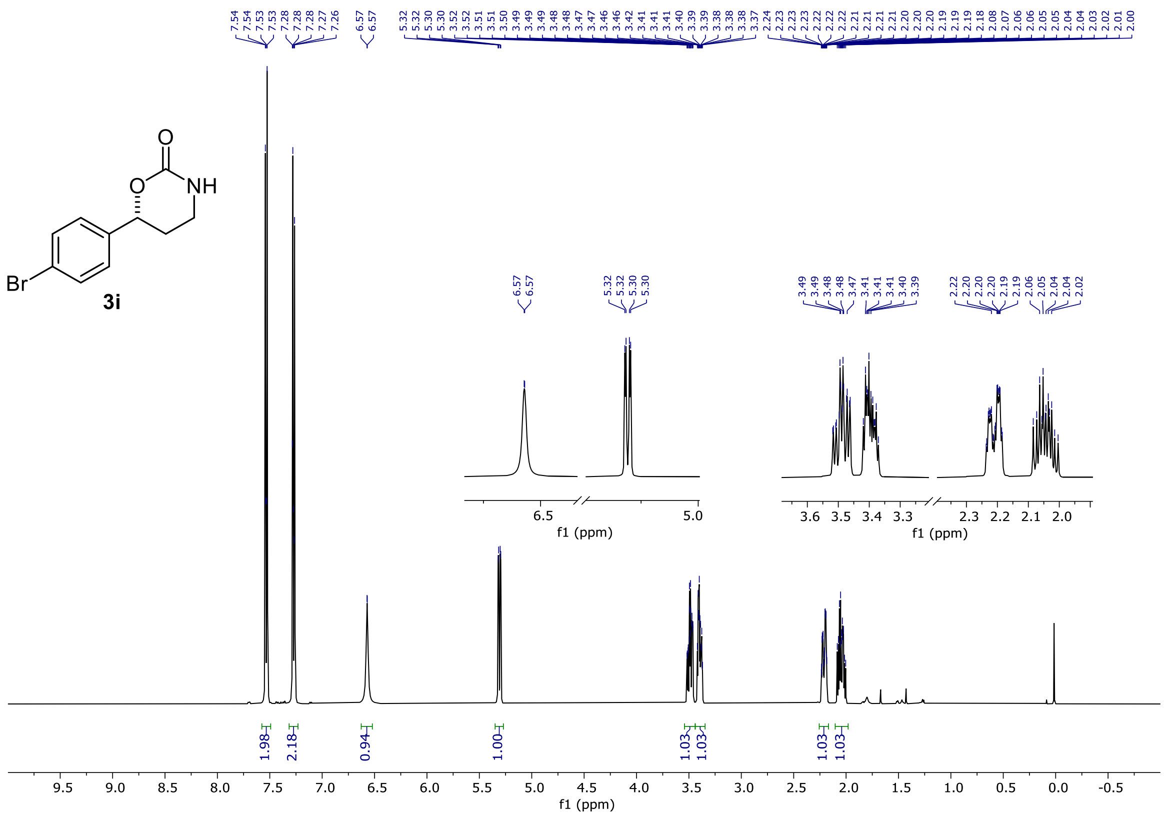

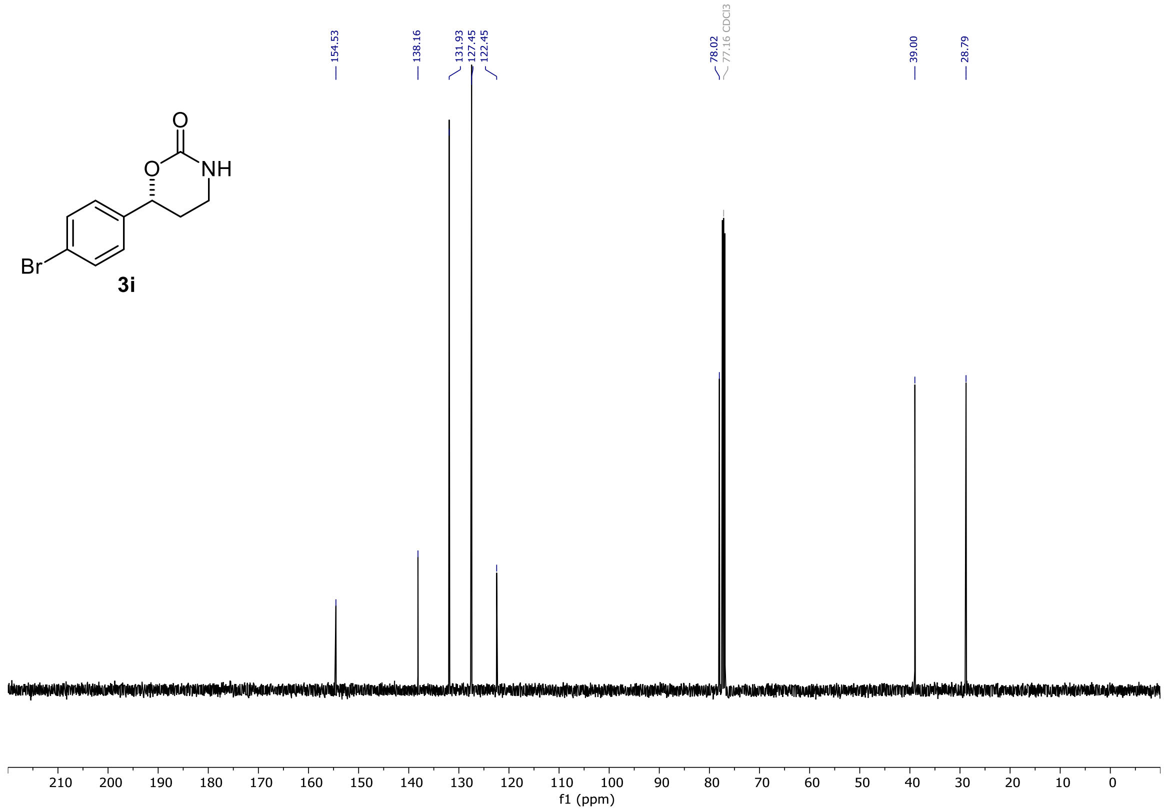

NMR spectra for compound **3i**: ^{1}H (501 MHz) and ^{13}C (126 MHz), in $CDCl_3$.

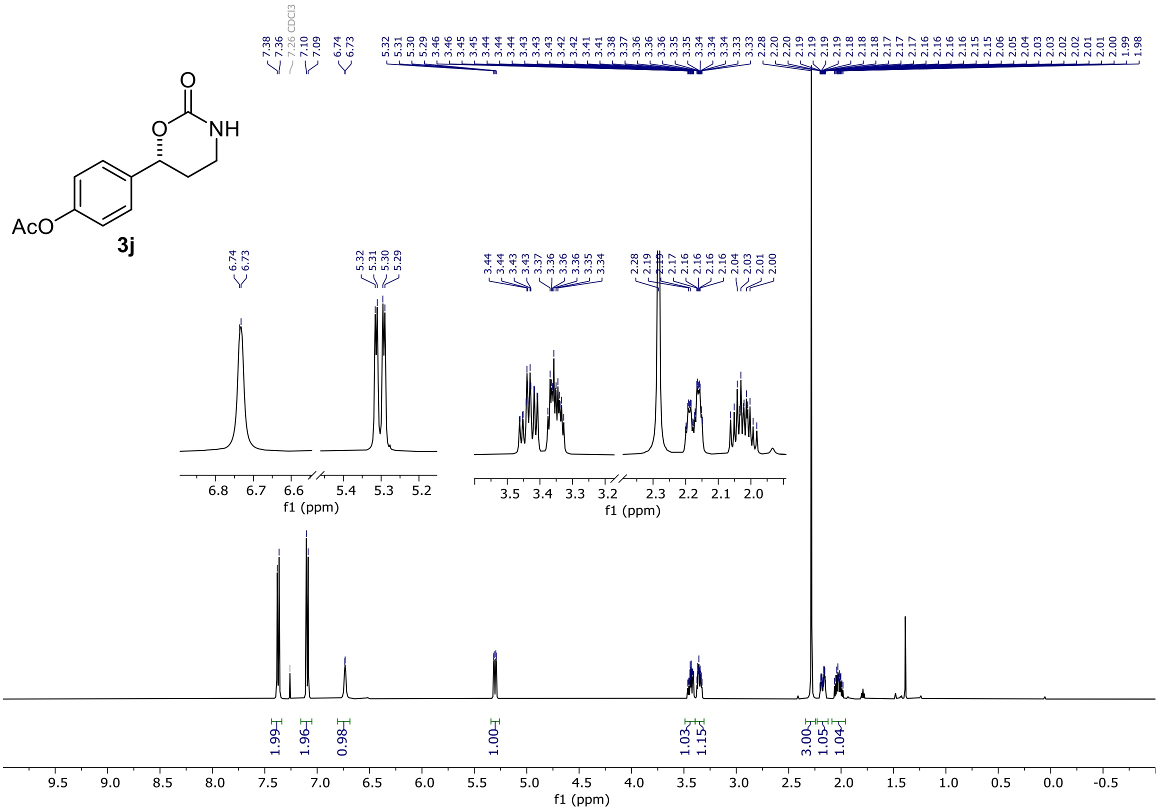

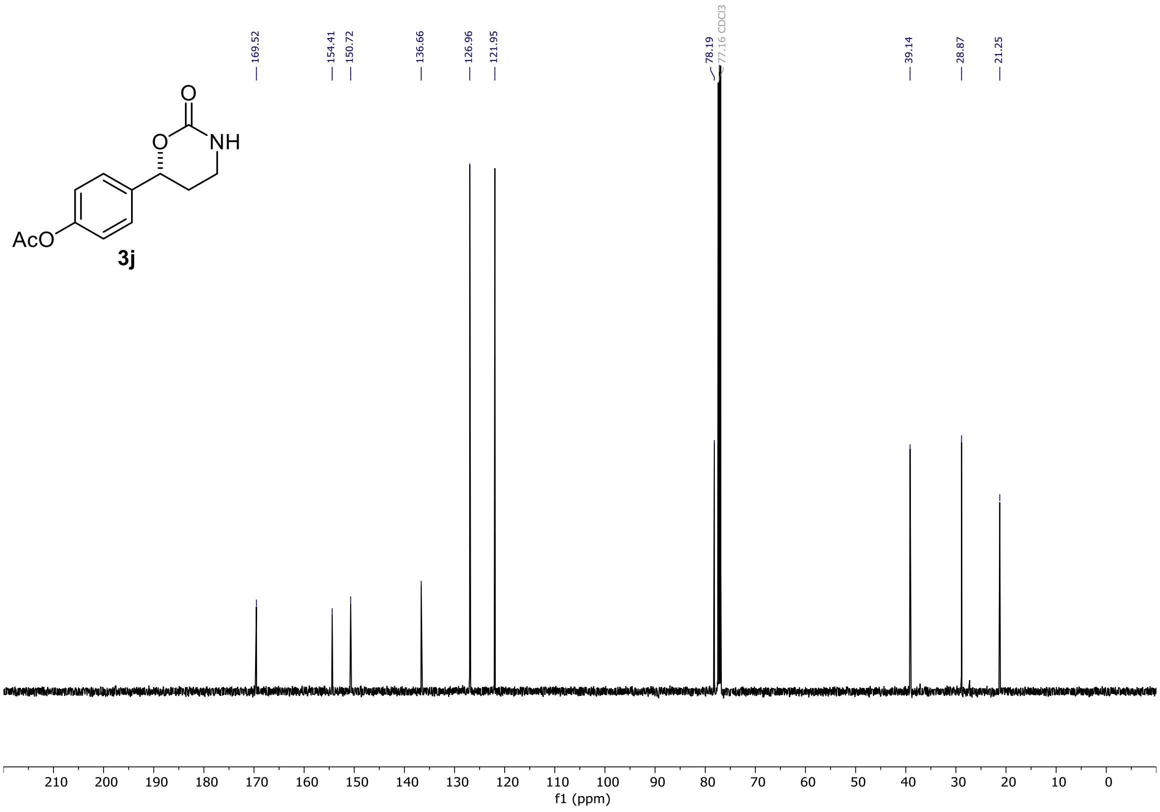

NMR spectra for compound **3j**: ^{1}H (501 MHz) and ^{13}C (126 MHz), in $CDCl_3$.

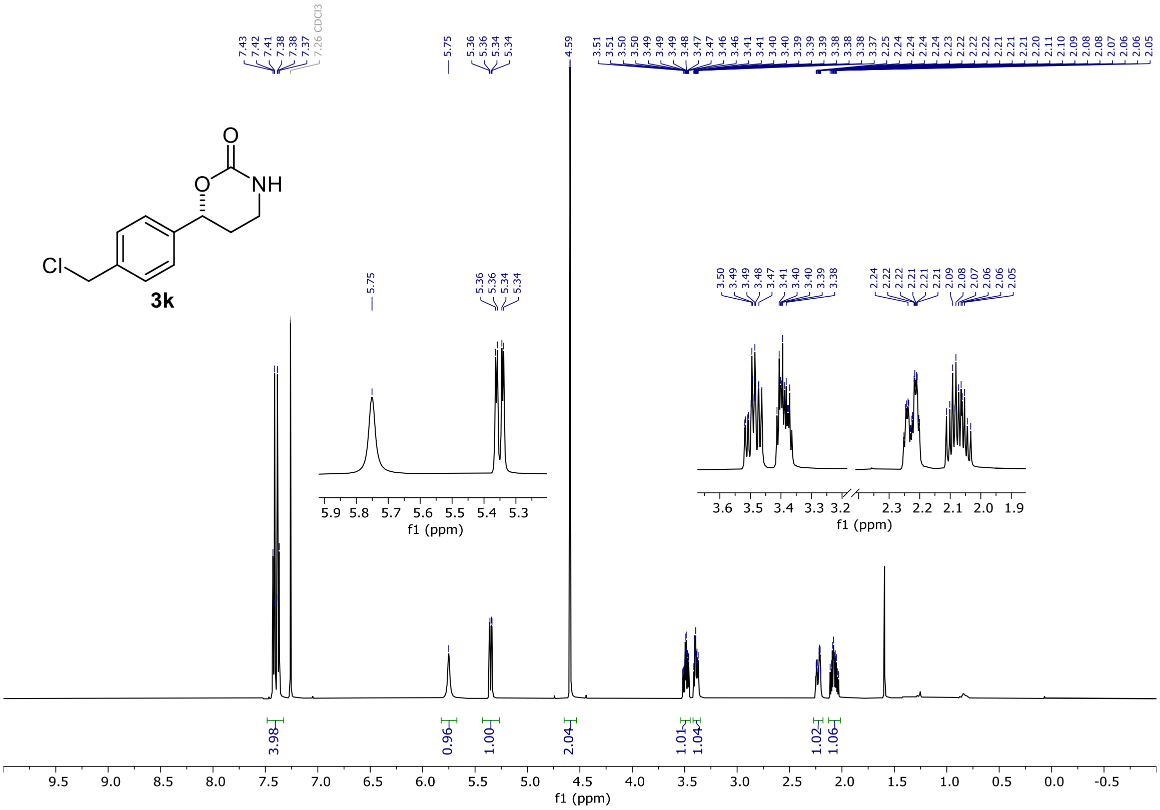

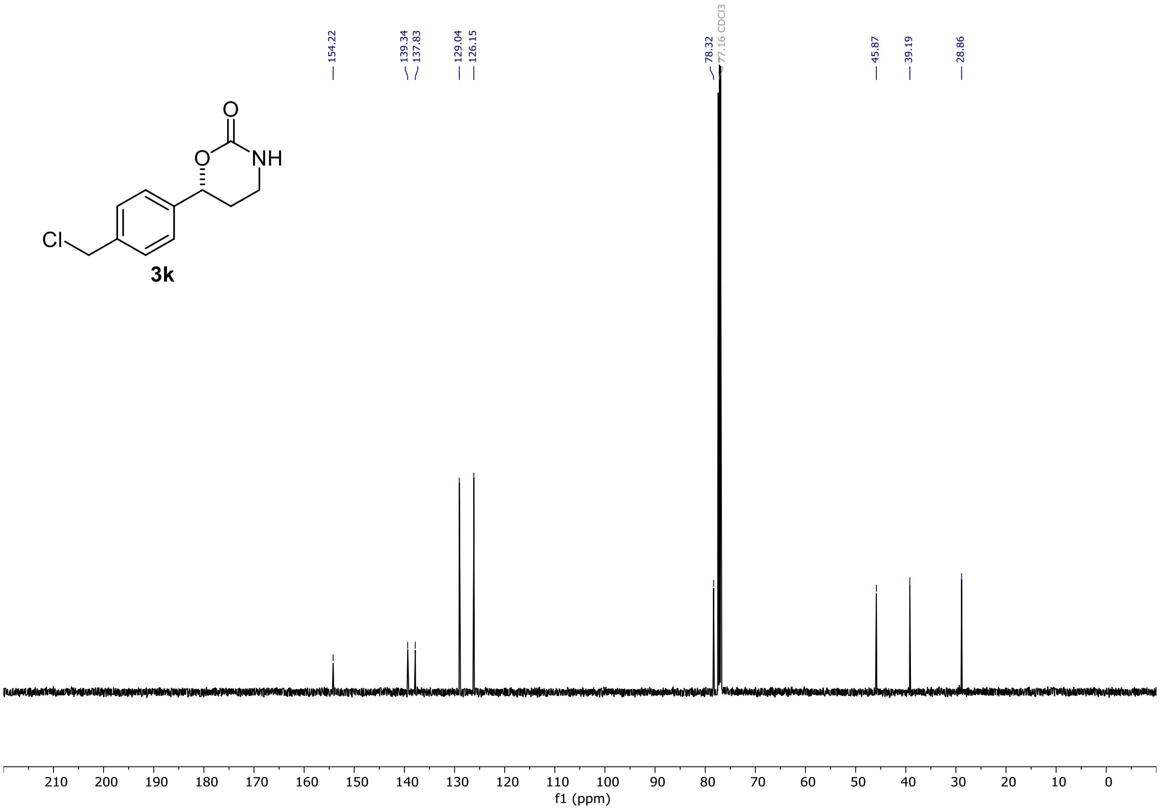

NMR spectra for compound **3k**: ^{1}H (501 MHz) and ^{13}C (126 MHz), in $CDCl_3$.

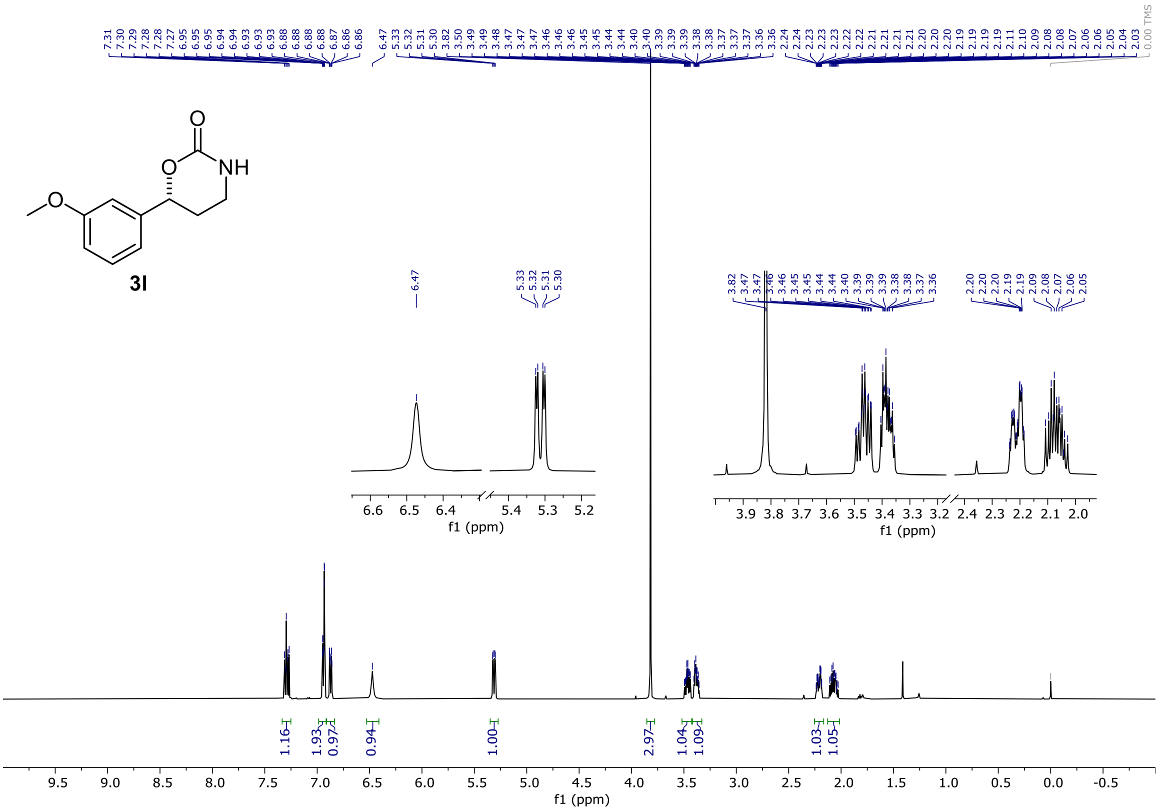

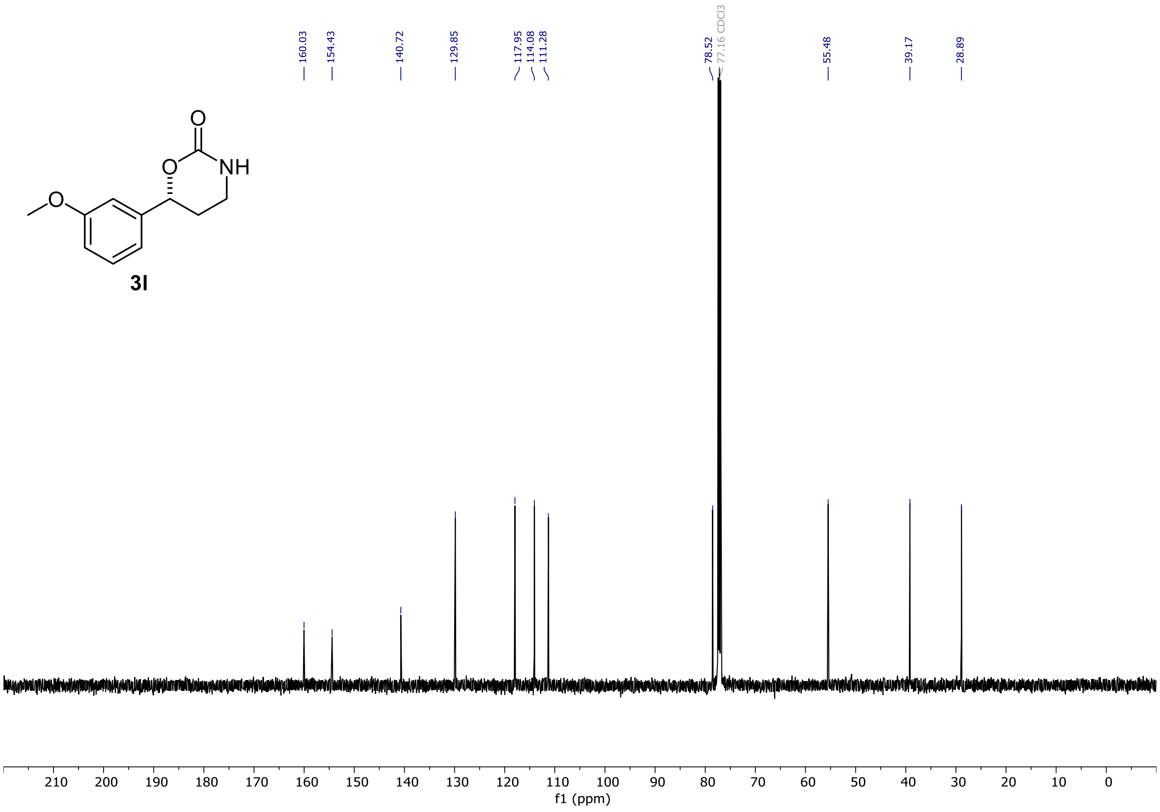

NMR spectra for compound **3l**: ^{1}H (501 MHz) and ^{13}C (126 MHz), in $CDCl_3$.

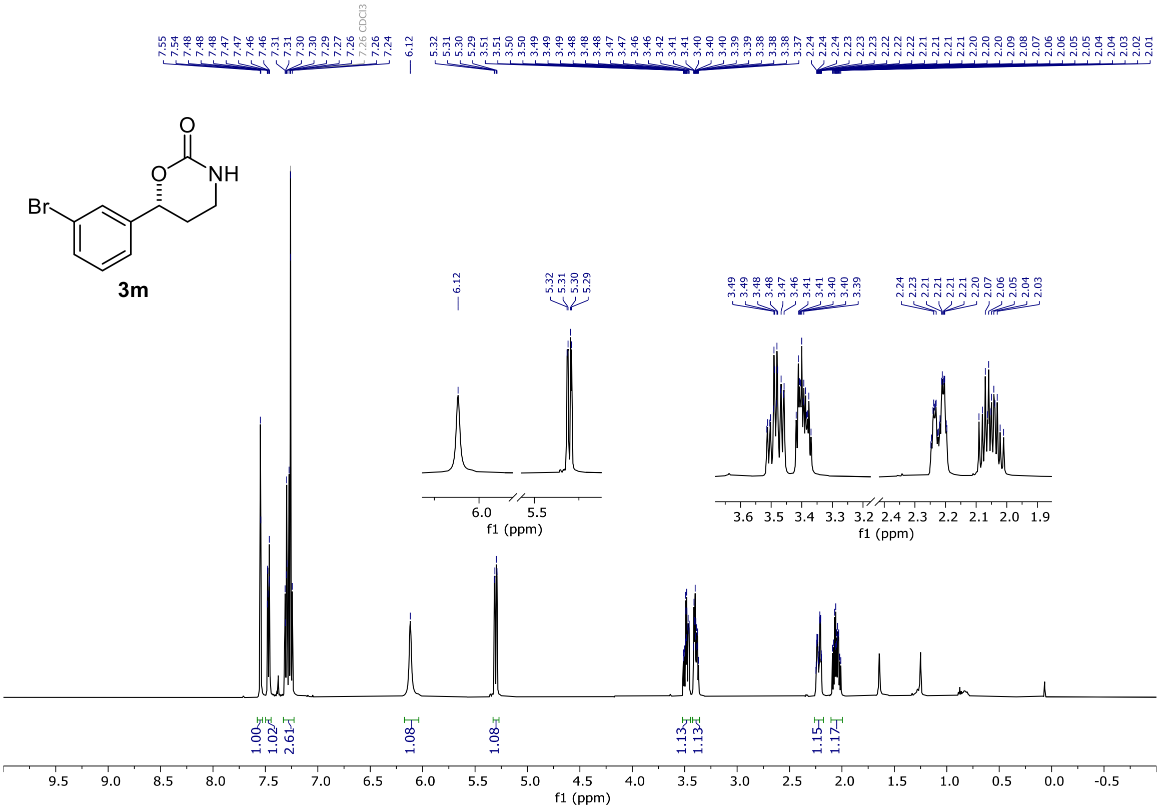

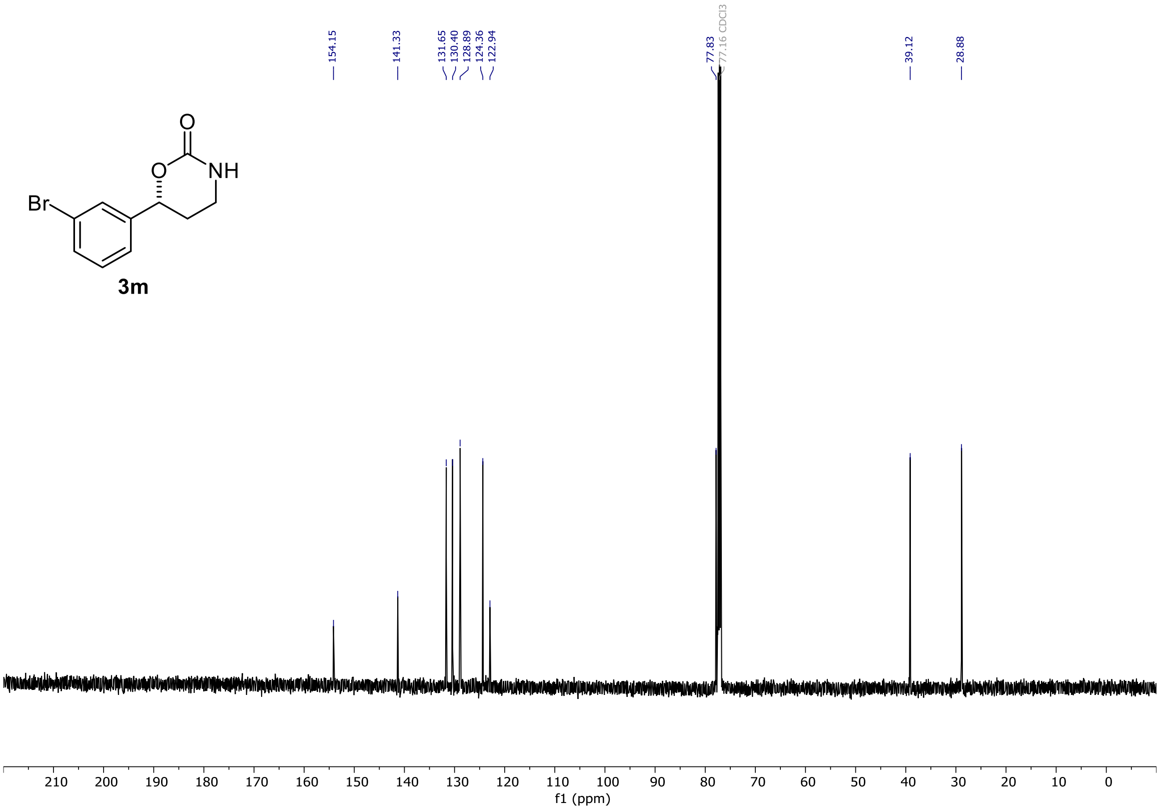

NMR spectra for compound **3m**: ^{1}H (501 MHz) and ^{13}C (126 MHz), in $CDCl_3$.

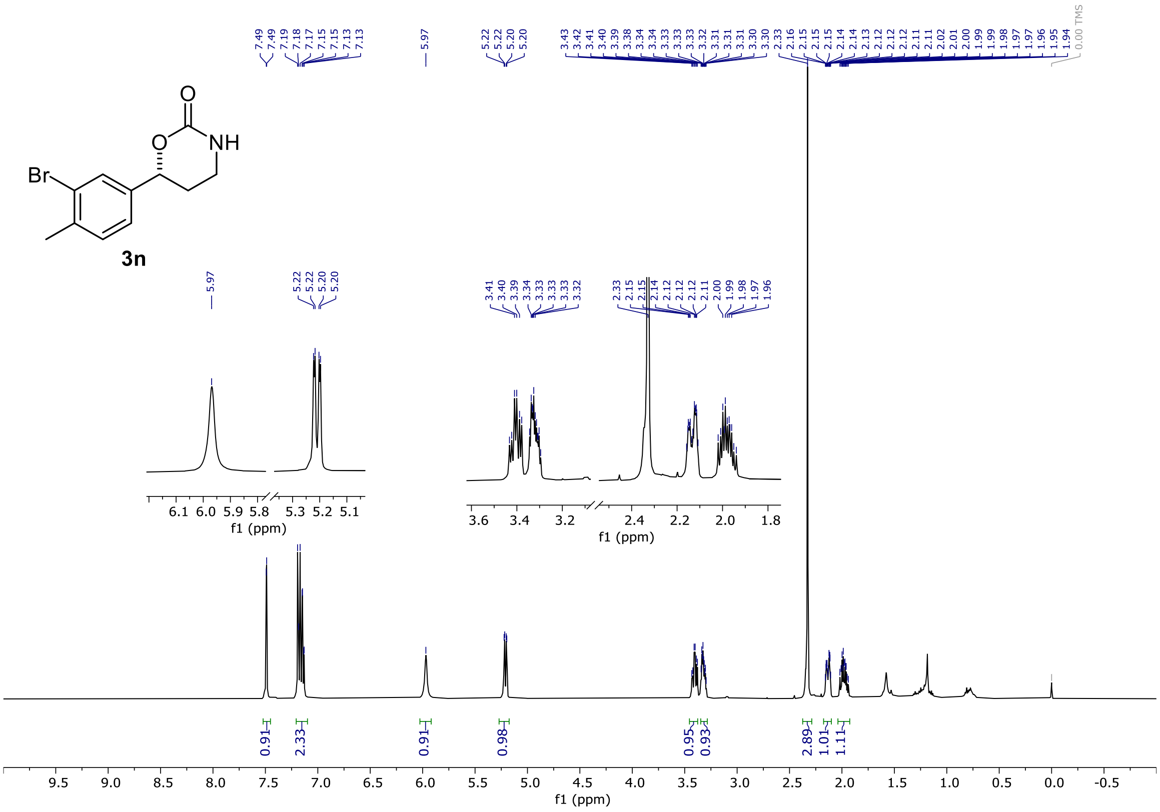

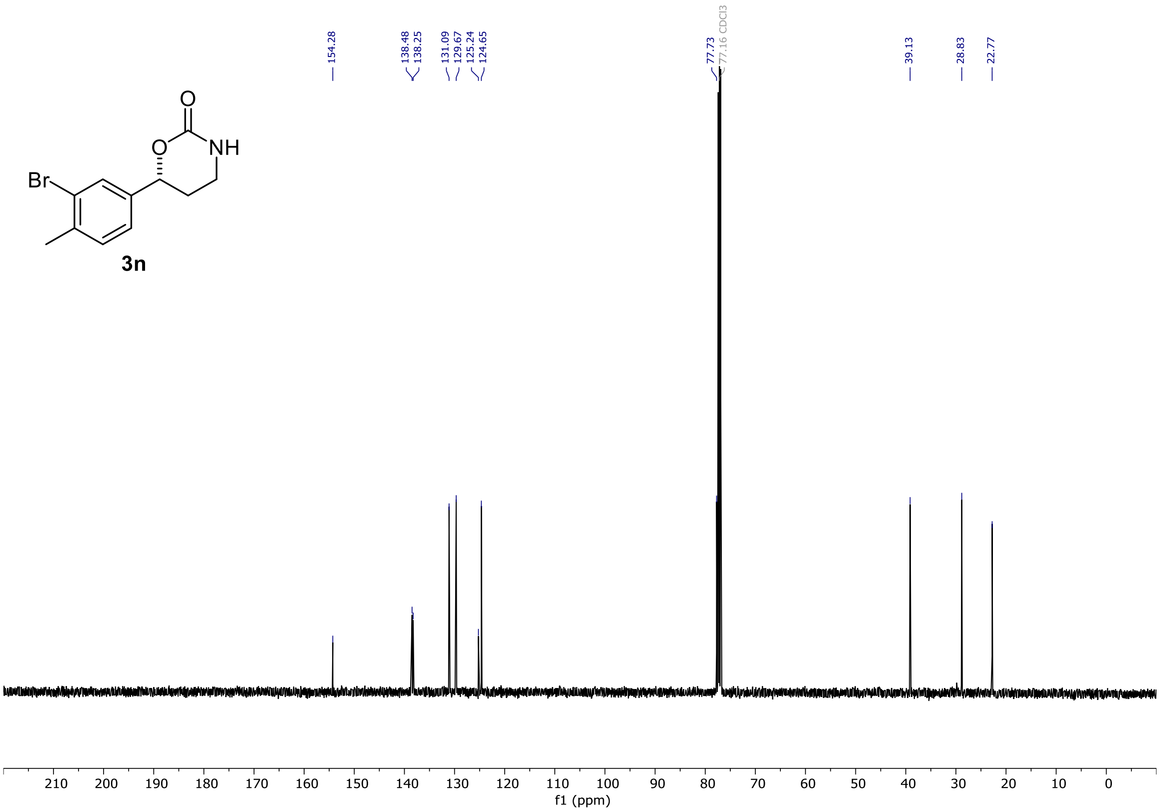

NMR spectra for compound **3n**: ^{1}H (501 MHz) and ^{13}C (126 MHz), in $CDCl_3$.

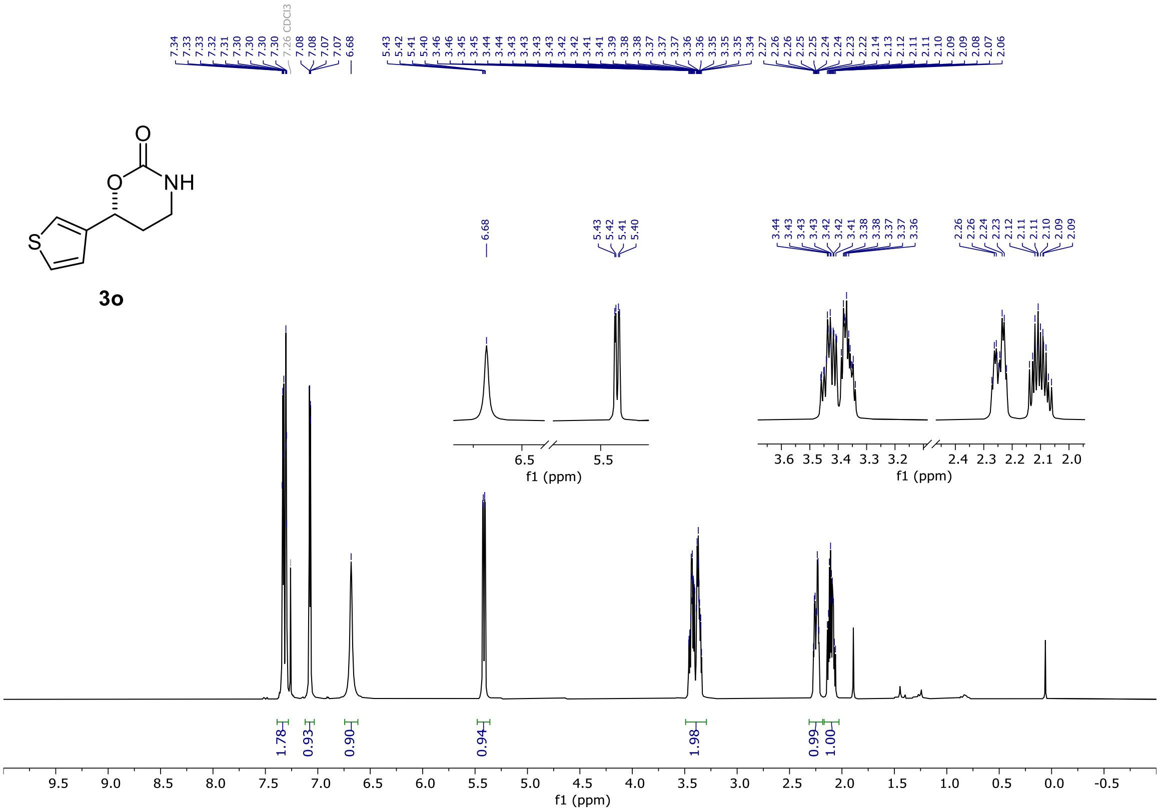

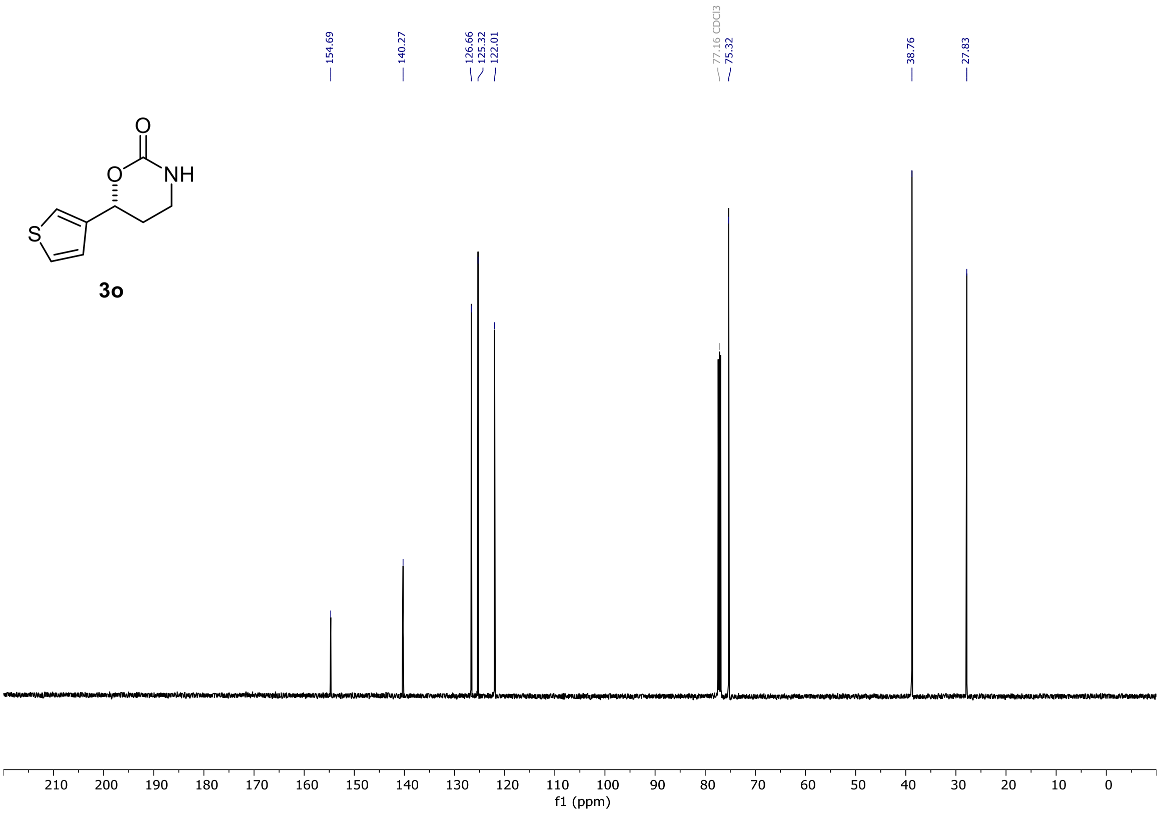

NMR spectra for compound **3o**: ^{1}H (501 MHz) and ^{13}C (126 MHz), in $CDCl_3$.

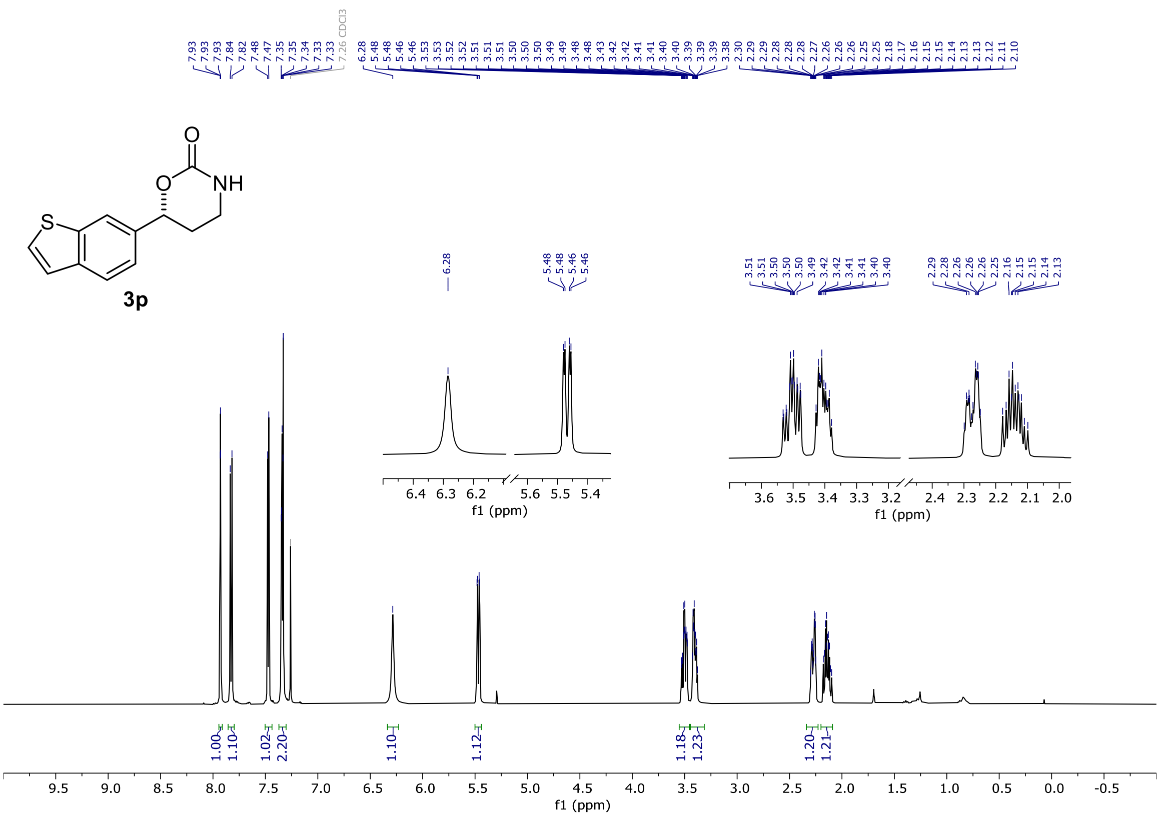

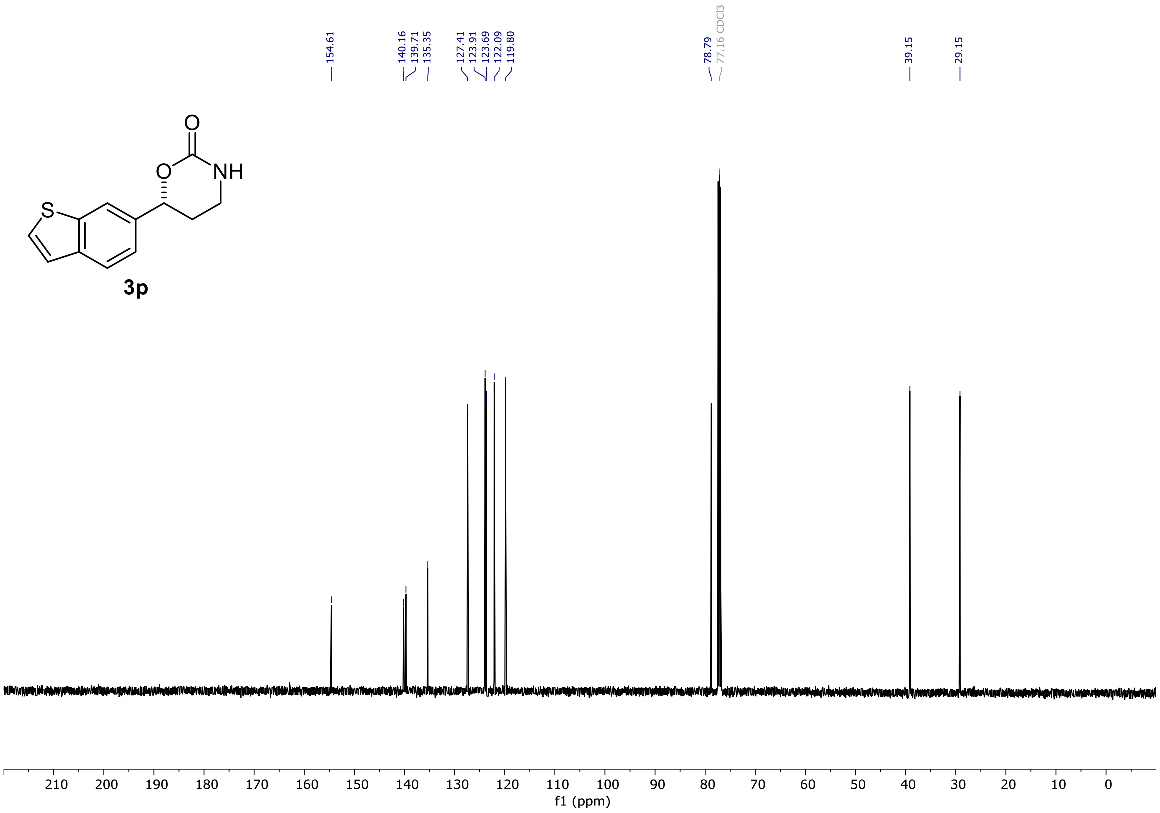

NMR spectra for compound **3p**: ^{1}H (501 MHz) and ^{13}C (126 MHz), in $CDCl_3$.

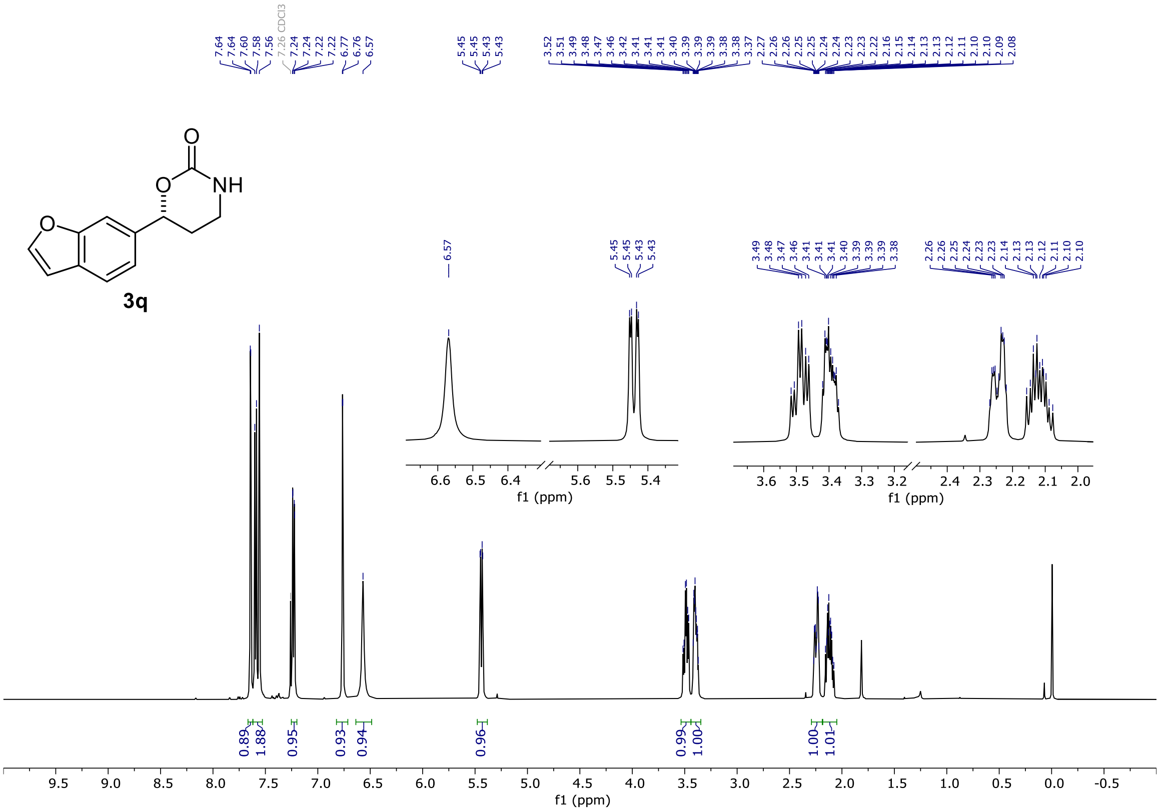

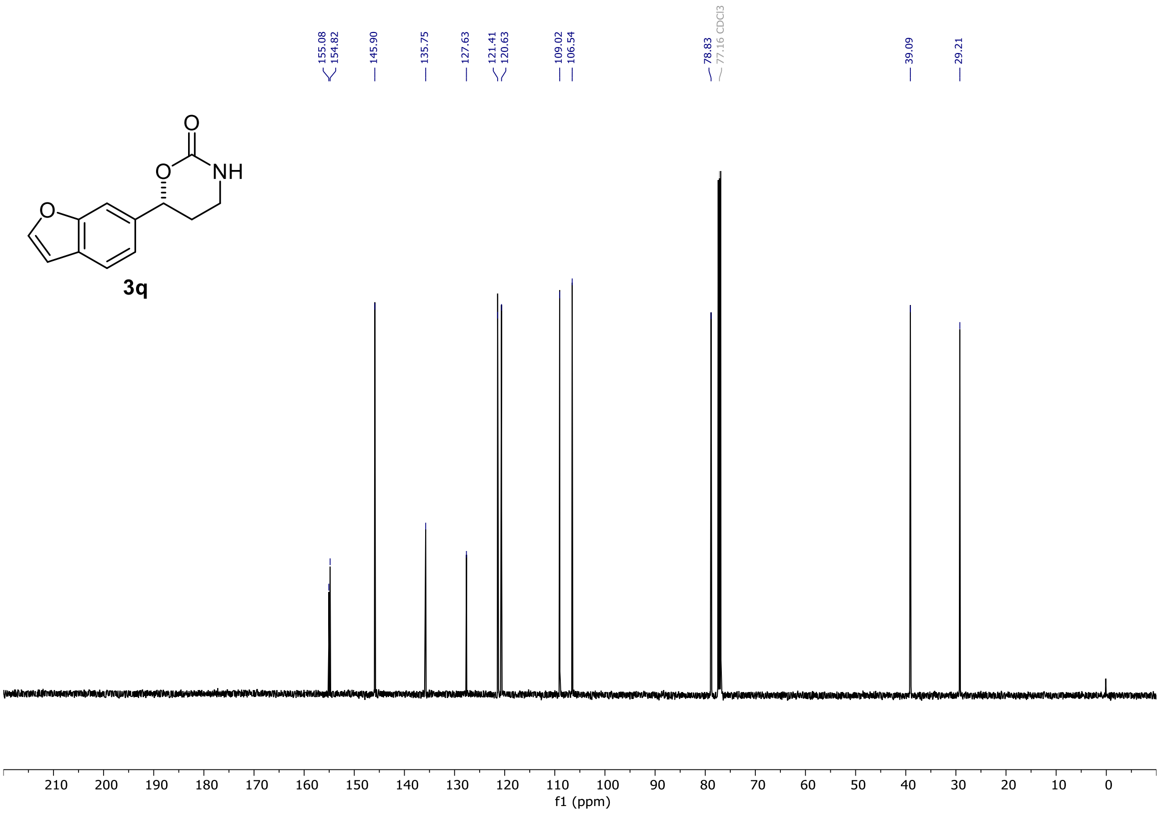

NMR spectra for compound **3q**: ^{1}H (501 MHz) and ^{13}C (126 MHz), in $CDCl_3$.

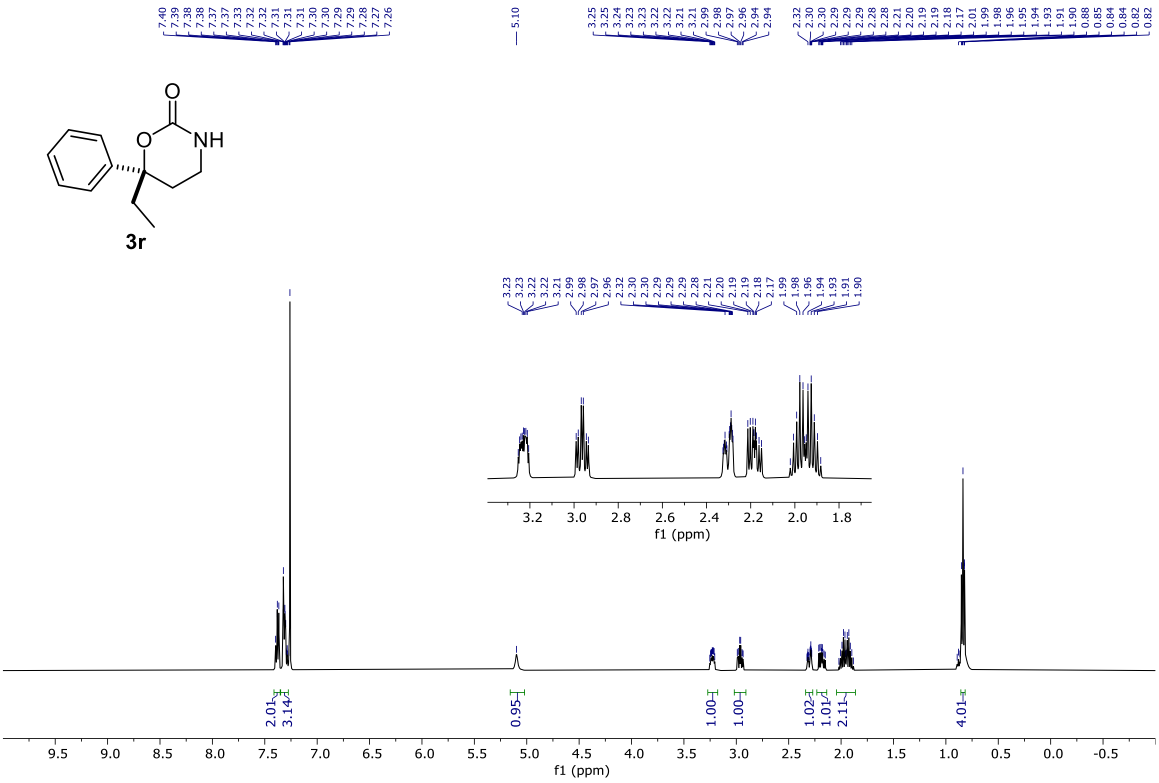

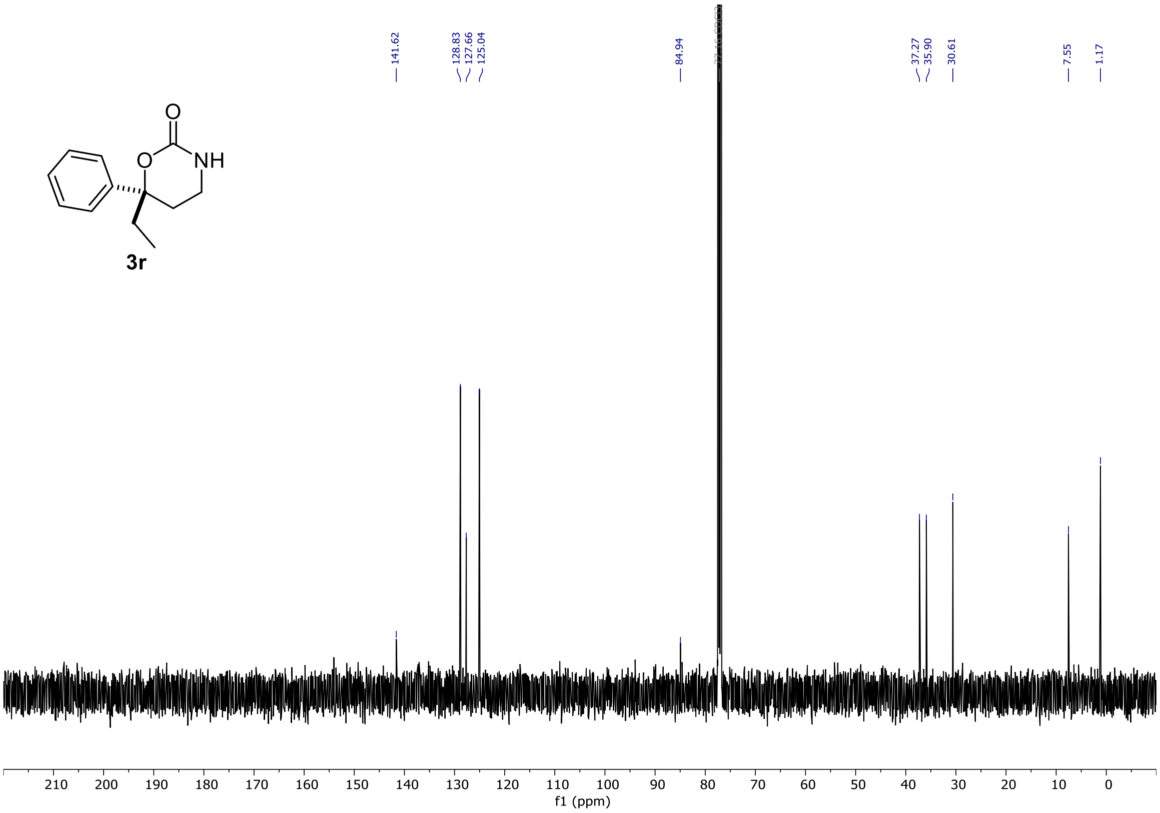

NMR spectra for compound **3r**: ^{1}H (501 MHz) and ^{13}C (126 MHz), in $CDCl_3$.

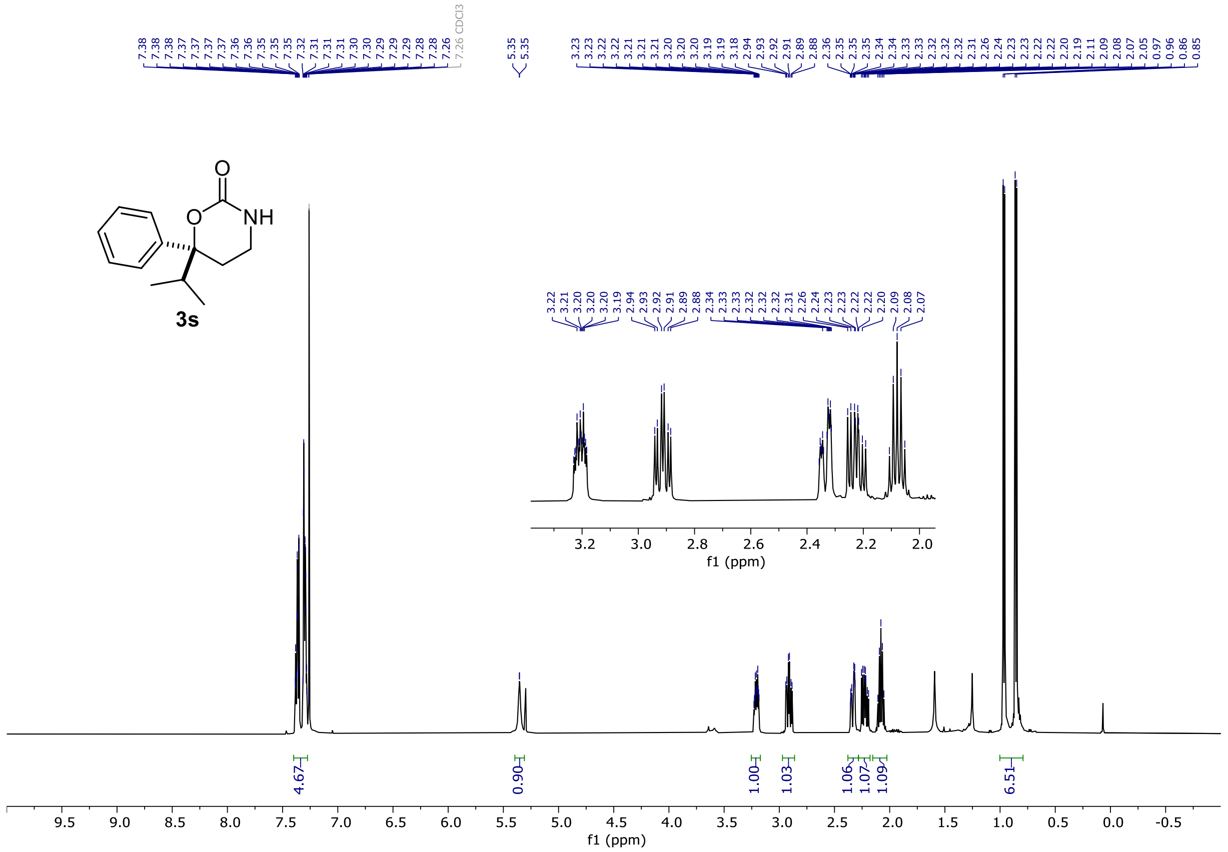

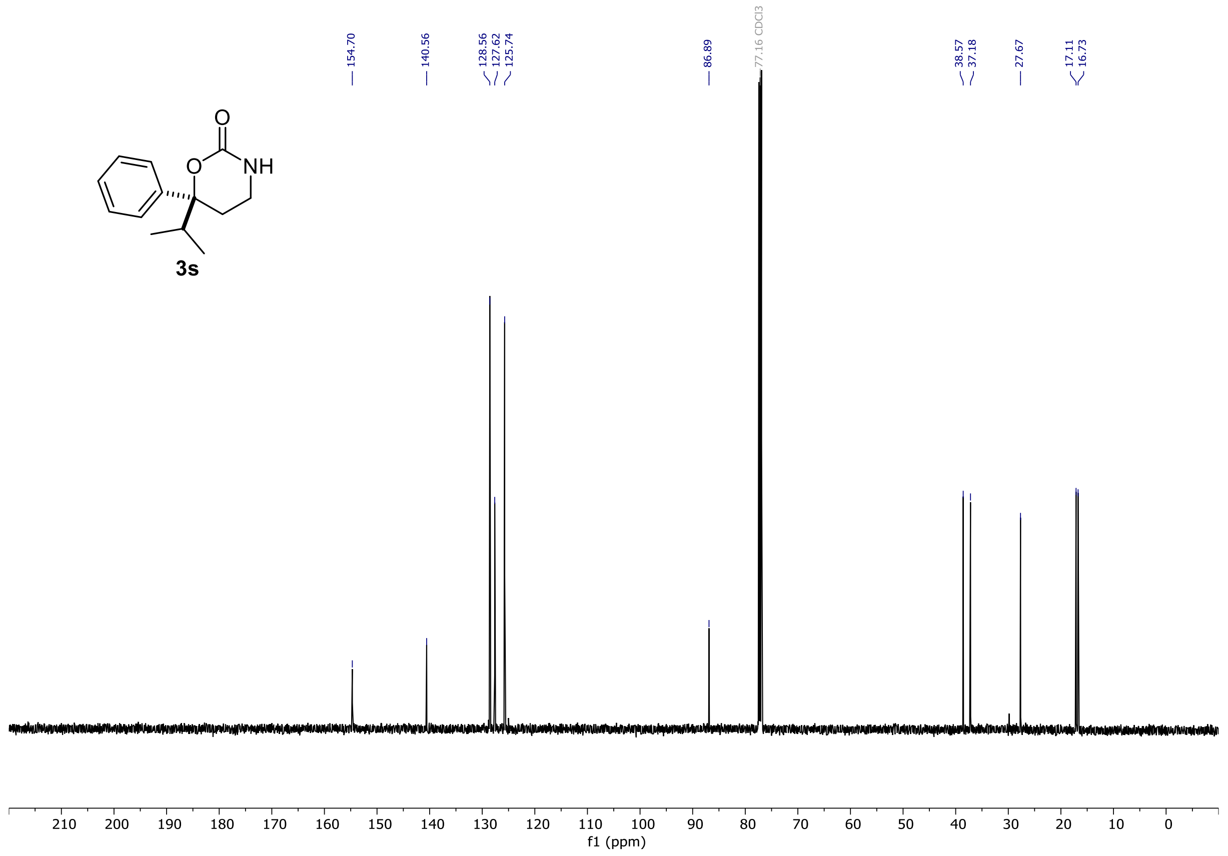

NMR spectra for compound **3s**: ^{1}H (501 MHz) and ^{13}C (126 MHz), in $CDCl_3$.

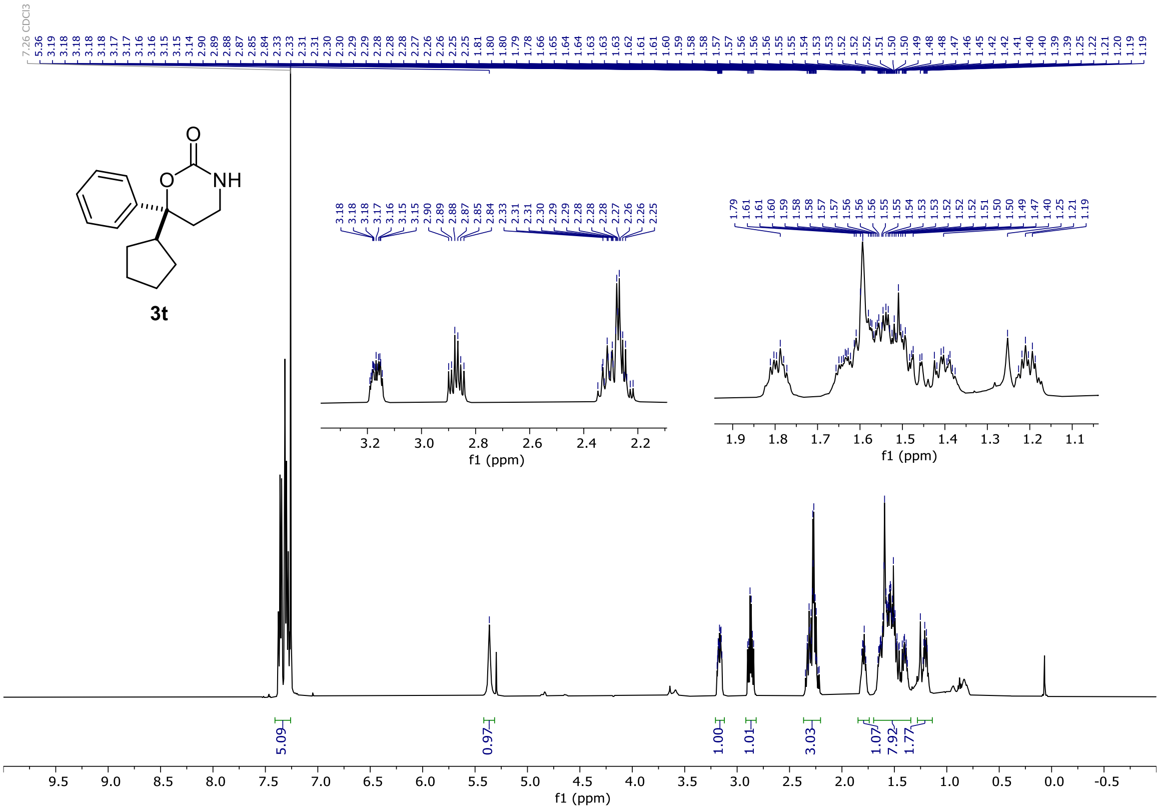

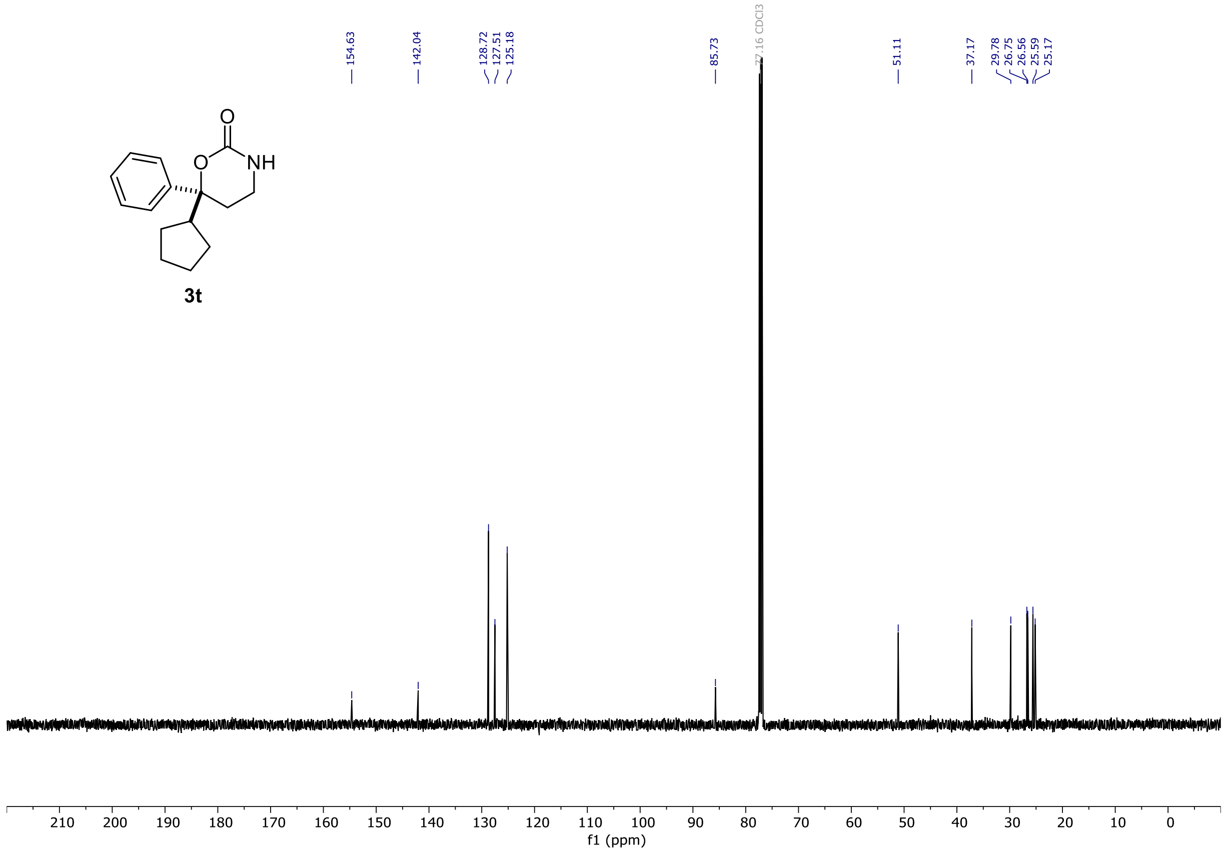

NMR spectra for compound **3t**: ^{1}H (501 MHz) and ^{13}C (126 MHz), in $CDCl_3$.

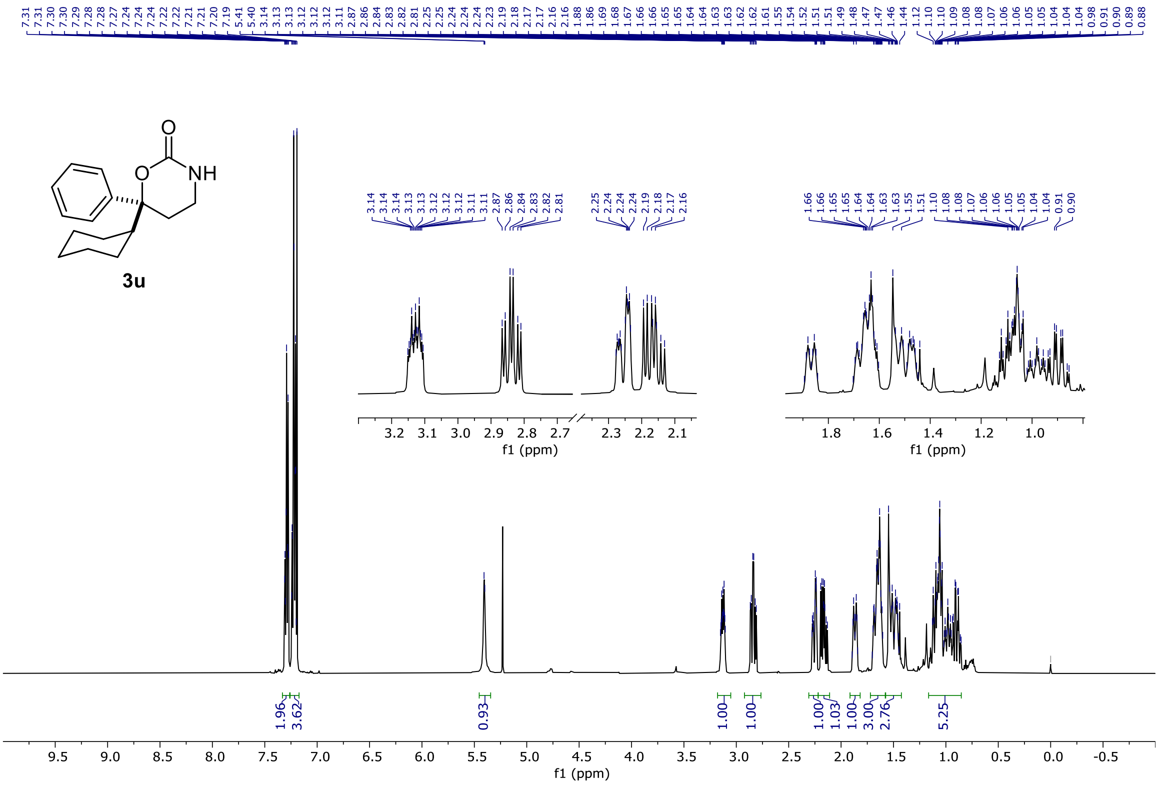

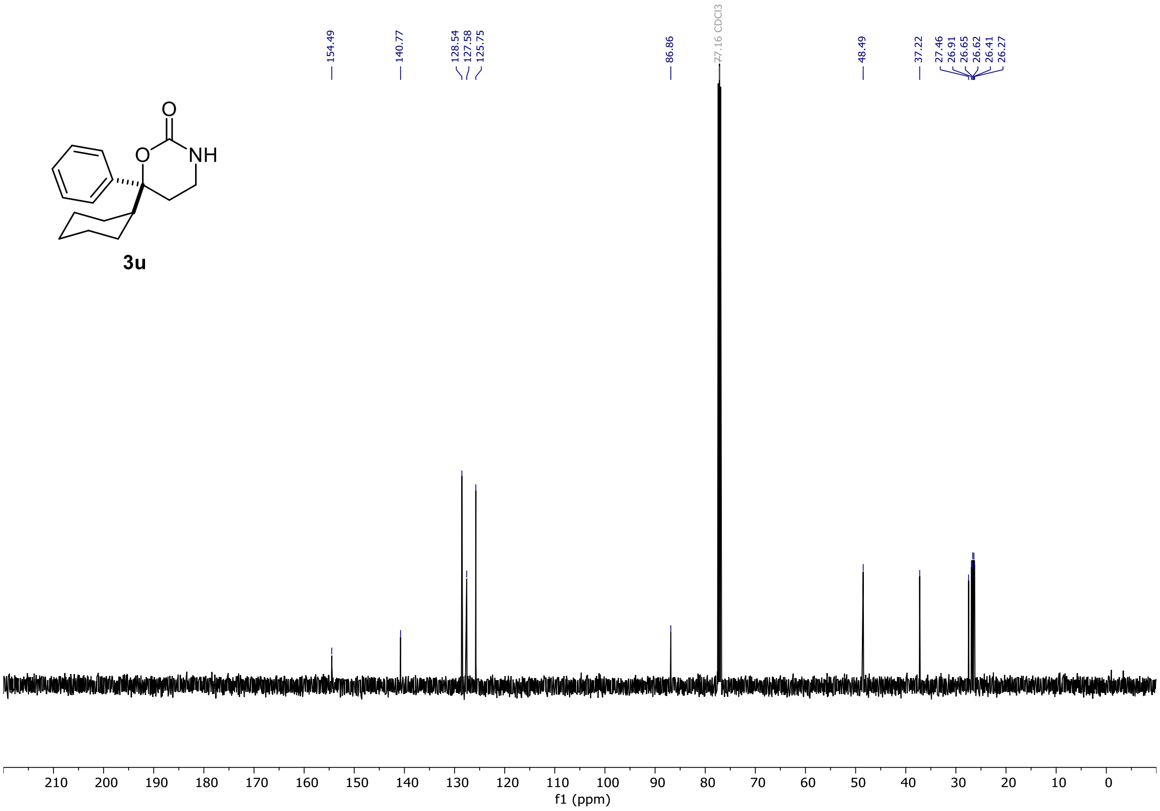

NMR spectra for compound **3u**: ^{1}H (501 MHz) and ^{13}C (126 MHz), in $CDCl_3$.

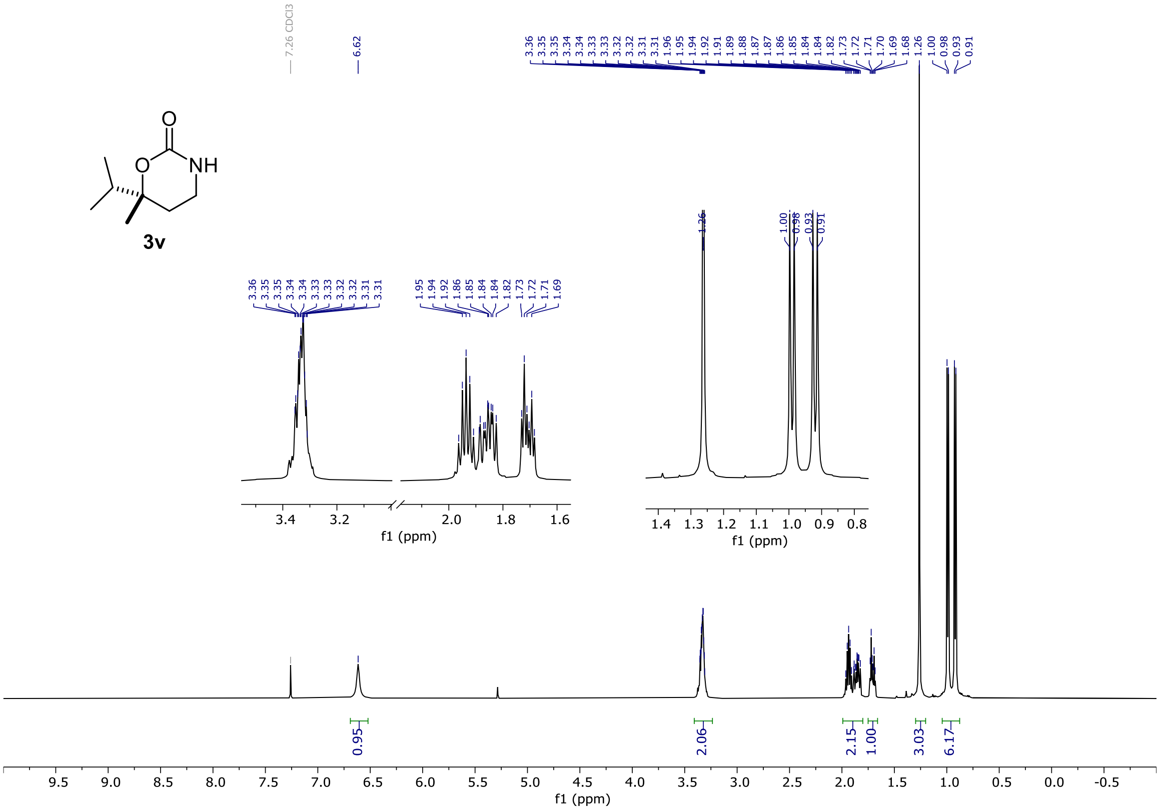

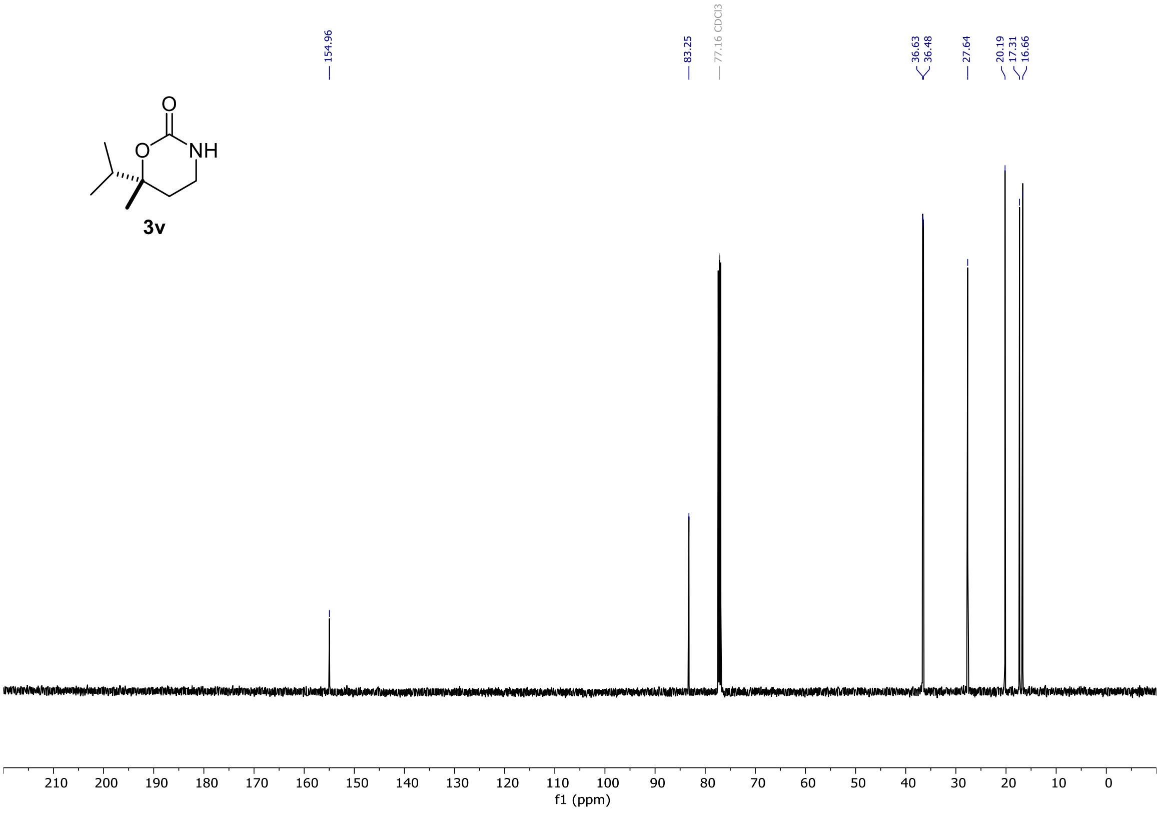

NMR spectra for compound **3v**: ^{1}H (501 MHz) and ^{13}C (126 MHz), in $CDCl_3$.

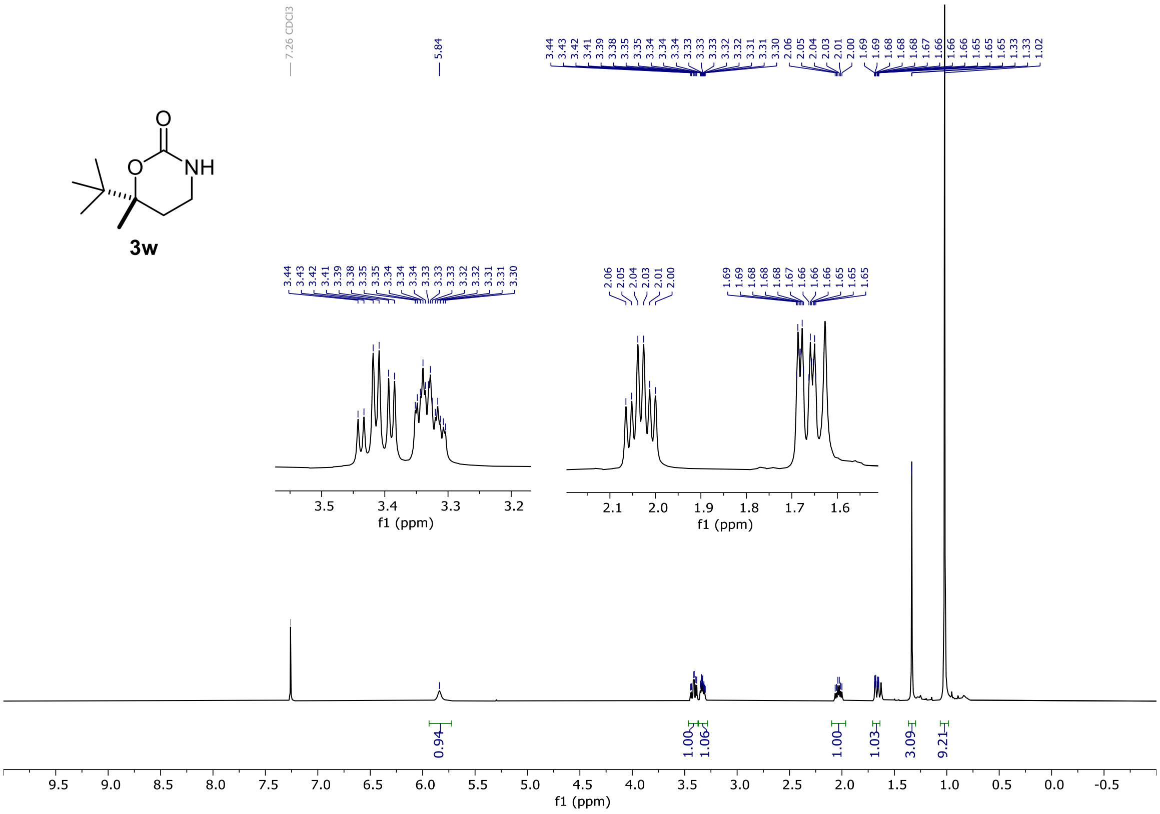

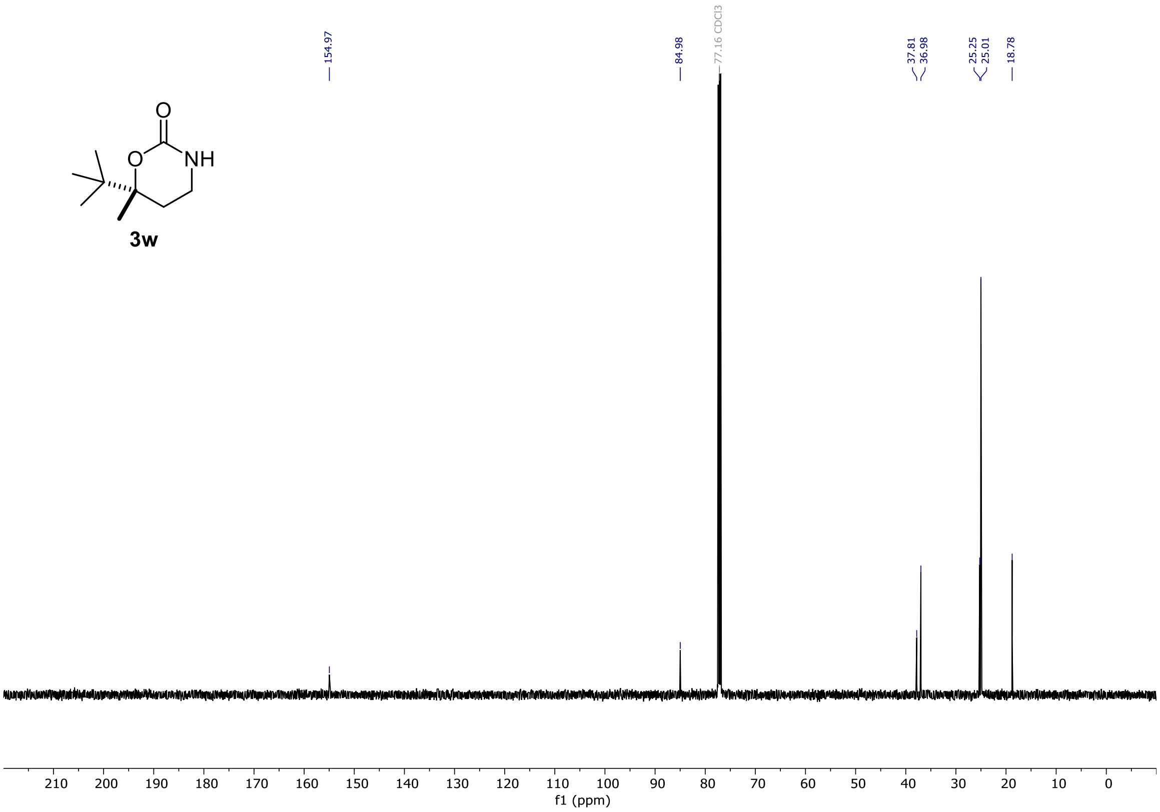

NMR spectra for compound **3w**: ^{1}H (501 MHz) and ^{13}C (126 MHz), in $CDCl_3$.

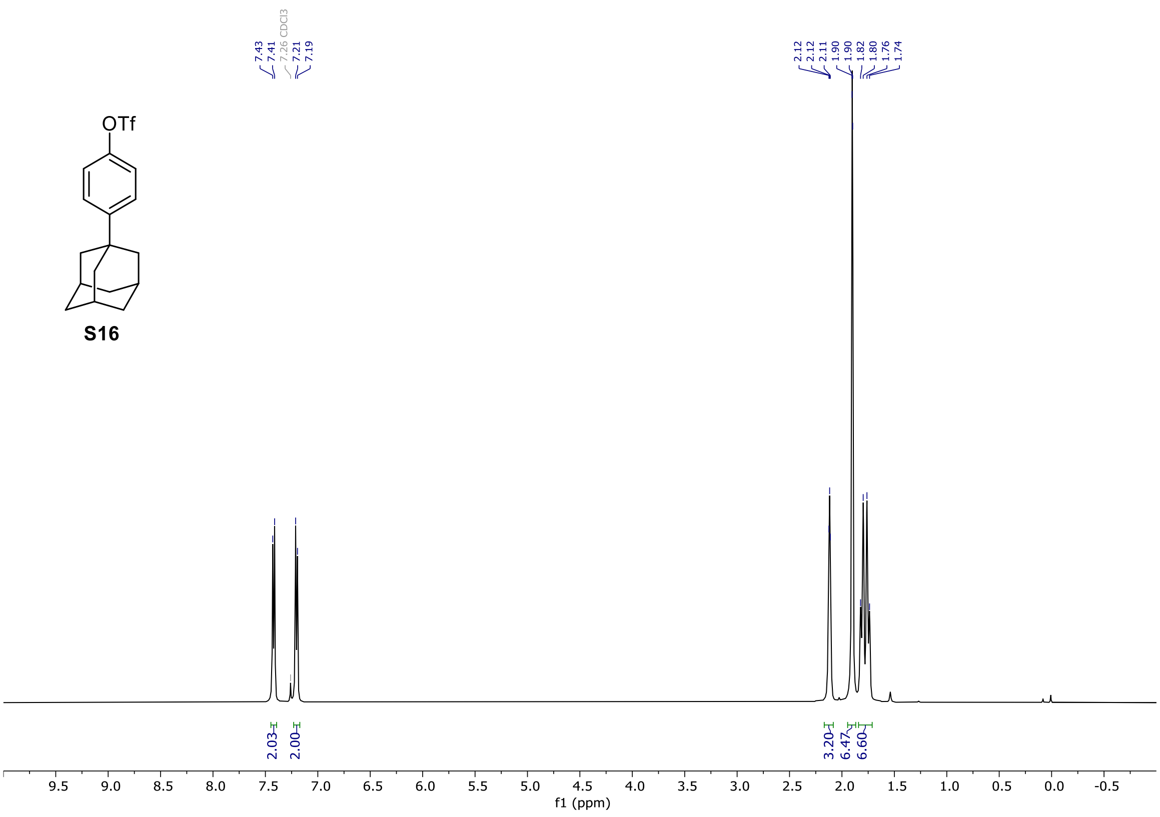
7.43
7.41
7.26 CDCl3
7.21
7.19
2.12
2.12
2.11
1.90
1.90
1.82
1.80
1.76
1.74
OTf
S16
2.03
2.00
3.20
6.47
6.60
9.5 9.0 8.5 8.0 7.5 7.0 6.5 6.0 5.5 5.0 4.5 4.0 3.5 3.0 2.5 2.0 1.5 1.0 0.5 0.0 -0.5
f1 (ppm)

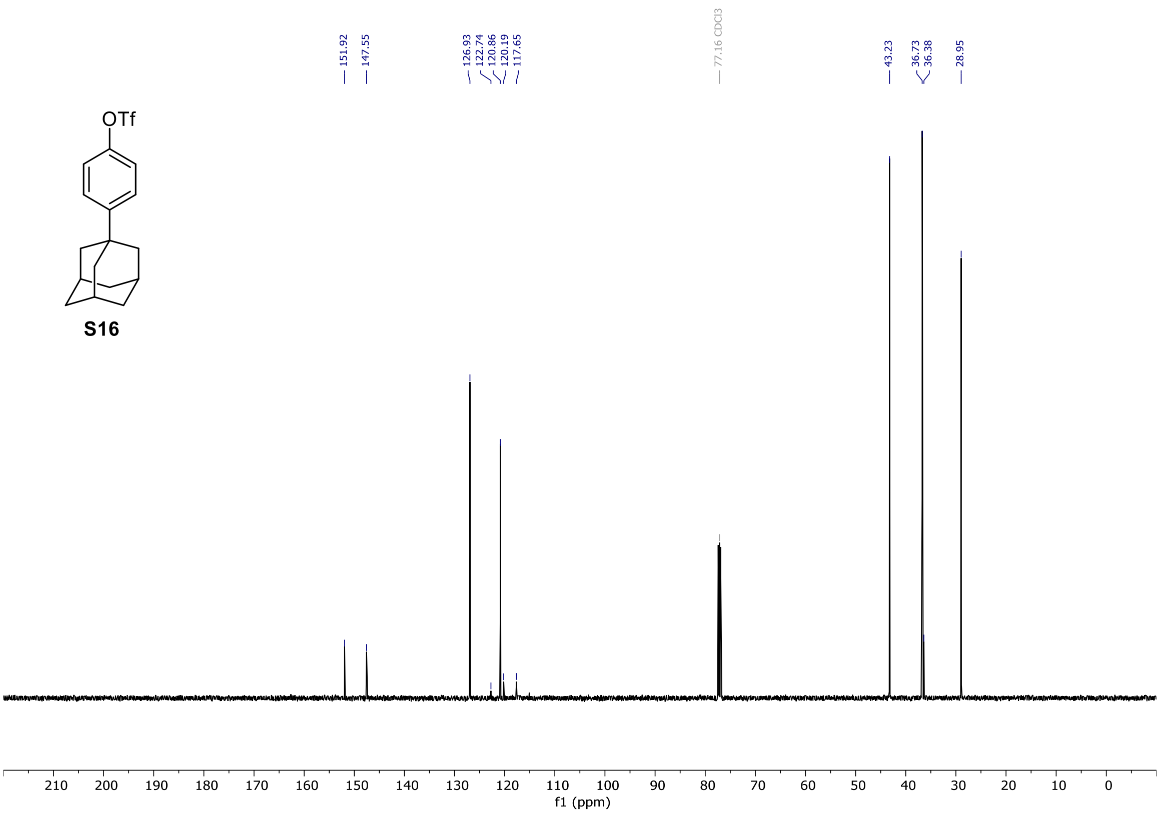
151.92
147.55
126.93
122.74
120.86
120.19
117.65
77.16 CDCl3
43.23
36.73
36.38
28.95
OTf
S16
210 200 190 180 170 160 150 140 130 120 110 100 90 80 70 60 50 40 30 20 10 0
f1 (ppm)

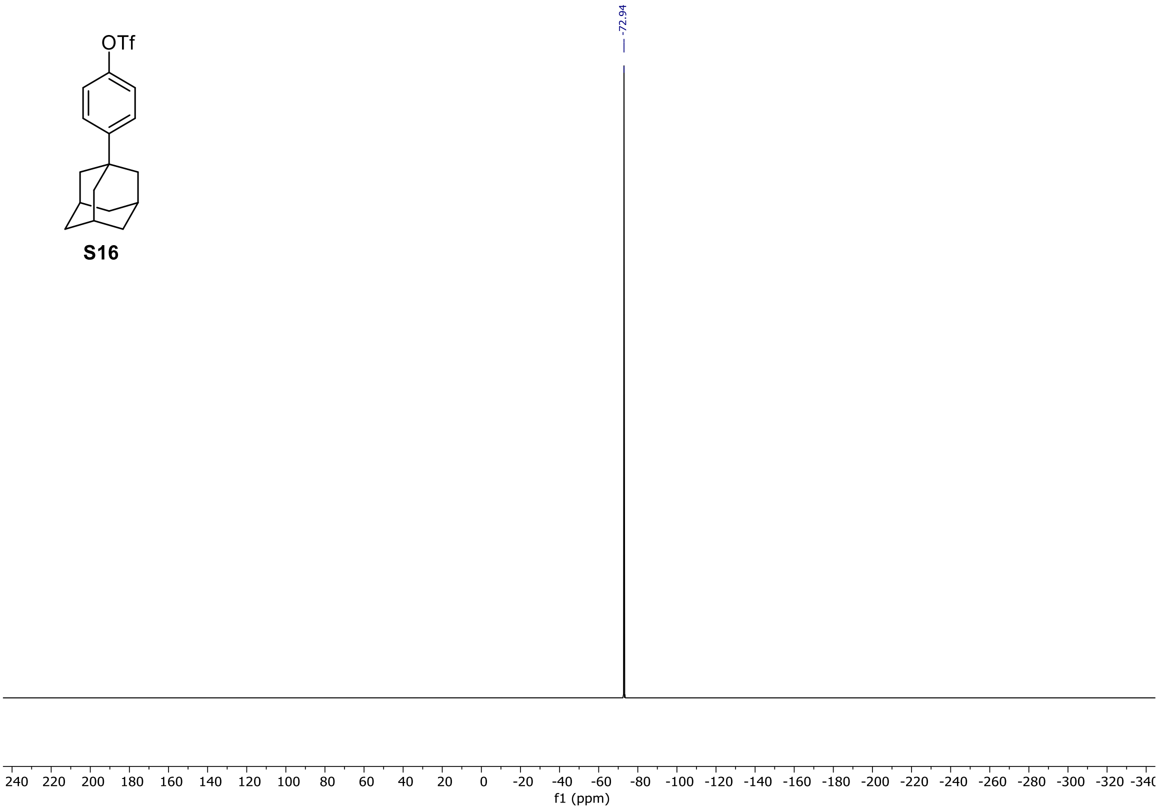

NMR spectra for compound **S16**: ^{1}H (501 MHz), ^{13}C (126 MHz), and ^{19}F (471 MHz), in $CDCl_3$.

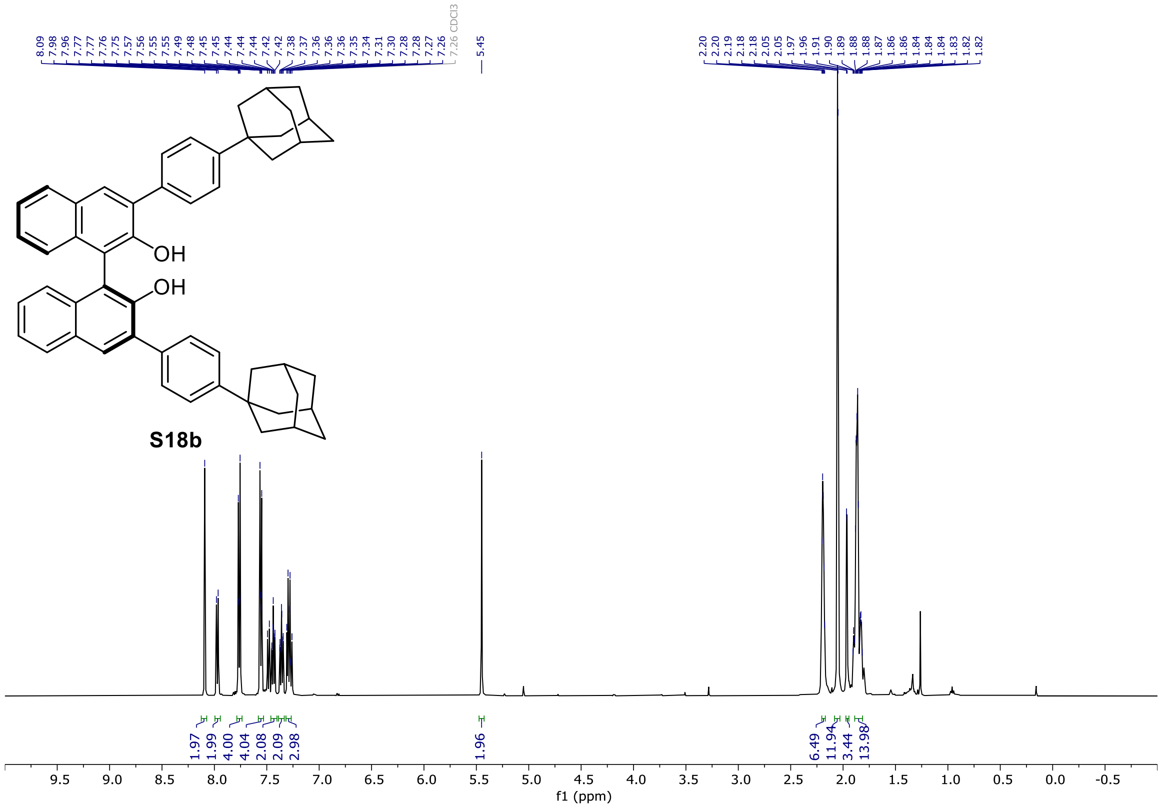

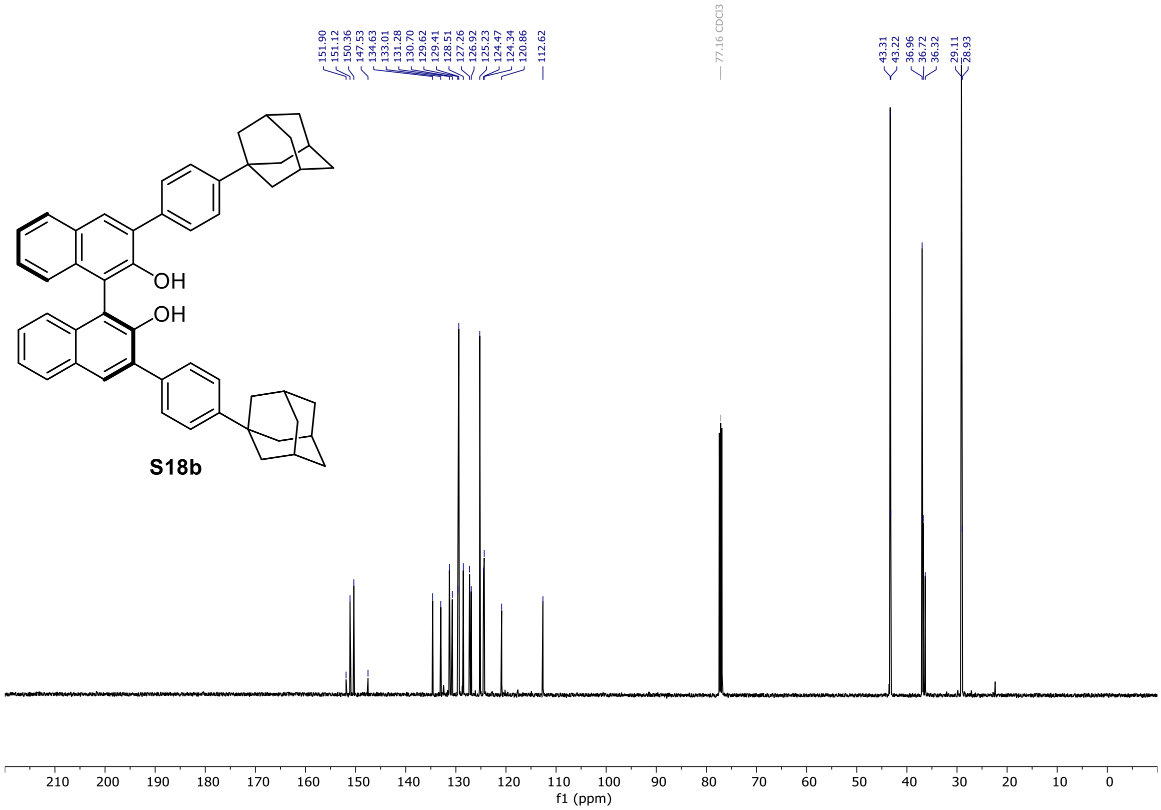

NMR spectra for compound **S18b**: ^{1}H (501 MHz) and ^{13}C (126 MHz), in $CDCl_3$.

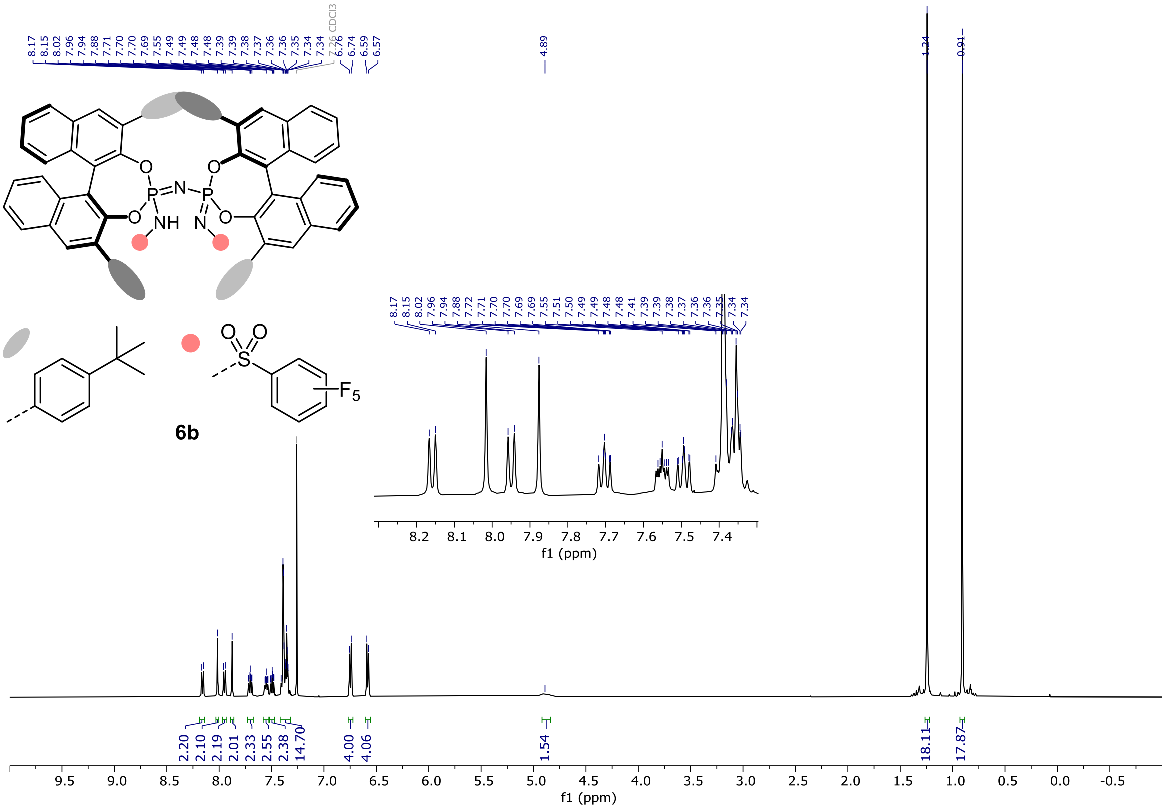
6b
F5
NH
f1 (ppm)

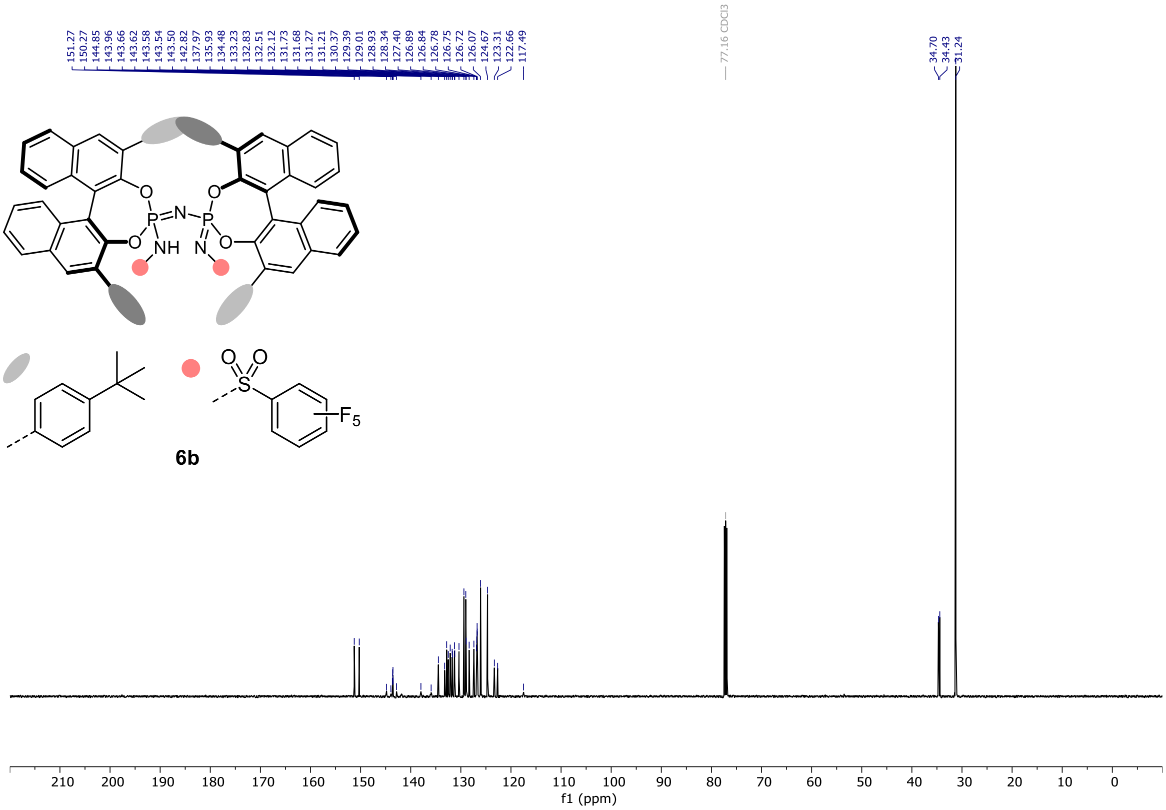
6b
F5
NH
f1 (ppm)

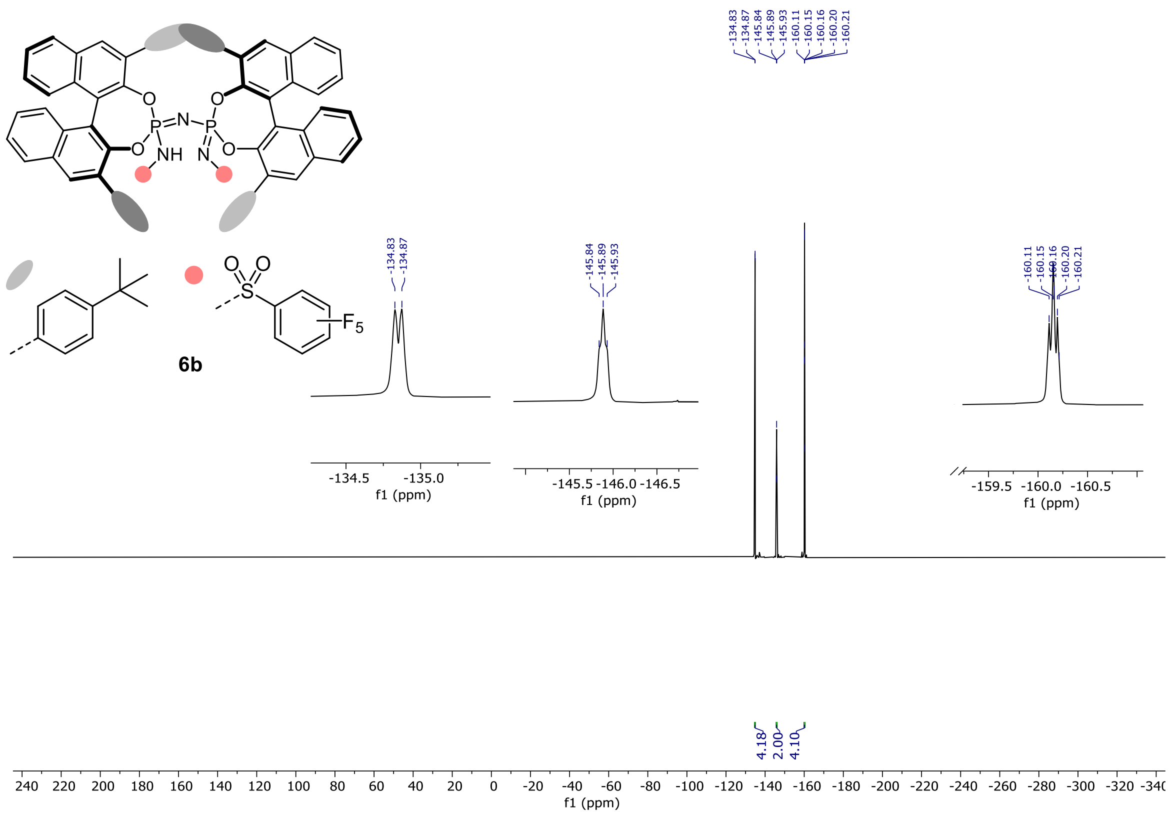

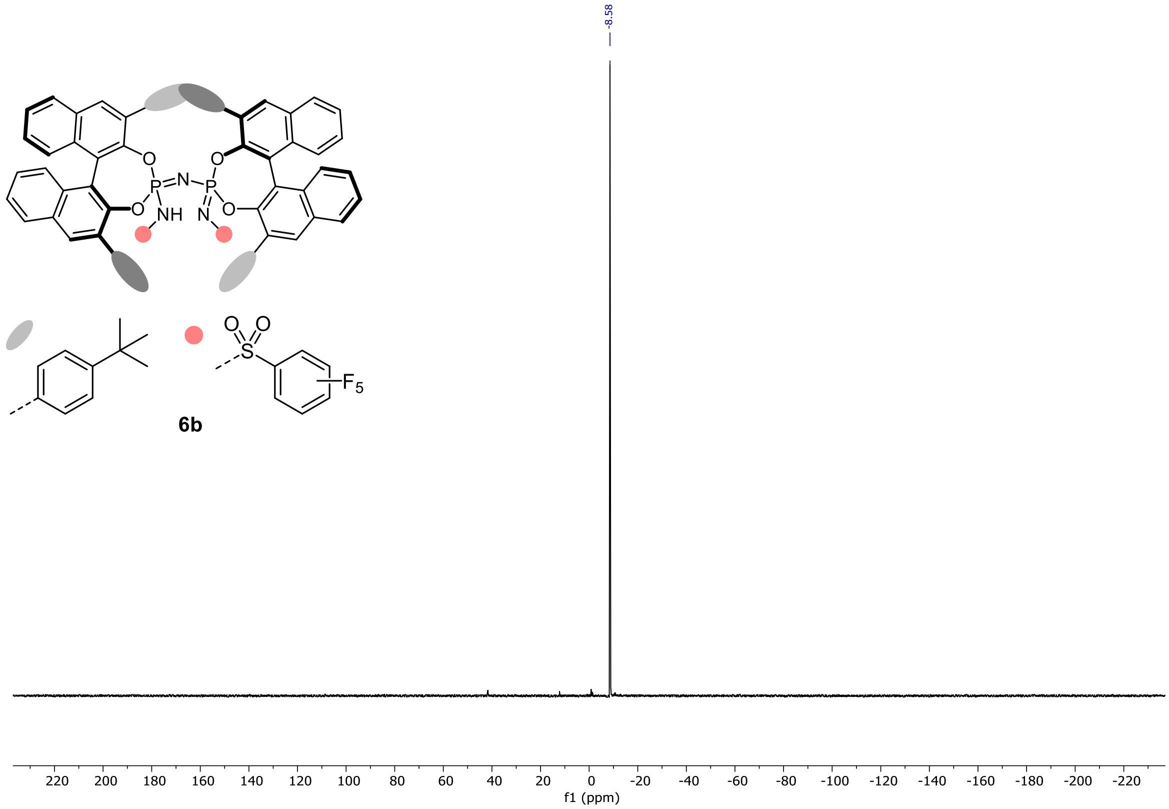

NMR spectra for compound **6b**: ^{1}H (501 MHz), ^{13}C (126 MHz), ^{19}F (471 MHz), and ^{31}P (203 MHz), in $CDCl_3$.

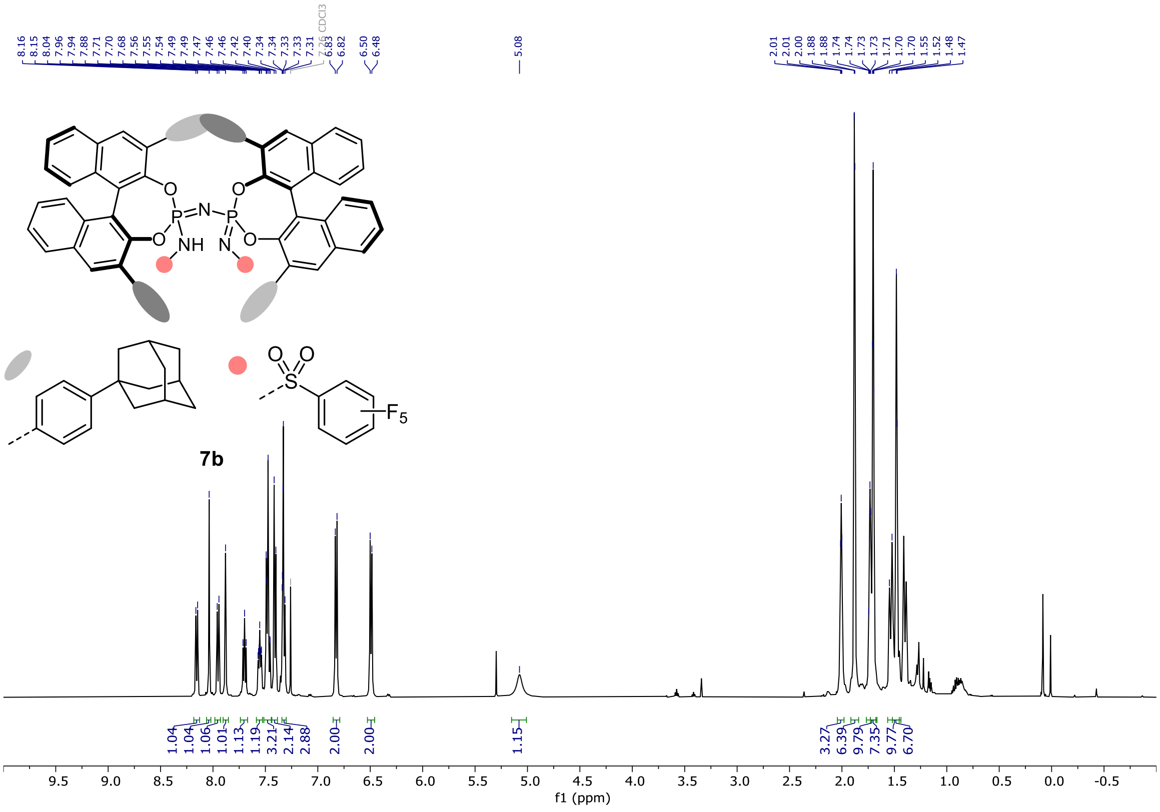
8.16 8.15 8.04 7.96 7.94 7.88 7.71 7.70 7.68 7.56 7.55 7.54 7.49 7.49 7.47 7.46 7.42 7.40 7.34 7.34 7.33 7.33 7.31
CDCl3
6.83 6.82 6.50 6.48
5.08
2.01 2.01 2.00 1.88 1.88 1.74 1.74 1.73 1.73 1.71 1.70 1.70 1.55 1.52 1.48 1.47
O
P=N
P
O
NH
N
F5
7b
1.04 1.04 1.06 1.01 1.13 1.19 3.21 2.14 2.88 2.00 2.00 1.15 3.27 6.39 9.79 7.35 9.77 6.70
9.5 9.0 8.5 8.0 7.5 7.0 6.5 6.0 5.5 5.0 4.5 4.0 3.5 3.0 2.5 2.0 1.5 1.0 0.5 0.0 -0.5
f1 (ppm)

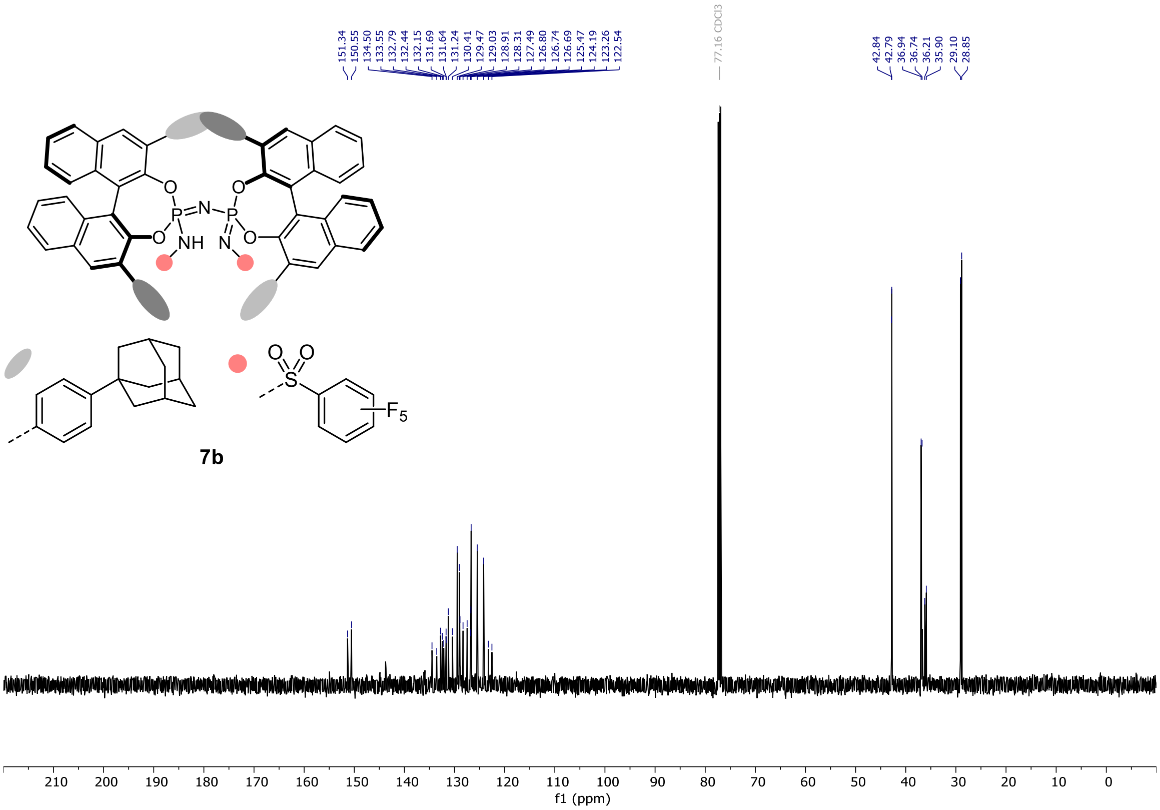
151.34 150.55 134.50 133.55 132.79 132.44 132.15 131.69 131.64 131.24 130.41 129.47 129.03 128.91 128.31 127.49 126.80 126.74 126.69 125.47 124.19 123.26 122.54
77.16 CDCl3
42.84 42.79 36.94 36.74 36.21 35.90 29.10 28.85
O
P=N
P
O
NH
N
F5
7b
210 200 190 180 170 160 150 140 130 120 110 100 90 80 70 60 50 40 30 20 10 0
f1 (ppm)

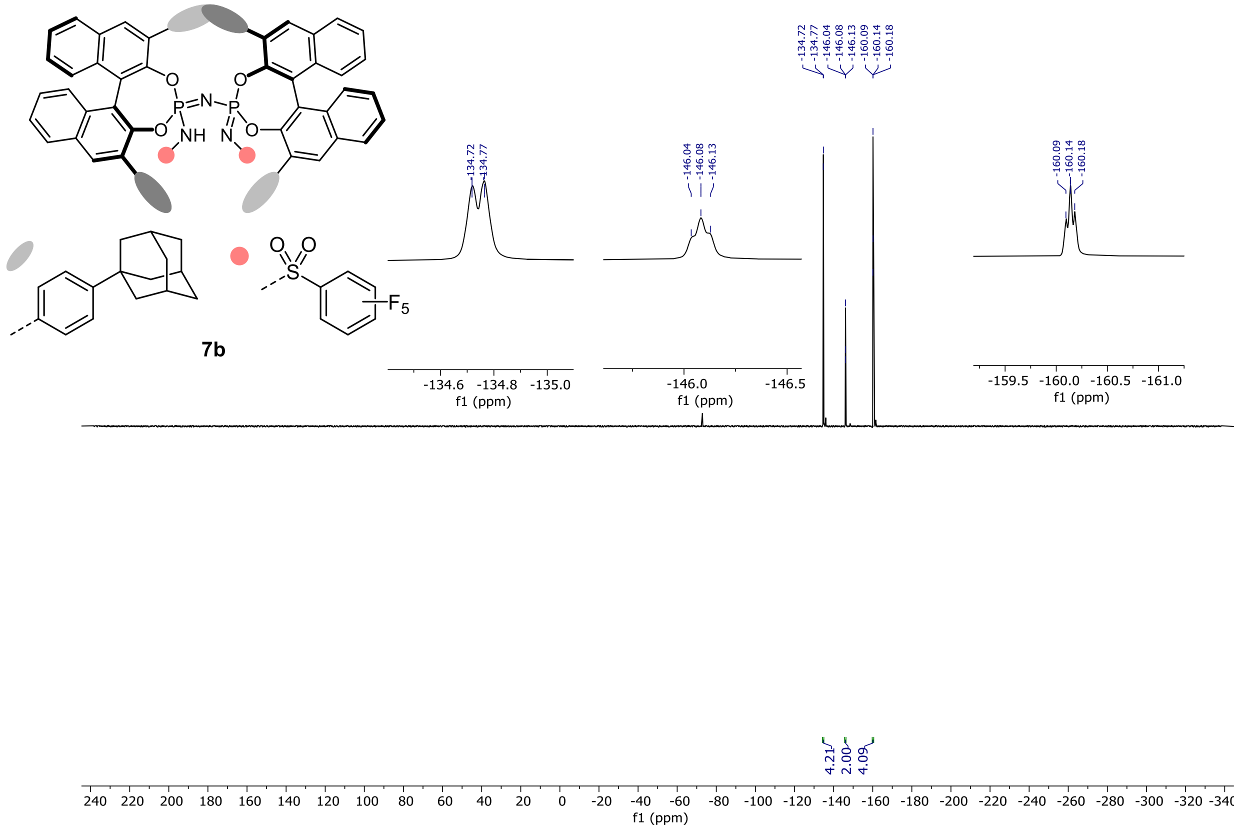

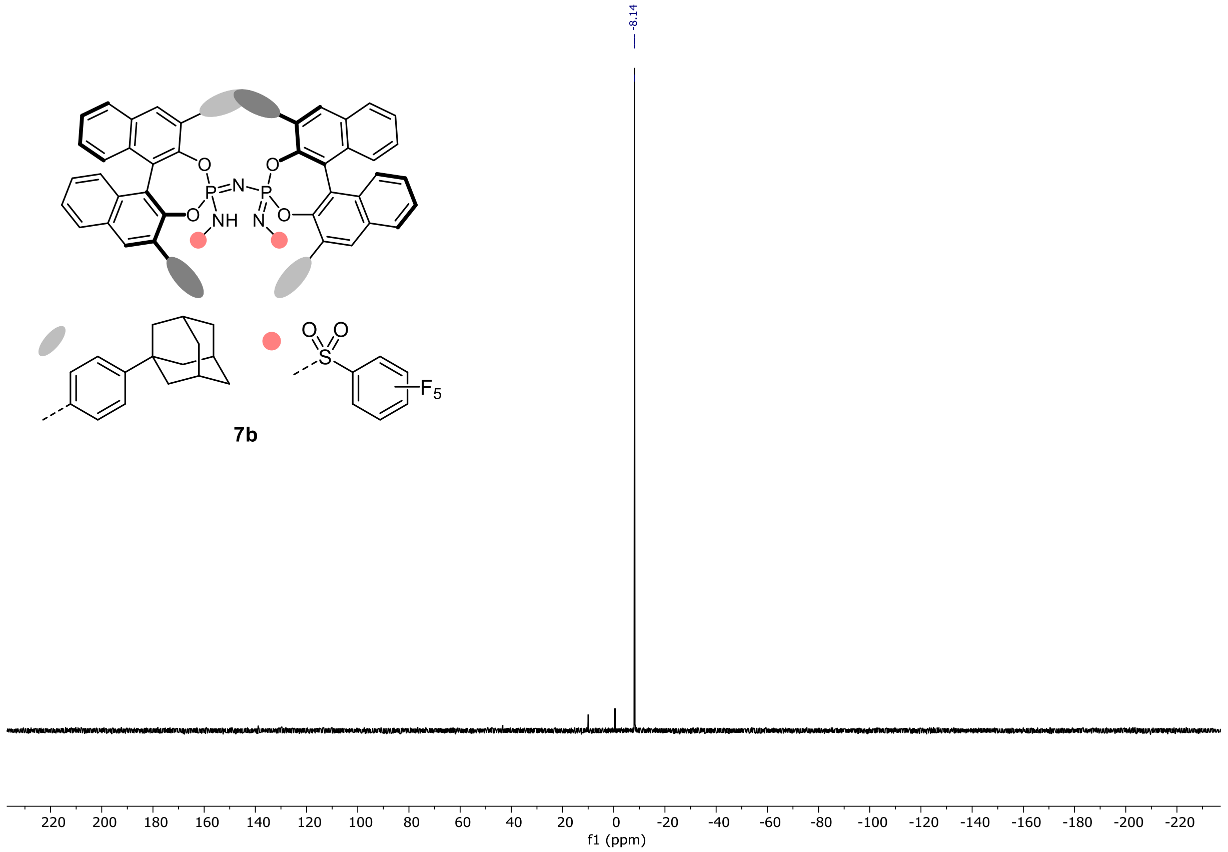

NMR spectra for compound **7b**: ^{1}H (501 MHz), ^{13}C (126 MHz), ^{19}F (471 MHz), and ^{31}P (203 MHz), in $CDCl_3$.

10. Copies of HPLC and GC Traces

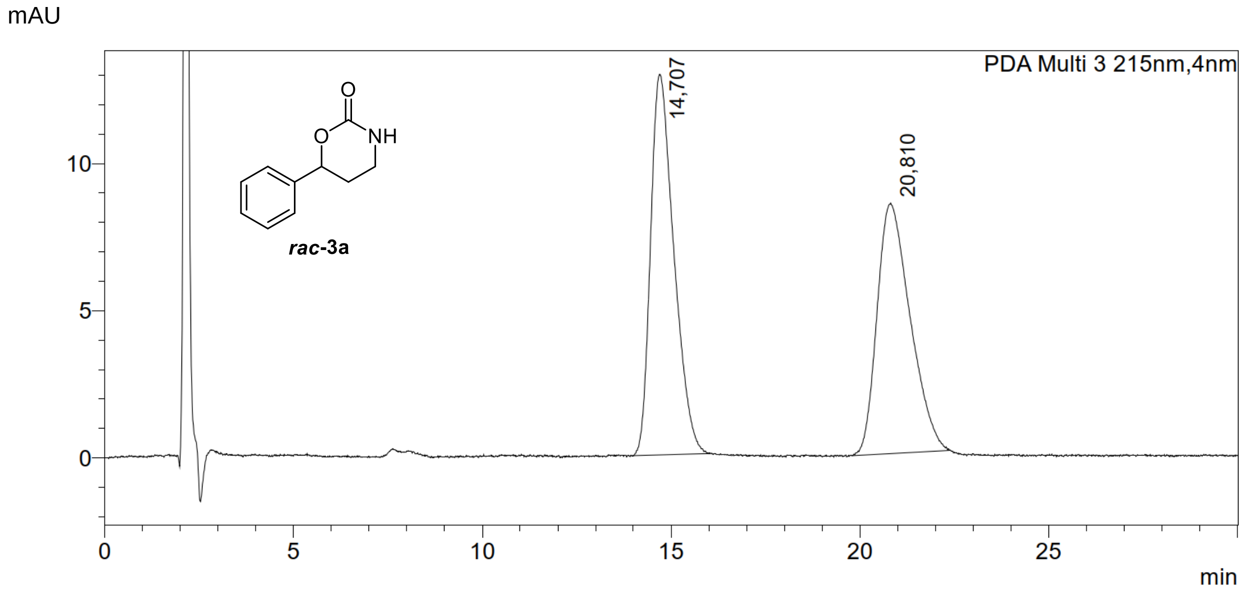

**3a**: IC-3, *n*-heptane/*i*-PrOH 50:50, 298 K, 215 nm		
Peak	Ret. time (min)	Area (%)
1	14.4	50.5
2	20.4	49.5

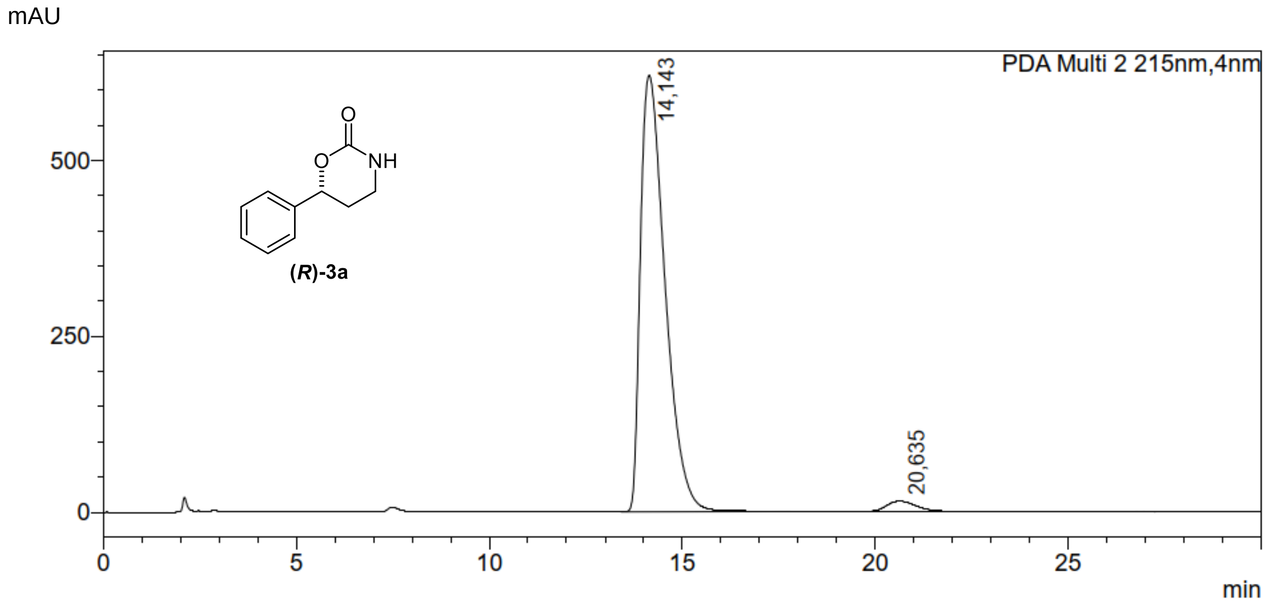

**3a**: IC-3, *n*-heptane/*i*-PrOH 50:50, 298 K, 215 nm		
Peak	Ret. time (min)	Area (%)
1	14.1	97.1
2	20.6	2.9

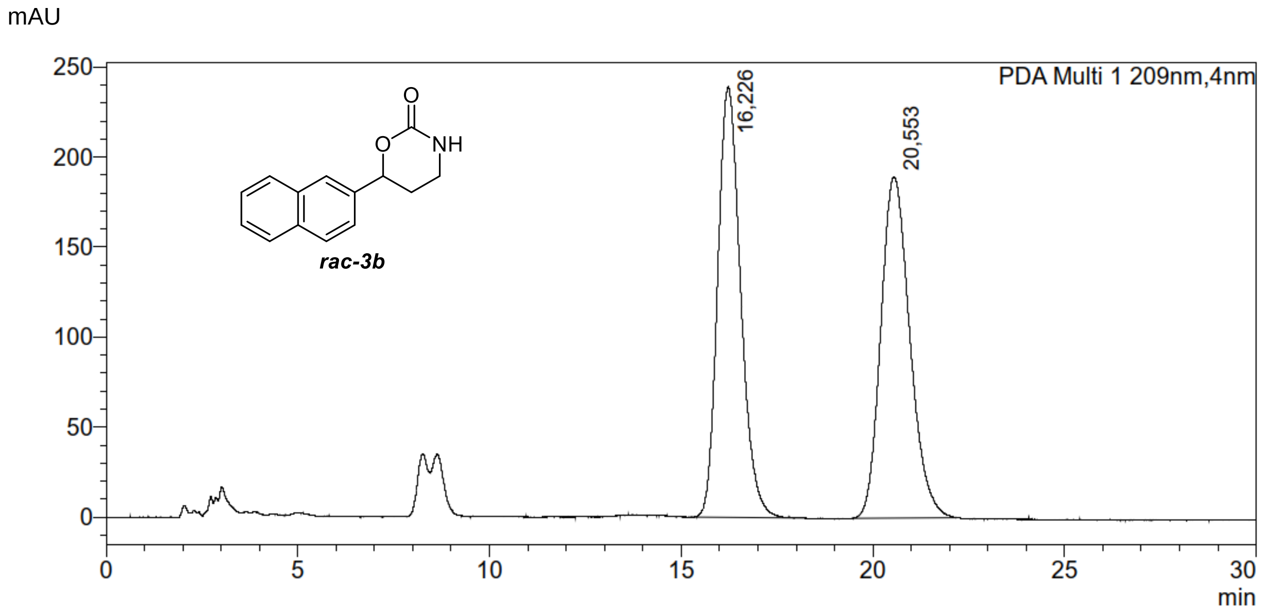

**3b**: IC-3, *n*-heptane/*i*-PrOH 50:50, 298 K, 209 nm		
Peak	Ret. time (min)	Area (%)
1	16.2	50.0
2	20.6	50.0

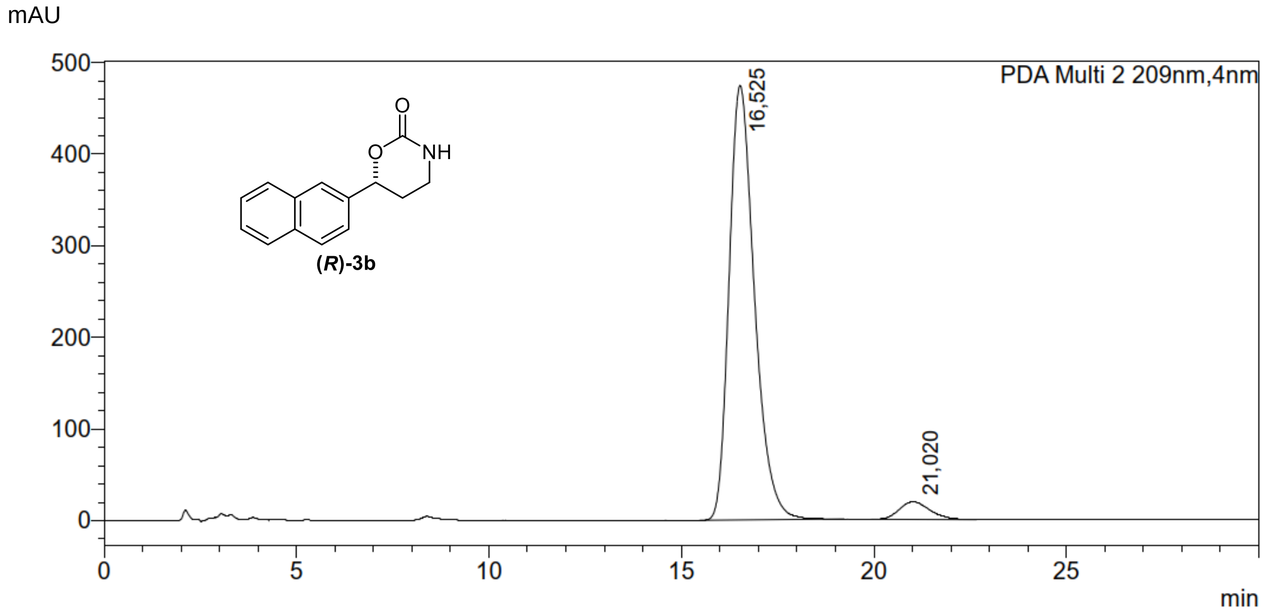

**3b**: IC-3, *n*-heptane/*i*-PrOH 50:50, 298 K, 209 nm		
Peak	Ret. time (min)	Area (%)
1	16.5	95.3
2	21.0	4.7

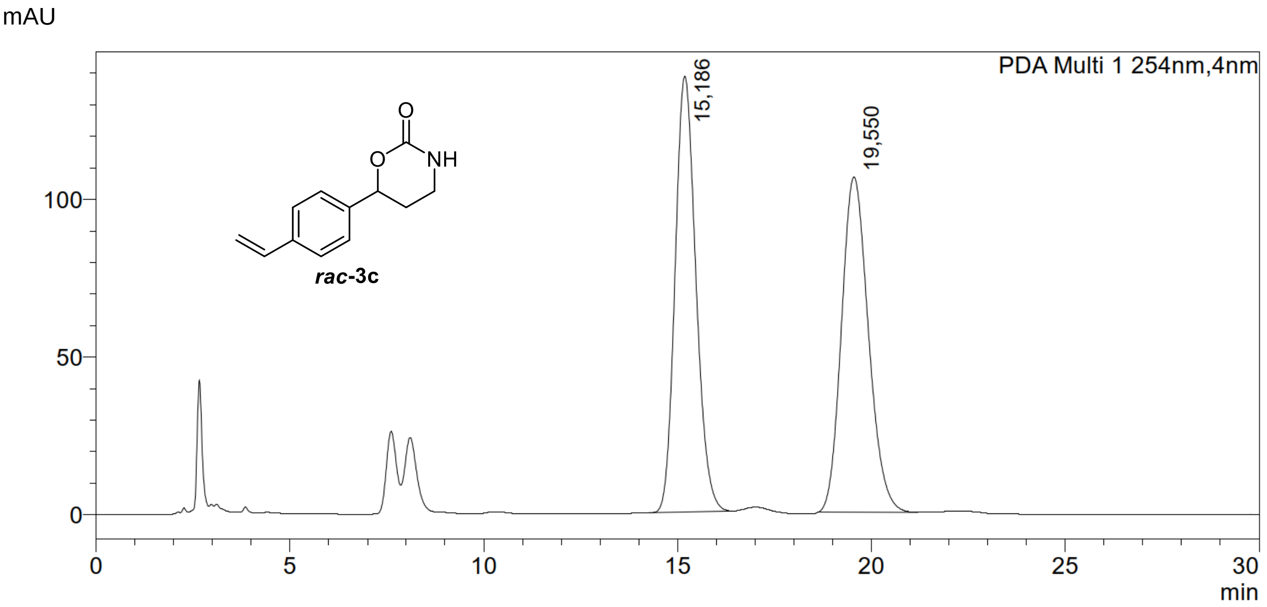

**3c**: IC-3, *n*-heptane/*i*-PrOH 50:50, 298 K, 254 nm		
Peak	Ret. time (min)	Area (%)
1	15.2	50.1
2	19.6	49.9

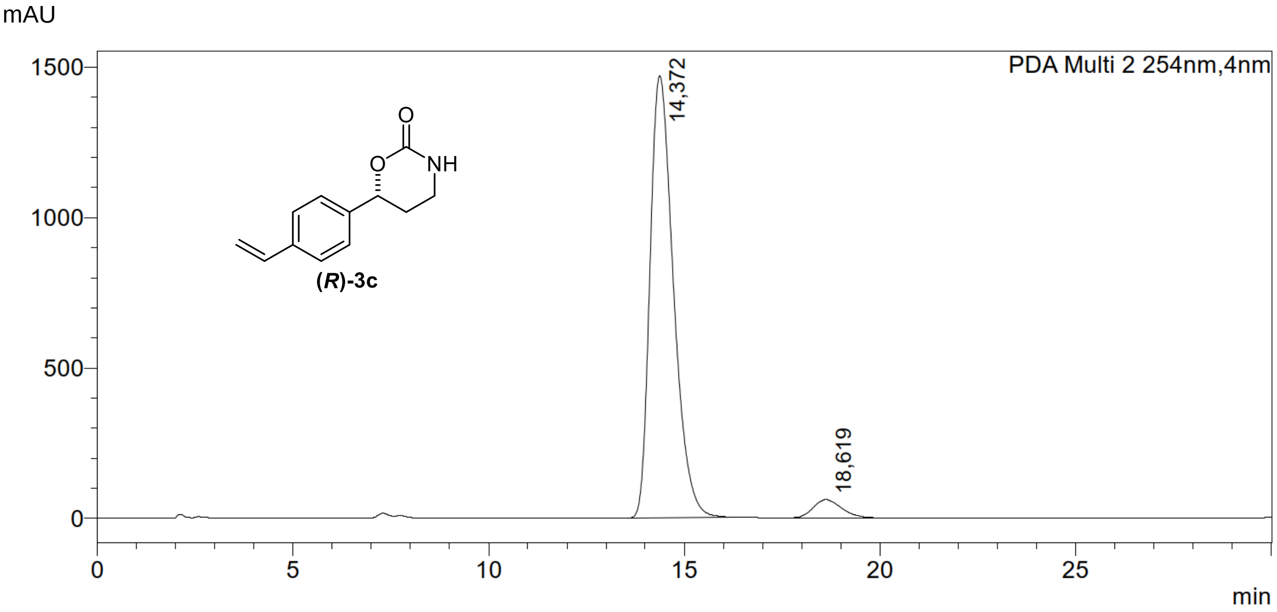

**3c**: IC-3, *n*-heptane/*i*-PrOH 50:50, 298 K, 254 nm		
Peak	Ret. time (min)	Area (%)
1	15.0	95.4
2	19.6	4.6

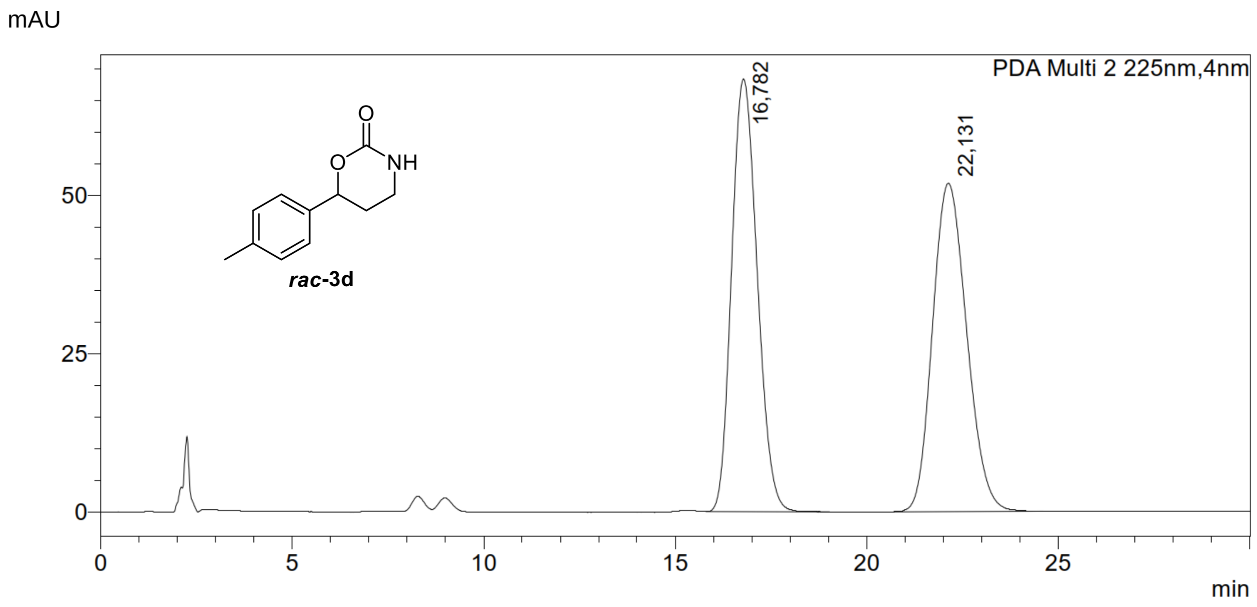

**3d**: IC-3, *n*-heptane/*i*-PrOH 50:50, 298 K, 225 nm		
Peak	Ret. time (min)	Area (%)
1	16.8	49.7
2	22.1	50.3

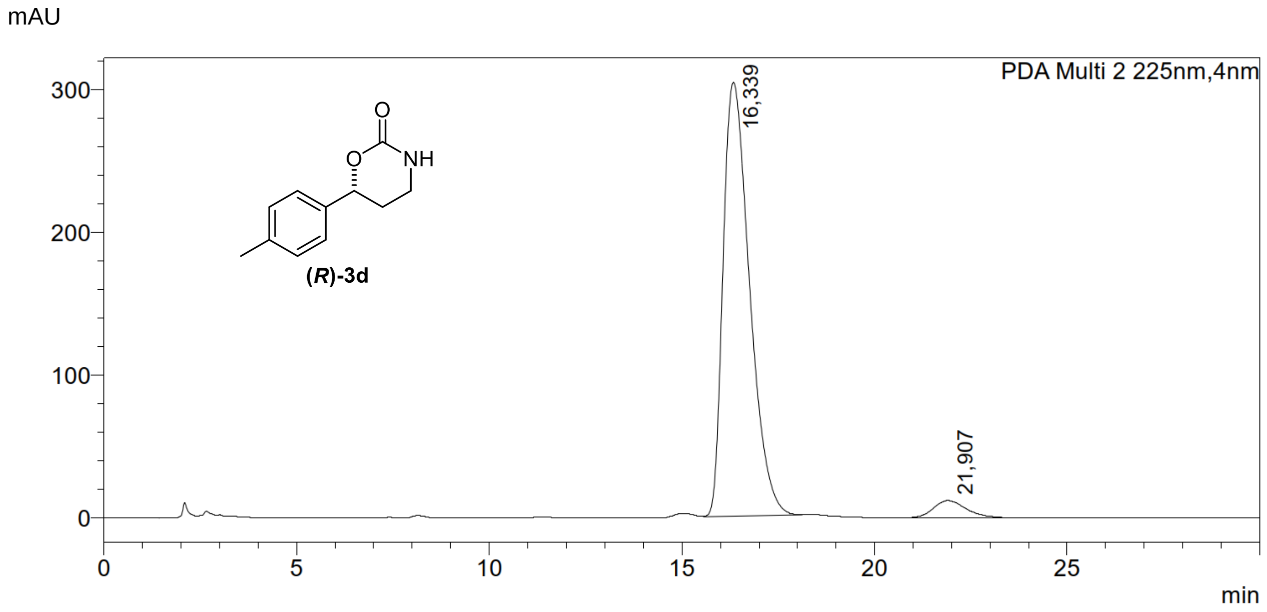

**3d**: IC-3, *n*-heptane/*i*-PrOH 50:50, 298 K, 225 nm		
Peak	Ret. time (min)	Area (%)
1	16.3	95.5
2	21.9	4.5

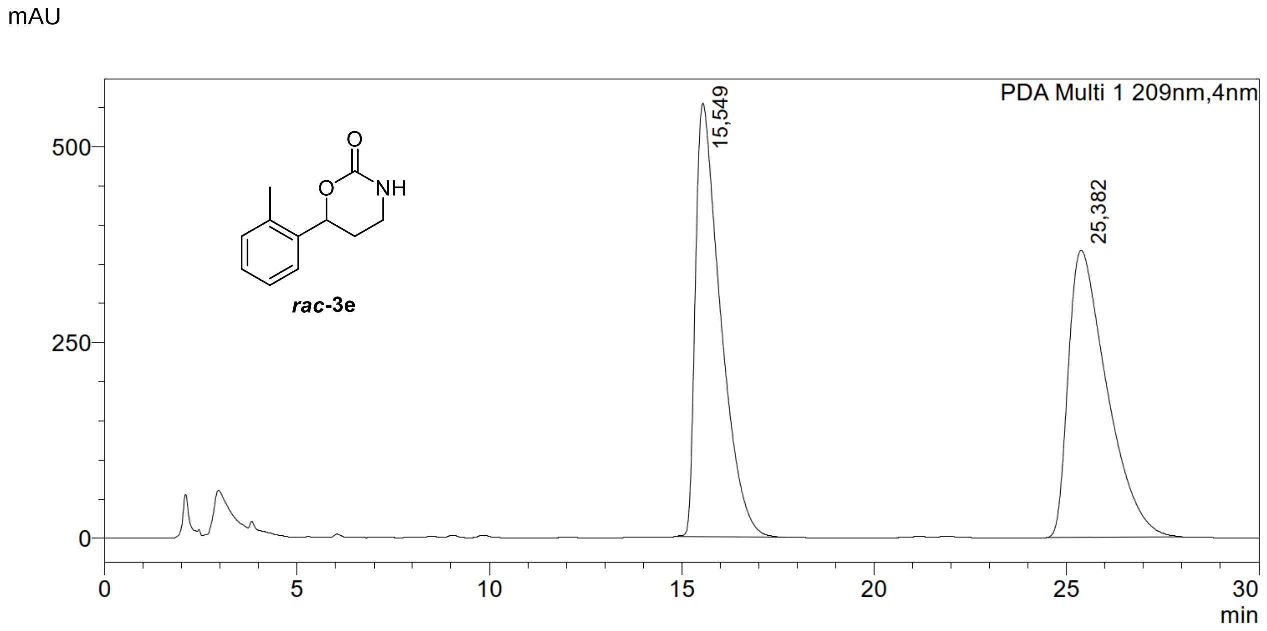

**3e**: IC-3, *n*-heptane/*i*-PrOH 50:50, 298 K, 209 nm		
Peak	Ret. time (min)	Area (%)
1	15.5	49.5
2	25.4	50.5

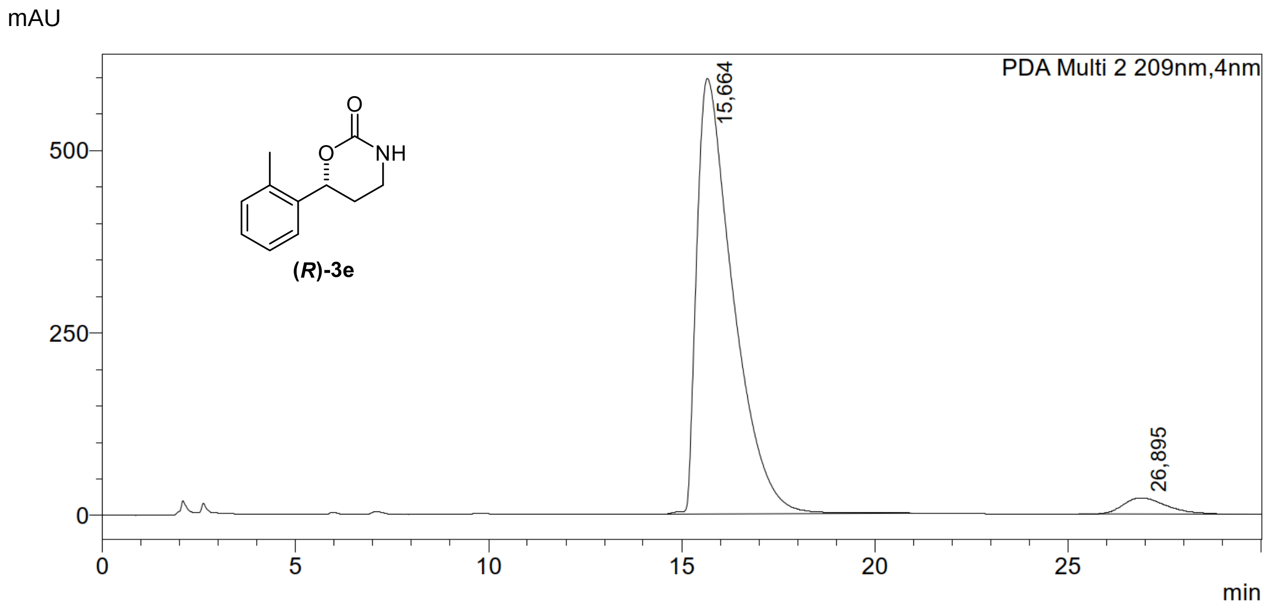

**3e**: IC-3, *n*-heptane/*i*-PrOH 50:50, 298 K, 209 nm		
Peak	Ret. time (min)	Area (%)
1	15.7	95.8
2	26.9	4.2

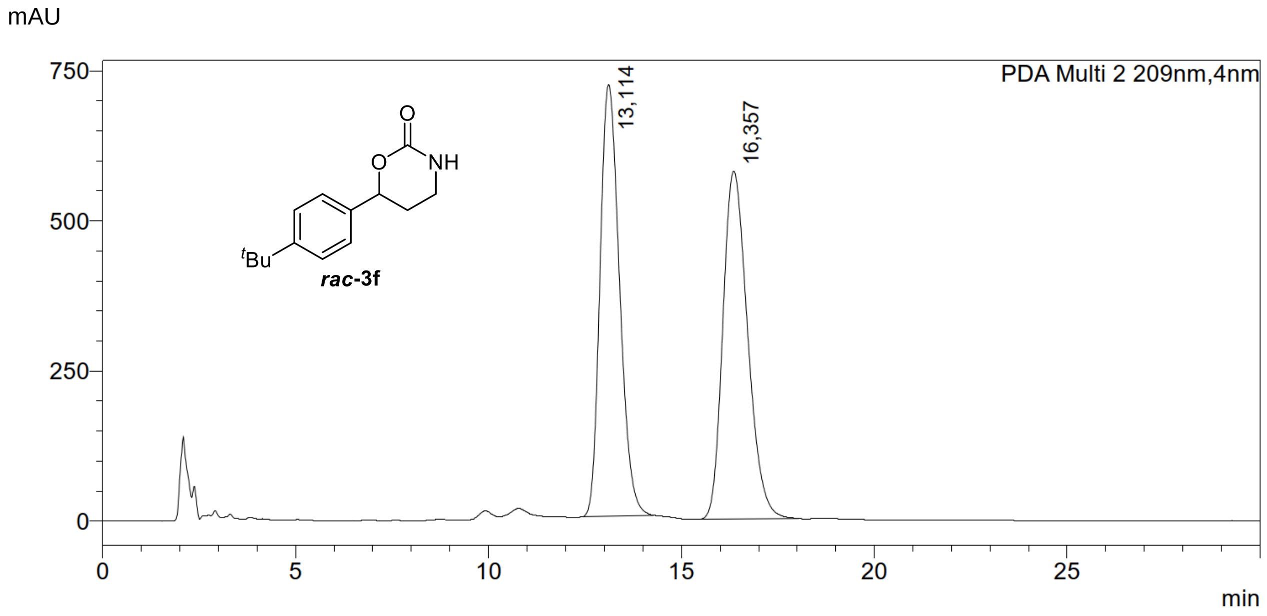

**3f**: IC-3, *n*-heptane/*i*-PrOH 50:50, 298 K, 209 nm		
Peak	Ret. time (min)	Area (%)
1	13.1	49.4
2	16.4	50.6

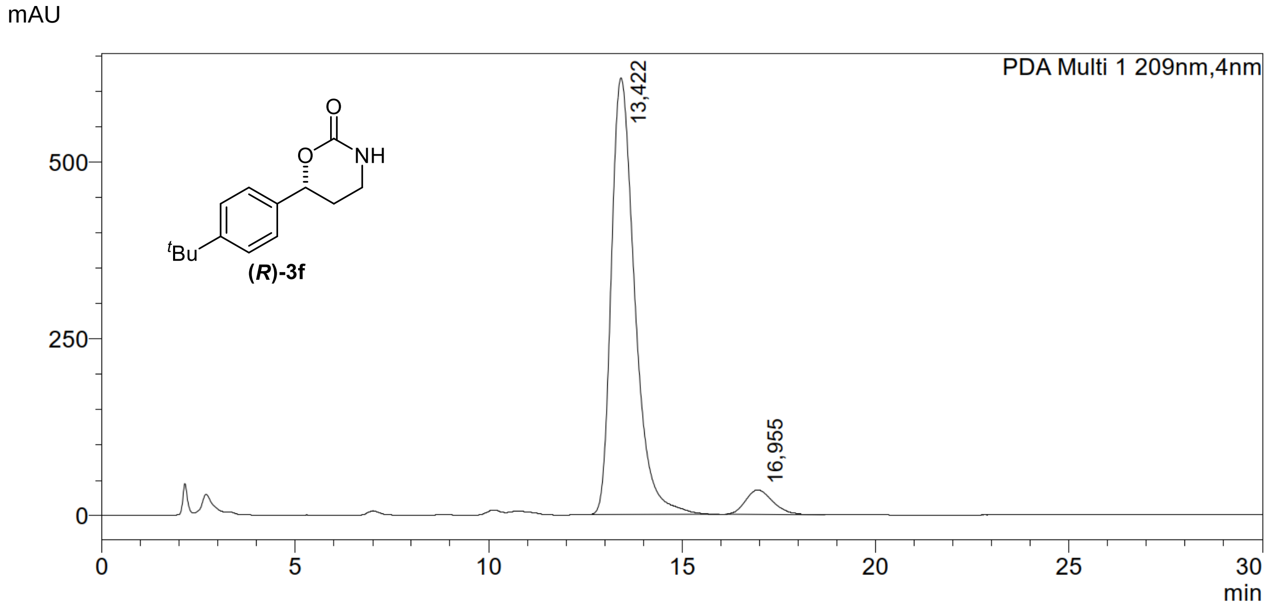

**3f**: IC-3, *n*-heptane/*i*-PrOH 50:50, 298 K, 209 nm		
Peak	Ret. time (min)	Area (%)
1	13.4	94.0
2	16.9	6.0

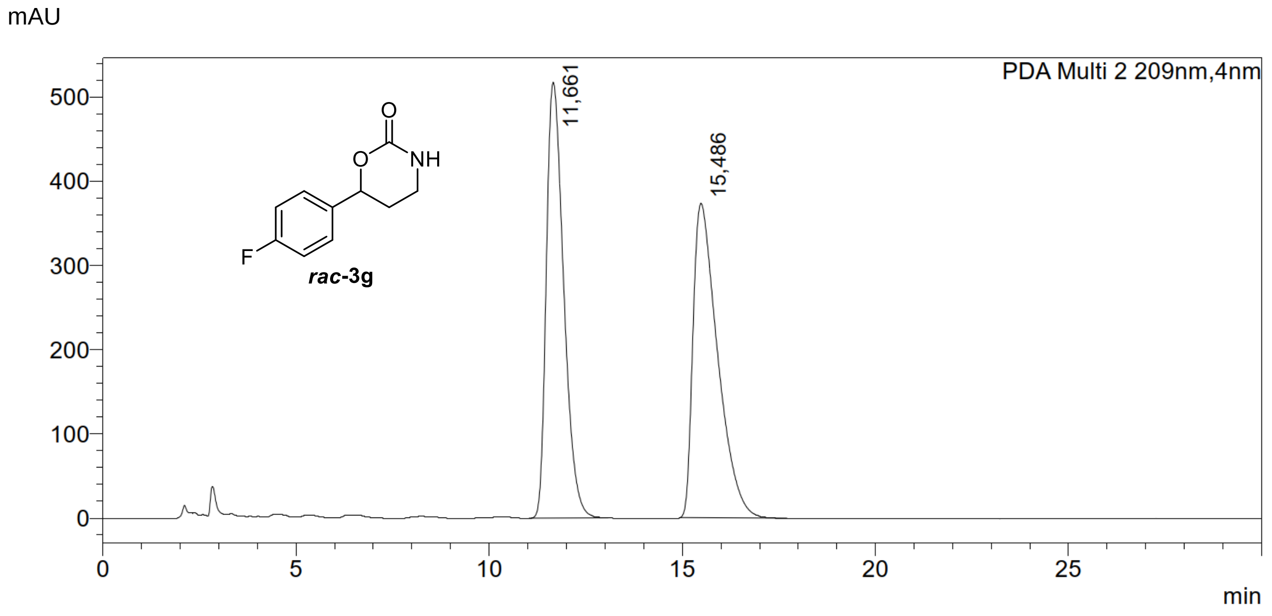

**3g**: IC-3, *n*-heptane/*i*-PrOH 50:50, 298 K, 209 nm		
Peak	Ret. time (min)	Area (%)
1	11.7	50.2
2	15.5	49.8

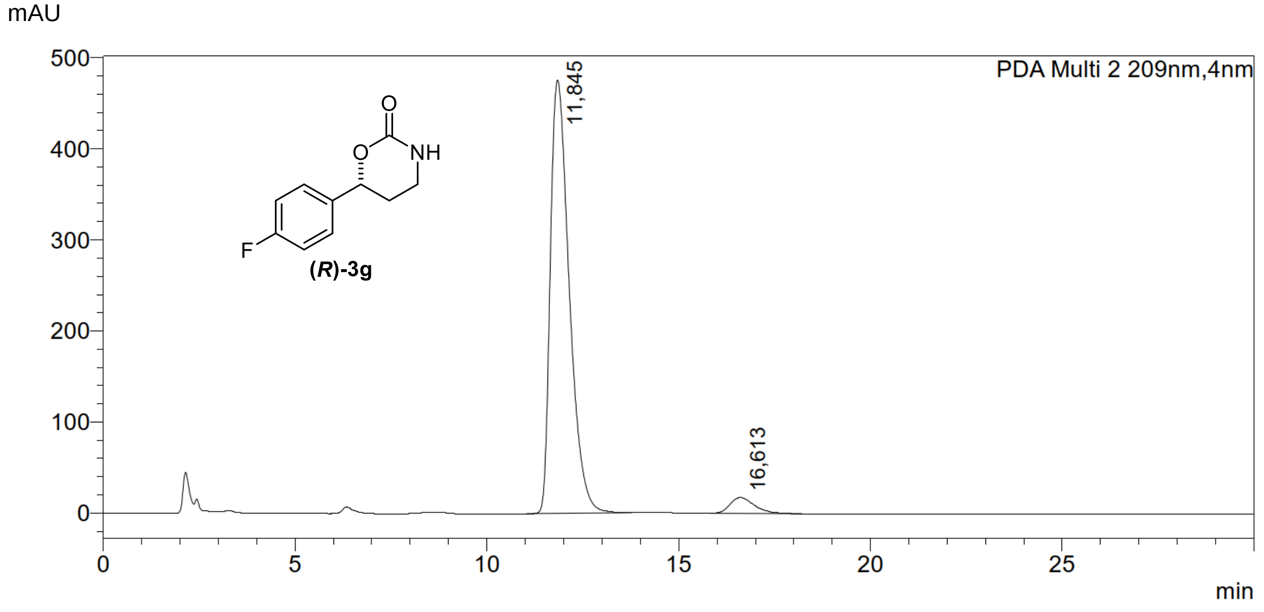

**3g**: IC-3, *n*-heptane/*i*-PrOH 50:50, 298 K, 209 nm		
Peak	Ret. time (min)	Area (%)
1	11.8	95.5
2	16.6	4.5

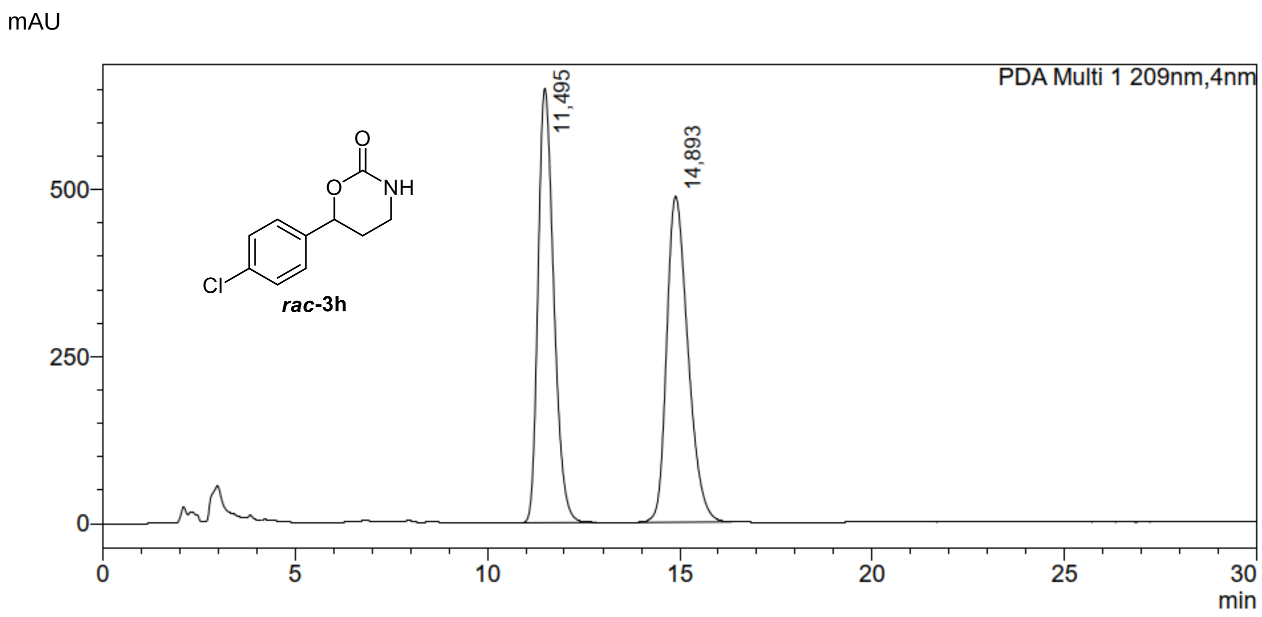

**3h**: IC-3, *n*-heptane/*i*-PrOH 50:50, 298 K, 209 nm		
Peak	Ret. time (min)	Area (%)
1	11.5	49.7
2	14.9	50.3

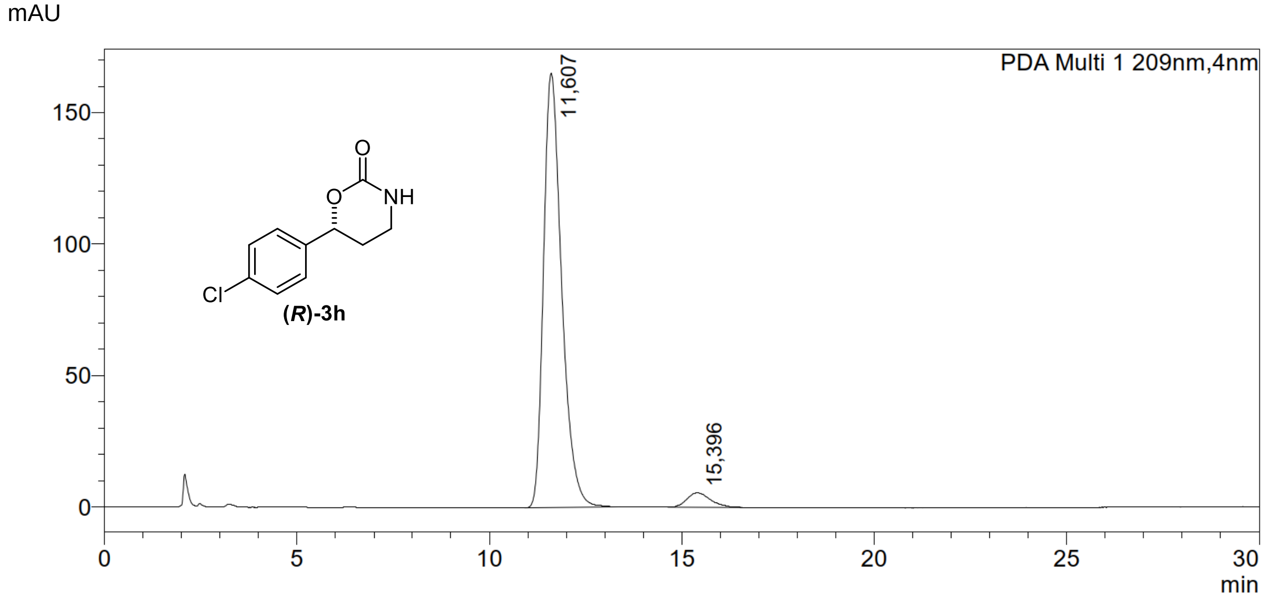

**3h**: IC-3, *n*-heptane/*i*-PrOH 50:50, 298 K, 209 nm		
Peak	Ret. time (min)	Area (%)
1	11.6	95.9
2	15.4	4.1

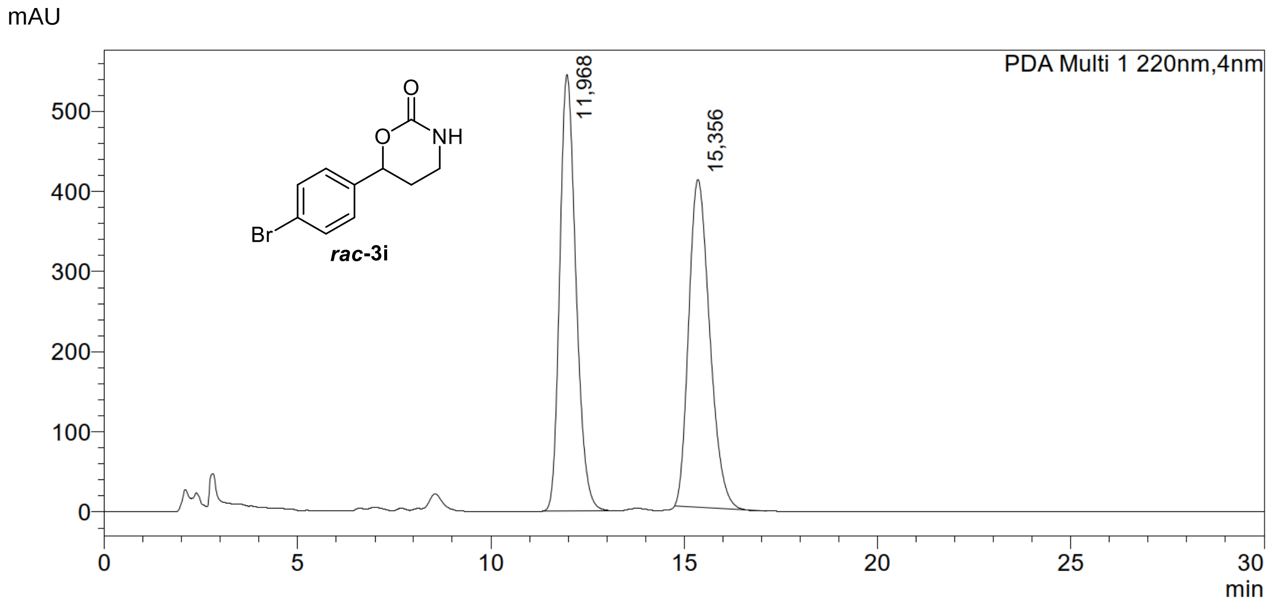

**3i**: IC-3, *n*-heptane/*i*-PrOH 50:50, 298 K, 220 nm		
Peak	Ret. time (min)	Area (%)
1	12.0	50.6
2	15.4	49.4

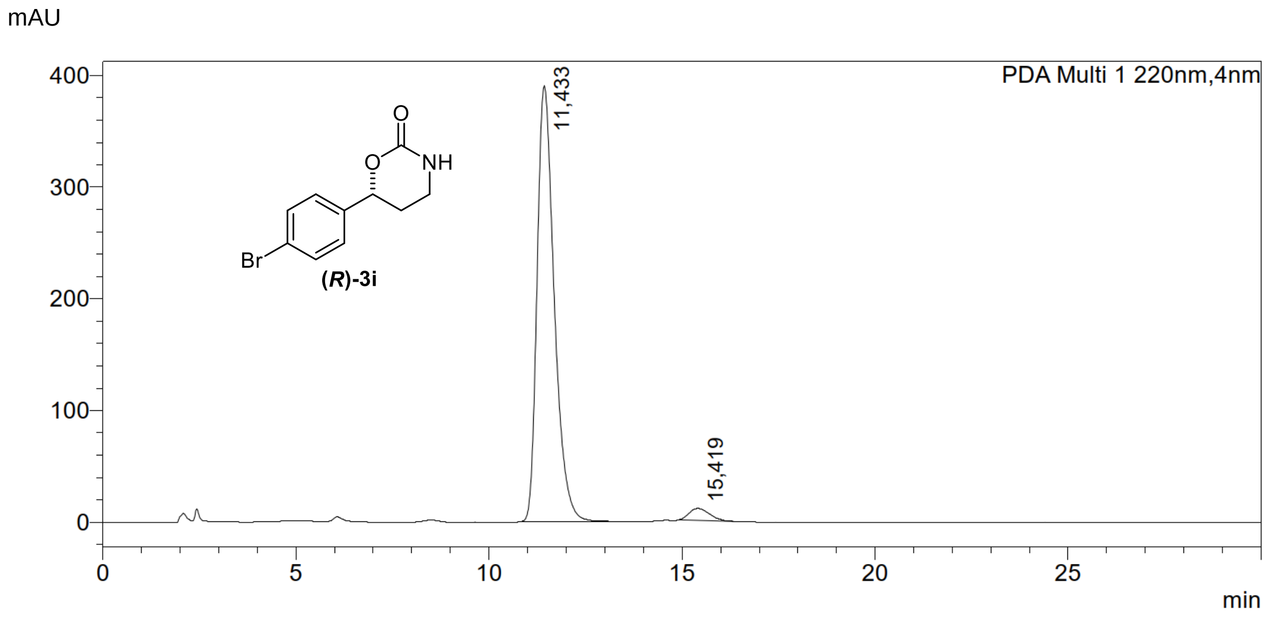

**3i**: IC-3, *n*-heptane/*i*-PrOH 50:50, 298 K, 220 nm		
Peak	Ret. time (min)	Area (%)
1	11.4	96.9
2	15.4	3.1

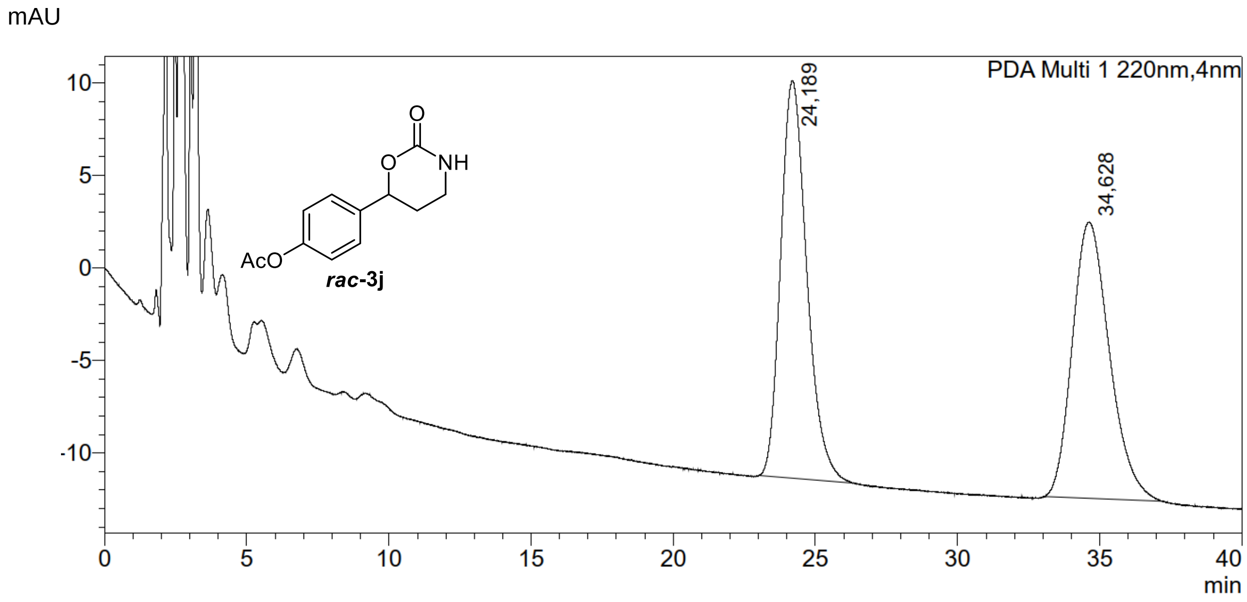

**3j**: IC-3, *n*-heptane/*i*-PrOH 50:50, 298 K, 220 nm		
Peak	Ret. time (min)	Area (%)
10	24.2	50.1
11	34.6	49.9

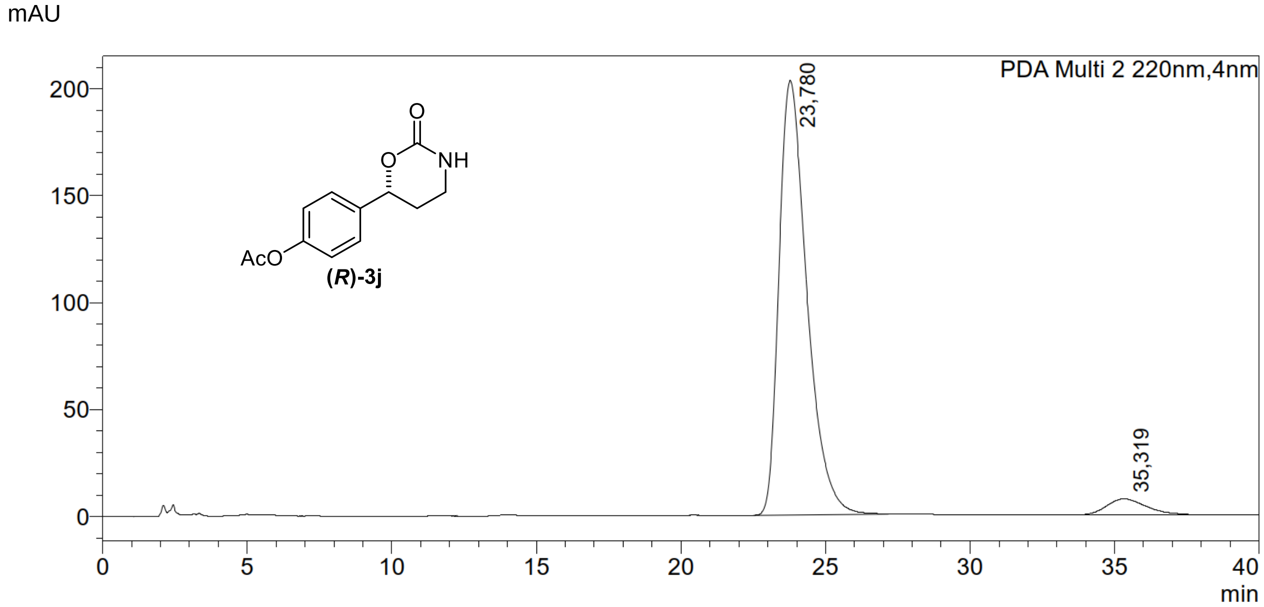

**3j**: IC-3, *n*-heptane/*i*-PrOH 50:50, 298 K, 220 nm		
Peak	Ret. time (min)	Area (%)
1	23.8	95.1
2	35.3	4.9

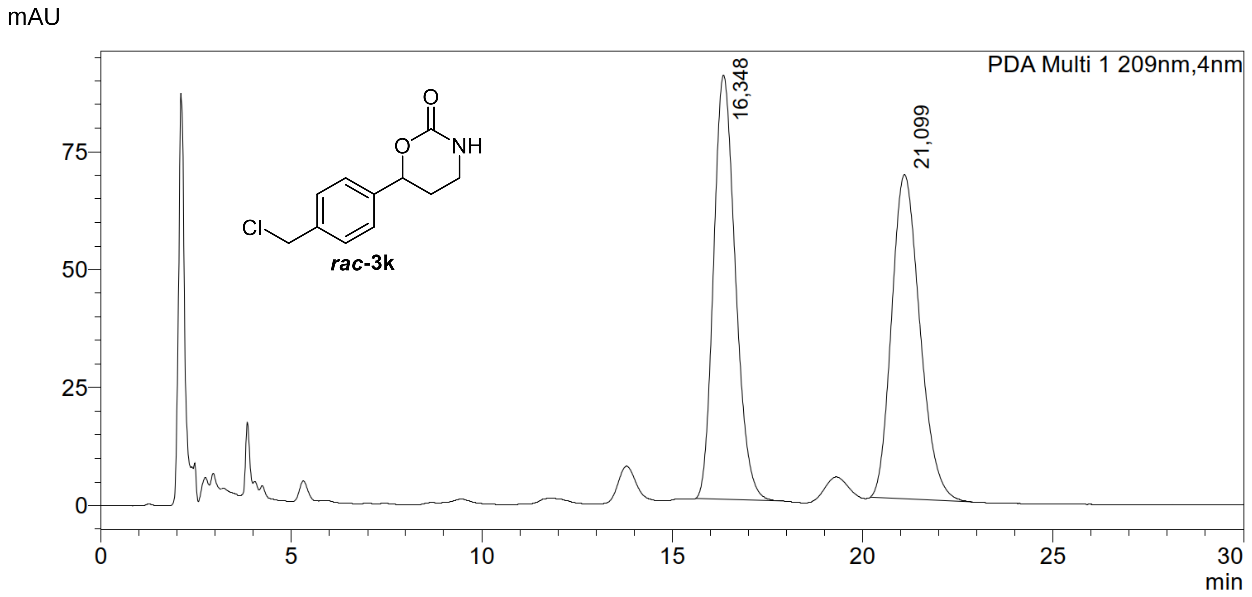

**3k**: IC-3, *n*-heptane/*i*-PrOH 50:50, 298 K, 209 nm		
Peak	Ret. time (min)	Area (%)
1	16.3	50.2
2	21.1	49.8

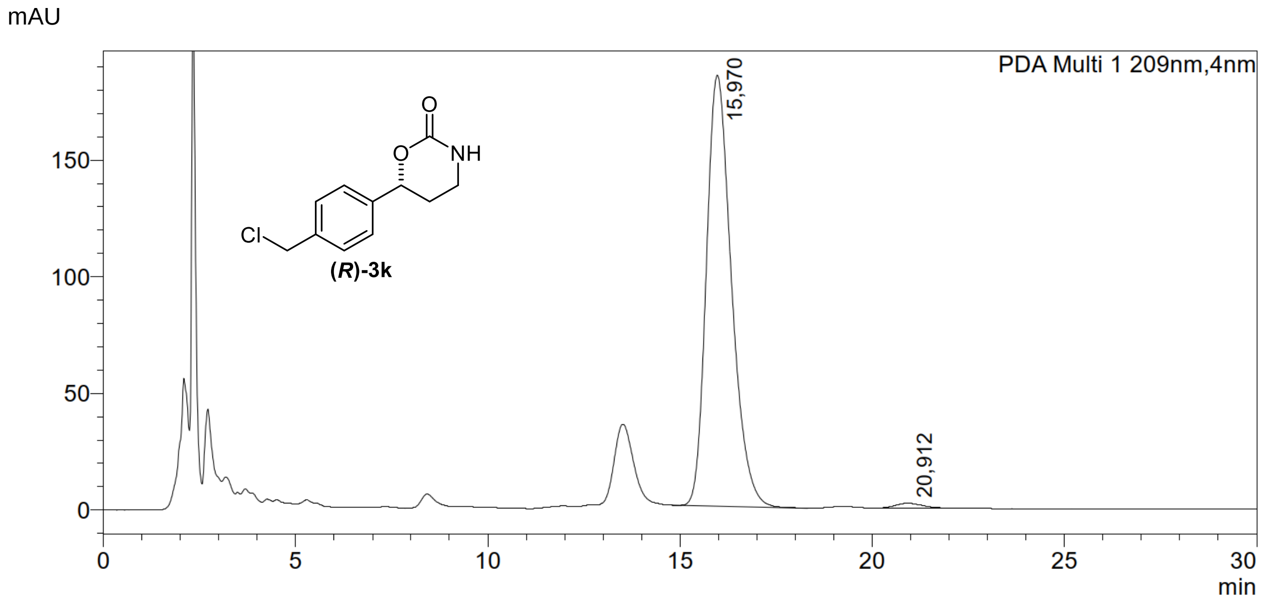

**3k**: IC-3, *n*-heptane/*i*-PrOH 50:50, 298 K, 209 nm		
Peak	Ret. time (min)	Area (%)
1	16.0	98.8
2	20.9	1.2

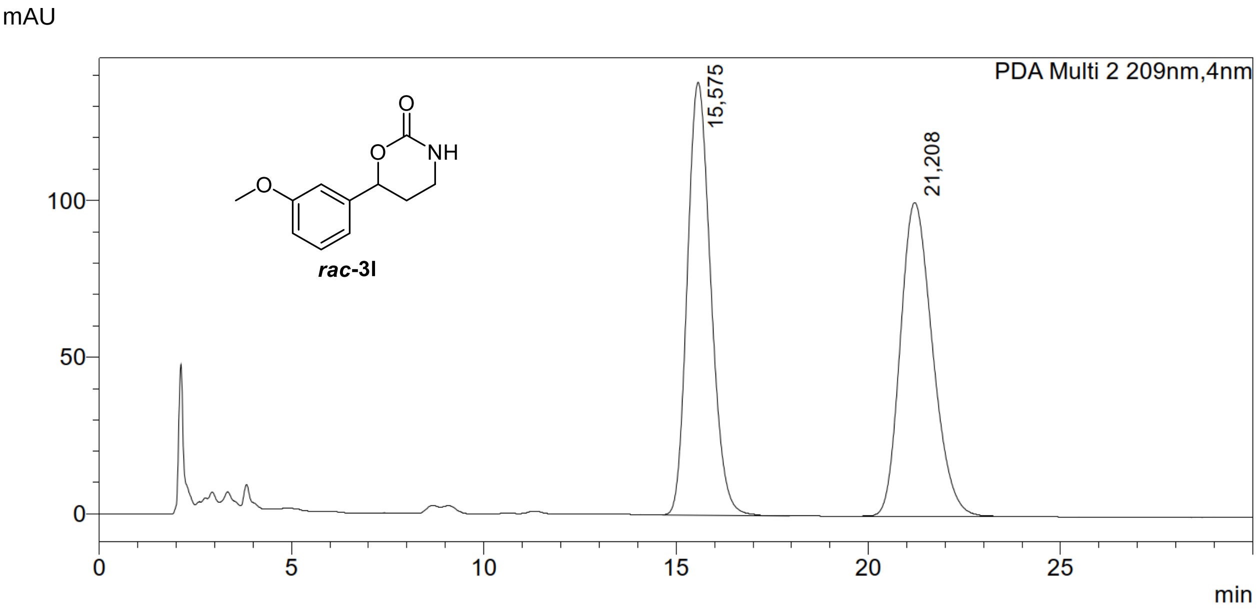

**3l**: IC-3, *n*-heptane/*i*-PrOH 50:50, 298 K, 209 nm		
Peak	Ret. time (min)	Area (%)
1	15.6	50.0
2	21.2	50.0

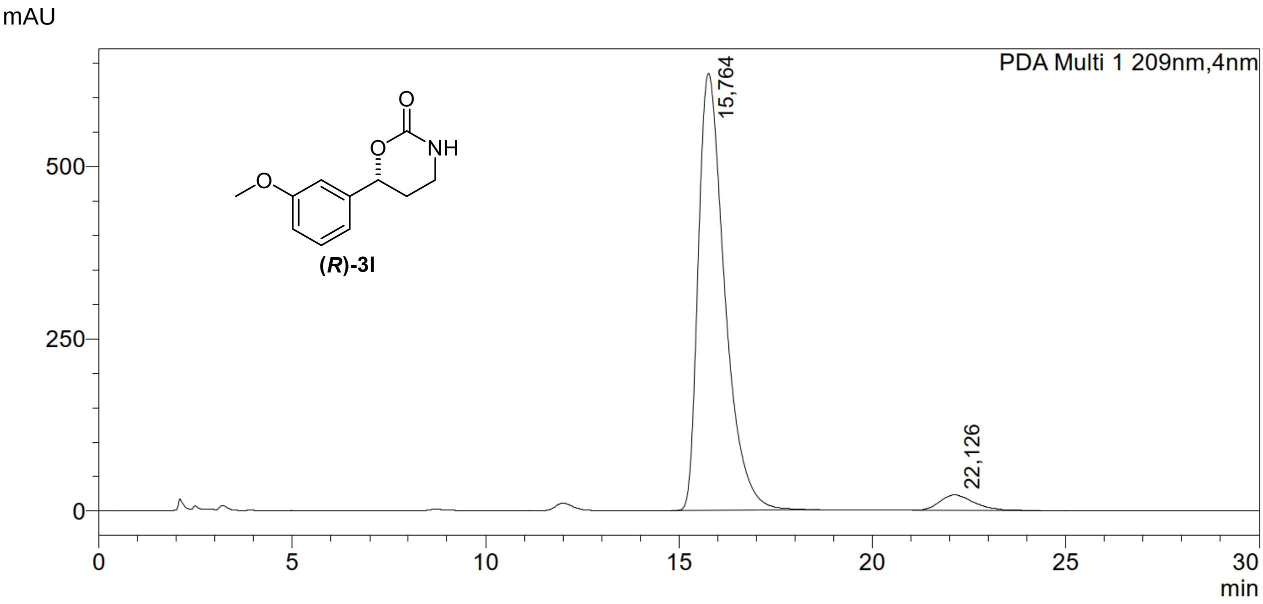

**3l**: IC-3, *n*-heptane/*i*-PrOH 50:50, 298 K, 209 nm		
Peak	Ret. time (min)	Area (%)
1	15.8	95.7
2	22.1	4.3

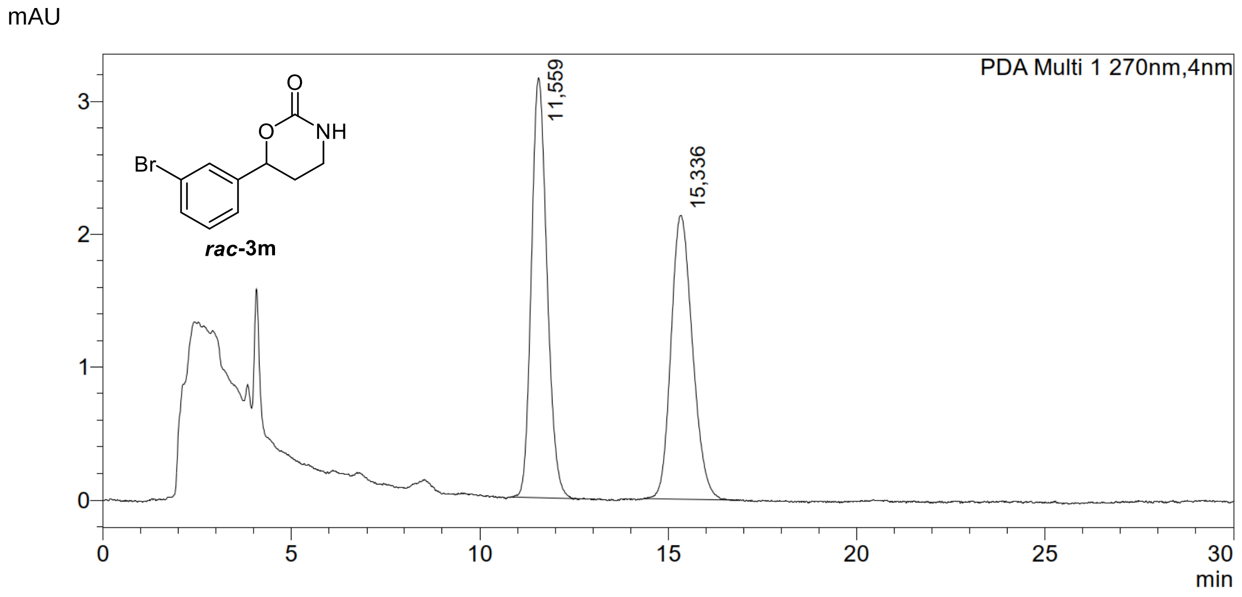

**3m**: IC-3, *n*-heptane/*i*-PrOH 50:50, 298 K, 270 nm		
Peak	Ret. time (min)	Area (%)
1	11.6	50.6
2	15.3	49.4

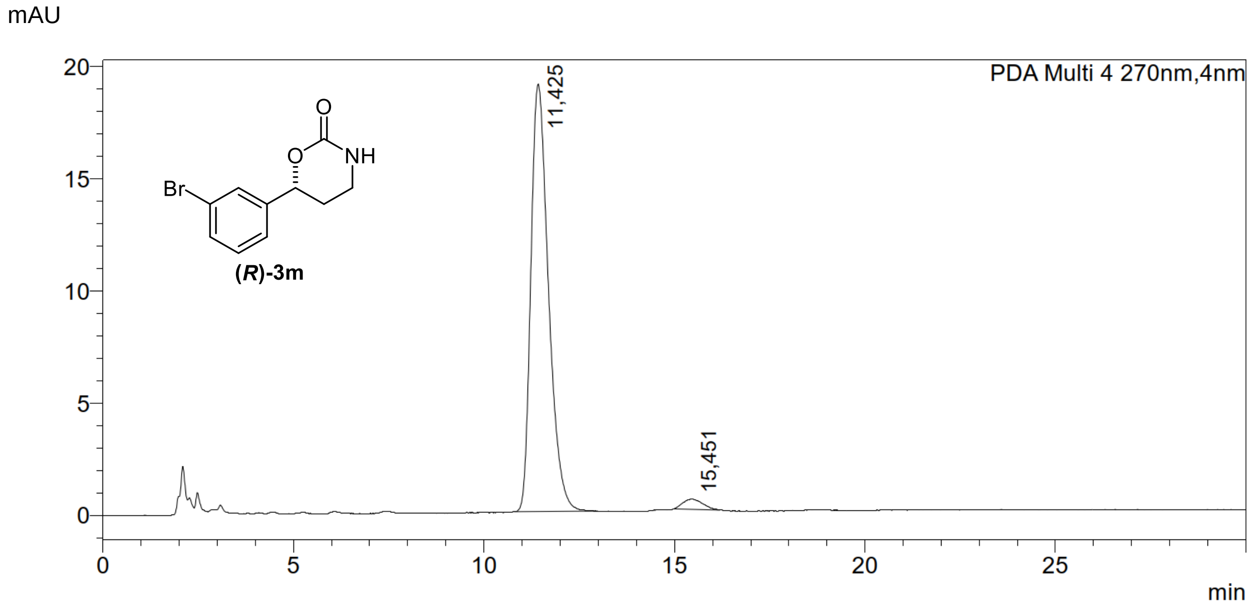

**3m**: IC-3, *n*-heptane/*i*-PrOH 50:50, 298 K, 270 nm		
Peak	Ret. time (min)	Area (%)
1	11.4	97.4
2	15.5	2.6

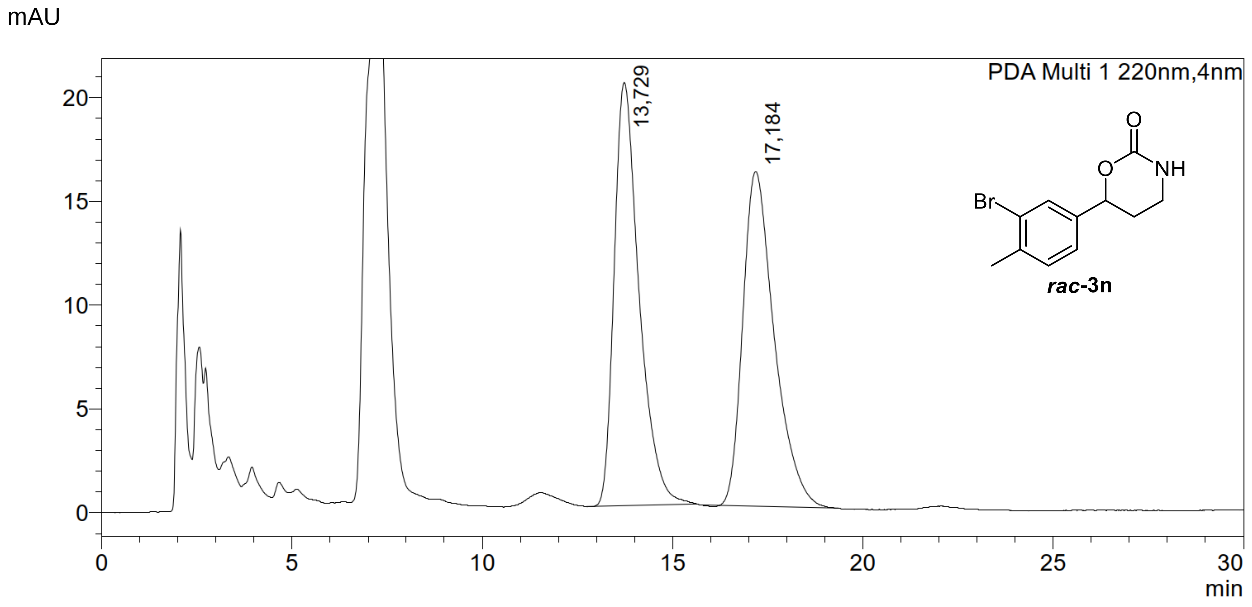

**3n**: IC-3, *n*-heptane/*i*-PrOH 50:50, 298 K, 220 nm		
Peak	Ret. time (min)	Area (%)
1	13.7	50.2
2	17.2	49.8

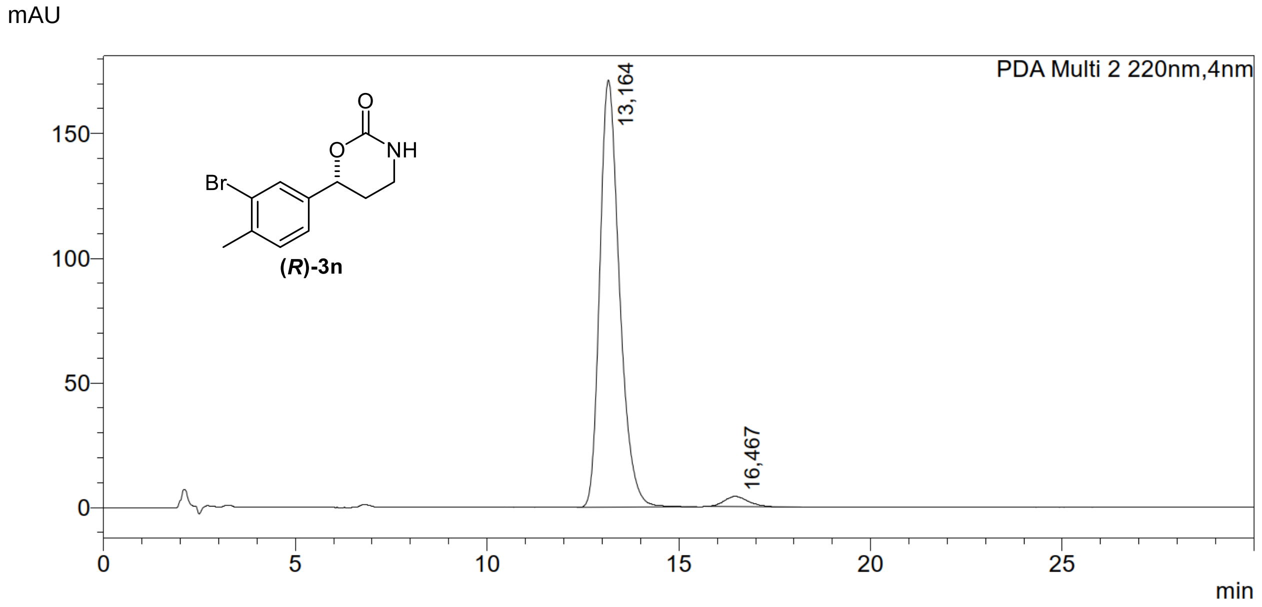

**3n**: IC-3, *n*-heptane/*i*-PrOH 50:50, 298 K, 220 nm		
Peak	Ret. time (min)	Area (%)
1	13.2	97.1
2	16.5	2.9

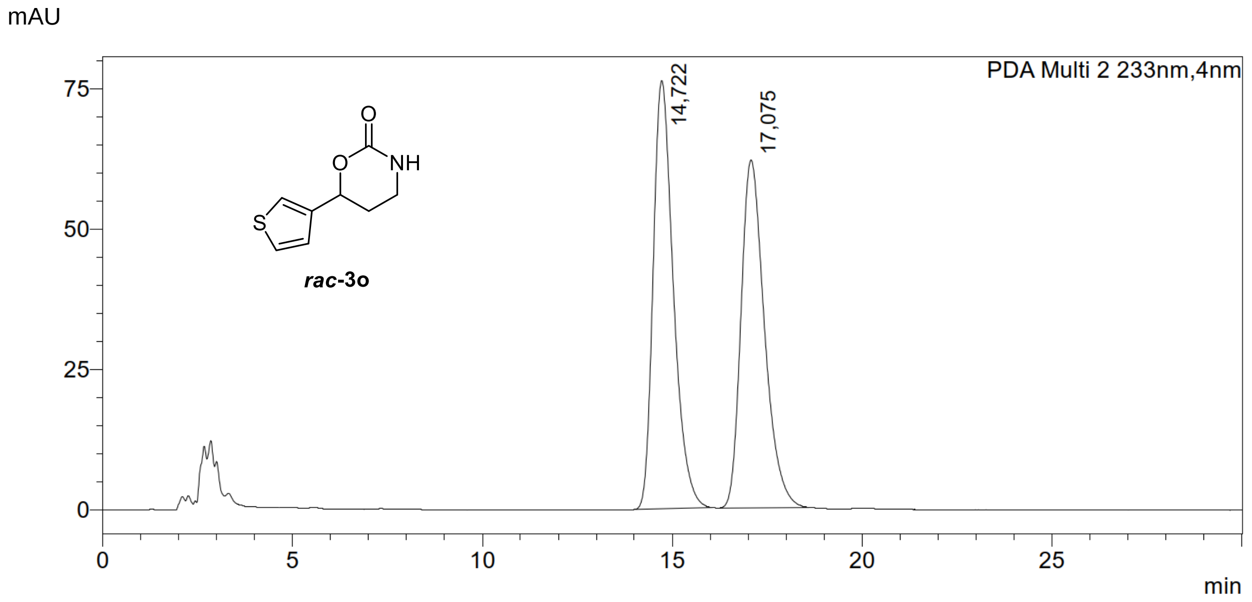

**3o**: IC-3, *n*-heptane/*i*-PrOH 50:50, 298 K, 233 nm		
Peak	Ret. time (min)	Area (%)
1	14.7	50.8
2	17.1	49.2

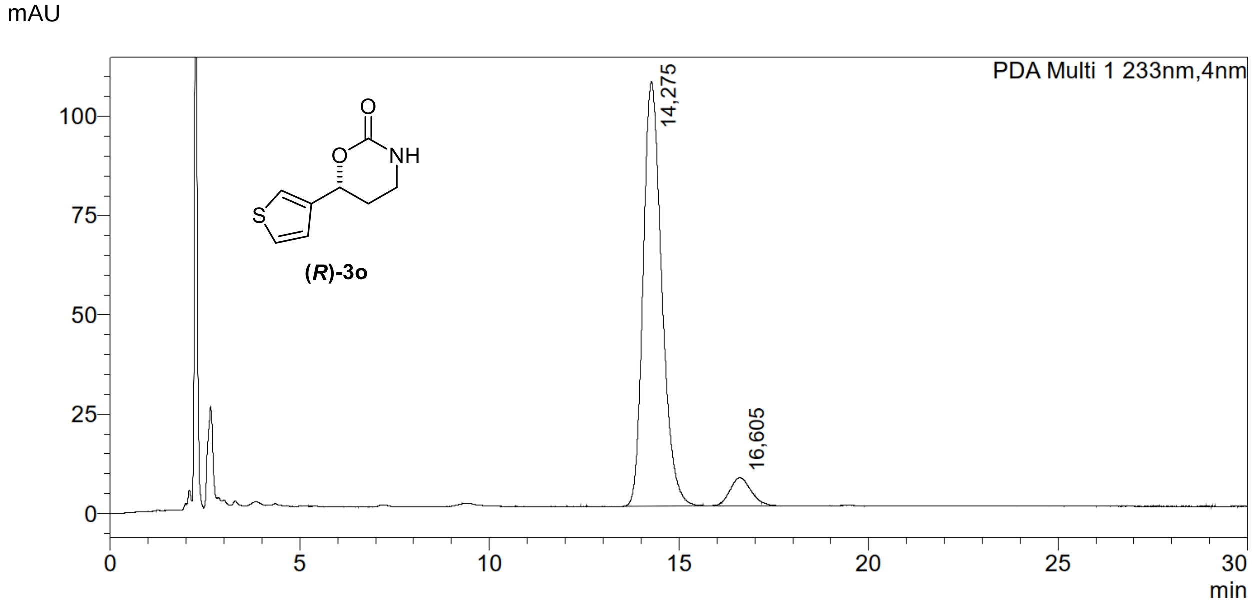

**3o**: IC-3, *n*-heptane/*i*-PrOH 50:50, 298 K, 233 nm		
Peak	Ret. time (min)	Area (%)
1	14.3	93.0
2	16.6	7.0

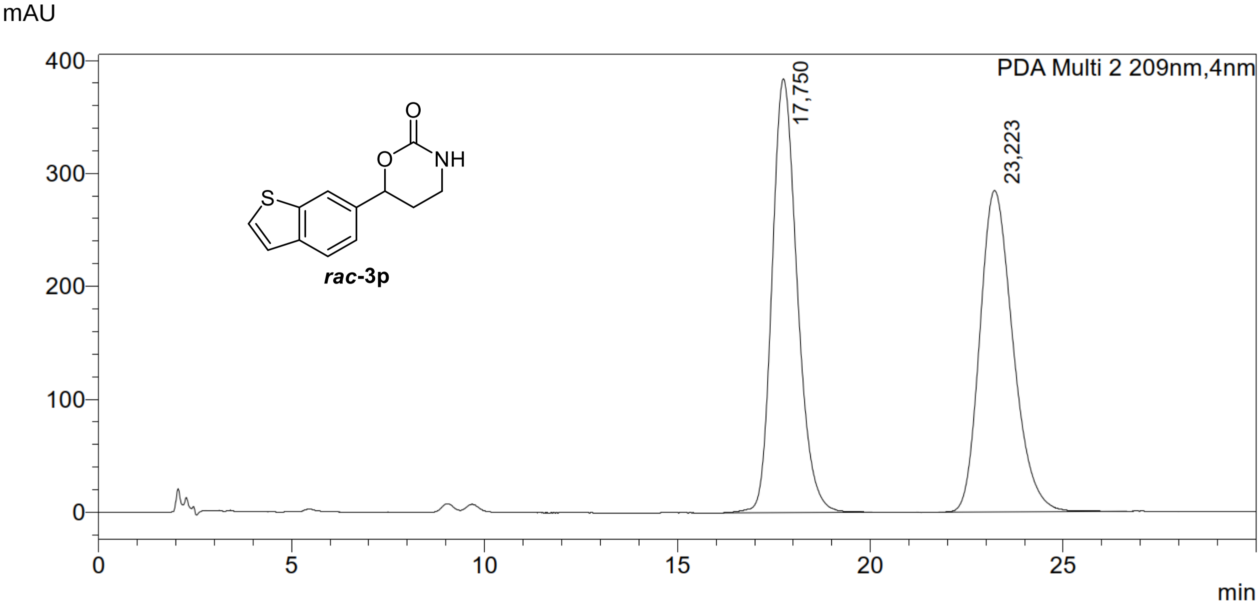

**3p**: IC-3, *n*-heptane/*i*-PrOH 50:50, 298 K, 209 nm		
Peak	Ret. time (min)	Area (%)
1	17.8	50.5
2	23.2	49.5

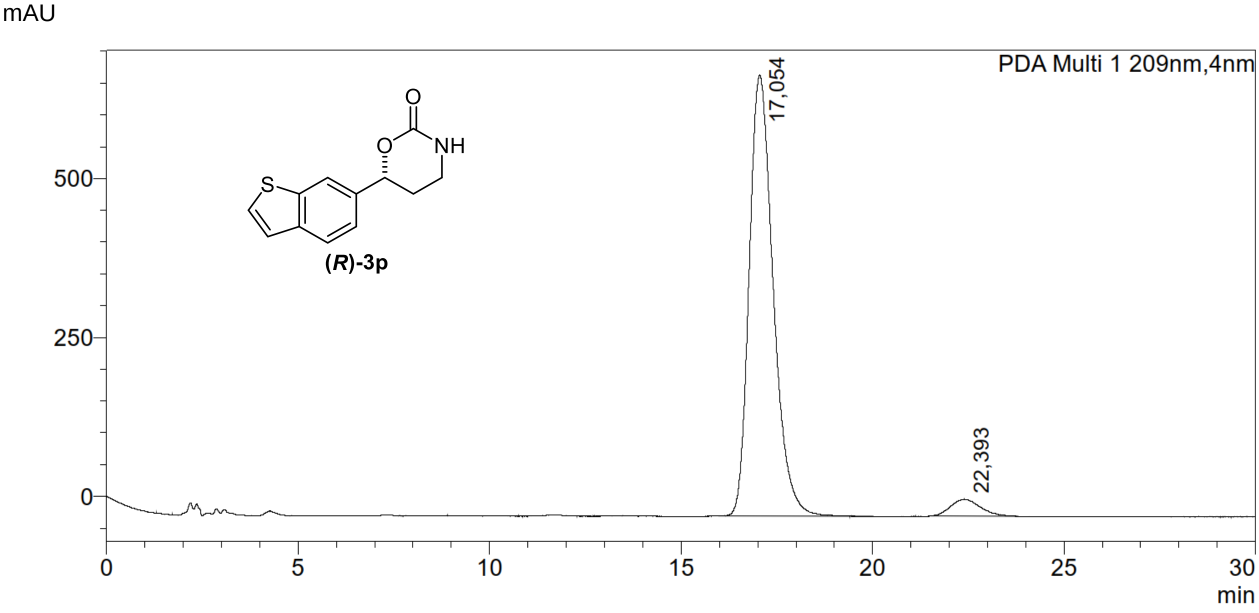

**3p**: IC-3, *n*-heptane/*i*-PrOH 50:50, 298 K, 209 nm		
Peak	Ret. time (min)	Area (%)
1	17.1	95.5
2	22.4	4.5

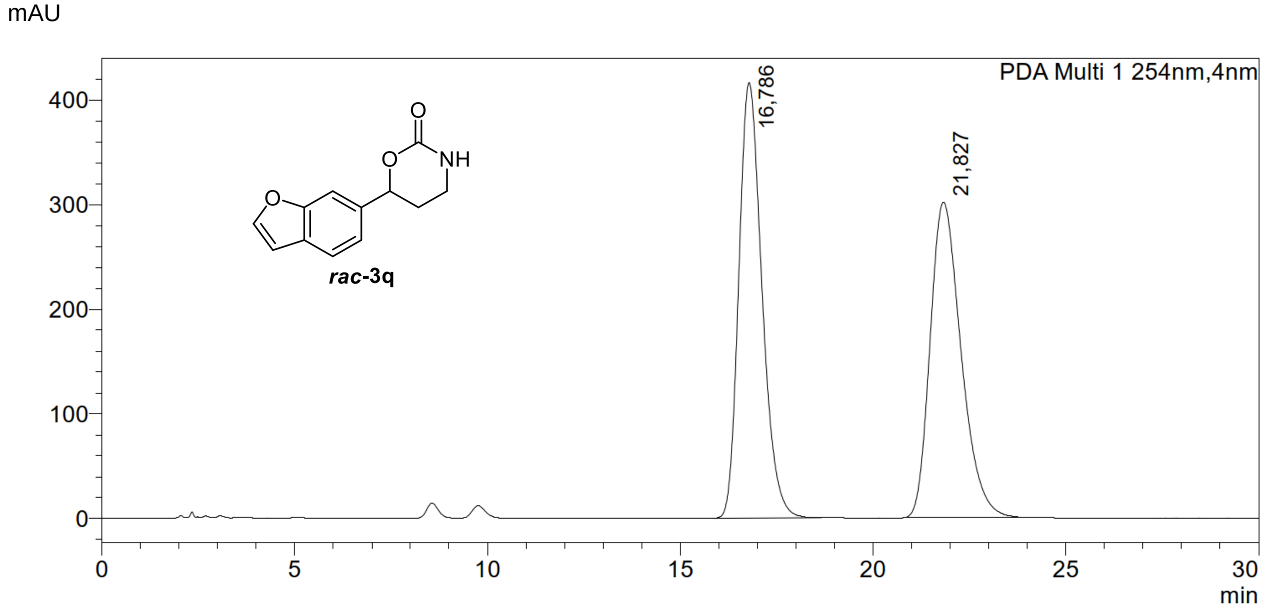

**3q**: IC-3, *n*-heptane/*i*-PrOH 50:50, 298 K, 254 nm		
Peak	Ret. time (min)	Area (%)
1	16.8	50.8
2	21.8	49.2

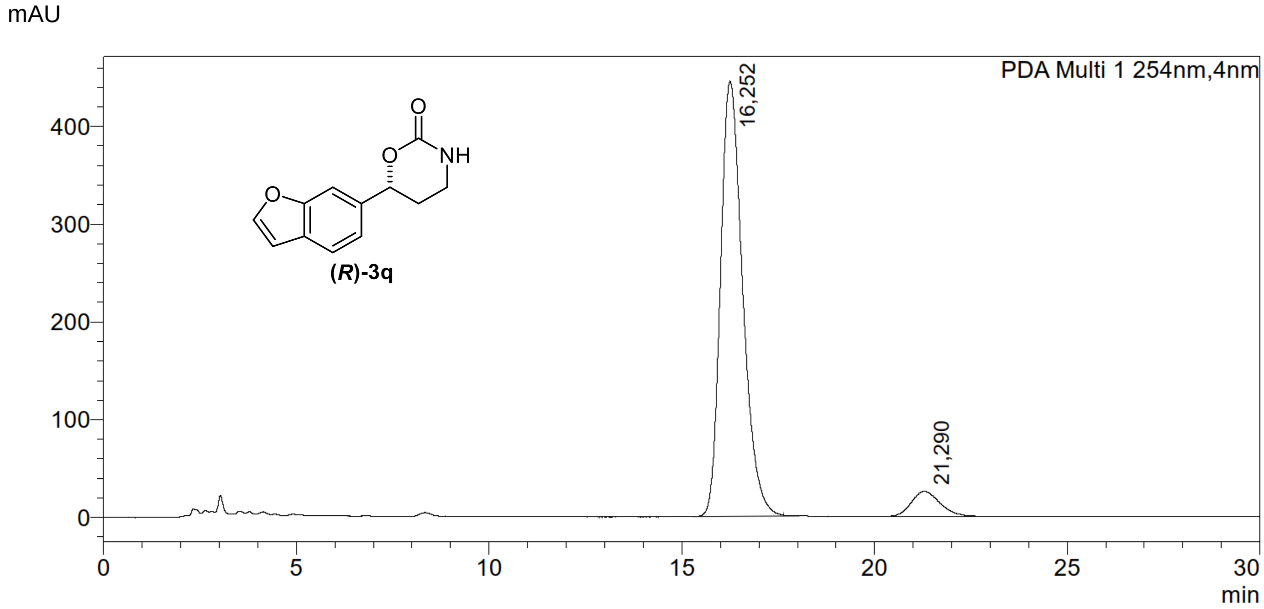

**3q**: IC-3, *n*-heptane/*i*-PrOH 50:50, 298 K, 254 nm		
Peak	Ret. time (min)	Area (%)
1	16.3	93.0
2	21.3	7.0

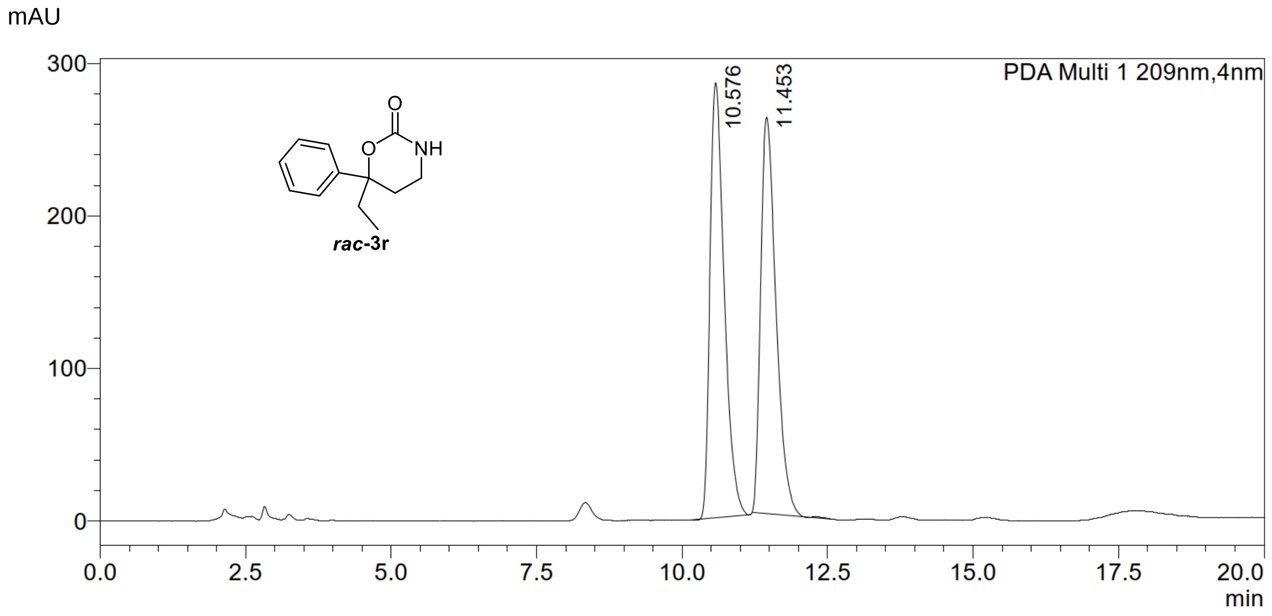

**3r**: IE-3, *n*-heptane/*i*-PrOH 80:20, 298 K, 209 nm		
Peak	Ret. time (min)	Area (%)
1	10.6	50.4
2	11.5	49.6

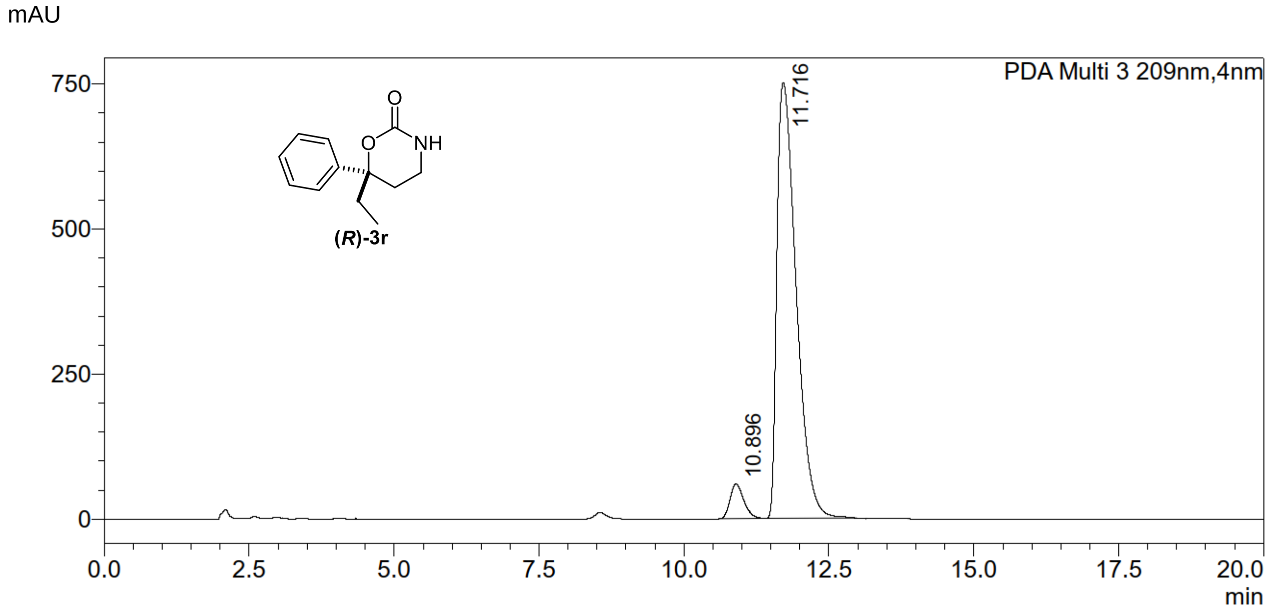

**3r**: IE-3, *n*-heptane/*i*-PrOH 80:20, 298 K, 209 nm		
Peak	Ret. time (min)	Area (%)
1	10.9	5.3
2	11.7	94.7

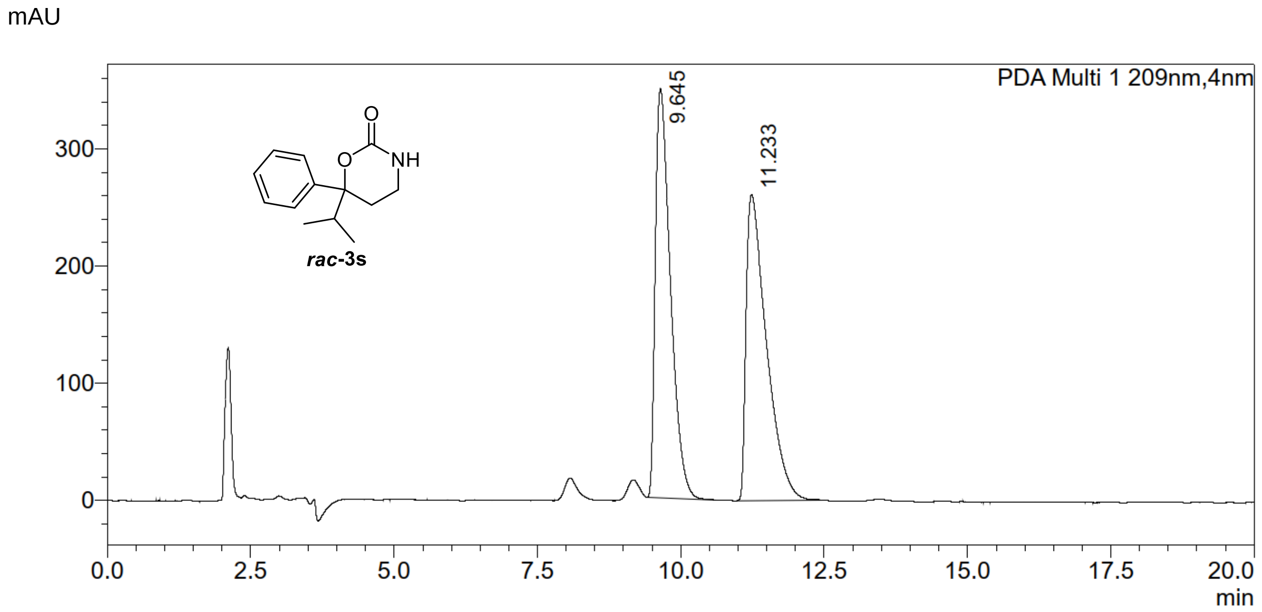

**3s**: IE-3, *n*-heptane/*i*-PrOH 80:20, 298 K, 209 nm		
Peak	Ret. time (min)	Area (%)
1	9.6	49.8
2	11.2	50.2

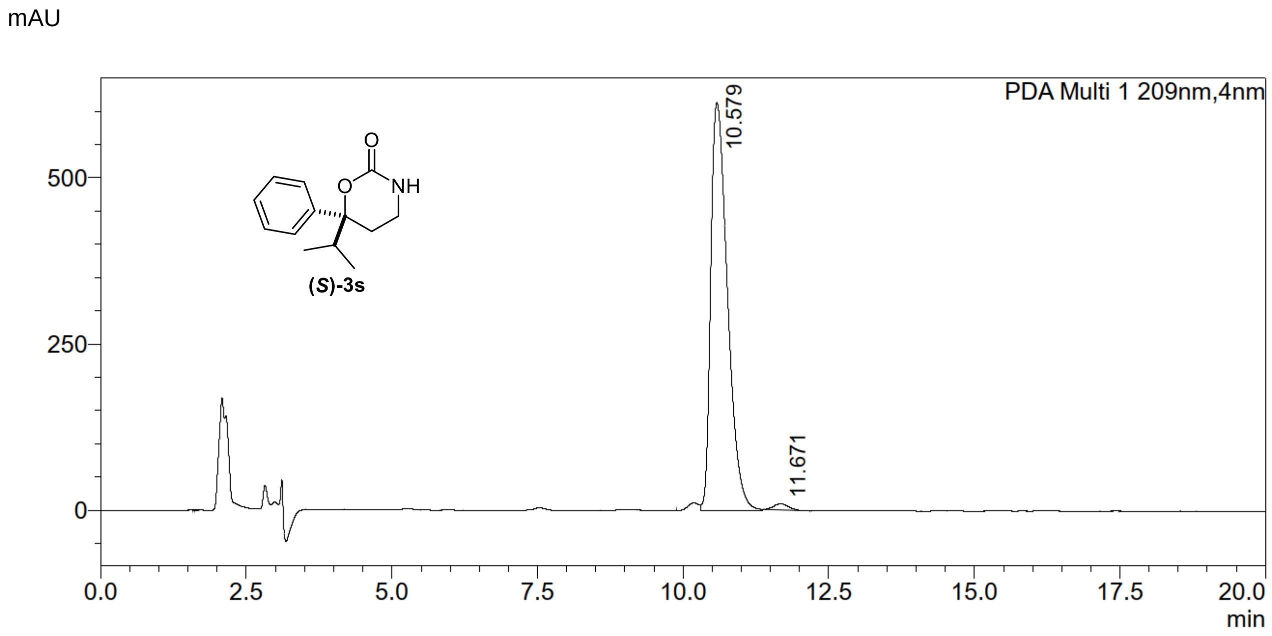

**3s**: IE-3, *n*-heptane/*i*-PrOH 80:20, 298 K, 209 nm		
Peak	Ret. time (min)	Area (%)
1	10.6	98.7
2	11.7	1.3

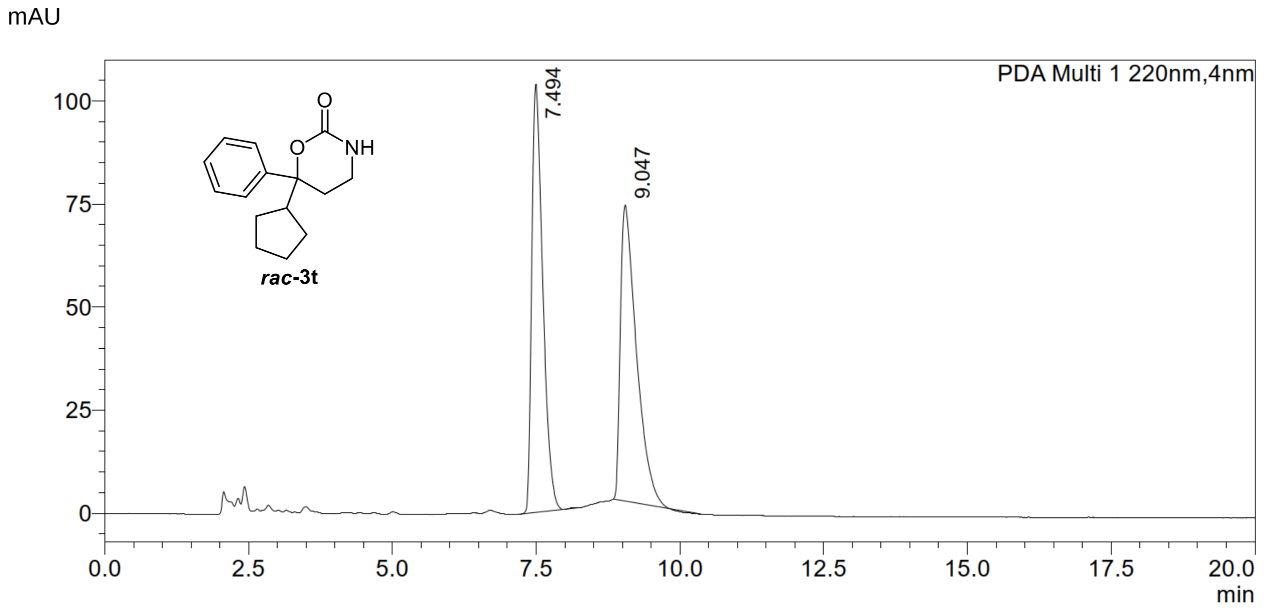

**3t**: ID-3, *n*-heptane/*i*-PrOH 80:20, 298 K, 220 nm		
Peak	Ret. time (min)	Area (%)
1	7.5	49.3
2	9.0	50.7

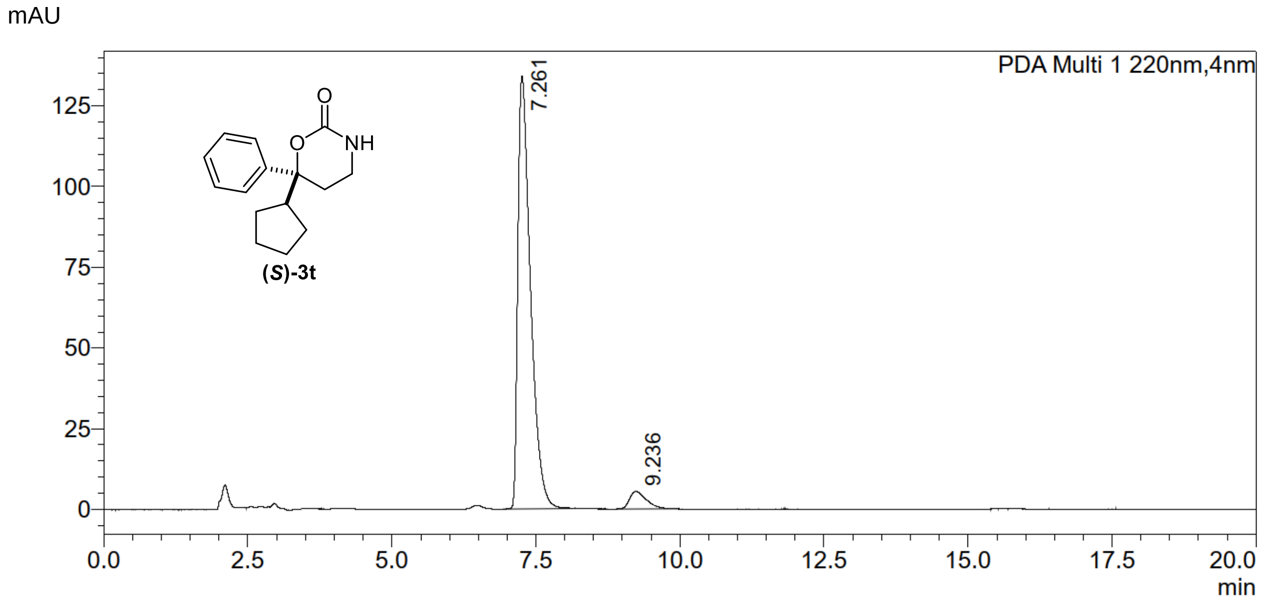

**3t**: ID-3, *n*-heptane/*i*-PrOH 80:20, 298 K, 220 nm		
Peak	Ret. time (min)	Area (%)
1	7.3	94.8
2	9.2	5.2

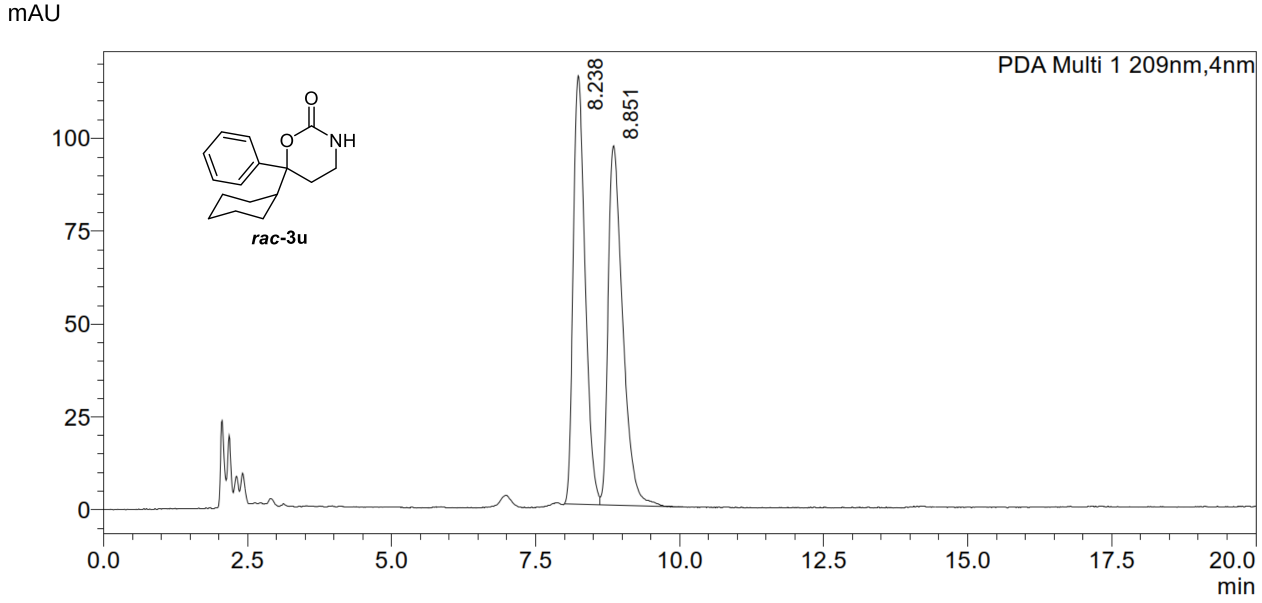

**3u**: ID-3, *n*-heptane/*i*-PrOH 80:20, 298 K, 209 nm		
Peak	Ret. time (min)	Area (%)
1	8.2	49.4
2	8.9	50.6

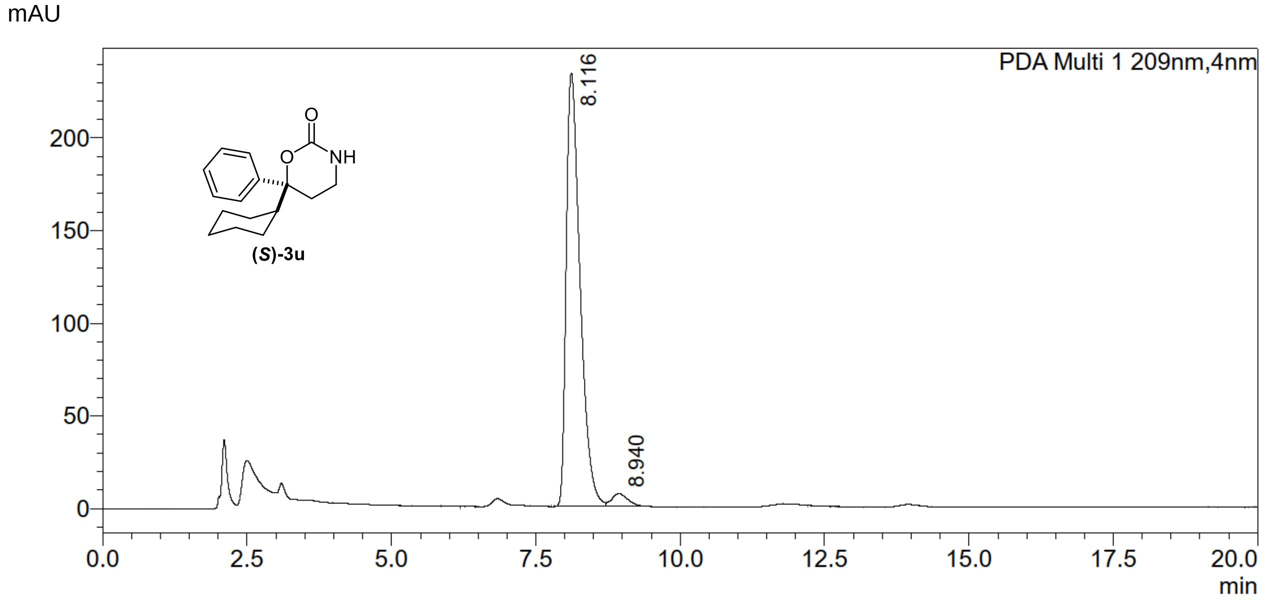

**3u**: ID-3, *n*-heptane/*i*-PrOH 80:20, 298 K, 209 nm		
Peak	Ret. time (min)	Area (%)
1	8.1	96.7
2	8.9	3.3

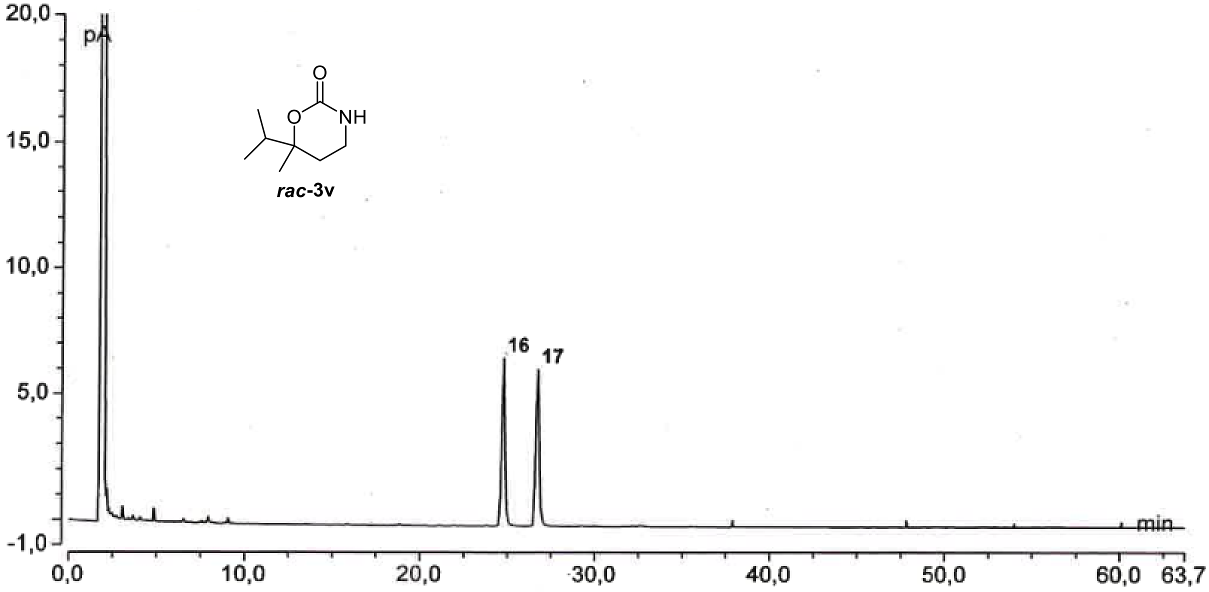

**3v**: 30.0 m BGB-174, injection temperature: 220 °C, 170 °C iso 30 min, 240 °C iso 10 min, 0.6 bar H_2		
Peak	Ret. time (min)	Area (%)
16	24.7	50.1
17	26.7	49.9

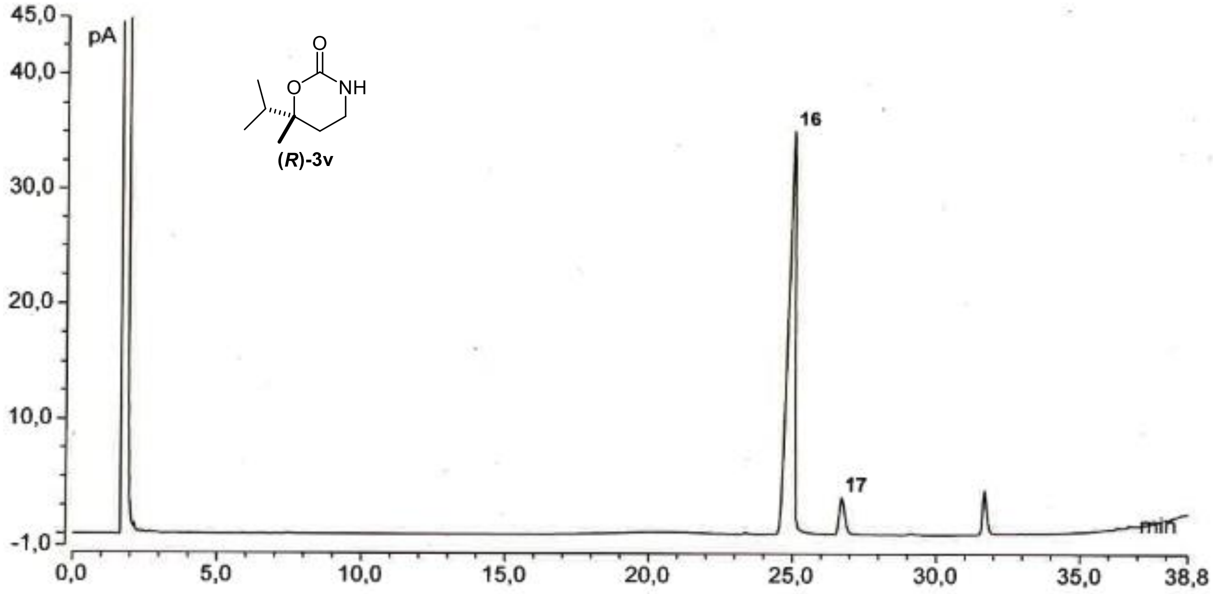

**3v**: 30.0 m BGB-174, injection temperature: 220 °C, 170 °C iso 30 min, 240 °C iso 10 min, 0.6 bar H_2		
Peak	Ret. time (min)	Area (%)
16	24.9	93.2
17	26.7	6.8

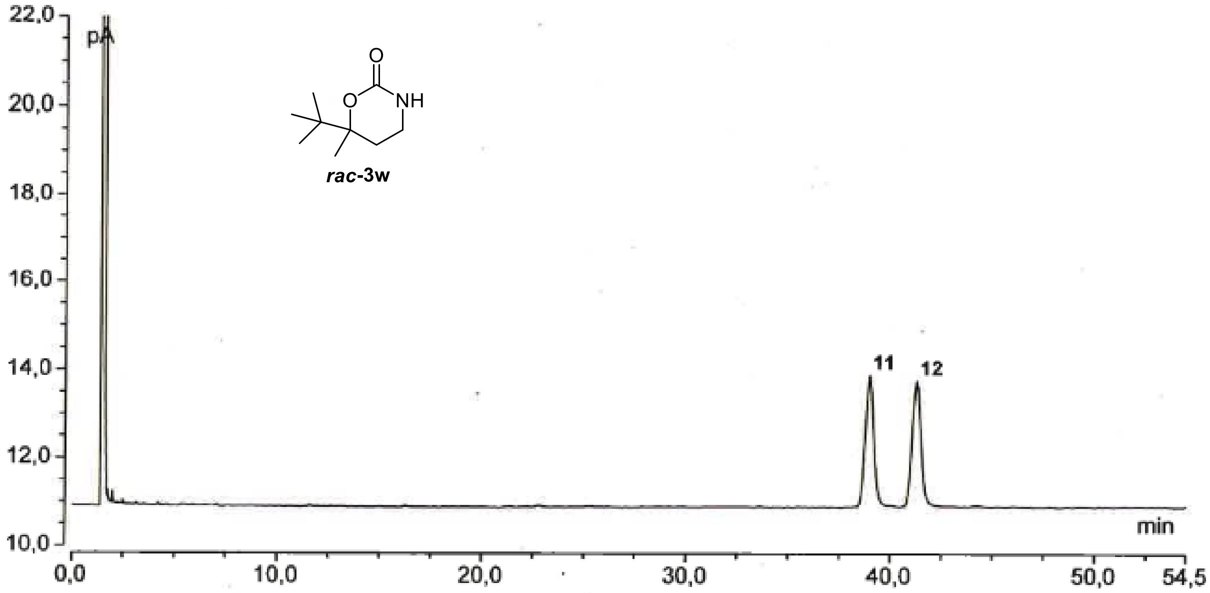

**3w**: 25.0 m Hydrodex beta-TBDAc, injection temperature: 220 °C, 150 °C iso 45 min, 8°C/min, 220 °C, 0.6 bar H_2		
Peak	Ret. time (min)	Area (%)
11	38.9	50.3
12	41.3	49.7

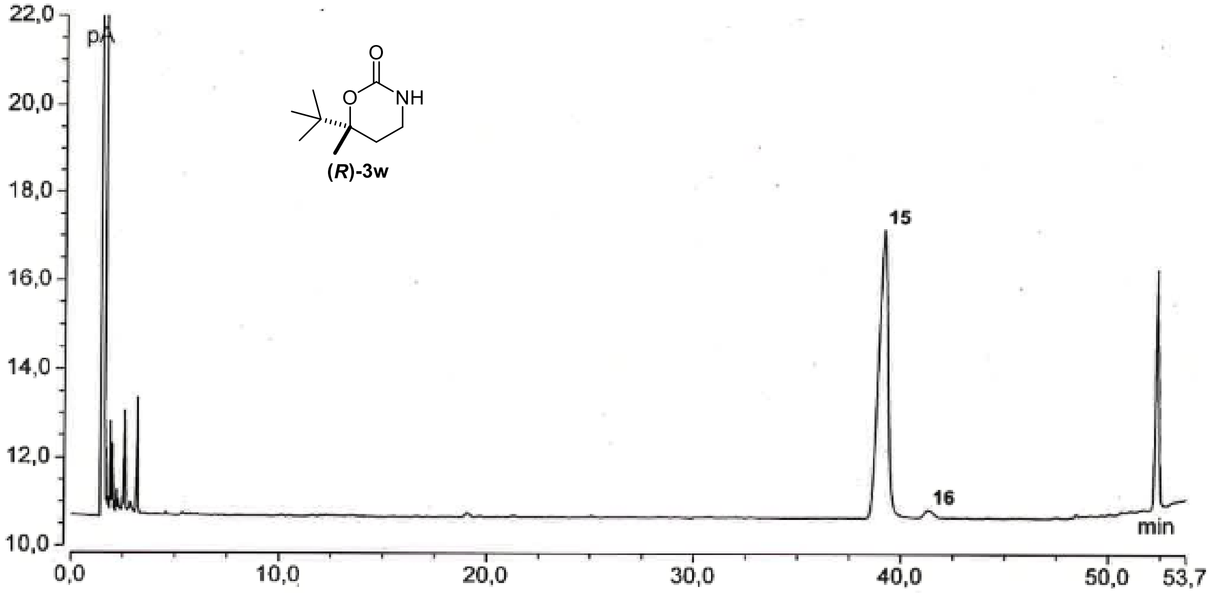

**3w**: 25.0 m Hydrodex beta-TBDAc, injection temperature: 220 °C, 150 °C iso 45 min, 8°C/min, 220 °C, 0.6 bar H_2		
Peak	Ret. time (min)	Area (%)
15	39.1	97.8
16	41.4	2.2

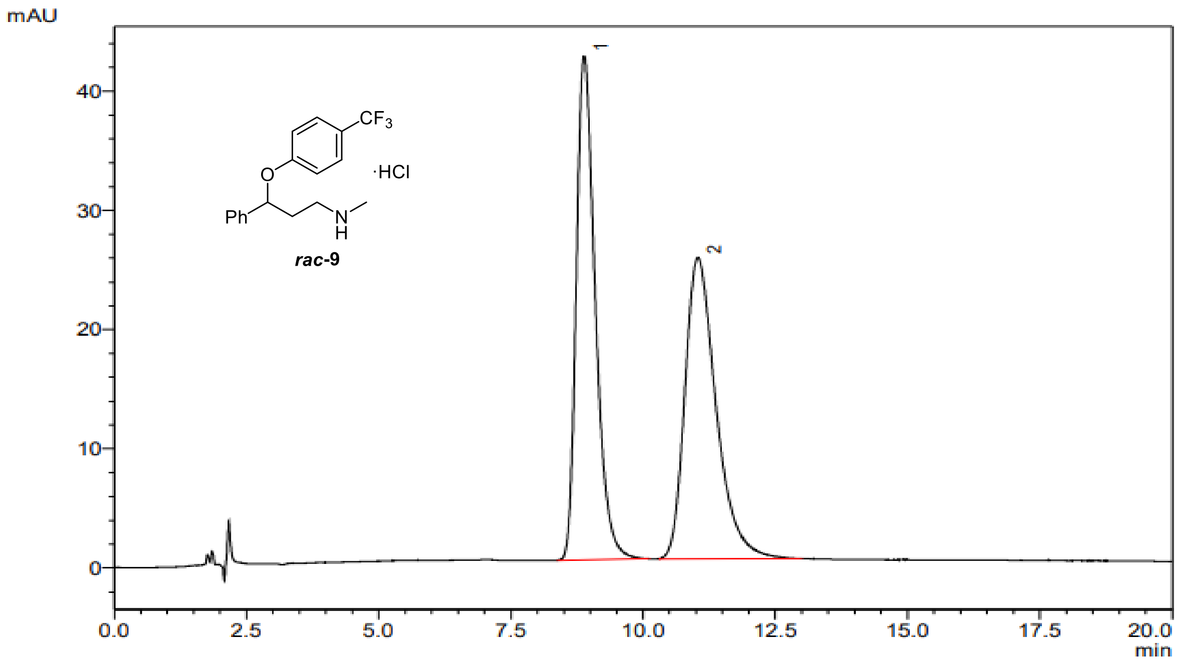

**9**: IG-3, 70:30 MeOH/20 mM NH_4HCO_3 aq. pH = 9, 298 K, 220 nm		
Peak	Ret. time (min)	Area (%)
1	8.9	50.4
2	11.0	49.6

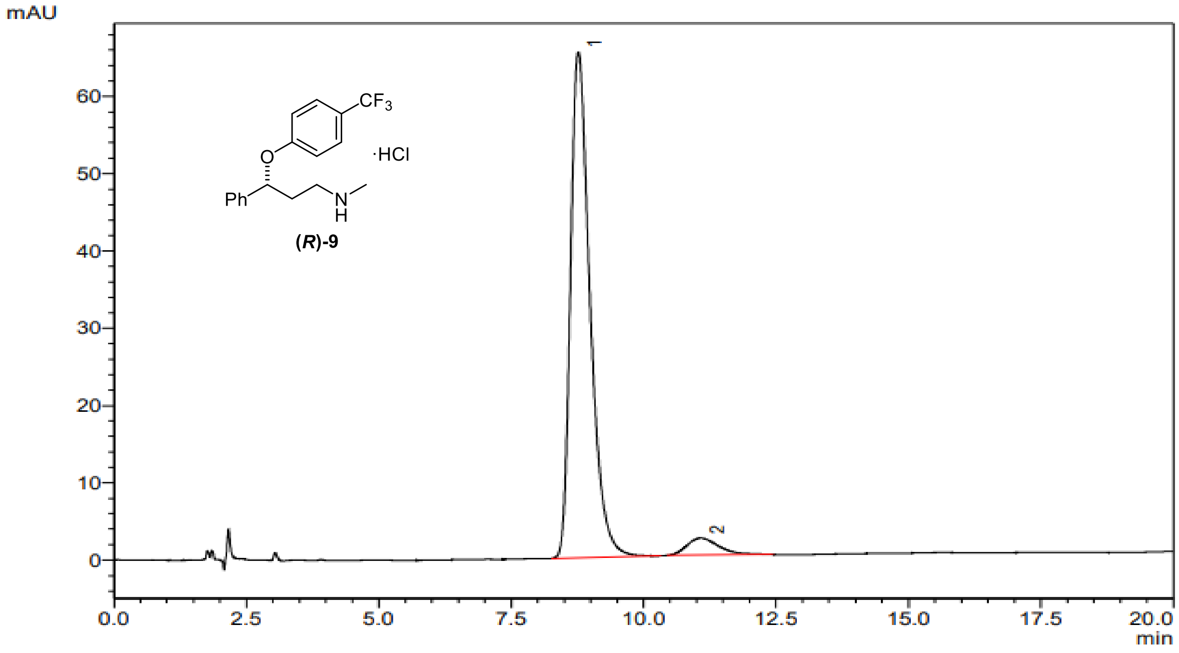

**9**: IG-3, 70:30 MeOH/20 mM NH_4HCO_3 aq. pH = 9, 298 K, 220 nm		
Peak	Ret. time (min)	Area (%)
1	8.8	96.5
2	11.1	3.5

11. References

1. Armarego, W. L. F., *Purification of laboratory chemicals*. 8th ed.; Butterworth-Heinemann: Woburn, MA, 2003.

2. Fustero, S.; Jiménez, D.; Moscardó, J.; Catalán, S.; Del Pozo, C., Enantioselective Organocatalytic Intramolecular Aza-Michael Reaction: a Concise Synthesis of (+)-Sedamine, (+)-Allosedamine, and (+)-Coniine. *Org. Lett.* **2007,** *9* (25), 5283–5286.

3. Esch, P. M.; Boska, I. M.; Hiemstra, H.; de Boer, R. F.; Speckamp, W. N., Tin Tetrachloride-Induced π-Cyclizations of Glycine Cation Equivalents to Substituted Pipecolic Acid Derivatives. *Tetrahedron* **1991,** *47* (24), 4039–4062.

4. You, Y. E.; Zhang, L.; Cui, L.; Mi, X.; Luo, S., Catalytic Asymmetric Mannich Reaction with *N*-Carbamoyl Imine Surrogates of Formaldehyde and Glyoxylate. *Angew. Chem. Int. Ed.* **2017,** *56* (44), 13814–13818.

5. Zhao, Y.; Zhu, H.; Sung, S.; Wink, D. J.; Zadrozny, J. M.; Driver, T. G., Counterion Control of *t*-BuO-Mediated Single Electron Transfer to Nitrostilbenes to Construct *N*-Hydroxyindoles or Oxindoles. *Angew. Chem. Int. Ed.* **2021,** *60* (35), 19207–19213.

6. Loev, B.; Kormendy, M. F., An Improved Synthesis of Carbamates. *J. Org. Chem.* **1963,** *28* (12), 3421–3426.

7. Cao, H.; Chen, T.; Zhou, Y.; Han, D.; Yin, S. F.; Han, L. B., Copper-Catalyzed Selective Semihydrogenation of Terminal Alkynes with Hypophosphorous Acid. *Adv. Synth. Catal.* **2014,** *356* (4), 765–769.

8. Díaz-Oviedo, C. D.; Maji, R.; List, B., The Catalytic Asymmetric Intermolecular Prins Reaction. *J. Am. Chem. Soc.* **2021,** *143* (49), 20598–20604.

9. Brégent, T.; Bouillon, J. P.; Poisson, T., Photocatalyzed E→Z Contra-thermodynamic Isomerization of Vinyl Boronates with Binaphthol. *Chem. Eur. J.* **2021,** *27* (56), 13966–13970.

10. Gonzalez-de-Castro, A.; Xiao, J., Green and Efficient: Iron-Catalyzed Selective Oxidation of Olefins to Carbonyls with O_2. *J. Am. Chem. Soc.* **2015,** *137* (25), 8206–8218.

11. Tsoukaki, A.; Skolia, E.; Triandafillidi, I.; Kokotos, C. G., Organocatalytic Synthesis of Thiiranes from Alkenes. *Eur. J. Org. Chem.* **2022,** *2022* (34).

12. Wolff, B.; Qu, Z. W.; Grimme, S.; Oestreich, M., Discrimination of the Enantiotopic Faces of Structurally Unbiased Carbenium Ions Employing a Cyclohexadiene-Based Chiral Hydride Source. *Angew. Chem. Int. Ed.* **2023,** *62* (29).

13. Kohler, D. G.; Gockel, S. N.; Kennemur, J. L.; Waller, P. J.; Hull, K. L., Palladium-Catalysed Anti-Markovnikov Selective Oxidative Amination. *Nat. Chem.* **2018,** *10* (3), 333–340.

14. Xu, S.; Ping, Y.; Li, W.; Guo, H.; Su, Y.; Li, Z.; Wang, M.; Kong, W., Enantioselective C(sp^3)–H Functionalization of Oxacycles via Photo-HAT/Nickel Dual Catalysis. *J. Am. Chem. Soc.* **2023,** *145* (9), 5231–5241.

15. Deng, C.-Q.; Deng, J., Ni-Catalyzed Asymmetric Hydrogenation of Aromatic Ketoacids for the Synthesis of Chiral Lactones. *Org. Lett.* **2022,** *24* (13), 2494–2498.

16. Dongala, E.; Mioskowski, C.; Solladié-Cavallo, M. A.; Solladié, M. G., Configuration Absolue de l'Acide β-Hydroxy β-Phényl-isocaproïque. *C. R. Acad. Sci. Paris. Série C, Sciences Chimiques [0567-6541]* **1973,** *277* (5), 251–253.

17. Gao, Y.; Sharpless, K. B., Asymmetric Synthesis of Both Enantiomers of Tomoxetine and Fluoxetine. Selective Reduction of 2,3-Epoxycinnamyl Alcohol with Red-Al. *J. Org. Chem.* **1988,** *53* (17), 4081–4084.

18. Kaib, P. S. J.; Schreyer, L.; Lee, S.; Properzi, R.; List, B., Extremely Active Organocatalysts Enable a Highly Enantioselective Addition of Allyltrimethylsilane to Aldehydes. *Angew. Chem. Int. Ed.* **2016,** *55* (42), 13200–13203.

19. Zhou, H.; Properzi, R.; Leutzsch, M.; Belanzoni, P.; Bistoni, G.; Tsuji, N.; Han, J. T.; Zhu, C.; List, B., Organocatalytic DYKAT of Si-Stereogenic Silanes. *J. Am. Chem. Soc.* **2023,** *145* (9), 4994–5000.

20. Lee, S.; Kaib, P. S. J.; List, B., Asymmetric Catalysis via Cyclic, Aliphatic Oxocarbenium Ions. *J. Am. Chem. Soc.* **2017,** *139* (6), 2156–2159.

21. Zhou, H.; Bae, H. Y.; Leutzsch, M.; Kennemur, J. L.; Bécart, D.; List, B., The Silicon–Hydrogen Exchange Reaction: A Catalytic σ-Bond Metathesis Approach to the Enantioselective Synthesis of Enol Silanes. *J. Am. Chem. Soc.* **2020,** *142* (32), 13695–13700.

22. Grell, Y.; Demirel, N.; Harms, K.; Meggers, E., Chiral Bis(oxazoline) Ligands as C_2-Symmetric Chiral Auxiliaries for the Synthesis of Enantiomerically Pure Bis-Cyclometalated Rhodium(III) Complexes. *Organometallics* **2019,** *38* (19), 3852–3859.

23. Skorobogatyi, M. V.; Ustinov, A. V.; Stepanova, I. A.; Pchelintseva, A. A.; Petrunina, A. L.; Andronova, V. L.; Galegov, G. A.; Malakhov, A. D.; Korshun, V. A., 5-Arylethynyl-2′-deoxyuridines, compounds active against HSV-1. *Org. Biomol. Chem.* **2006,** *4* (6), 1091.

24. Lee, S.; Bae, H. Y.; List, B., Can a Ketone Be More Reactive than an Aldehyde? Catalytic Asymmetric Synthesis of Substituted Tetrahydrofurans. *Angew. Chem. Int. Ed.* **2018,** *57* (37), 12162–12166.

25. Guillén, M.; Liu, S.; Díaz-Oviedo, C. D.; Klussmann, M.; List, B., Acid-Catalyzed Oxy-aminomethylation of Styrenes. *ACS Catal.* **2024,** *14* (2), 751–756.

26. Risley, J. M.; Van Etten, R. L., An ^{18}O Isotope Shift Upon ^{13}C NMR Spectra and its Application to the Study of Oxygen Exchange Kinetics. *J. Am. Chem. Soc.* **1979,** *101* (1), 252–253.

27. Das, S.; Mitschke, B.; De, C. K.; Harden, I.; Bistoni, G.; List, B., Harnessing the Ambiphilicity of Silyl Nitronates in a Catalytic Asymmetric Approach to Aliphatic β^3-Amino Acids. *Nat. Catal.* **2021,** *4* (12), 1043–1049.

28. Mayr, H.; Ofial, A. R.; Würthwein, E.-U.; Aust, N. C., NMR Spectroscopic Evidence for the Structure of Iminium Ion Pairs. *J. Am. Chem. Soc.* **1997,** *119* (52), 12727–12733.

29. Burés, J., A Simple Graphical Method to Determine the Order in Catalyst. *Angew. Chem. Int. Ed.* **2016,** *55* (6), 2028–2031.

30. Burés, J., What is the Order of a Reaction? *Top. Catal.* **2017,** *60* (8), 631–633.

31. Baxter, R. D.; Sale, D.; Engle, K. M.; Yu, J.-Q.; Blackmond, D. G., Mechanistic Rationalization of Unusual Kinetics in Pd-Catalyzed C–H Olefination. *J. Am. Chem. Soc.* **2012,** *134* (10), 4600–4606.
